# Decoupling electron transfer defines a quantitative kinetic framework for oxygen evolution catalysis

**DOI:** 10.1038/s41467-026-74392-3

**Published:** 2026-06-10

**Authors:** Haoyin Zhong, Junchen Yu, Qi Zhang, Xin Zhang, Shanlin Li, Qingli Xu, Zhi-Gen Yu, Cheng-Hao Chuang, Shibo Xi, Xiaopeng Wang, Junmin Xue

**Affiliations:** 1 Department of Materials Science and Engineering, National University of Singapore, Singapore, Singapore; 2 Institute of Materials Research, Tsinghua Shenzhen International Graduate School, Tsinghua University, Shenzhen, China; 3 National Engineering Laboratory for Modern Silk, College of Textile and Clothing Engineering, Soochow University, Suzhou, China; 4 Institute of High Performance Computing, Agency for Science, Technology and Research, Singapore, Singapore; 5 Department of Physics, Tamkang University, New Taipei City, Taiwan, ROC; 6 Institute of Sustainability for Chemical, Energy and Environment (ISCE²), Agency for Science, Technology and Research, Singapore, Singapore; 7 State Key Laboratory of Intelligent Construction and Healthy Operation and Maintenance of Deep Underground Engineering, College of Materials Science and Engineering, Sichuan University, Chengdu, China

**Keywords:** Electrocatalysis, Electrocatalysis, Hydrogen energy, Electrocatalysis

## Abstract

The oxygen evolution reaction underpins many energy conversion technologies, yet its performance is fundamentally constrained by sluggish reaction kinetics at catalyst surfaces. Current catalyst design remains largely empirical because most strategies correlate bulk structural descriptors with overall activity rather than resolving the intrinsic kinetics of elementary reaction steps. Here, we show that open-circuit voltage–pulse voltammetry can quantitatively determine the ^*^OOH formation rate, a rate-determining step in oxygen evolution. Unlike conventional electrochemical techniques, this method isolates ^*^OOH formation-related electron transfer by interrupting electron transfer from electrocatalyst to external circuit while sustaining electron supply from hydroxide ions. Coupling this descriptor with a pulse voltammetry method for quantifying ^*^OH deprotonation kinetics yields a unified, step-resolved kinetic framework that reveals how different dopants selectively accelerate either ^*^OOH formation or ^*^OH deprotonation. Fe primarily facilitates ^*^OOH formation, whereas Mn selectively promotes ^*^OH deprotonation. Guided by these insights, a rationally designed NiFeMn catalyst concurrently enhances both processes, delivering improved oxygen evolution performance. This methodology provides a practical means to quantify elementary reaction kinetics and accelerate the discovery of high-performance electrocatalysts.

## Abstract (web summary)

Oxygen evolution is central to clean energy technologies, but catalyst design is limited by difficulty in measuring individual reaction steps. Here, the authors report a kinetic framework that quantifies key oxygen evolution steps and guides catalyst design.

## Introduction

The oxygen evolution reaction (OER), a four-electron process central to water electrolysis and other renewable energy conversion technologies, continues to suffer from sluggish kinetics that necessitate the development of highly efficient electrocatalysts^1,2^. Addressing this kinetic limitation has motivated extensive efforts to design catalysts that can accelerate the multistep OER process. Over the past decade, OER catalyst design has become increasingly data- and theory-driven, encompassing strategies, such as electronic structure modulation, high-throughput screening, and machine-learning-assisted predictions^3–5^. Nevertheless, these approaches remain largely empirical because they rely on correlating bulk structural descriptors with overall activity metrics, rather than quantitatively resolving the intrinsic kinetics of the elementary reaction steps. Since the intrinsic catalytic activity is ultimately dictated by the kinetics of individual electron transfer processes, decoupling these step-specific reactions is essential to identify the fundamental kinetic bottlenecks and to establish a quantitative kinetic framework that can guide rational catalyst design.

To experimentally resolve the step-specific kinetic processes governing OER activity, it is essential to isolate and quantitatively probe each elementary electron-transfer step. For most electrocatalysts, the oxygen evolution proceeds via the adsorbate evolution mechanism (AEM), wherein a series of surface-bound oxygen intermediates, such as ^*^OH, ^*^O, ^*^OOH, and ^*^OO, are sequentially formed. Among these, the formation of the ^*^OOH intermediate, which is widely recognized as the rate-determining step (RDS) in the AEM, represents the most critical and challenging process to capture^6–9^. As a short-lived intermediate, ^*^OOH exists only transiently on the catalyst surface during the OER. Consequently, the critical challenge in quantifying the ^*^OOH formation rate lies in monitoring its transient concentration under operando conditions. In principle, changes in the ^*^OOH concentration can be inferred by observing bond formation and cleavage and the associated electron transfer processes. Bond formation and cleavage are typically detected using spectroscopic chemical probing techniques, such as in situ shell-isolated nanoparticle-enhanced Raman spectroscopy (SHINERS) and Fourier transform infrared spectroscopy (FTIR)^10–15^. However, owing to the subpicosecond timescale of ^*^OOH formation, current spectroscopic methods lack the temporal resolution necessary for accurate quantification of its dynamic concentration^16^. Given these spectroscopic limitations, alternative approaches have been employed to gain insight into OER kinetics through indirect means. To date, Tafel analysis has been widely employed to explore the mechanism of electron transfer kinetics. For Tafel analysis, the overpotential (*η*) versus the logarithm of the current density (log *J*) is plotted to capture the overall kinetics of the OER, including all four electron transfer steps, using Eqs. 1–4^17–19^. Unfortunately, the electron transfer step that produces ^*^OOH overlaps with the prior step in which its precursor ^*^O is generated and the subsequent deprotonation of ^*^OOH^1,20^, making it difficult to isolate and quantify the electron transfer rate of ^*^OOH formation alone. Currently, an experimental method that allows direct, quantitative measurement of the ^*^OOH formation rate under realistic operating conditions is lacking.

Here we develop a facile electrochemical method to directly quantify the ^*^OOH formation rate, thereby enabling the establishment of a kinetic framework that bridges elementary electron transfer kinetics and catalyst design. With NiOOH as the model material, we demonstrate that when an open-circuit voltage (OCV) procedure is implemented following charging the electrocatalyst, electron transfer from the electrocatalyst to the external circuit is blocked, whereas electron transfer from the electrolyte to the electrocatalyst associated with ^*^OOH formation/^*^OO evolution is maintained. Further investigation revealed that the ^*^OOH formation rate remained stable during a brief OCV interval. Based on these findings, an OCV–pulse voltammetry (OCV‒PV) electrochemical method is developed to quantify electron transfer associated with ^*^OOH formation. As a result, the rates of ^*^OOH formation from NiOOH under different charging biases are identified, e.g., 4.20E-8 mmol cm^−2^ s^−1^ at 1.503 V.

Combined with PV measurement that probes the ^*^OH deprotonation kinetics, this integrated strategy enables parallel quantification of ^*^OOH formation and ^*^OH deprotonation. This, in turn, yields a kinetic framework that elucidates how different dopant elements modulate specific electron transfer steps in the OER pathway. Within this framework, Fe is identified to primarily facilitate ^*^OOH formation, while Mn preferentially promotes ^*^OH deprotonation. Guided by these insights, Fe and Mn are co-incorporated into NiOOH to synergistically accelerate both ^*^OOH formation and ^*^OH deprotonation, achieving substantially enhanced OER activity. This methodological advance not only clarifies the electron transfer mechanism governing the OER but also establishes a practical kinetic descriptor for evaluating and screening next-generation electrocatalysts.

## Theoretical analysis for quantifying the ^*^OOH formation rate

**Fig. 1:**
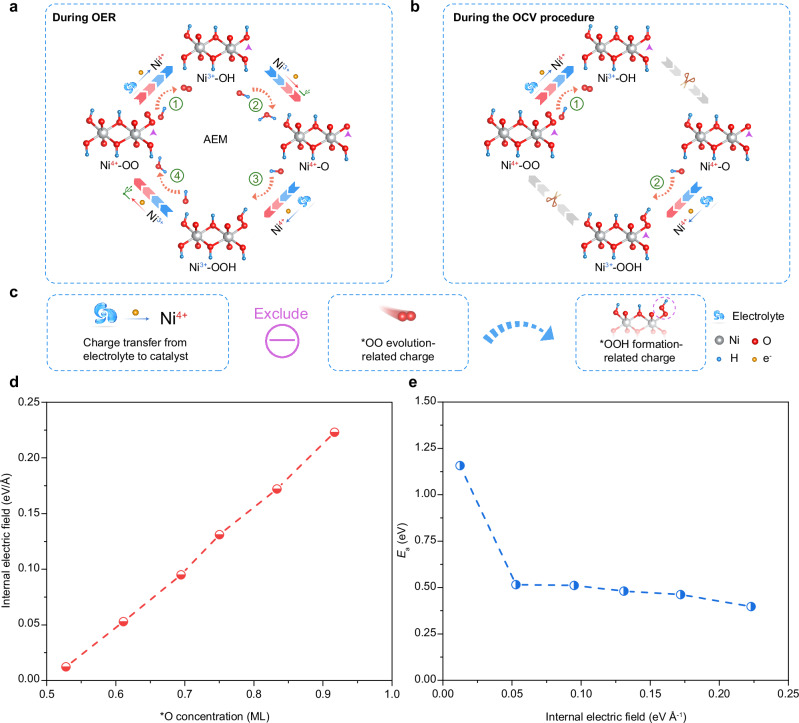
Theoretical analysis for quantifying the ^*^OOH formation rate. **a** Schematic of the adsorbate evolution mechanism (AEM) pathway, in which a series of surface-bound oxygen intermediates, such as ^*^OH, ^*^O, ^*^OOH, and ^*^OO, are sequentially formed. **b** Schematic of electron transfer upon conducting an open-circuit voltage (OCV) procedure. The scissor icon indicates that electron transfer from the electrocatalyst to the external circuit is blocked. **c** Schematic of the method used to selectively isolate the ^*^OOH formation-related charge from the total charge transferred from the electrolyte to the catalyst. **d** The effect of ^*^O concentration on internal electric field. **e** The effect of internal electric field on ^*^OOH formation energy barrier, where *E*_a_ denotes the activation energy. Source data are provided as a Source data file.

Our proposed approach for quantifying ^*^OOH is based on metal redox chemistry along the AEM pathway, where the Ni valence state converts between 3+ and 4+ in association with each OER step. The AEM pathway proceeds via the formation of four oxygen intermediates, ^*^OH, ^*^O, ^*^OOH, and ^*^OO (Fig. 1a). Of these, ^*^O would be the most abundant intermediate during the OER since ^*^OOH formation is recognized as the RDS^6–8,21^. Considering the Ni^3+^/Ni^4+^ redox couple in the AEM route, two kinds of electron transfer pathways can be classified in the OER: a) electron transfer from the electrocatalyst to the external circuit, which involves ^*^OH deprotonation and ^*^OOH deprotonation and is associated with an increase in the Ni valence state from 3+ to 4+ (the second and fourth steps in Fig. 1a, b) electron transfer from the electrolyte (OH^-^) to the electrocatalyst, leading to a reduction in the Ni valence from 4+ to 3+ corresponding to ^*^OOH formation and ^*^OO evolution (the first and third steps in Fig. 1a)^7^. Under an applied bias, the Ni active site transitions between the Ni^3+^ and Ni^4+^ states during the OER process, reaching an average Ni^3+x^ valence state (0 < *x* < 1).

When an OCV procedure is conducted after NiOOH charging under an applied bias, electron transfer from the electrocatalyst to the external circuit associated with ^*^OH deprotonation/^*^OOH deprotonation is immediately blocked (Fig. 1b)^22^. However, during this stage, a portion of the Ni sites remain in the oxidized Ni^4+^ state and are thermodynamically unstable in the absence of an applied bias. Theoretically, oxidized Ni^4+^ can be reduced to Ni^3+^ by accepting electrons from OH^−^ ions, primarily through the ^*^OOH formation pathway, given that ^*^O is the most abundant intermediate. This provides an opportunity to quantify the ^*^OOH formation rate by measuring the total charge transferred during the OCV and subtracting the ^*^OO evolution-related charge (Fig. 1c). Therefore, the continued ^*^OOH formation under OCV is thermodynamically driven by the spontaneous relaxation of the pre-charged, highly oxidized NiOOH surface toward a lower-energy state with reduced Ni valence, rather than by a sustained external bias.

To further investigate ^*^OOH formation on charged NiOOH, density functional theory (DFT) calculations were performed on NiOOH model samples with varying surface ^*^O concentrations (Fig. 1d, e, and Supplementary Figs. 1–5). The results indicate that when the Ni active sites are charged, a surface electric field is generated, promoting ^*^OOH formation by attracting OH^⁻^ ions generated from H_2_O dissociation (Fig. 1d). Notably, the energy barrier (*E*_a_) for ^*^OOH formation decreases sharply to below 0.75 eV (Fig. 1e), which suggests that ^*^OOH formation becomes kinetically favourable on the charged NiOOH surface^8,23^. These results provide theoretical support for continued ^*^OOH formation on charged NiOOH during OCV.

## Results

### ^*^OOH formation during the OCV procedure

**Fig. 2:**
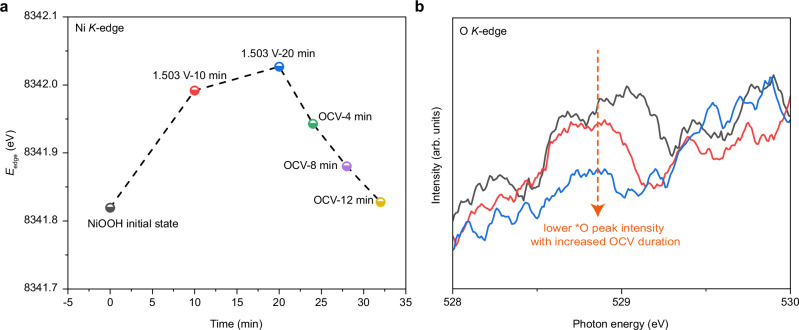
Continued formation of ^*^OOH during the OCV. **a** Photon energy of the half-height position (*E*_edge_) of NiOOH obtained from the normalized in situ Ni *K*-edge XAS spectra (Supplementary Fig. 6) under charging and OCV procedures. **b** In situ O *K*-edge XAS spectra of the change in ^*^O peak intensity for NiOOH during the OCV procedure. Source data are provided as a Source data file.

Since ^*^OOH formation occurs via the reaction ^*^O + OH^−^ → ^*^OOH, with electron transfer occurring from OH^−^ to Ni^4+^, the observed decreases in the Ni valence state, and ^*^O concentration reflect the continued formation of ^*^OOH during the OCV procedure. To validate the continued formation of ^*^OOH during the OCV on charged NiOOH, we first aimed to determine the change in the Ni valence state during the charging and OCV procedures by in situ Ni *K*-edge and O *K*-edge X-ray absorption spectroscopy (XAS). For in situ Ni *K*-edge XAS measurements, a bias of 1.503 V vs. reversible hydrogen electrode (RHE) was first applied to charge NiOOH in a 1 M KOH electrolyte, followed by an OCV test (Supplementary Fig. 6). As shown in Fig. 2a and Supplementary Fig. 7, charging NiOOH at 1.503 V vs. RHE led to a gradual blueshift in the photon energy of the absorption edge, moving from approximately 8341.8 to 8342.0 eV. This shift, observed at the half-height position in the normalized XAS spectrum, reflects a progressive increase in the average valence state of nickel from Ni^3+^ towards Ni^4+^, suggesting that NiOOH deprotonation occurs upon charging. The change in the ^*^O peak intensity associated with the deprotonation of NiOOH was subsequently analysed via in situ O *K*-edge XAS (Supplementary Fig. 8). A new peak emerged at approximately 528.9 eV under an applied potential of 1.36 V, which is attributed to the ^*^O species^24^. The emergence of the ^*^O intermediate is consistent with NiOOH deprotonation under bias. This also indicates that ^*^O is the most abundant intermediate during the OER due to ^*^OOH formation being the RDS. Furthermore, the ^*^O peak intensity gradually increased as the applied potential increased from 1.36 to 1.56 V, indicating that more ^*^O accumulated on the NiOOH surface with ^*^OH deprotonation.

The OCV procedure was then introduced after the NiOOH sample was charged. The in situ Ni *K*-edge XAS results revealed that the absorption edge of charged NiOOH gradually moved to a lower photon energy, indicating a decrease in the Ni valence state with increasing OCV duration (Fig. 2a and Supplementary Fig. 7). This decrease in valence validates the persistence of electron transfer from OH^−^ to charged Ni^4+^ sites during the OCV process. Moreover, the ^*^O peak intensity also gradually decreased with increasing OCV duration, confirming that ^*^O was consumed to form the ^*^OOH intermediate (Fig. 2b). To further validate the ^*^OOH formation during OCV, in situ attenuated total reflectance surface-enhanced infrared absorption spectroscopy (ATR-SEIRAS) was conducted. A prominent peak at 1163 cm^−1^ was detected in the ATR-SEIRAS spectra by charging NiOOH at 1.503 V vs. RHE, which can be attributed to ^*^OOH intermediate formed during OER (Supplementary Fig. 9). Upon introducing the OCV procedure, the ^*^OOH-related peak intensity progressively increased, providing compelling evidence for the continuous formation and accumulation of ^*^OOH intermediates under open-circuit conditions. Notably, this feature is absent in the initial state without applied bias or in the bare carbon cloth under applied bias (Supplementary Fig. 10), excluding a static contribution from the substrate or background electrolyte. Therefore, combining the in situ Ni *K*-edge, O *K*-edge, and ATR-SEIRAS results, we successfully reveal that ^*^OOH can form and persist on charged NiOOH by accepting electrons from OH^−^ during the OCV procedure.

### Design of the OCV‒PV protocol to quantify the ^*^OOH formation rate

**Fig. 3:**
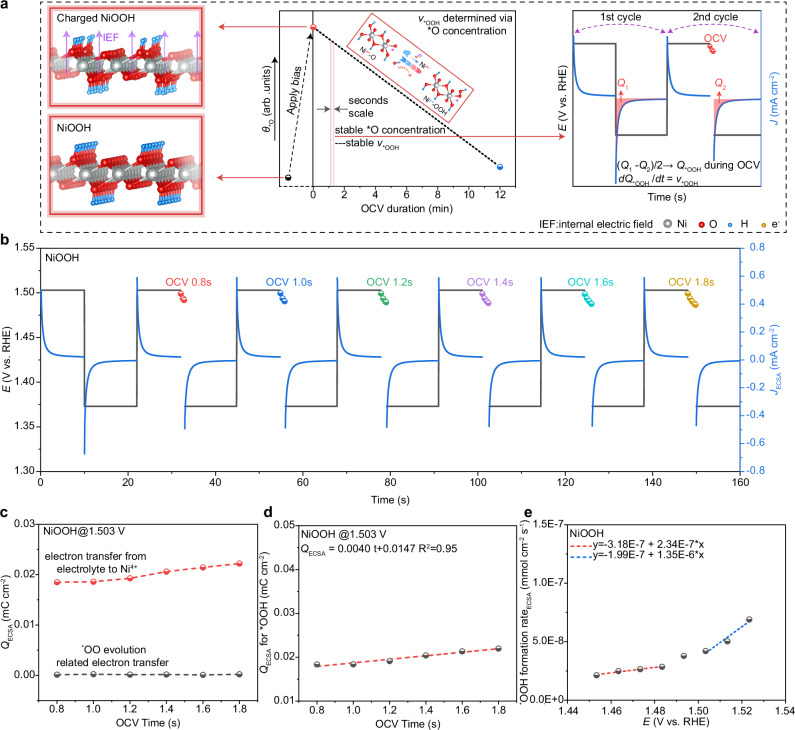
Quantification of ^*^OOH formation kinetics on NiOOH. **a** Schematic illustration of the basis of the open-circuit voltage–pulse voltammetry (OCV‒PV) measurement. Left, charged NiOOH and relaxed NiOOH structures; IEF denotes the internal electric field. Middle, schematic change in ^*^O coverage and ^*^OOH formation rate during the OCV period, where θ_*O_ denotes the surface ^*^O concentration and v_*OOH_ denotes the apparent ^*^OOH-formation rate. Right, OCV‒PV protocol. The applied potential is shown in black, the corresponding current response is shown in blue, the OCV period is indicated by the red label, and the purple dashed arrows indicate the first and second pulse voltammetry cycles. *Q*_1_ and *Q*_2_ denote the reductive readout charges measured in the first and second cycles, respectively, and Q_*OOH_ denotes the charge associated with ^*^OOH formation during OCV. **b** OCV‒PV protocol with a charging potential of 1.503 V, where the PV protocol (black) shows an oxidative and reductive pulse with a current response (blue), and the OCV procedure is shown as dots. **c** Estimated transferred charge normalized to the electrochemical surface area (ECSA) during the OCV procedure. The contribution of oxygen evolution to charge transfer was derived from in situ DEMS measurements. **d** ^*^OOH formation-related charge versus OCV duration after excluding the contribution from the ^*^OO evolution step with an applied potential of 1.503 V. **e** ^*^OOH formation rate of NiOOH under different charging potentials (1.453 to 1.523 V) via OCV‒PV after excluding the contribution from the oxygen evolution step. All electrochemical data in this work are presented without iR compensation. Source data are provided as a Source data file.

Our results showed that ^*^OOH formation could persist during the OCV after charging the electrocatalyst. However, the consumption of ^*^O during OCV also indicated that the ^*^O concentration continuously changed with ^*^OOH formation. This would lead to a dynamic change in the ^*^OOH formation rate, making quantifying its kinetics challenging. Therefore, ensuring that the ^*^O concentration remains relatively stable during the OCV is crucial for quantifying the ^*^OOH formation rate. The effect of OCV duration on the change in ^*^O concentration and corresponding change in ^*^OOH formation rate were subsequently investigated. Since ^*^OOH formation occurs via the reaction ^*^O + OH^−^ → ^*^OOH, the ^*^OOH formation rate is determined by the ^*^O concentration. This trend is also supported by DFT calculations, which indicated that the energy barrier for ^*^OOH formation decreases significantly with increasing concentrations of ^*^O (Supplementary Fig. 4)^24^. The in situ Ni *K*-edge XAS results show that it takes approximately 12 min (720 s) for Ni to return to its initial valence state (Fig. 2a). Reduction of the Ni valence state is associated with the consumption of ^*^O, which was proven by in situ O *K*-edge XAS characterization (Fig. 2b). These findings indicate that the change in ^*^O concentration during an applied OCV occurs at a relatively slow rate. If the OCV time is shortened to the second scale (1 to 2 s), the ^*^O concentration could be considered constant, leading to a steady ^*^OOH formation rate (Fig. 3a, middle). This time window ensures that kinetic analysis of ^*^OOH formation is not influenced by dynamic changes in surface ^*^O intermediate coverage. In addition, although the OCV relaxation is accompanied by a decrease in the bulk-averaged Ni valence, no obvious structural change was observed in the bulk-sensitive EXAFS spectra even after prolonged OCV relaxation during in situ Ni *K*-edge characterization (Supplementary Fig. 11), suggesting that bulk proton diffusion is unlikely to dominate the much shorter 1–2 s OCV window used for kinetic analysis. Building on these findings, an OCV‒PV measurement was designed to determine the ^*^OOH formation rate (Fig. 3a, right). During the first PV cycle, a high potential (*E*_h_) was applied to charge NiOOH. The subsequent cathodic voltage pulse was intentionally designed as a readout step to quantify the total charge associated with the reduction of oxidized Ni species (Ni^4+^ → Ni^3+^), denoted as *Q*_1_^24,25^. The OCV procedure was then introduced within a short timescale of a few seconds, and electron transfer from OH^−^ to charged NiOOH reduced the Ni valence state. During the following PV cycle, the amount of charge needed to reduce the remaining Ni^4+^ to Ni^3+^ (*Q*_2_) was quantified by integrating the current response to the cathodic voltage pulse. Generally, ^*^OOH formation proceeds via the reaction ^*^O + OH^−^ → ^*^OOH + e^−^, in which one electron is generated during the interfacial proton-coupled electron-transfer step. Under an applied bias, this electron can be transferred to the external circuit^7,8^. Under OCV conditions, however, electron transfer to the external circuit is blocked. Therefore, the electron generated from ^*^OOH formation must be redistributed locally within the catalyst and can be accommodated by nearby high-valent Ni^4+^ species, reducing them to Ni^3+^. In this sense, ^*^OOH formation under OCV is coupled to an internal redox-compensation process. Accordingly, one ^*^OOH formation event during OCV is reflected electrochemically as a two-electron reduction of Ni^4+^ species. Consequently, half of the charge difference between the two PV cycles [(*Q*_1_ − *Q*_2_)/2)] correlates to the amount of ^*^OOH formed. Thus, the ^*^OOH formation rate can be evaluated by dividing the transferred charge by the OCV duration (Fig. 3a, right). Here, the ^*^OOH formation rate measured under OCV should be understood as the initial relaxation kinetics of a pre-charged metastable surface under defined initial conditions, rather than the steady-state ^*^OOH formation rate under continuously applied bias.

Leveraging this understanding, OCV‒PV measurements were conducted at various OCV durations (1, 1.2, 1.4, 1.6, and 1.8 s) to experimentally analyse the ^*^OOH formation rate (Fig. 3b). The initial discharging current observed upon switching to the cathodic pulse corresponds to the designed cathodic readout response of the pre-charged NiOOH surface and reflects the reduction of oxidized Ni species. The amount of charge transferred from OH^−^ to Ni^4+^ under different OCV durations and *E*_h_ values normalized to the electrochemical surface area (ECSA) (*Q*_ECSA_), was then quantified (Fig. 3c and Supplementary Figs. 12–14). The number of transferred charges increases with increasing OCV duration, which agrees well with our proposed mechanism in which electron transfer from the electrolyte to the electrocatalyst persists during the OCV procedure. Moreover, the contribution of the ^*^OO evolution step was determined using in situ DEMS (details on the DEMS procedure are provided in the “Methods” section). Specifically, the calibrated DEMS signal measured within the OCV window was first converted to the amount of O_2_ detected during OCV. This O_2_ amount was then converted to the corresponding ^*^OO evolution-related charge contribution. As shown in Fig. 3c and Supplementary Fig. 15, compared with the total charge (*Q*_1_ − *Q*_2_)/2 obtained from the OCV‒PV measurement, the recorded charge associated with ^*^OO evolution is significantly lower and therefore does not dominate the extracted OCV kinetics, which agrees well with our hypothesis that ^*^O is the most abundant intermediate in the formation of ^*^OOH during the OCV. Based on this charge partition, the apparent Faradaic efficiency toward ^*^OOH formation exceeds 98% across the investigated OCV durations (Supplementary Fig. 16), indicating that the OCV-derived charge is predominantly associated with ^*^OOH formation rather than ^*^OO evolution. We note that the extracted OCV charge should therefore be understood as the apparent initial kinetics of ^*^OOH formation, which intrinsically includes the interfacial OH^−^ attack/adsorption involved in the ^*^O + OH^−^ → ^*^OOH step, whereas the contribution from the ^*^OO evolution is negligible under the present conditions. Possible adsorbate rearrangement pathways, such as 2^*^OH → H_2_O + ^*^O, are not expected to contribute to the measured OCV charge because they do not involve net electron transfer to oxidized Ni sites. In addition, possible self-discharge through oxidation of the carbon-cloth substrate was examined by Raman spectroscopy before and after the OCV–PV test, and no detectable change was observed (Supplementary Fig. 17), supporting that the measured OCV charge is not significantly contributed by carbon oxidation.

The *Q*_ECSA_ values exhibited an approximately linear relationship with the OCV duration (Fig. 3d and Supplementary Fig. 18), indicating that the slope derived from the linear fit can be used to evaluate the electron transfer rate related to ^*^OOH formation. In addition, because all measurements were conducted in 1 M KOH and within a short OCV window, significant OH⁻ depletion or O_2_ accumulation is unlikely under the present conditions, supporting that the extracted kinetics are not dominated by mass-transport limitation. Notably, the approximately linear increase of *Q*_ECSA_ with OCV duration within this short time window is inconsistent with a simple residual capacitive-discharge process and instead supports a reaction-controlled chemical relaxation of the pre-charged NiOOH surface. The charge transfer rate was 0.0040 mC cm^−2^ for NiOOH with a charging bias of 1.503 V, corresponding to a ^*^OOH formation rate of 4.20E-8 mmol cm^−2^ s^−1^. Compared to the current density, the quantified ^*^OOH formation rate was within a reasonable range (a detailed discussion is provided in Supplementary Fig. 19). The ^*^OOH formation rate under each applied potential was subsequently quantified, with the fitted slope representing the rate of change in ^*^OOH formation with respect to the applied bias (Fig. 3e). The ^*^OOH formation rate for NiOOH increases from 2.13E-8 mmol cm^−2^ s^−1^ at 1.453 V to 6.90E-8 mmol cm^−2^ s^−1^ at 1.523 V. A larger slope indicates a faster increase in the ^*^OOH formation rate, suggesting more favourable ^*^OOH kinetics for the electrocatalyst. We further find that the ^*^OOH formation rate has a bilinear response to the charging potential, increasing from 2.34E-7 mmol cm^−2^ s^−1^ V^−1^ in the low potential region to a significantly higher value of 1.35E-6 mmol cm^−2^ s^−1^ V^−1^ in the high potential region (Fig. 3e). The increase in the ^*^OOH formation rate in the high potential region is responsible for the bend observed in the Tafel slope, which reflects a change in the reaction kinetics^24^ (Supplementary Fig. 20). We note that the present OCV–PV analysis is interpreted within a Ni-centered AEM framework, consistent with the current understanding of NiOOH-based OER^8^, and is not designed to independently resolve contributions from oxygen-redox pathways, which would require dedicated operando probes for direct identification.

To preliminarily examine the generality of the OCV-driven relaxation framework, we further performed OCV–PV measurements on CoOOH under an identical charging bias. A similar qualitative OCV-induced charge evolution was observed (Supplementary Fig. 21), suggesting that the present framework is not unique to NiOOH and may be applicable to other transition-metal oxyhydroxides capable of forming high-valent surface states under OER conditions.

### Decoupling electron transfer to guide catalyst design

**Fig. 4:**
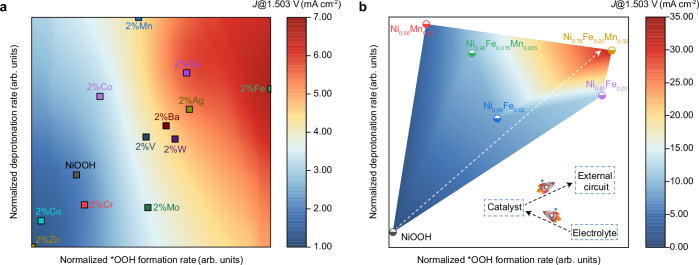
Decoupling electron transfer to guide catalyst design. **a** Influence of various cation dopants (2 at%) on tuning ^*^OOH formation rate, ^*^OH deprotonation ability, and corresponding OER activity for Ni-based oxyhydroxide materials. **b** Experimental demonstration of the proposed design strategy using the (Fe, Mn) co-doped NiOOH system, where concurrent facilitation of electron transfer from the catalyst to the external circuit and from the electrolyte to the catalyst optimizes the overall OER kinetics. All electrochemical data in this work are presented without iR compensation. Source data are provided as a Source data file.

Doping represents one of the simplest yet most effective strategies for modulating the electronic structure and catalytic performance of NiOOH. However, due to the inherent difficulty in decoupling electron transfer processes, the influence of dopant elements on the OER electron transfer kinetics remains poorly understood. Consequently, this knowledge gap hinders the rational design of catalysts to efficiently optimize OER performance. Here, we demonstrate that the OCV–PV measurements provide an effective approach to quantitatively determine the ^*^OOH formation rate, while the previously proposed PV method can be employed to evaluate the ^*^OH deprotonation kinetics^24,25^. As such, these complementary techniques enable decoupled evaluation of key electron transfer steps, offering a mechanistic basis for correlating intrinsic kinetics with overall OER activity. To achieve this, a series of NiOOH-based materials were synthesized by incorporating a range of cation dopants (each at a concentration of 2 at%), including V, Cr, Mn, Fe, Co, Cu, Zn, and others. XRD patterns of all 2 at% doped samples show diffraction features consistent with the parent Ni(OH)_2_ phase, without detectable impurity peaks or phase segregation (Supplementary Fig. 22). We note that different dopants may also influence conductivity, defect density, and surface reconstruction behavior. Therefore, the present OCV–PV/PV framework is not intended to assume identical microscopic properties across all doped catalysts. Rather, it is used to compare the apparent step-specific kinetics under identical testing conditions. The ^*^OOH formation kinetics of these materials were evaluated at a charging potential of 1.503 V using the OCV‒PV method. A fixed potential of 1.503 V was chosen here as a representative comparison point because it lies in the OER-relevant region where all of the doped NiOOH catalysts are electrochemically active, while still providing a sufficiently oxidized surface state for resolving dopant-dependent differences in step-specific kinetics under identical conditions. Meanwhile, PV measurements were conducted to evaluate their ^*^OH deprotonation abilities. The PV protocol was shown in Supplementary Fig. 23, with NiOOH as the model sample. The OCV‒PV and PV measurements results for these NiOOH-based materials are provided in Supplementary Figs. 24–35. To elucidate the interdependence between the ^*^OOH formation rate, ^*^OH deprotonation rate, and overall OER activity, a contour map was constructed by plotting the normalized ^*^OOH formation rate (x-axis) and normalized ^*^OH deprotonation rate (y-axis) against the corresponding OER current density at 1.503 V vs. RHE, using NiOOH as the benchmark sample (Fig. 4a). It is revealed that different cation dopants exhibit varying capabilities in modulating either the ^*^OOH formation rate or the ^*^OH deprotonation rate, which fundamentally accounts for the distinct extents of OER activity enhancement observed across the doped samples. These results suggest that different dopants selectively perturb different parts of the Ni–O reaction coordinate within the NiOOH lattice, thereby facilitating different elementary OER steps to different extents. Remarkably, Fe doping exhibits the strongest capability in accelerating ^*^OOH formation, resulting in the highest OER activity. In contrast, Mn and Ce doping more effectively enhance the ^*^OH deprotonation kinetics, suggesting distinct capabilities for modulating OER activity. We emphasize that these dopant-dependent differences are identified at the level of experimentally measured apparent step-specific kinetics. The present results do not distinguish whether such effects originate predominantly from catalyst electronic-structure modulation, from changes in the interfacial solvation/electrical-double-layer environment, or from their combined contributions. Direct verification of dopant-induced restructuring of interfacial water would require dedicated operando interfacial spectroscopy and is beyond the scope of the present work.

As previously discussed, the OER pathway is governed by two electron transfer processes: from the electrolyte to the catalyst and from the catalyst to the external circuit. Therefore, if both electron transfer pathways can be kinetically optimized simultaneously, it is possible to achieve a substantial enhancement in overall catalytic performance. To achieve this, a Fe, Mn co-doped NiOOH system was designed as a model catalyst (Fig. 4b). By incorporating 1.5% Fe and 0.5% Mn into NiOOH, it was revealed that the two dopants could synergistically optimize the bidirectional electron transfer kinetics between the electrolyte and the external circuit, resulting in significantly enhanced OER activity compared to Ni_0.98_Fe_0.02_OOH and Ni_0.98_Mn_0.02_OOH (Fig. 4b and Supplementary Figs. 26, 27, 36). Here, the roles of Fe and Mn are discussed at the level of experimentally resolved apparent step-specific kinetics, rather than at the level of atomically identified active-site motifs. The present results, therefore, do not directly resolve the local Fe–Mn dopant configuration or the exact sites for ^*^O and ^*^OOH formation, which would require dedicated operando structural characterization. Building upon this, the Fe concentration was further increased to 20%, which has been widely recognized as the optimal composition for achieving high OER activity in Fe-doped NiOOH systems^25^. The ^*^OOH formation rate was substantially increased by Fe doping at this level (Supplementary Fig. 37). Our previous work reveals that the increase in Fe content can lead to stronger NiO_6_ distortion, which is associated with enhanced *e*_g_^*^ band broadening. Such electronic-structure modulation is expected to increase the density of states near the Fermi level and thereby facilitate electron transfer between the electrolyte and the electrocatalyst. To further support this interpretation, we performed DFT calculations on Ni_1-x_Fe_x_OOH (*x* = 0, 0.02, and 0.2) to evaluate the energy barrier for ^*^OOH formation. The calculated barrier decreases with increasing Fe content, following the order NiOOH > Ni_0.98_Fe_0.02_OOH > Ni_0.8_Fe_0.2_OOH (Supplementary Fig. 38). This trend is consistent with the OCV–PV results and suggests that higher Fe incorporation may facilitate ^*^OOH formation in NiFeOOH. Subsequently, an additional 2% Mn was introduced to further accelerate the ^*^OH deprotonation process (Supplementary Fig. 39). Consequently, both ^*^OOH formation and ^*^OH deprotonation kinetics were effectively enhanced in Ni_0.78_Fe_0.20_Mn_0.02_OOH, leading to enhanced OER activity of 30.99 mA cm^−2^ at 1.503 V, which is more than twice that of Ni_0.80_Fe_0.20_OOH (12.22 mA cm^−2^) (Fig. 4b and Supplementary Fig. 40a). The Ni_0.78_Fe_0.20_Mn_0.02_OOH catalyst maintains stable chronopotentiometric performance at 10 mA cm^−2^ over 100 h without element segregation or dissolution, supporting that the kinetically guided catalyst design also delivers robust OER stability (Supplementary Figs. 40b, 41, 42, and Table 1). In principle, further increasing Mn content may provide additional benefit; however, such improvement is expected to be limited once ^*^OOH formation remains the dominant RDS in the present framework, whereas ^*^OH deprotonation serves as a secondary kinetic process. As such, by decoupling the electron transfer processes, this study provides experimental insight into how dopant elements influence the OER electron transfer kinetics and corresponding OER activity. This quantitative metric offers a valuable tool for catalyst screening and can be leveraged to identify promising candidates with optimized electronic properties.

## Discussion

In conclusion, by decoupling electron transfer pathways through the OCV–PV strategy, we quantitatively determine the ^*^OOH formation rate and establish it as a kinetic descriptor that governs oxygen evolution catalysis. When integrated with PV analysis of ^*^OH deprotonation kinetics, this approach constructs a step-specific kinetic framework that clarifies how different dopant elements regulate distinct electron-transfer steps. Guided by this framework, Fe and Mn co-doping is demonstrated to synergistically accelerate both ^*^OOH formation and ^*^OH deprotonation, resulting in enhanced OER performance. This work transforms the understanding of OER kinetics from phenomenological observation to quantitative descriptor-driven design and paves the way for extending electron transfer decoupling strategies to define kinetic descriptors in other multistep electrocatalytic reactions.

## Methods

### Materials

Nickel nitrate hexahydrate (Ni(NO_3_)_2_·6H_2_O, 99.999% trace metals basis), iron nitrate nonahydrate (Fe(NO_3_)_3_·9H_2_O, ≥98%), chromium nitrate nonahydrate (Cr(NO_3_)_3_·9H_2_O, ≥99.99% trace metals basis), cobalt nitrate hexahydrate (Co(NO_3_)_2_·6H_2_O, ≥98%), manganese nitrate tetrahydrate (Mn(NO_3_)_2_·4H_2_O, ≥99.9% trace metals basis), silver nitrate (AgNO_3_, ≥99%), cerium nitrate hexahydrate (Ce(NO_3_)_3_·6H_2_O, ≥98%), copper nitrate trihydrate (Cu(NO_3_)_2_·3H_2_O, 99–104%), zinc nitrate hexahydrate (Zn(NO_3_)_2_·6H_2_O, 98%), barium nitrate (Ba(NO_3_)_2_, ≥99%), vanadium chloride (VCl_3_, 97%), tungsten chloride (WCl_6_, ≥99.9% trace metals basis), molybdenum chloride (MoCl_5_, 99.9% trace metals basis), urea (99.0–100.5%), are purchased from the Sigma-Aldrich. These chemicals were reagent grade and used as received without further purification.

### Synthesis of Ni(OH)_2_ based electrocatalysts

A facile hydrothermal method is conducted to synthesize Ni(OH)_2_ based samples on carbon cloth^26^. First, 2 mmol total amount of Ni(NO_3_)_2_·6H_2_O, 10 mmol urea were added into 35 mL deionized (DI) water and stirred for 15 min to form uniform solution. One piece of carbon cloth with a size of 2 cm × 3 cm was immersed into the solution. Then, they were transferred into a 50 mL Teflon-lined stainless-steel autoclave and kept in oven at 120 °C for 10 h. The obtained Ni(OH)_2_ was then washed by DI water and ethanol for at least three times, and further dried under 70 °C in air for 4 h. The catalyst loading was estimated from the mass difference of the carbon cloth before and after Ni(OH)_2_ growth and drying, and normalized by the geometric electrode area. The representative catalyst loading of Ni(OH)_2_ was ~2.3 mg cm^−2^. For 2% X doped Ni(OH)_2_ (X = V, Cr, Mn, Fe, Co, Cu, Zn, Ba, W, Mo, Ag, Ce), the total amount of (Ni + X) is kept at 2 mmol.

### Preparation of Fe-purified KOH electrolyte

The alkaline electrolyte was purified to minimize trace Fe contamination following a reported Ni(OH)_2_ scavenging protocol^26^. A 1 M KOH solution was first prepared by diluting commercial KOH (45 wt% in H_2_O) with deionized water. Ni(OH)_2_ was generated in situ by adding 0.5 g Ni(NO_3_)_2_·6H_2_O to 30 mL of 1 M KOH, and the resulting suspension was centrifuged at 7826 × *g* for 10 min. The isolated Ni(OH)_2_ solid was transferred into 50 mL of 1 M KOH and stirred for 10 min, allowing trace Fe species to be captured by the Ni(OH)_2_ phase. After 24 h of equilibration, the mixture was centrifuged at 7826 × *g* for 10 min, and the clear supernatant was used for electrochemical measurements. The pH value of the purified 1 M KOH is measured to be 13.65 ± 0.06^26^.

### Material characterizations

The in situ nickel *K*-edge X-ray absorption spectra were collected in transmission mode at the XAFCA beamline of the Singapore Synchrotron Light Source. During the measurements, the storage ring operated at 0.7 GeV with a beam current of approximately 200 mA^27^. A Ni foil was measured simultaneously as the energy reference for calibration. Extended X-ray absorption fine-structure data were processed by applying a Hanning window to the k^2^-weighted spectra over a *k*-range of 2.5–10.5 Å^−1^, followed by Fourier transformation. This three-electrode setup employed sample based on carbon cloth as the working electrode, carbon rod as the counter electrode, and Ag/AgCl as the reference electrode in 1 M KOH electrolyte. The in situ oxygen *K*-edge XAS spectra were obtained by recording fluorescence yield and selected by silicon drift detector (FAST SDD@ Ultra High Performance Silicon Drift Detector, Ametek) in 0.1 M KOH media, using in an ionization chamber at tender X-ray beamline BL16A1 at the National Synchrotron Radiation Research Center (NSRRC), which is equipped with double crystal (Si (1110)) monochromator (DCM).

### Electrochemical measurements

All the electrochemical measurements were performed using an electrochemical workstation (VPM3, BiO-logic Inc) in a three-electrode setup in Fe-purified 1 M KOH. Current, potential, and time responses were recorded directly using the workstation software and exported for post-analysis. The Hg/HgO electrode was chosen as the reference electrode. The Hg/HgO electrode was calibrated to the reversible hydrogen electrode (RHE) scale according to ${E}_{{\rm{RHE}}}={E}_{{\rm{Hg}}/{\rm{HgO}}}+0.098\,{\rm{V}}+0.059\times {\rm{pH}}$ at 23 °C. The Pt plate was used as the counter electrode. The electrocatalyst grown on carbon cloth was used as the working electrode. The linear scan voltammetry (LSV) was measured at a scan rate of 0.1 mV s^−1^. Specifically, a negative scan was carried out for the LSV measurement to avoid the influence of Ni^2+/3+^ redox current on the OER activity. All electrochemical data in this work are presented without iR compensation. The geometric dimensions of the electrodes for LSV are 1 cm^2^. The electrochemical surface area (ECSA) of electrocatalyst was evaluated by recording the electrochemical double-layer capacitance through cyclic voltammograms (CVs) measurement. Specifically, the potential range for CVs was set at 0.02–0.12 V (versus Hg/HgO) to avoid the Faradaic process. The CVs were conducted in the quiescent electrolyte with 5 scan rates, including 10, 20, 30, 40, and 50 mV s^−1^. The charging current *I *(mA) was plotted versus the scan rate (V s^−1^). Then, a straight line could be fitted where the slope represented the double-layer capacitance (*C*_dl_). The ECSA was derived by dividing *C*_dl_ by specific capacitance *C*_s_ = 0.04 mF cm^−2^. Unless otherwise stated, the electrochemical measurements in this work were performed once. Therefore, no standard deviations or error bars are provided for the corresponding measured or derived values.

### OCV–PV measurement

Before the OCV–PV measurement, the Ni(OH)_2_ grown on carbon cloth was fully oxidized to NiOOH species at 10 mA cm^−2^ for 1 h. Then, the potential was kept at a low potential at 1.373 V for 10 min to make sure that all the accumulated oxidative charge in NiOOH during the previous oxidation process had been transferred to external circuit. When beginning the first OCV–PV test, a higher potential (*E*_h_ = 1.453 V) was applied on the NiOOH for 10 s, then switched and kept at a lower potential (*E*_l_ = 1.373 V) for 12 s. Then, this cycle was repeated except that an OCV protocol was inserted before switching to *E*_l_. The OCV time was gradually increased from 0.8 to 1.8 s in 0.2 s/cycle. The test was repeated while increasing *E*_h_ from 1.453 to 1.523 V in 10 mV/interval and keep *E*_l_ unchanged. With this OCV–PV measurement, the number of transferred electron normalized to ECSA (*Q*_ECSA_) during various OCV time at each applied *E*_h_ could be evaluated. Since the electron transfer contributed by O_2_ evolution was negligible compared to the *Q*_ECSA_, we directly use the *Q*_ECSA_ to evaluate the contribution from ^*^OOH formation. Specifically, the fitted linear slope obtained from *Q*_ECSA_-OCV time was used to evaluate the electron transfer rate related to ^*^OOH formation (mC cm^−2^ s^−1^), which was then converted to the ^*^OOH formation rate (mmol cm^−2^ s^−1^). The electrolyte was continuously stirred during the measurements.

### DEMS measurement

Differential electrochemical mass spectrometry (DEMS) was used to quantify the evolved oxygen molecules within the OCV window at different OCV durations during the OCV–PV measurement. A schematic of DEMS electrochemical cell is provided in Supplementary Fig. 43. L-DP01 membrane from Shanghai Linglu is employed. The Ag/AgCl electrode was chosen as the reference electrode. The Pt wire was used as the counter electrode. The electrolyte is 1 M KOH. Specifically, after electrochemical activation, the catalyst was held at 1.503 V under steady OER conditions, and the electrochemical charge passed during the steady-state interval was converted to the corresponding O_2_ amount using Faraday’s law *n*(O_2_) = *Q*_cell_/4 *F* (Supplementary Fig. 15). The simultaneously recorded, baseline-corrected DEMS *m/z* = 32 signal was integrated over the same time window to obtain the detector response, from which an effective molar sensitivity was determined as *k* = 3.7 mol/C. This calibration factor was then applied to the OCV-period DEMS signal to estimate the amount of O_2_ detected within the OCV window. Within the present mechanistic framework, this O_2_ amount was converted to the corresponding ^*^OO evolution-related charge contribution according to: *Q*(^*^OO) = *F n*(O_2_).

### Computational method

Density functional theory calculations were carried out using the Vienna ab initio simulation package (VASP) code. The Perdew–Burke–Ernzerhof functional was used to describe exchange–correlation interactions, and the projector augmented-wave method was adopted for the ion-electron interactions^28–30^. The computational settings followed previously reported NiOOH-based oxygen-evolution calculations with appropriate modifications for the present models^26^. For the projector augmented-wave potentials, the valence electron configurations were for 3*d*^8^4*s*^2^ Ni, 2*s*^2^2*p*^4^ for O, and 1*s*^1^ for H. Spin-polarized calculations with a Hubbard correction were used for the Ni 3d states, with *U*_eff_ = 5.3 eV. The plane-wave cutoff energy was tested from 300 to 600 eV, and 500 eV was selected because the total energy was converged within 10 meV. The CONTCAR of the optimized models is provided in Supplementary Data 1.

The four steps in the AEM route are as follows:

1 $${}^{\ast }+{{\rm{OH}}}^{-}\to {}^{\ast }{\rm{O}}{\rm{H}}+{{\rm{e}}}^{-}$$

2 $${}^{\ast }{\rm{O}}{{\rm{H}}+{\rm{OH}}}^{-}\to {}^{\ast }{\rm{O}}{+{\rm{H}}}_{2}{{\rm{O}}+{\rm{e}}}^{-}$$

3 $${}^{\ast }{\rm{O}}{+{\rm{OH}}}^{-}\to {}^{\ast }{\rm{O}}{{\rm{OH}}+{\rm{e}}}^{-}$$

4 $${}^{\ast }{\rm{O}}{{\rm{OH}}+{\rm{OH}}}^{-}\to {}^{\ast }{\rm{O}}{\rm{O}}({\rm{O}}_{2}{\rm{evolution}})+{\rm{H}}_{2}{{\rm{O}}+{\rm{e}}}^{-}$$

## Supplementary information

Supplementary Information

Description of Additional Supplementary Files

Supplementary Data 1

Transparent Peer Review File

## Source data

Source data

## Data Availability

All the data generated in this study are provided in the Supplementary Information/Source Data file. Source data are provided with this paper.
