# Supplementary material for: Decoupling electron transfer defines a quantitative kinetic framework for oxygen evolution catalysis
Source: Nat Commun. 2026 Jun 10;17:7377. doi: 10.1038/s41467-026-74392-3 (PMC13402610; doi:10.1038/s41467-026-74392-3)
Supplement: Supplementary file 1 — Supplementary Information [file 41467_2026_74392_MOESM1_ESM.pdf]

Supplementary information

Decoupling electron transfer defines a quantitative kinetic framework for oxygen evolution catalysis

Authors

Haoyin Zhong(钟豪胤)[1], Junchen Yu(禹俊辰)[1], Qi Zhang(张起)[1], Xin Zhang(张歆)[1], Shanlin Li(李善霖)[2], Qingli Xu(许庆丽)[3], Zhi-Gen Yu(余志根)[4], Cheng-Hao Chuang(莊程豪)[5], Shibo Xi(席识博)[1,6*], Xiaopeng Wang(王晓鹏)[7*], Junmin Xue(薛军民)[1*]

Affiliations

[1] Department of Materials Science and Engineering, National University of Singapore, Singapore, 117575, Singapore.

[2] Institute of Materials Research, Tsinghua Shenzhen International Graduate School, Tsinghua University, Shenzhen 518055, China.

[3] National Engineering Laboratory for Modern Silk, College of Textile and Clothing Engineering, Soochow University, Suzhou 215127, China

[4] Institute of High Performance Computing, Agency for Science, Technology and Research, Singapore, 138632, Singapore.

[5] Department of Physics, Tamkang University, New Taipei City 112074, Taiwan.

[6] Institute of Sustainability for Chemical, Energy and Environment (ISCE^2), Agency for Science, Technology and Research, Singapore, 627833, Singapore.

[7] College of Materials Science and Engineering, State Key Laboratory of Intelligent Construction and Healthy Operation and Maintenance of Deep Underground Engineering, Sichuan University, Chengdu 610065, China.

[*]Correspondence to: msexuejm@nus.edu.sg (Junmin Xue); wangxiaopeng0620@163.com (Xiaopeng Wang); xi_shibo@isce2.a-star.edu.sg (Shibo Xi)

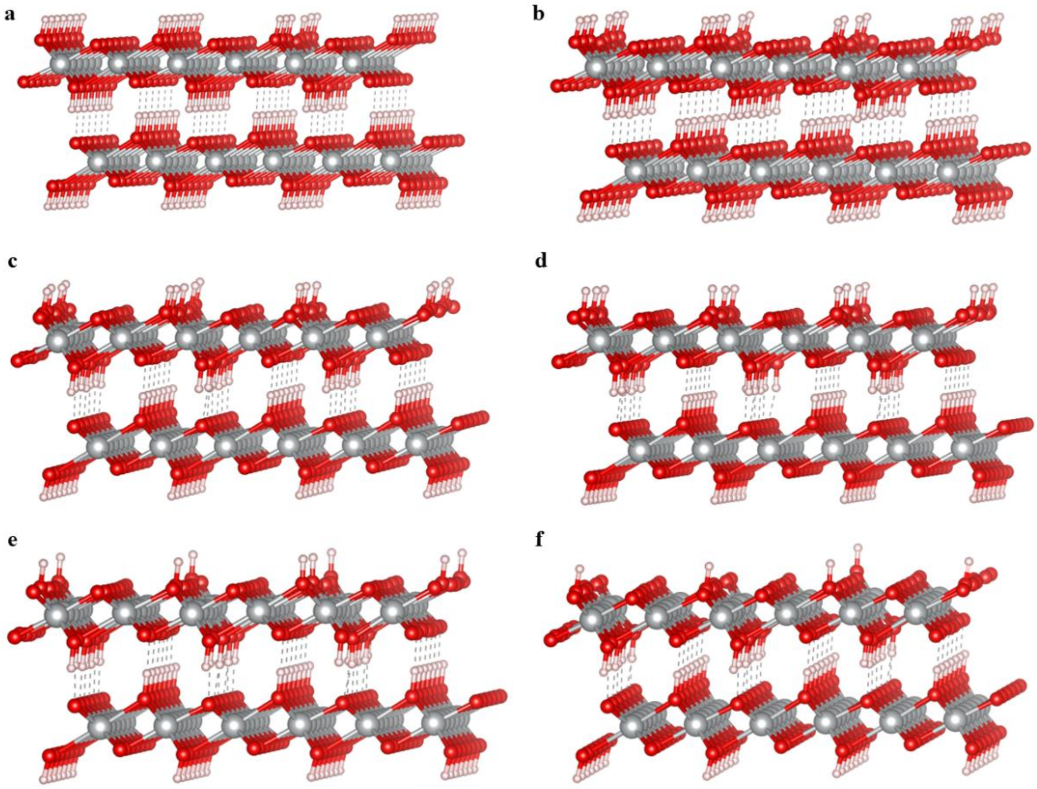

Supplementary Fig. 1. The models of NiOOH with different surface *O concentration. The number of H on the NiOOH monolayer surface (ML) is set as (a) 17 H, (b) 14 H, (c) 11 H, (d) 9 H, (e) 6 H, and (f) 3 H, respectively.

There were 18 H on the pristine NiOOH monolayer surface (ML). We changed the *O concentration on the NiOOH ML by varying the deprotonation extent. The *O concentration was 0.50 for pristine NiOOH with 18 H, 0.53 for NiOOH with 17 H, 0.61 for NiOOH with 14 H, 0.69 for NiOOH with 11 H, 0.75 for NiOOH with 9 H, 0.83 for NiOOH with 6 H, and 0.92 for NiOOH with 3 H, respectively.

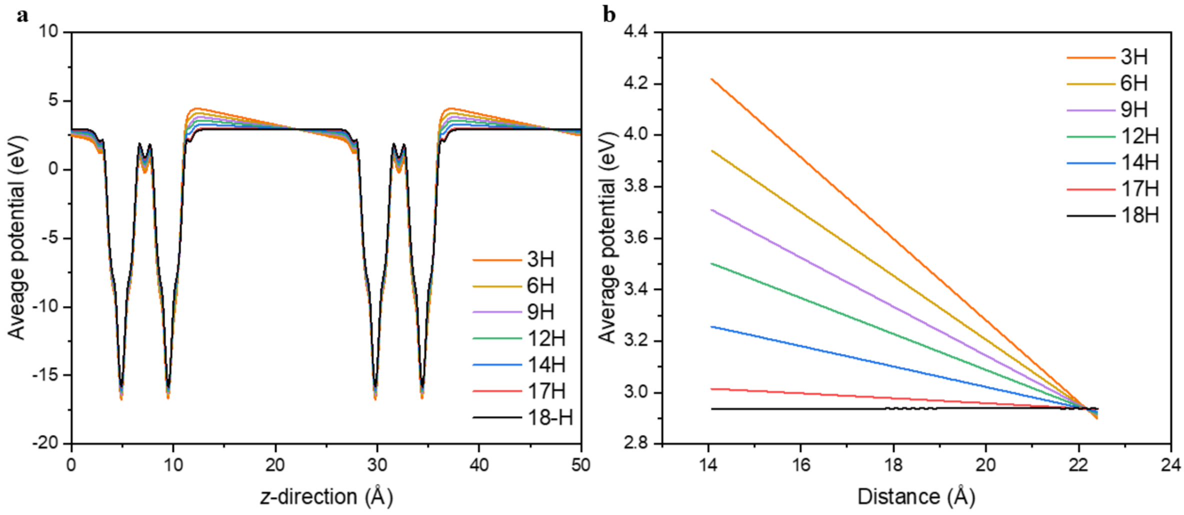

Supplementary Fig. 2. LOCPOT calculation for NiOOH with different number of H on monolayer surface (a) Average potential change along *z*-dirention. (b) enlarged data from (a). Source data are provided as a Source Data file.

It can be observed that there was no potential change along *z*-dirention for NiOOH with 18 H. However, with the *O concentration increased (less number of H) on NiOOH ML, a stronger potential change could be observed, indicating a stronger internal electric field.

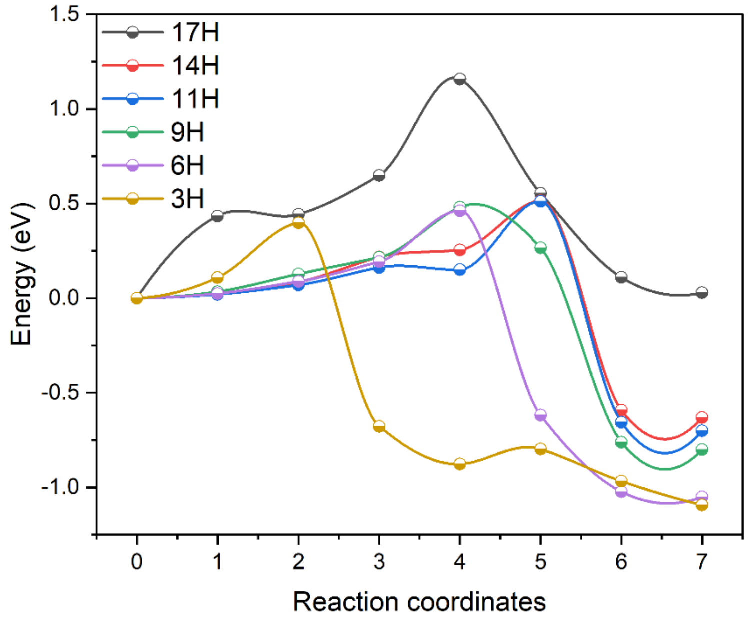

Supplementary Fig. 3. DFT calculation of $^{*}OOH$ formation for NiOOH with varying extent of $^{*}O$ concentration. Source data are provided as a Source Data file.

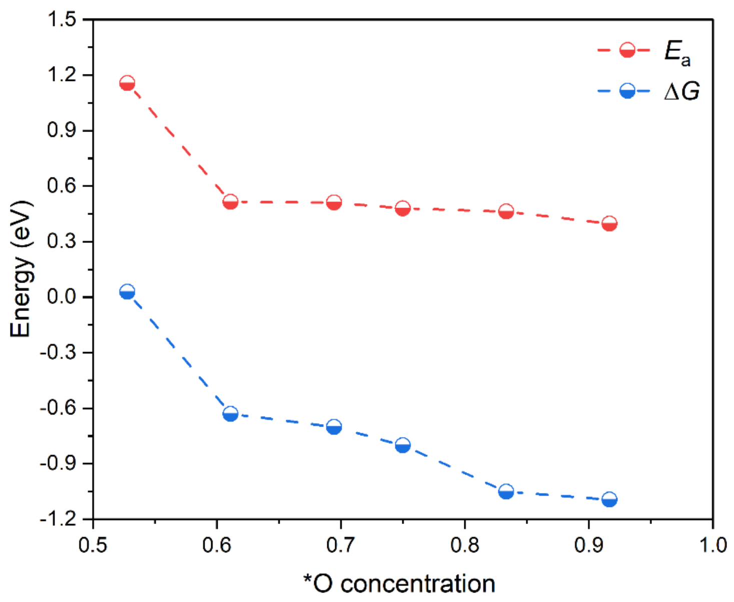

Supplementary Fig. 4. The effect of *O concentration change on the ΔG and E_a for H_2O dissociation to form *O-OH and *O-H. Source data are provided as a Source Data file.

The ΔG and E_a were obtained from Supplementary Figure 3. It was revealed that with higher *O concentration, both ΔG and E_a became lower, indicating the *OOH formation was more energetically favorable.

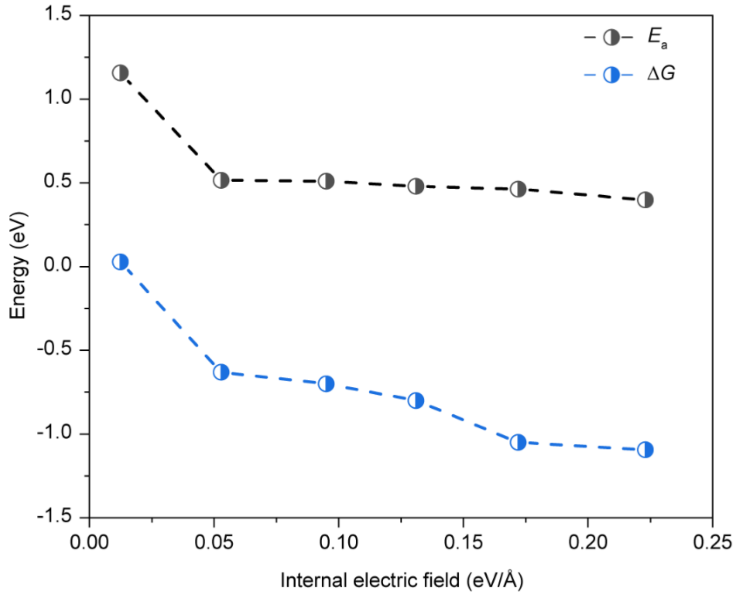

Supplementary Fig. 5. The relation between internal electric field and *OOH formation energy (E_a and $\triangle G$). Source data are provided as a Source Data file.

As the internal electric field strengthens, both the E_a and $\triangle G$ for H_2O dissociation to form *O-OH and *O-H became more energetically favorable.

a b

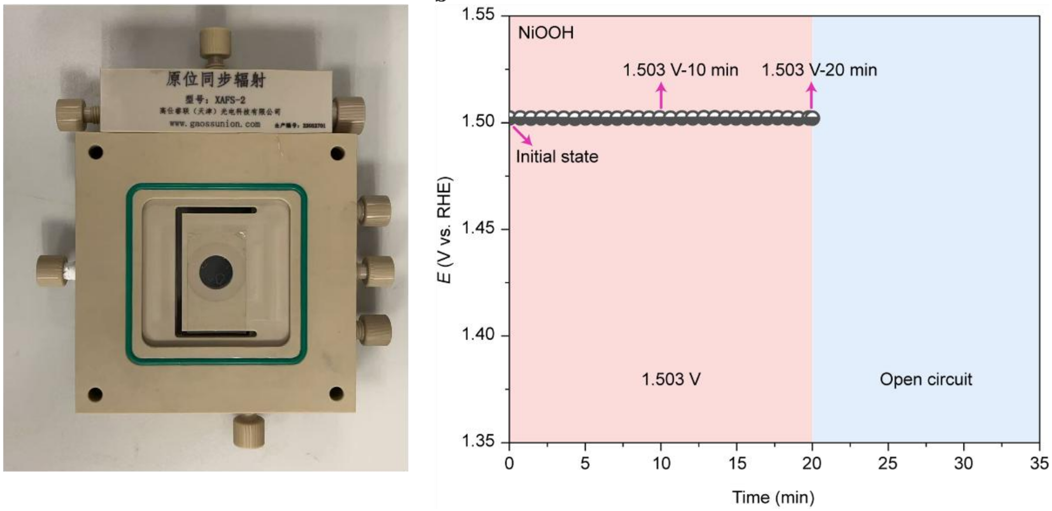

Supplementary Fig. 6. Experimental set-up for the in situ Ni *K*-edge XAS measurement. (a) Photograph of the custom-built electrochemical cell used for the in situ synchrotron XAS measurement. This three-electrode setup employed sample based on carbon cloth as the working electrode, carbon rod as the counter electrode, and Ag/AgCl as the reference electrode in 1 M KOH electrolyte. (b) Electrochemical measurement protocol used for *in situ* Ni *K*-edge XAS, showing the initial oxidation process at 1.503 V (black) followed by the OCV procedure.

First, the NiOOH was subjected to a charge potential at 1.503 V vs. RHE for 20min. Then OCV was conducted after charging the NiOOH.

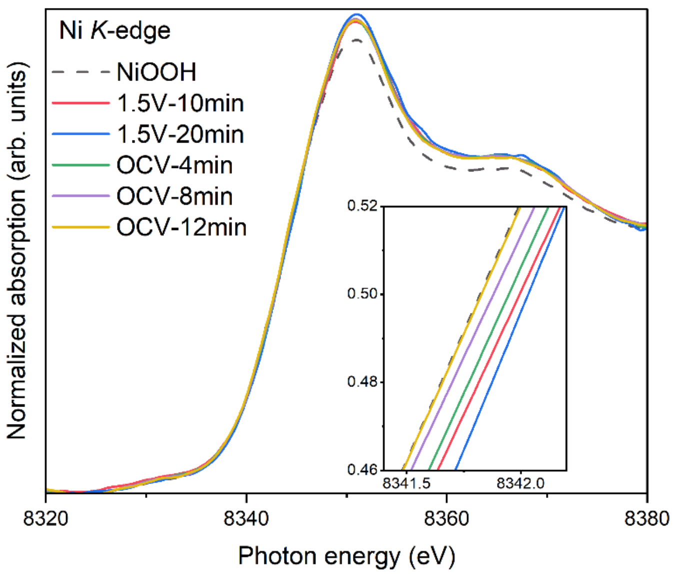

Supplementary Fig. 7. *In-situ* Ni *K*-edge XAS characterization of NiOOH. Source data are provided as a Source Data file.

The *in-situ* Ni *K*-edge XAS characterization was conducted at the same time with the electrochemical test shown in Supplementary Figure 6. The half-height position (E_{edge}) for NiOOH was obtained to determine the valence state change. A higher energy position of E_{edge} would indicate a higher valence state of Ni in NiOOH.

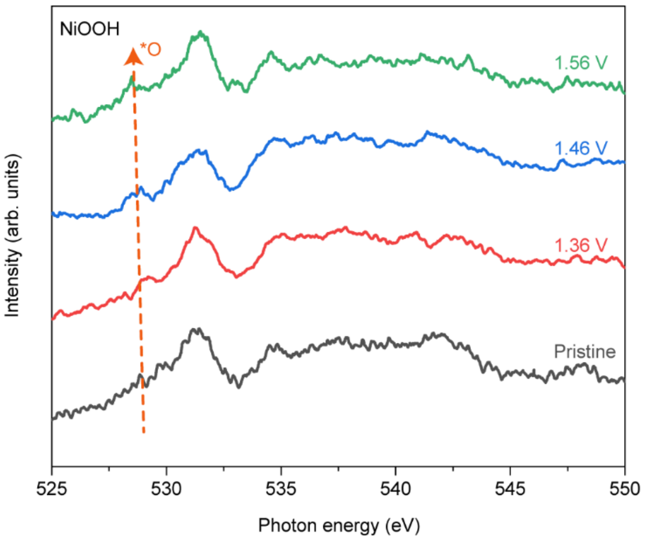

Supplementary Fig. 8. *In situ* O *K*-edge XAS characterization of NiOOH under applied potentials of 1.36 V, 1.46 V, and 1.56 V in 0.1 M KOH. Source data are provided as a Source Data file.

It was observed that a new peak with higher intensity emerged at approximately 528.9 eV under a higher charging potential, which was identified as the *O intermediate. The accumulation of *O intermediate would indicate the *OH deprotonation on NiOOH under charging bias, which was consistent with the *in situ* XAS test.

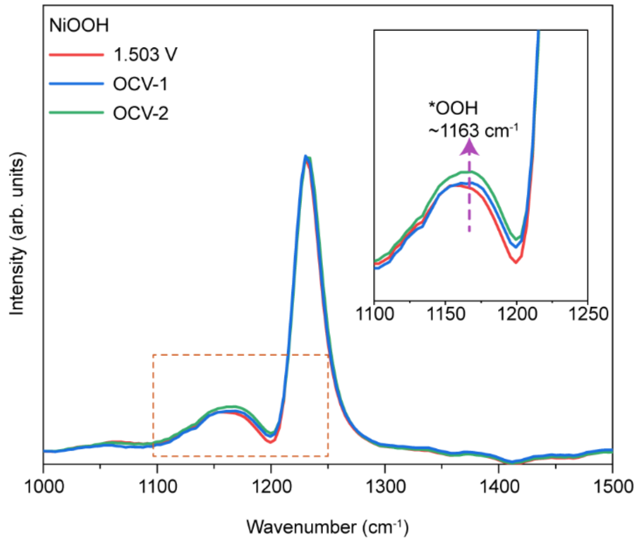

Supplementary Fig. 9. *In situ* ATR-SEIRAS characterization on NiOOH under applied potentials of 1.403 V, 1.503 V, and following OCV procedure. Source data are provided as a Source Data file.

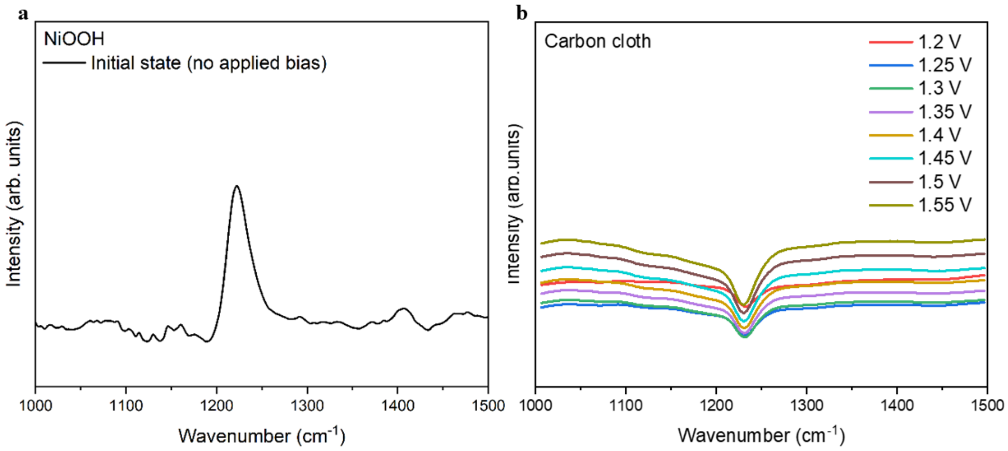

Supplementary Fig. 10. ATR-SEIRAS characterization. **a** NiOOH initial state without applied bias. **b** Bare carbon cloth. Source data are provided as a Source Data file.

The initial state (without applied bias) does not exhibit a discernible band at 1160 cm^{-1}. Meanwhile, no intermediate evolution is observed for bare carbon cloth under applied bias. These observations exclude a static C–O vibration of the carbon support or a background electrolyte absorption as the origin of this band.

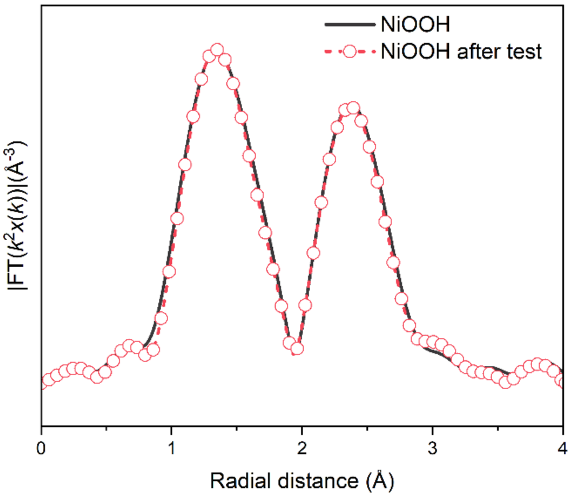

Supplementary Fig. 11. EXAFS spectra of NiOOH before and after *in situ* Ni *K*-edge XAS characterization. Source data are provided as a Source Data file.

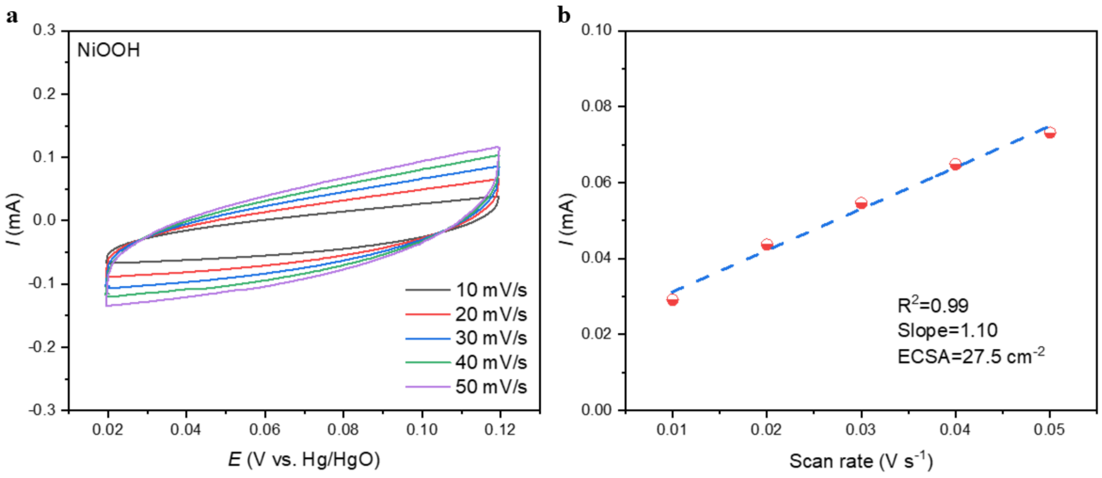

Supplementary Fig. 12. ECSA characterization of NiOOH. (a) The Cyclic voltammograms curves of the NiOOH and corresponding current (*I*) vs. scan rate plots (b). Plots of current vs. scan rate data were obtained from CV curves at 0.07 V vs. Hg/HgO. Source data are provided as a Source Data file.

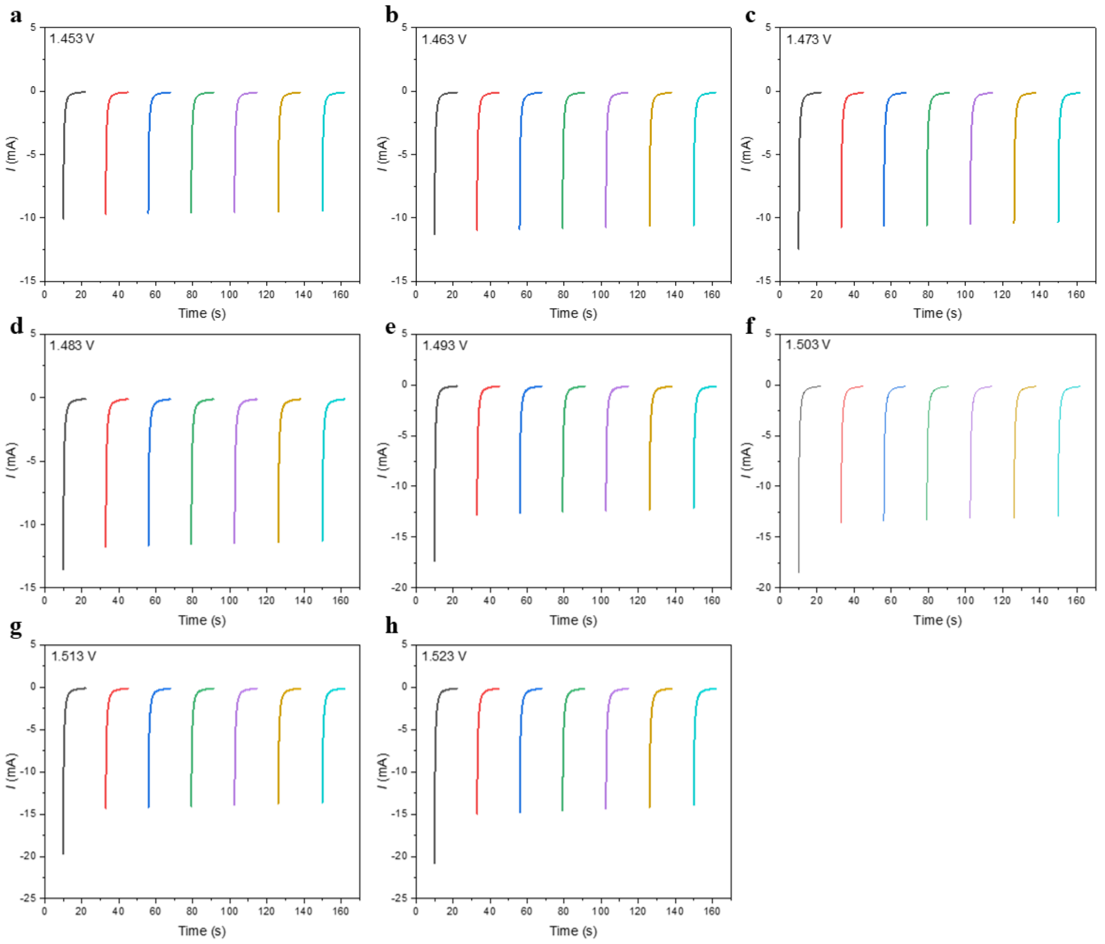

Supplementary Fig. 13. The reductive pulse with the current response in OCV-PV test for NiOOH under each applied potential. (a) 1.453 V (b) 1.463 V (c) 1.473 V (d) 1.483 V (e) 1.493 V (f) 1.503 V (g) 1.513 V (h) 1.523 V. Source data are provided as a Source Data file.

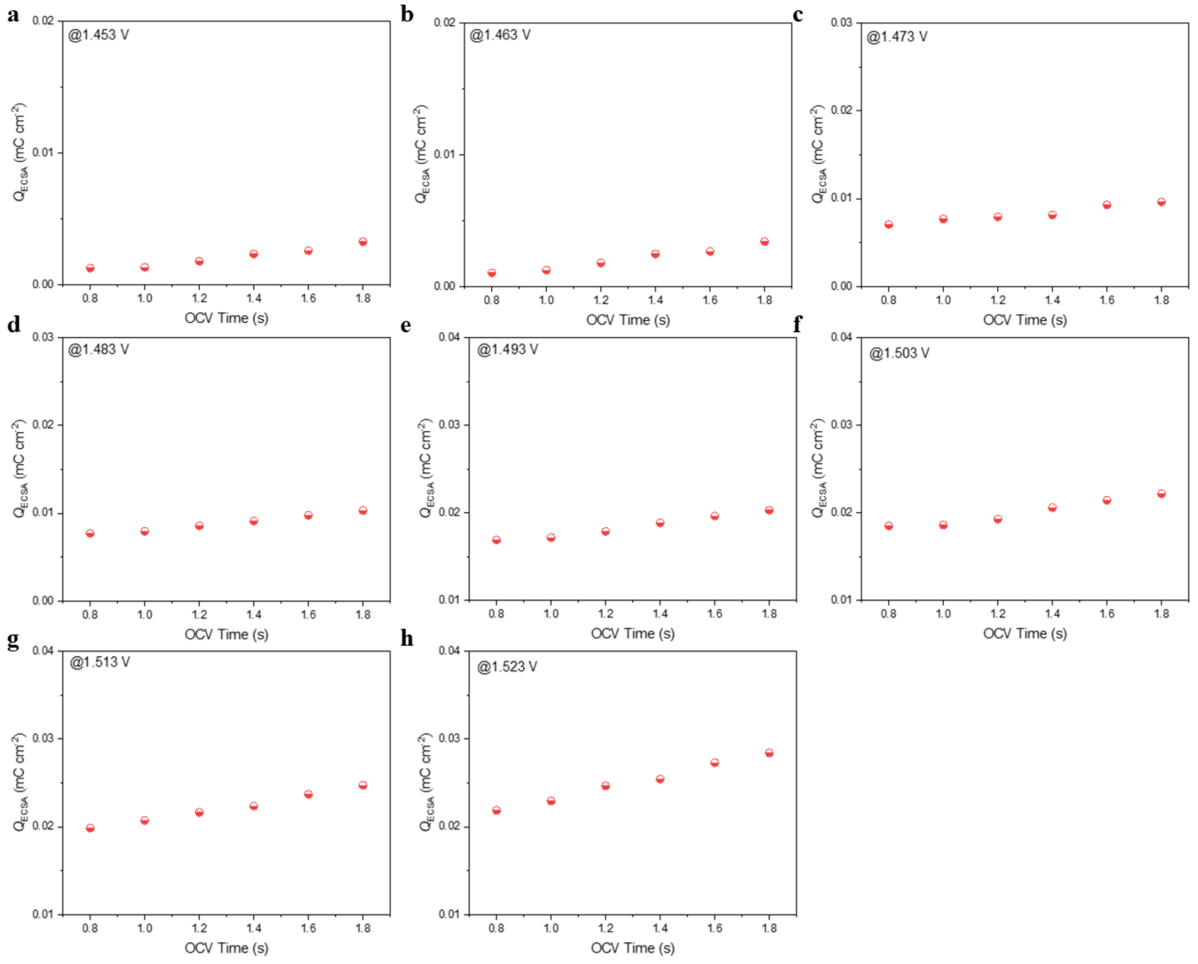

Supplementary Fig. 14. Estimated transferred charge normalized to ECSA of NiOOH during OCV time under each applied potential. (a) 1.453 V (b) 1.463 V (c) 1.473 V (d) 1.483 V (e) 1.493 V (f) 1.503 V (g) 1.513 V (h) 1.523 V. Source data are provided as a Source Data file.

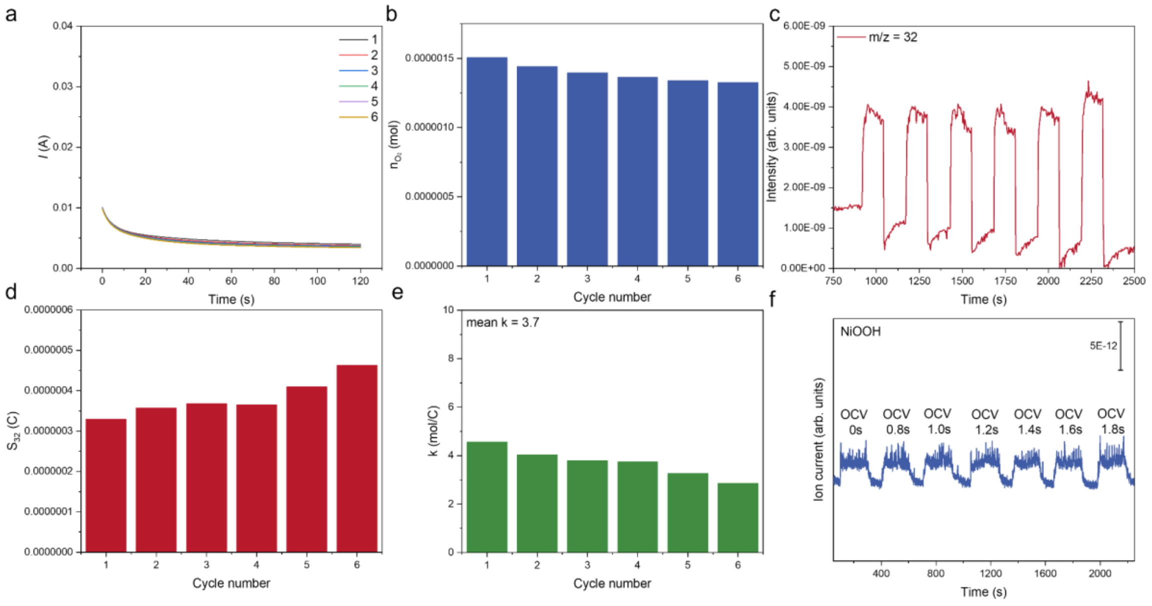

Supplementary Fig. 15. Calibration of the DEMS response and OCV-time-dependent DEMS measurements. (a) *I-t* curves recorded at 1.503 V vs. RHE for six repeated calibration cycles. (b) O_2 amount ($n(O_2)$) estimated from the integrated electrochemical charge in each calibration cycle. (c) Simultaneously recorded DEMS signal at m/z=32 during the calibration cycles. (d) Integrated baseline-corrected DEMS m/z=32 signal (S_{32}) for each calibration cycle. (e) Effective DEMS calibration factor k obtained from the corresponding electrochemical and DEMS integrals, with an average value of k=3.7 mol/C (f) DEMS m/z=32 signal recorded under identical pre-charging conditions while varying only the OCV duration (0, 0.8, 1.0, 1.2, 1.4, 1.6, and 1.8 s), used to evaluate the additional O_2 signal develops with increasing OCV time. Source data are provided as a Source Data file.

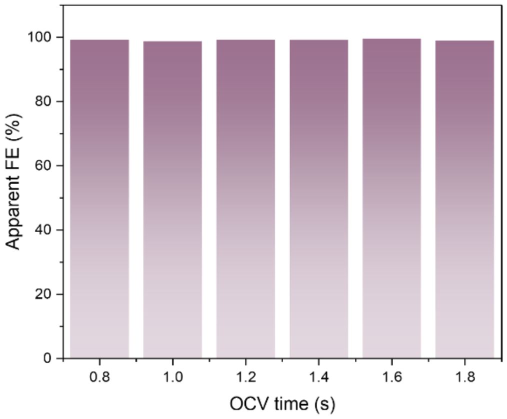

Supplementary Fig. 16. Apparent FE toward *OOH formation at different OCV durations for NiOOH. The charging bias is 1.503 V vs. RHE. Source data are provided as a Source Data file.

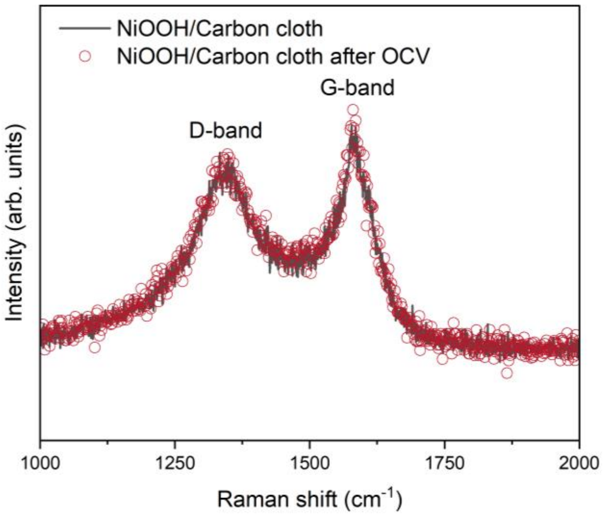

Supplementary Fig. 17. Raman spectra of NiOOH/Carbon cloth before and after OCV. Source data are provided as a Source Data file.

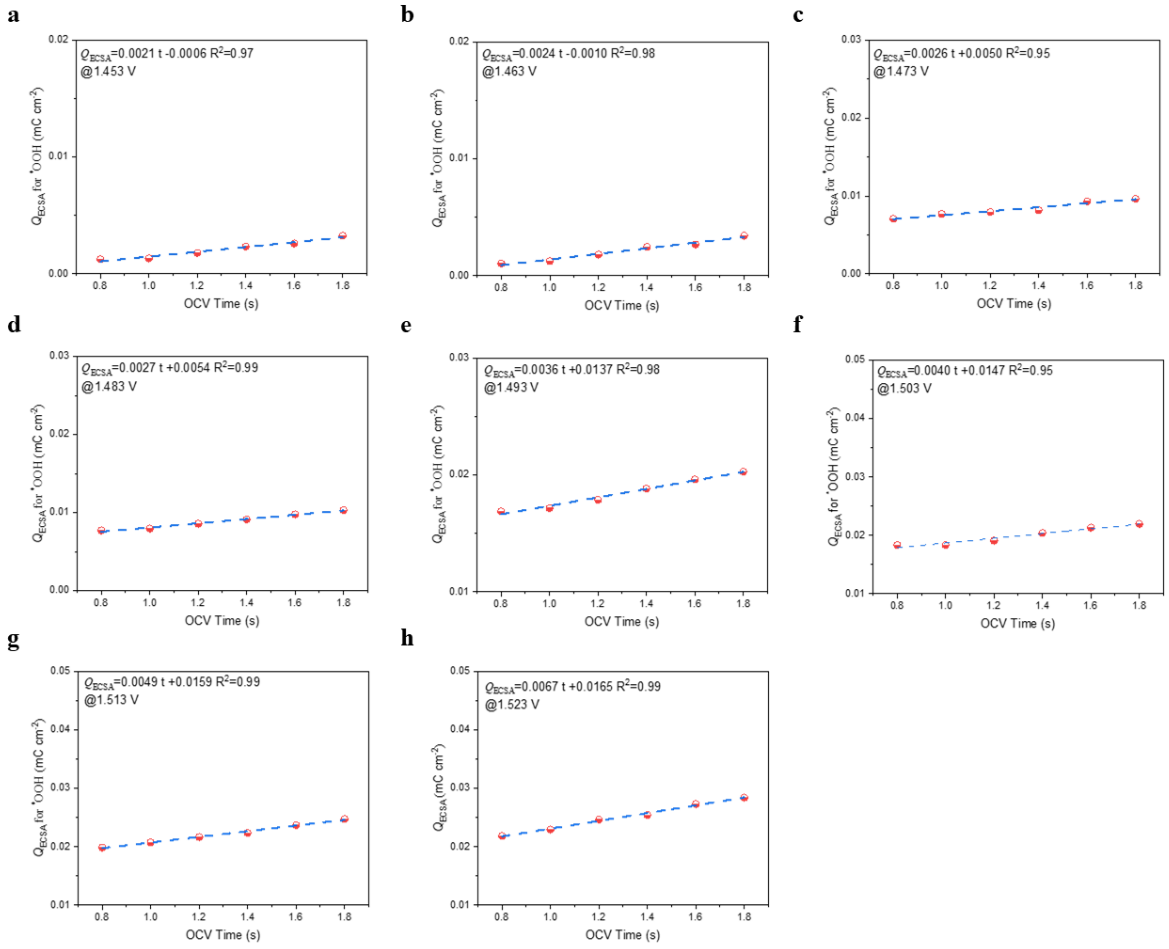

Supplementary Fig. 18. Estimated electron transfer rate related to *OOH formation under each applied potential. (a) 1.453 V (b) 1.463 V (c) 1.473 V (d) 1.483 V (e) 1.493 V (f) 1.503 V (g) 1.513 V (h) 1.523 V. Source data are provided as a Source Data file.

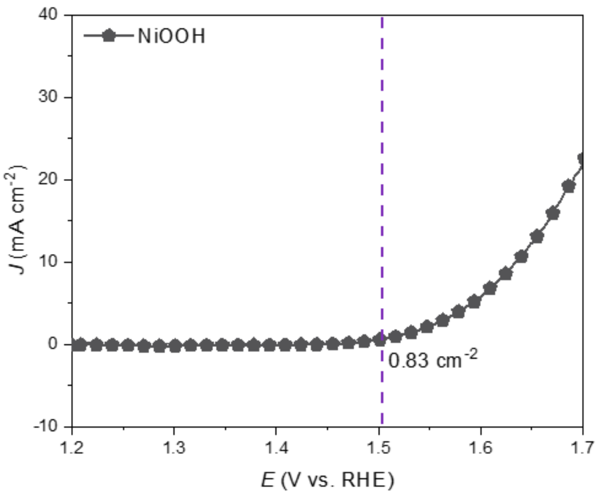

Supplementary Fig. 19. LSV characterization of NiOOH without iR compensation. Source data are provided as a Source Data file.

Compared to the current density, the quantified *OOH formation rate is within a reasonable range. For NiOOH, the current density at 1.503 V vs. RHE is 0.83 mA cm^{-2} (0.8 cm^2 area size). We assume that 0.83/4 = 0.20 mA cm^{-2} comes from *OOH formation due to the four electron transfer steps. After normalizing ECSA (27.5 cm^2), the current density is 5.81E-6 A cm^{-2}. Then the *OOH formation rate can be estimated via 5.81E-6*6.24E18/6.02E23=6.02E-8mmol cm^{-2} s^{-1}. The OCV-PV quantified *OOH formation rate for undoped NiOOH is 4.2E-8 mmol cm^{-2} s^{-1}, at the same magnitude. Therefore, this result proves the quantified *OOH formation rate is within a reasonable range.

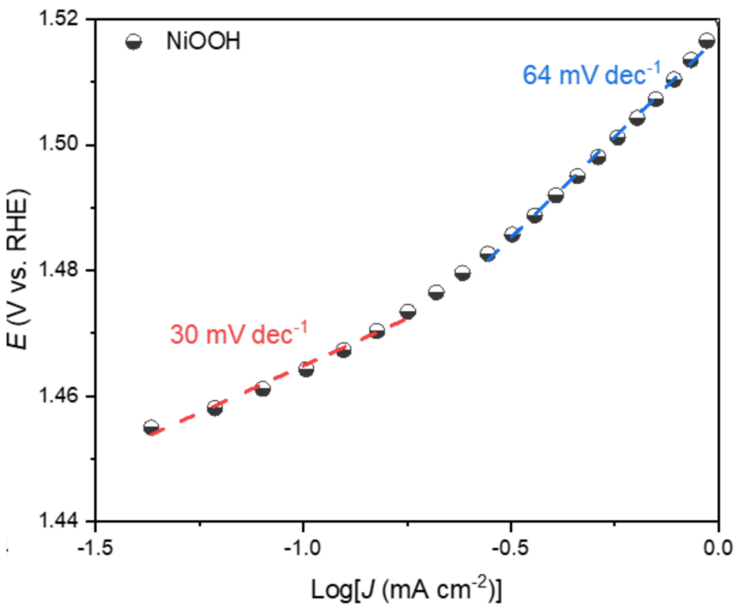

Supplementary Fig. 20. Tafel slope analysis of NiOOH. Source data are provided as a Source Data file.

As shown in Supplementary Figure 16, with increasing potential, the Tafel slopes exhibited two distinct values: 30 mV dec^{-1} and 64 mV dec^{-1}. According to the Tafel equation and its parameter calculations, a Tafel slope of 30 mV dec^{-1} is consistent with a chemical *OOH formation step and two electron transfers that precede this step.[1] In contrast, a Tafel slope of 64 mV dec^{-1} indicates a rate-limiting *OH deprotonation step and one electron transfer before this step. Therefore, the change in the Tafel slope could be attributed to the change in the RDS from *OOH formation to *OH deprotonation. In the low-potential region where the RDS is *OOH formation, the increase in its rate is limited by its high energy barrier. In the high-potential region, *OOH formation becomes much faster; hence, the RDS changes to *OH deprotonation. Therefore, the proposed OCV-PV method successfully reveals the fundamental reason for the change in the current response to the applied potential in the LSV curve, which is attributed to the RDS transition from the chemical step to the electrochemical step.

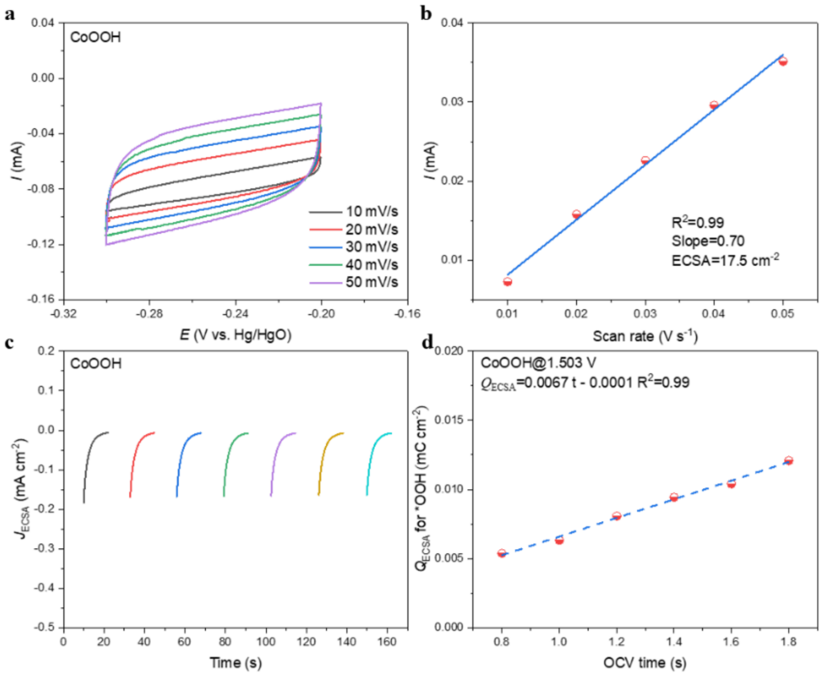

Supplementary Fig. 21. OCV–PV and PV measurements on CoOOH. (a) The Cyclic voltammograms curves of the CoOOH and corresponding current (I) vs. scan rate plots (b). Plots of current vs. scan rate data were obtained from CV curves at -0.25 V vs. Hg/HgO. (c) The reductive pulse with the current response in OCV-PV test for CoOOH under charging bias of 1.503 V. (d) *OOH formation-related charge versus OCV duration. Source data are provided as a Source Data file.

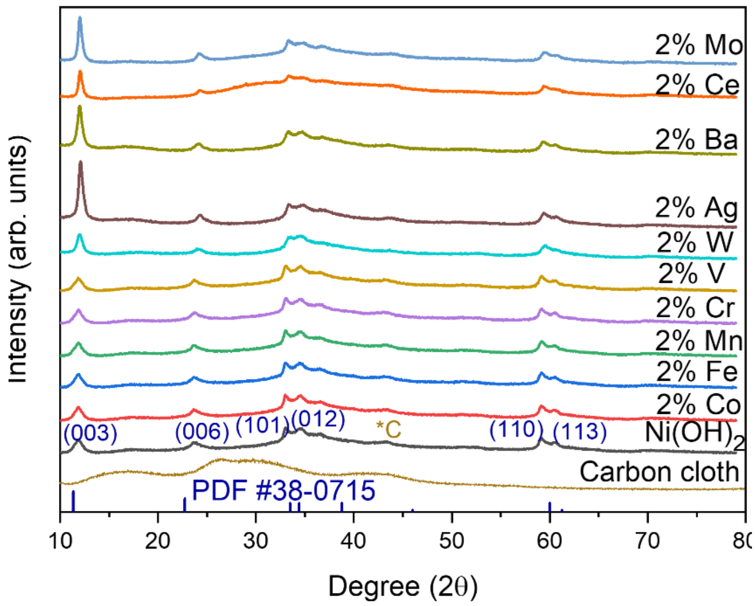

Supplementary Fig. 22. XRD patterns of $Ni(OH)_2$-based catalysts with various cation dopants (2 at%). Source data are provided as a Source Data file.

We note that slight peak shifts relative to the standard diffraction card are observed across the entire sample series, including the undoped $Ni(OH)_2$ reference. Such deviations are common for substrate-supported hydroxide films and do not indicate impurity phase formation. Importantly, no additional reflections or peak splitting associated with secondary crystalline phases are observed.

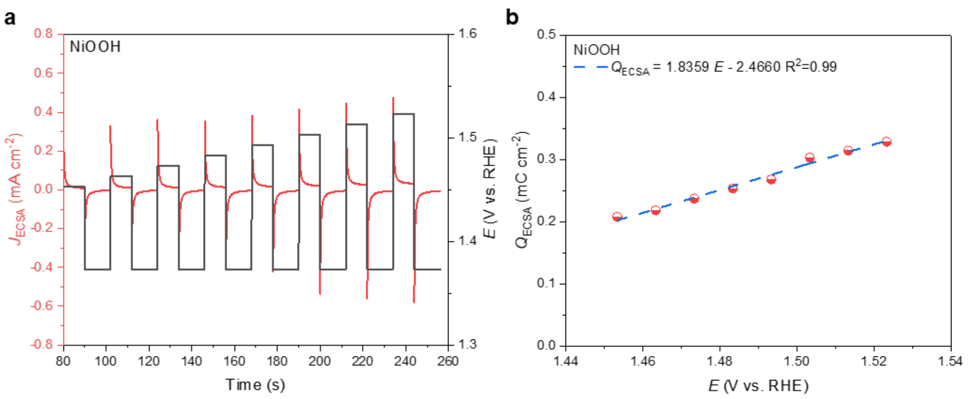

Supplementary Fig. 23. PV measurement on NiOOH to evaluate *OH deprotonation ability. (a) PV protocol (black) showing an oxidative and reductive pulse with the current response (red). (b). Charge versus potential from PV, where the fitted slope is used to represent the *OH deprotonation ability. Source data are provided as a Source Data file.

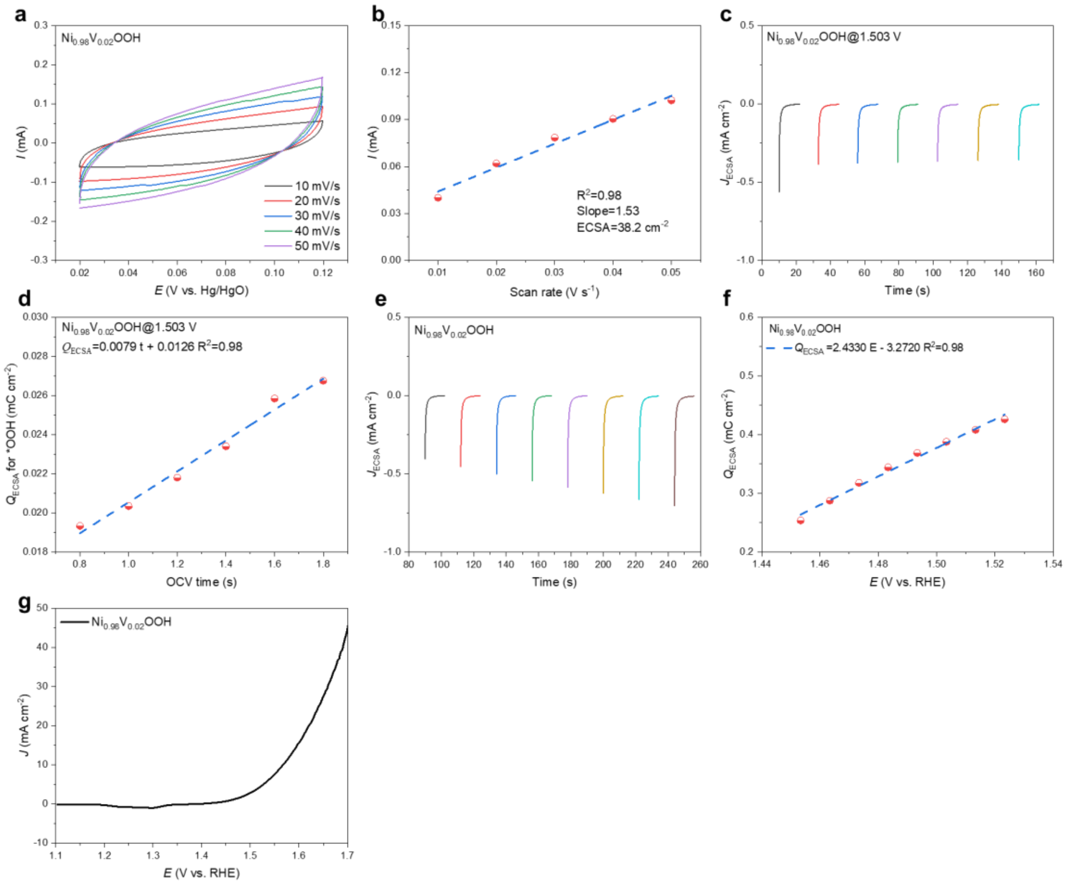

Supplementary Fig. 24. OCV–PV and PV measurements on $Ni_{0.98}V_{0.02}OOH$. (a) The Cyclic voltammograms curves of the $Ni_{0.98}V_{0.02}OOH$ and corresponding current (*I*) vs. scan rate plots (b). Plots of current vs. scan rate data were obtained from CV curves at 0.07 V vs. Hg/HgO. (c) The reductive pulse with the current response in OCV-PV test for $Ni_{0.98}V_{0.02}OOH$ under charging bias of 1.503 V. (d) $^*OOH$ formation-related charge versus OCV duration. (e) The reductive pulse with the current response in PV test for $Ni_{0.98}V_{0.02}OOH$. (f) $^*OH$ deprotonation-related charge versus charging bias. (g) LSV curve of $Ni_{0.98}V_{0.02}OOH$ without iR compensation. Source data are provided as a Source Data file.

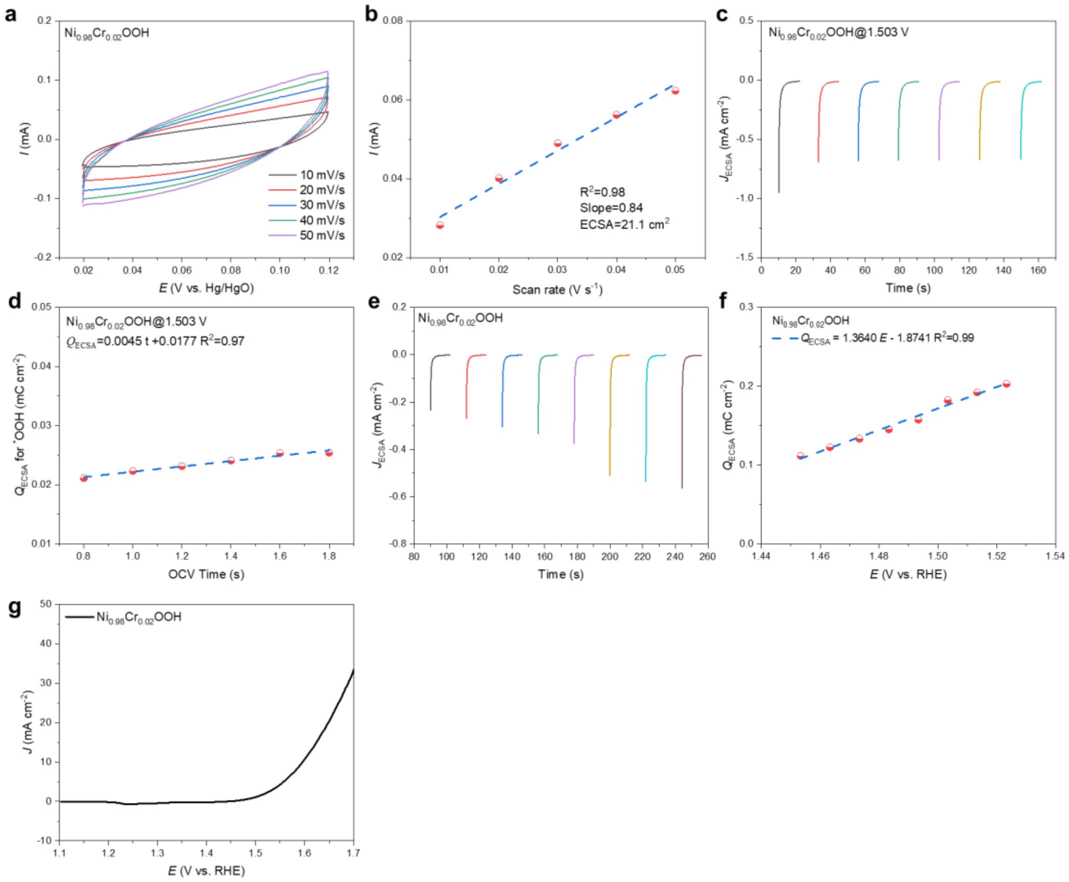

Supplementary Fig. 25. OCV–PV and PV measurements on $Ni_{0.98}Cr_{0.02}OOH$. (a) The Cyclic voltammograms curves of the $Ni_{0.98}Cr_{0.02}OOH$ and corresponding current (*I*) vs. scan rate plots (b). Plots of current vs. scan rate data were obtained from CV curves at 0.07 V vs. Hg/HgO. (c) The reductive pulse with the current response in OCV-PV test for $Ni_{0.98}Cr_{0.02}OOH$ under charging bias of 1.503 V. (d) *OOH formation-related charge versus OCV duration. (e) The reductive pulse with the current response in PV test for $Ni_{0.98}Cr_{0.02}OOH$. (f) *OH deprotonation-related charge versus charging bias. (g) LSV curve of $Ni_{0.98}Cr_{0.02}OOH$ without iR compensation. Source data are provided as a Source Data file.

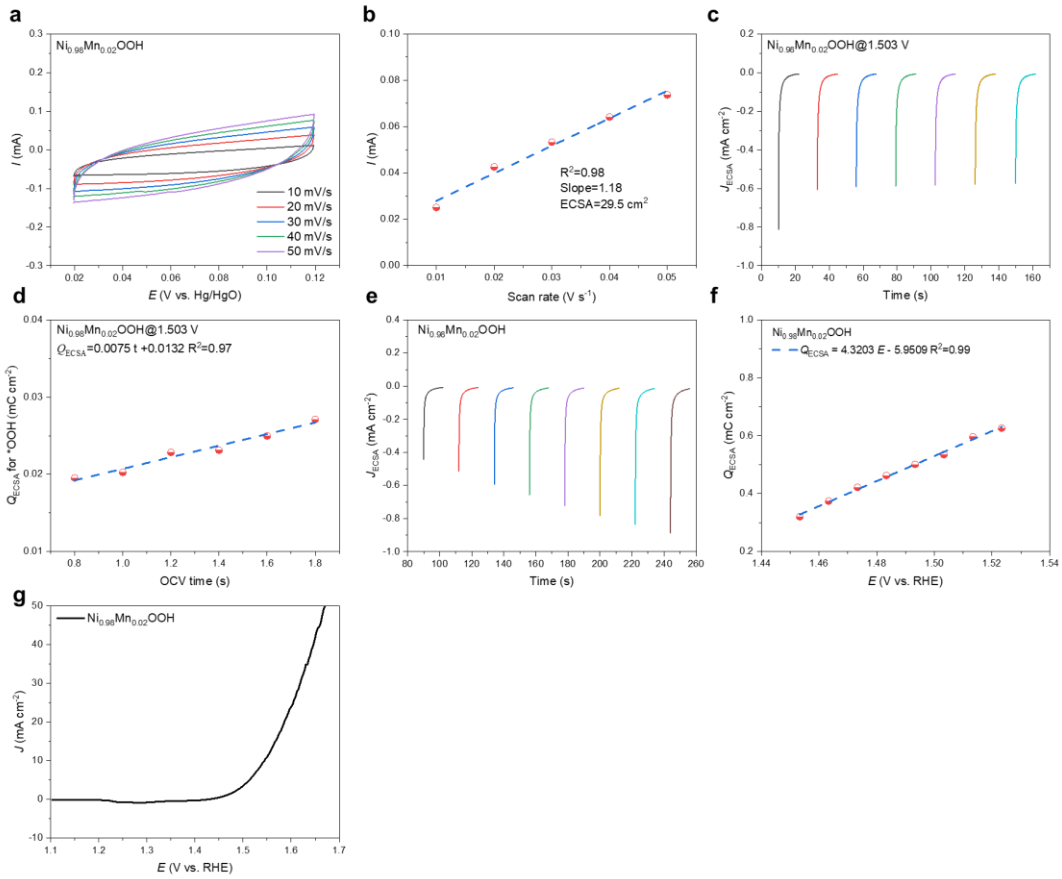

Supplementary Fig. 26. OCV–PV and PV measurements on $Ni_{0.98}Mn_{0.02}OOH$. (a) The Cyclic voltammograms curves of the $Ni_{0.98}Mn_{0.02}OOH$ and corresponding current (I) vs. scan rate plots (b). Plots of current vs. scan rate data were obtained from CV curves at 0.07 V vs. Hg/HgO. (c) The reductive pulse with the current response in OCV-PV test for $Ni_{0.98}Mn_{0.02}OOH$ under charging bias of 1.503 V. (d) *OOH formation-related charge versus OCV duration. (e) The reductive pulse with the current response in PV test for $Ni_{0.98}Mn_{0.02}OOH$. (f) *OH deprotonation-related charge versus charging bias. (g) LSV curve of $Ni_{0.98}Mn_{0.02}OOH$ without iR compensation. Source data are provided as a Source Data file.

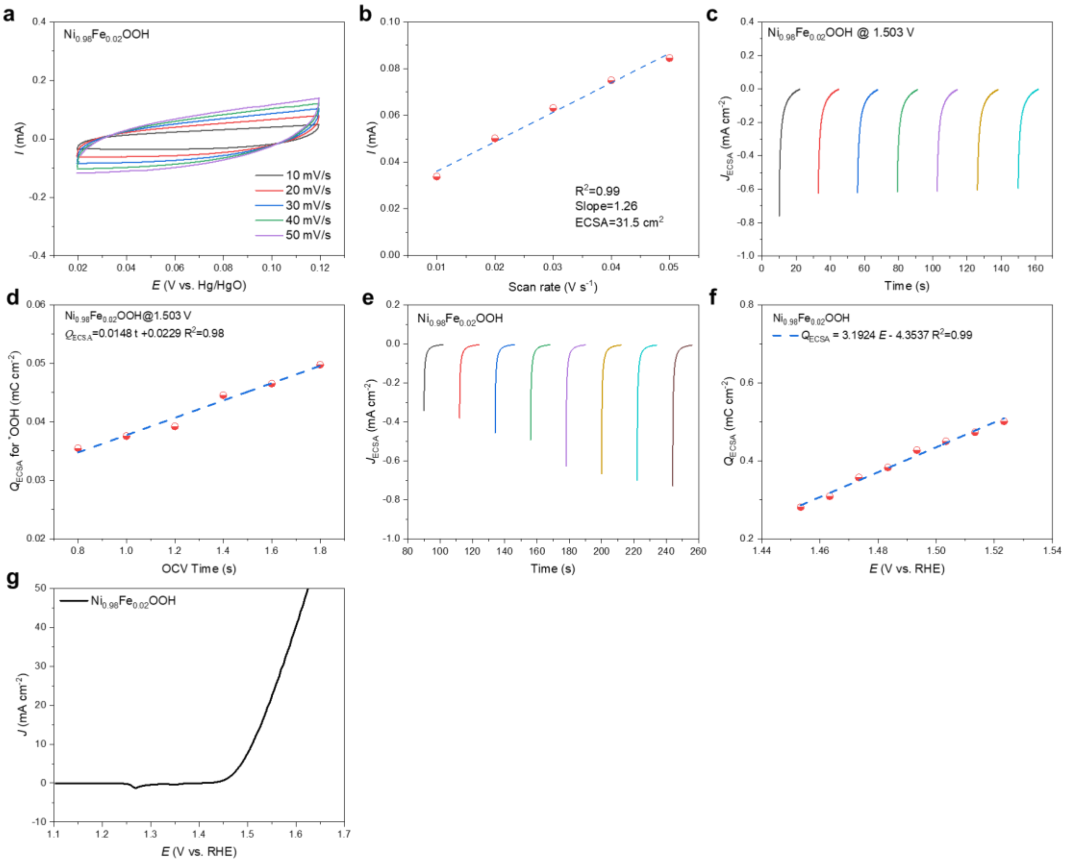

Supplementary Fig. 27. OCV–PV and PV measurements on $Ni_{0.98}Fe_{0.02}OOH$. (a) The Cyclic voltammograms curves of the $Ni_{0.98}Fe_{0.02}OOH$ and corresponding current (I) vs. scan rate plots (b). Plots of current vs. scan rate data were obtained from CV curves at 0.07 V vs. Hg/HgO. (c) The reductive pulse with the current response in OCV-PV test for $Ni_{0.98}Fe_{0.02}OOH$ under charging bias of 1.503 V. (d) *OOH formation-related charge versus OCV duration. (e) The reductive pulse with the current response in PV test for $Ni_{0.98}Fe_{0.02}OOH$. (f) *OH deprotonation-related charge versus charging bias. (g) LSV curve of $Ni_{0.98}Fe_{0.02}OOH$ without iR compensation. Source data are provided as a Source Data file.

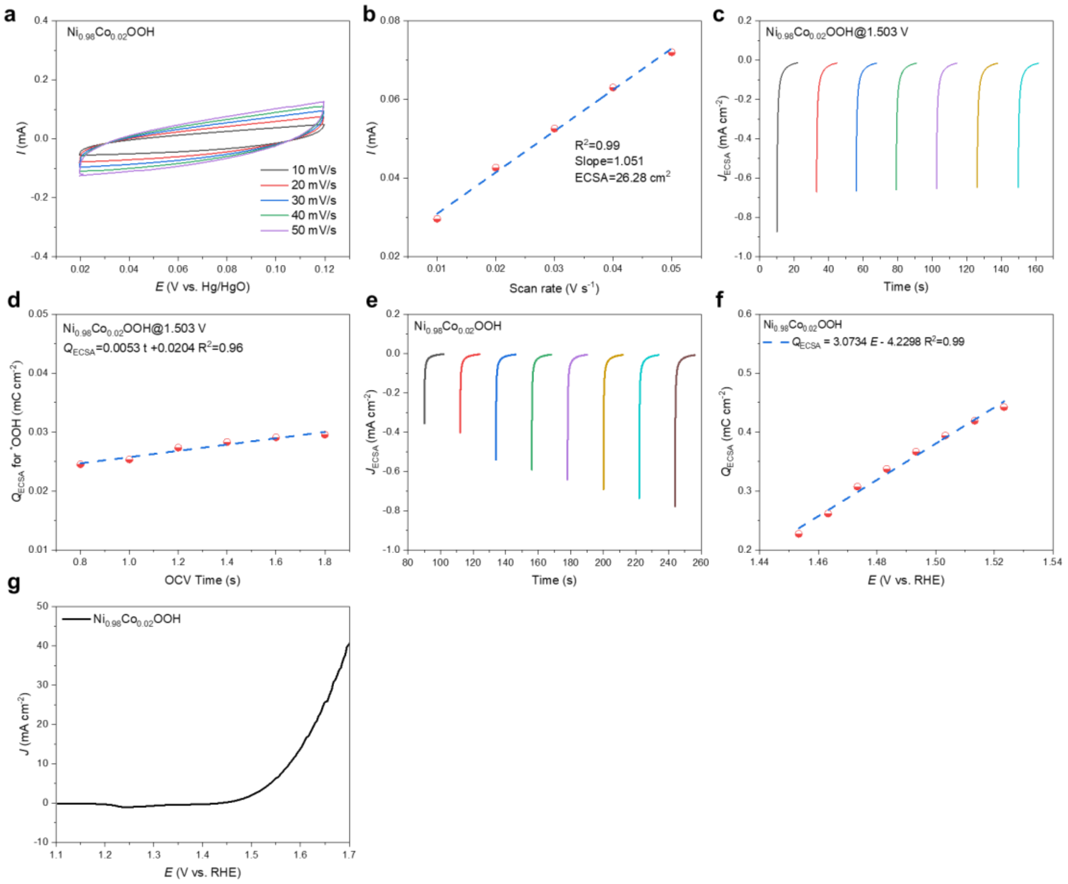

Supplementary Fig. 28. OCV–PV and PV measurements on $Ni_{0.98}Co_{0.02}OOH$. (a) The Cyclic voltammograms curves of the $Ni_{0.98}Co_{0.02}OOH$ and corresponding current (I) vs. scan rate plots (b). Plots of current vs. scan rate data were obtained from CV curves at 0.07 V vs. Hg/HgO. (c) The reductive pulse with the current response in OCV-PV test for $Ni_{0.98}Co_{0.02}OOH$ under charging bias of 1.503 V. (d) $^*OOH$ formation-related charge versus OCV duration. (e) The reductive pulse with the current response in PV test for $Ni_{0.98}Co_{0.02}OOH$. (f) $^*OH$ deprotonation-related charge versus charging bias. (g) LSV curve of $Ni_{0.98}Co_{0.02}OOH$ without iR compensation. Source data are provided as a Source Data file.

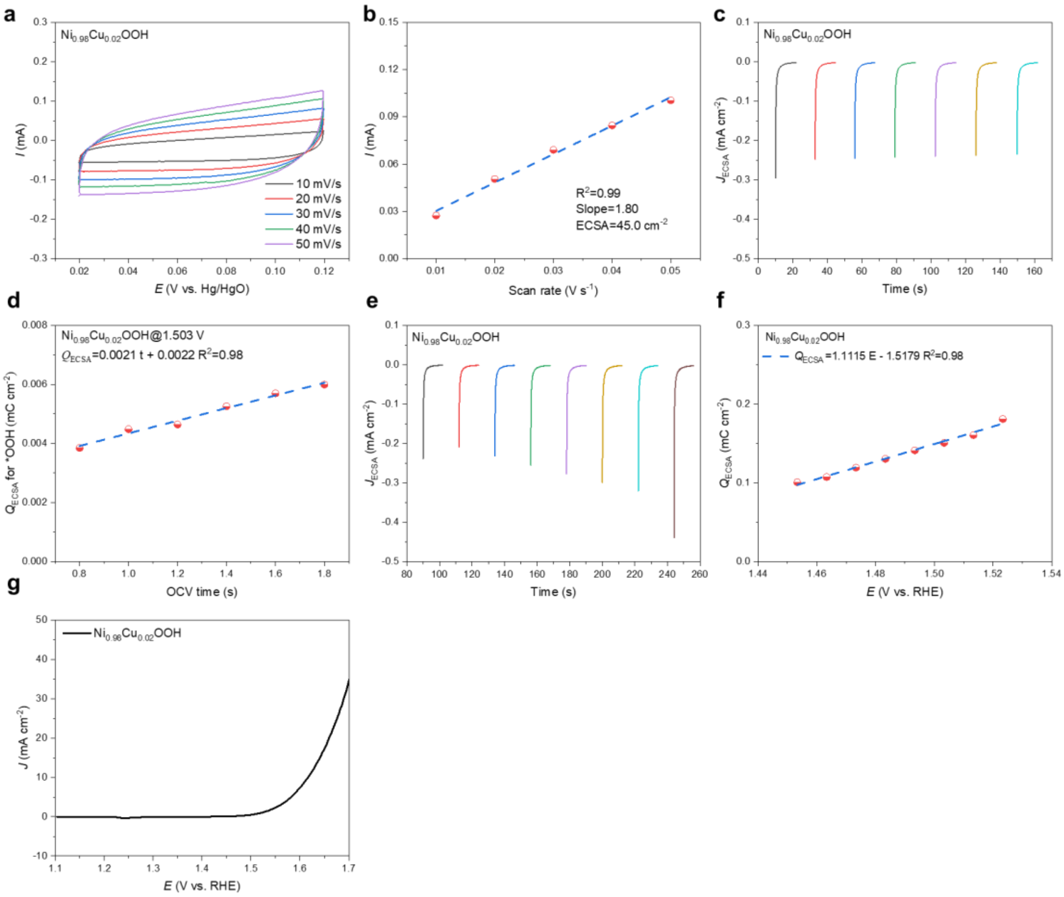

Supplementary Fig. 29. OCV–PV and PV measurements on $Ni_{0.98}Cu_{0.02}OOH$. (a) The Cyclic voltammograms curves of the $Ni_{0.98}Cu_{0.02}OOH$ and corresponding current (*I*) vs. scan rate plots (b). Plots of current vs. scan rate data were obtained from CV curves at 0.07 V vs. Hg/HgO. (c) The reductive pulse with the current response in OCV-PV test for $Ni_{0.98}Cu_{0.02}OOH$ under charging bias of 1.503 V. (d) $^{*}OOH$ formation-related charge versus OCV duration. (e) The reductive pulse with the current response in PV test for $Ni_{0.98}Cu_{0.02}OOH$. (f) $^{*}OH$ deprotonation-related charge versus charging bias. (g) LSV curve of $Ni_{0.98}Cu_{0.02}OOH$ without iR compensation. Source data are provided as a Source Data file.

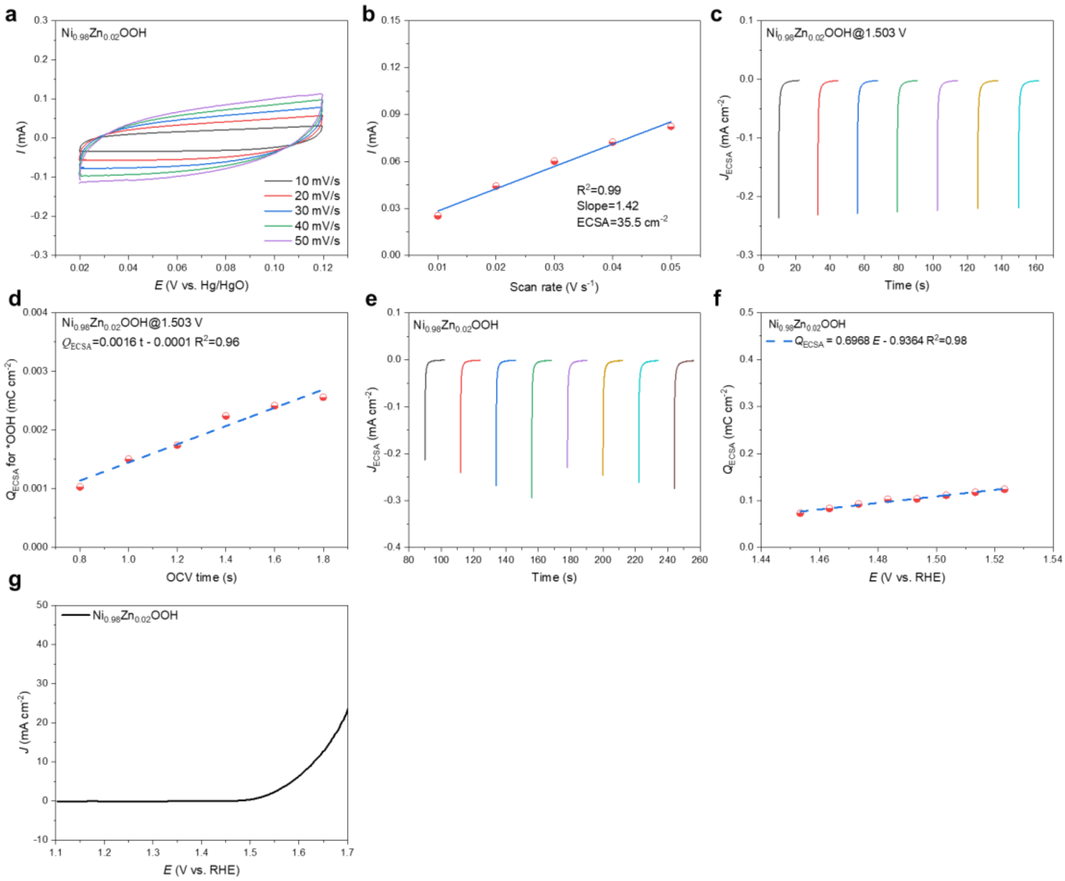

Supplementary Fig. 30. OCV–PV and PV measurements on $Ni_{0.98}Zn_{0.02}OOH$. (a) The Cyclic voltammograms curves of the $Ni_{0.98}Zn_{0.02}OOH$ and corresponding current (*I*) vs. scan rate plots (b). Plots of current vs. scan rate data were obtained from CV curves at 0.07 V vs. Hg/HgO. (c) The reductive pulse with the current response in OCV-PV test for $Ni_{0.98}Zn_{0.02}OOH$ under charging bias of 1.503 V. (d) $^*OOH$ formation-related charge versus OCV duration. (e) The reductive pulse with the current response in PV test for $Ni_{0.98}Zn_{0.02}OOH$. (f) $^*OH$ deprotonation-related charge versus charging bias. (g) LSV curve of $Ni_{0.98}Zn_{0.02}OOH$ without iR compensation. Source data are provided as a Source Data file.

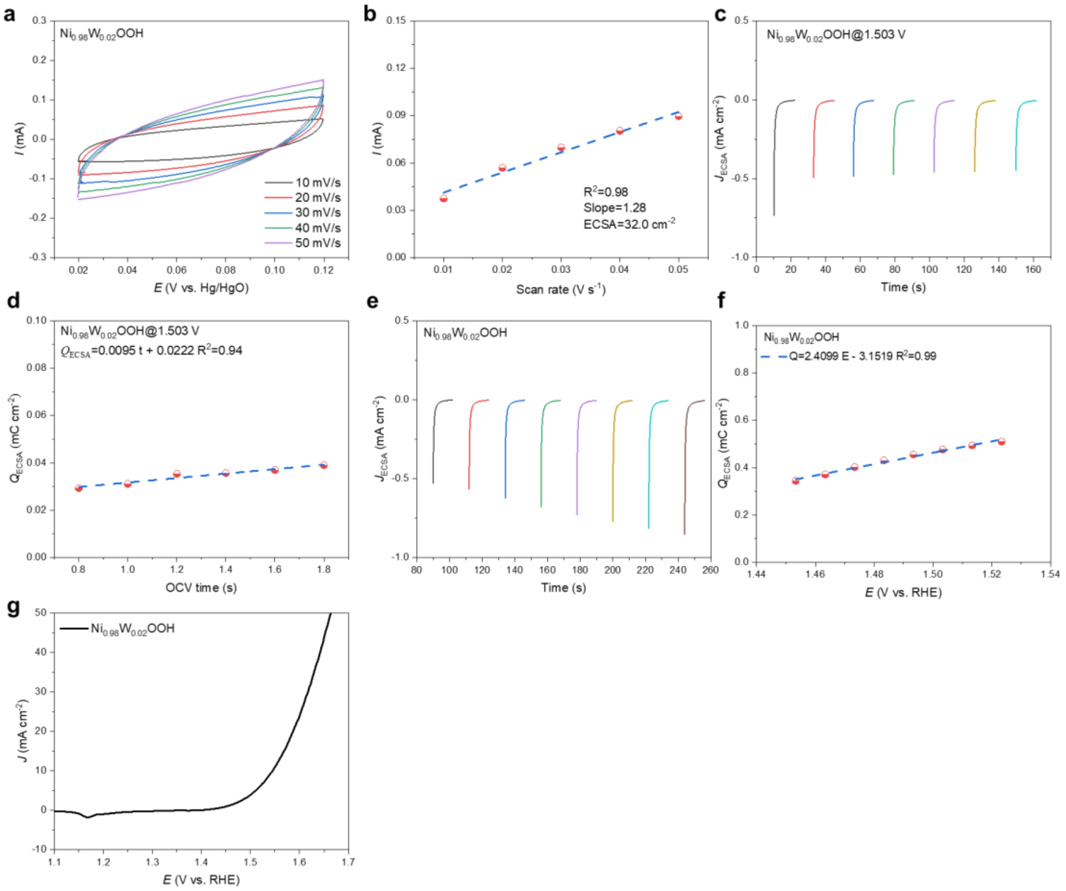

Supplementary Fig. 31. OCV–PV and PV measurements on $Ni_{0.98}W_{0.02}OOH$. (a) The Cyclic voltammograms curves of the $Ni_{0.98}W_{0.02}OOH$ and corresponding current (*I*) vs. scan rate plots (b). Plots of current vs. scan rate data were obtained from CV curves at 0.07 V vs. Hg/HgO. (c) The reductive pulse with the current response in OCV-PV test for $Ni_{0.98}W_{0.02}OOH$ under charging bias of 1.503 V. (d) *OOH formation-related charge versus OCV duration. (e) The reductive pulse with the current response in PV test for $Ni_{0.98}W_{0.02}OOH$. (f) *OH deprotonation-related charge versus charging bias. (g) LSV curve of $Ni_{0.98}W_{0.02}OOH$ without iR compensation. Source data are provided as a Source Data file.

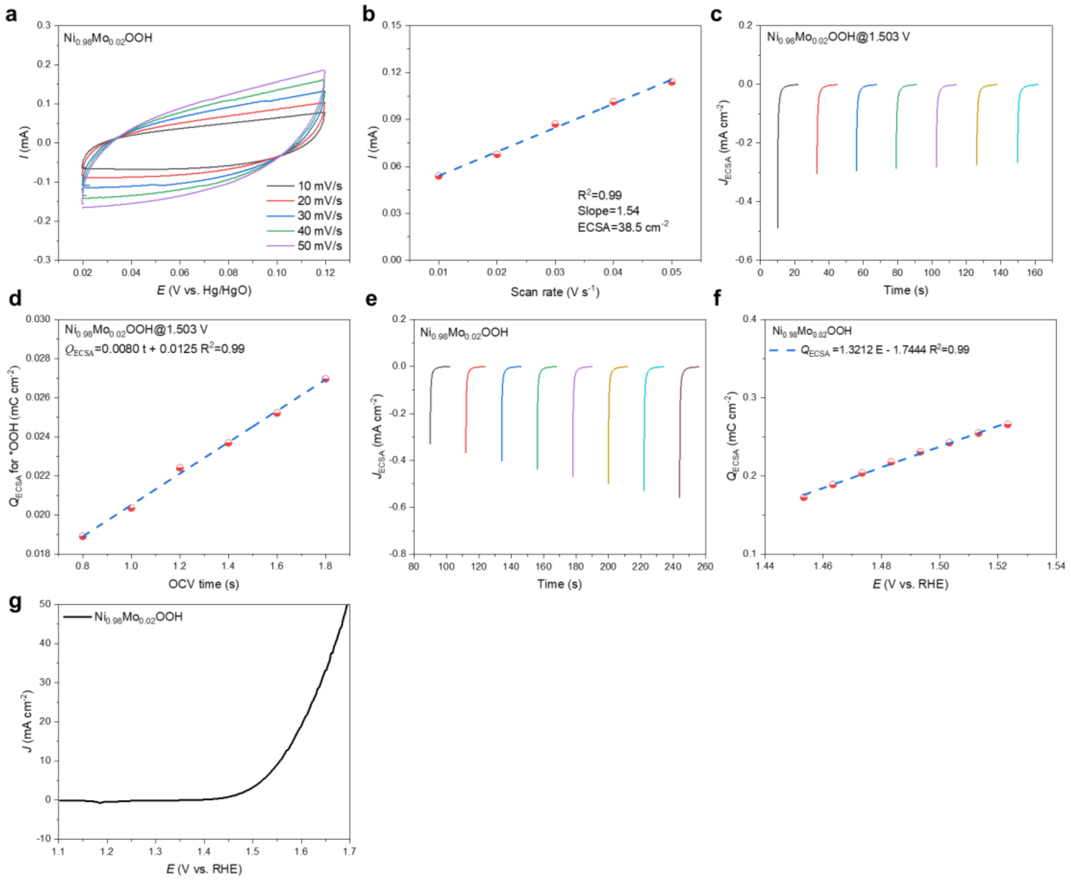

Supplementary Fig. 32. OCV–PV and PV measurements on $Ni_{0.98}Mo_{0.02}OOH$. (a) The Cyclic voltammograms curves of the $Ni_{0.98}Mo_{0.02}OOH$ and corresponding current (*I*) vs. scan rate plots (b). Plots of current vs. scan rate data were obtained from CV curves at 0.07 V vs. Hg/HgO. (c) The reductive pulse with the current response in OCV-PV test for $Ni_{0.98}Mo_{0.02}OOH$ under charging bias of 1.503 V. (d) $^*OOH$ formation-related charge versus OCV duration. (e) The reductive pulse with the current response in PV test for $Ni_{0.98}Mo_{0.02}OOH$. (f) $^*OH$ deprotonation-related charge versus charging bias. (g) LSV curve of $Ni_{0.98}Mo_{0.02}OOH$ without iR compensation. Source data are provided as a Source Data file.

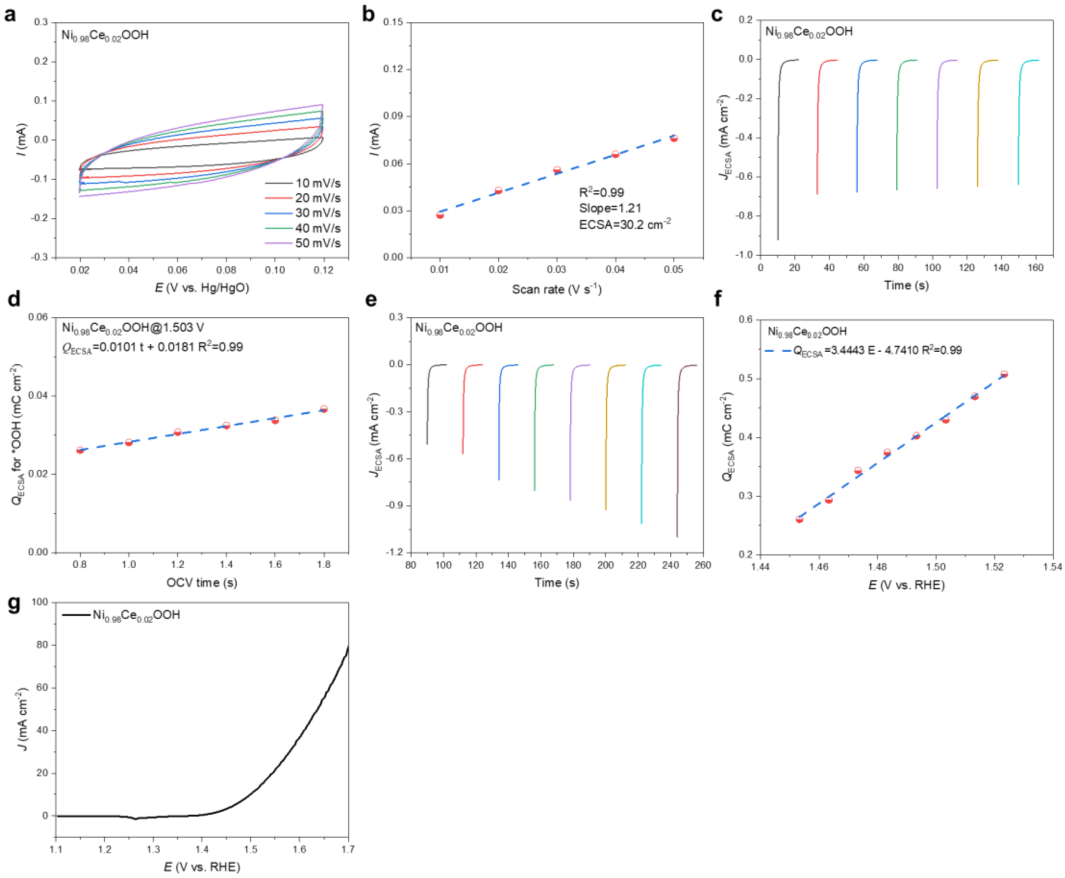

Supplementary Fig. 33. OCV–PV and PV measurements on $Ni_{0.98}Ce_{0.02}OOH$. (a) The Cyclic voltammograms curves of the $Ni_{0.98}Ce_{0.02}OOH$ and corresponding current (*I*) vs. scan rate plots (b). Plots of current vs. scan rate data were obtained from CV curves at 0.07 V vs. Hg/HgO. (c) The reductive pulse with the current response in OCV-PV test for $Ni_{0.98}Ce_{0.02}OOH$ under charging bias of 1.503 V. (d) $^*OOH$ formation-related charge versus OCV duration. (e) The reductive pulse with the current response in PV test for $Ni_{0.98}Ce_{0.02}OOH$. (f) $^*OH$ deprotonation-related charge versus charging bias. (g) LSV curve of $Ni_{0.98}Ce_{0.02}OOH$ without iR compensation. Source data are provided as a Source Data file.

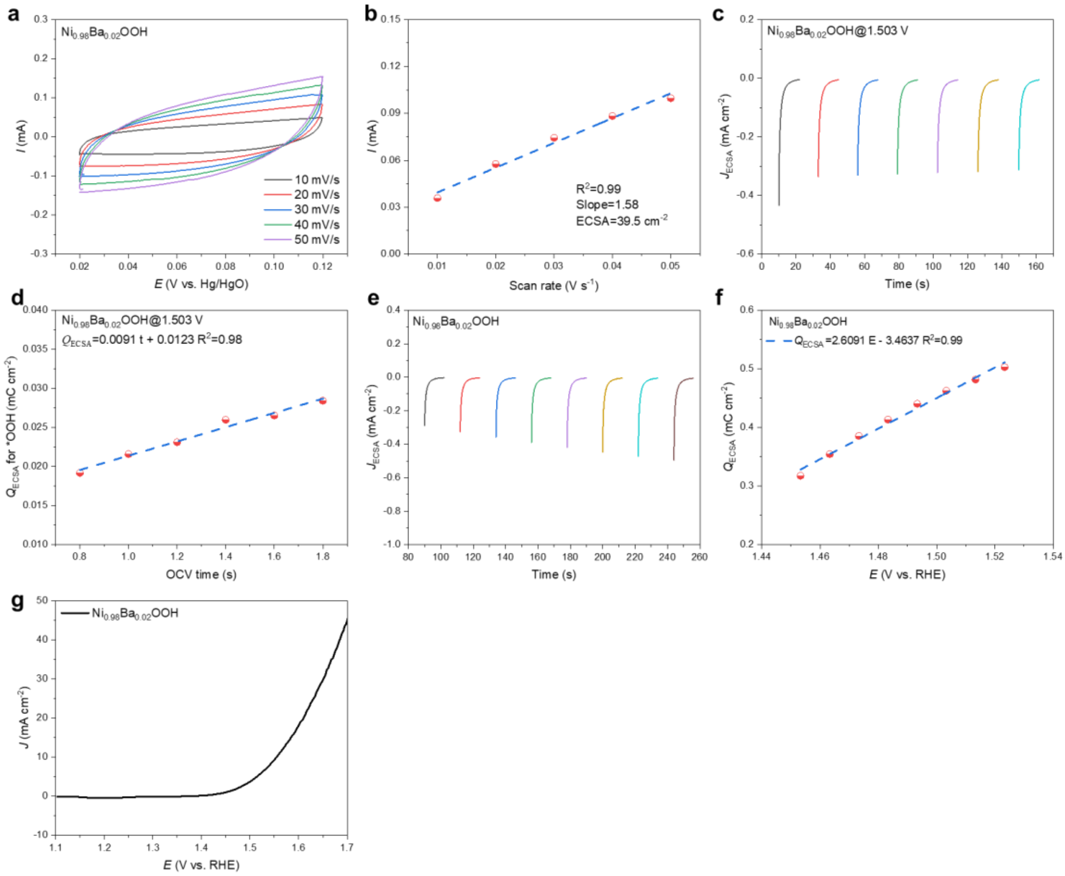

Supplementary Fig. 34. OCV–PV and PV measurements on $Ni_{0.98}Ba_{0.02}OOH$. (a) The Cyclic voltammograms curves of the $Ni_{0.98}Ba_{0.02}OOH$ and corresponding current (*I*) vs. scan rate plots (b). Plots of current vs. scan rate data were obtained from CV curves at 0.07 V vs. Hg/HgO. (c) The reductive pulse with the current response in OCV-PV test for $Ni_{0.98}Ba_{0.02}OOH$ under charging bias of 1.503 V. (d) $^{*}OOH$ formation-related charge versus OCV duration. (e) The reductive pulse with the current response in PV test for $Ni_{0.98}Ba_{0.02}OOH$. (f) $^{*}OH$ deprotonation-related charge versus charging bias. (g) LSV curve of $Ni_{0.98}Ba_{0.02}OOH$ without iR compensation. Source data are provided as a Source Data file.

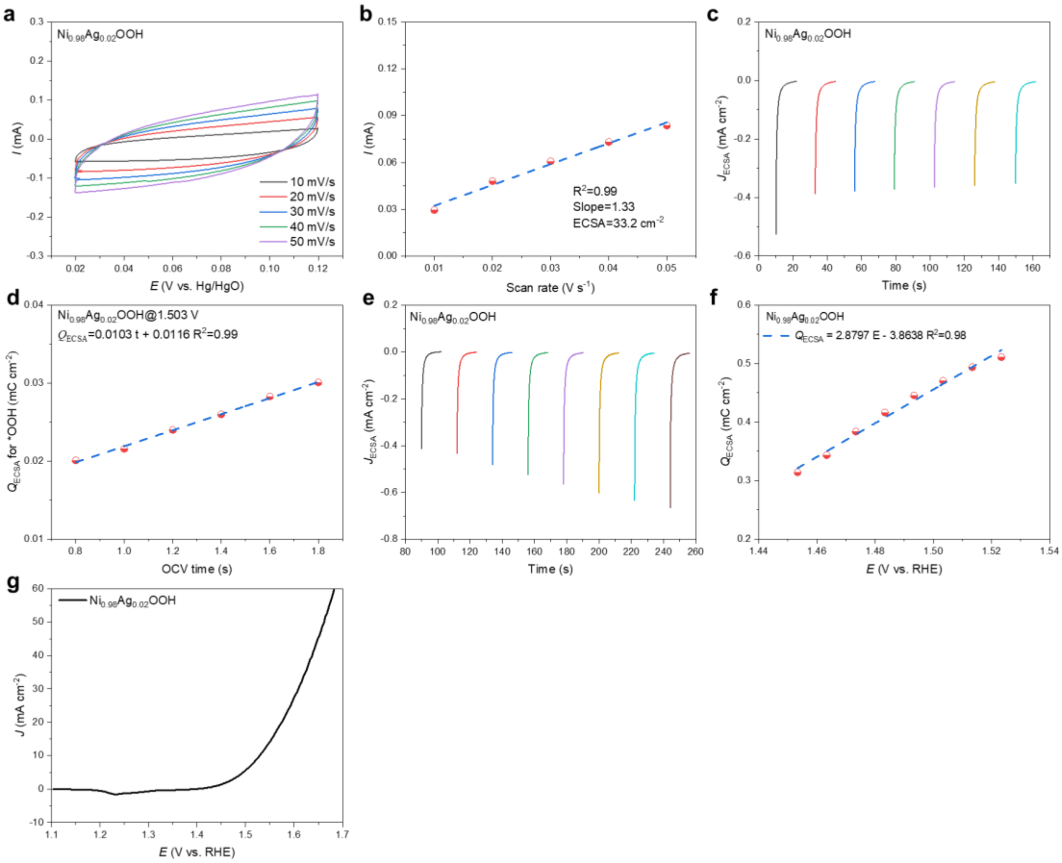

Supplementary Fig. 35. OCV–PV and PV measurements on $Ni_{0.98}Ag_{0.02}OOH$. (a) The Cyclic voltammograms curves of the $Ni_{0.98}Ag_{0.02}OOH$ and corresponding current (*I*) vs. scan rate plots (b). Plots of current vs. scan rate data were obtained from CV curves at 0.07 V vs. Hg/HgO. (c) The reductive pulse with the current response in OCV-PV test for $Ni_{0.98}Ag_{0.02}OOH$ under charging bias of 1.503 V. (d) *OOH formation-related charge versus OCV duration. (e) The reductive pulse with the current response in PV test for $Ni_{0.98}Ag_{0.02}OOH$. (f) *OH deprotonation-related charge versus charging bias. (g) LSV curve of $Ni_{0.98}Ag_{0.02}OOH$ without iR compensation. Source data are provided as a Source Data file.

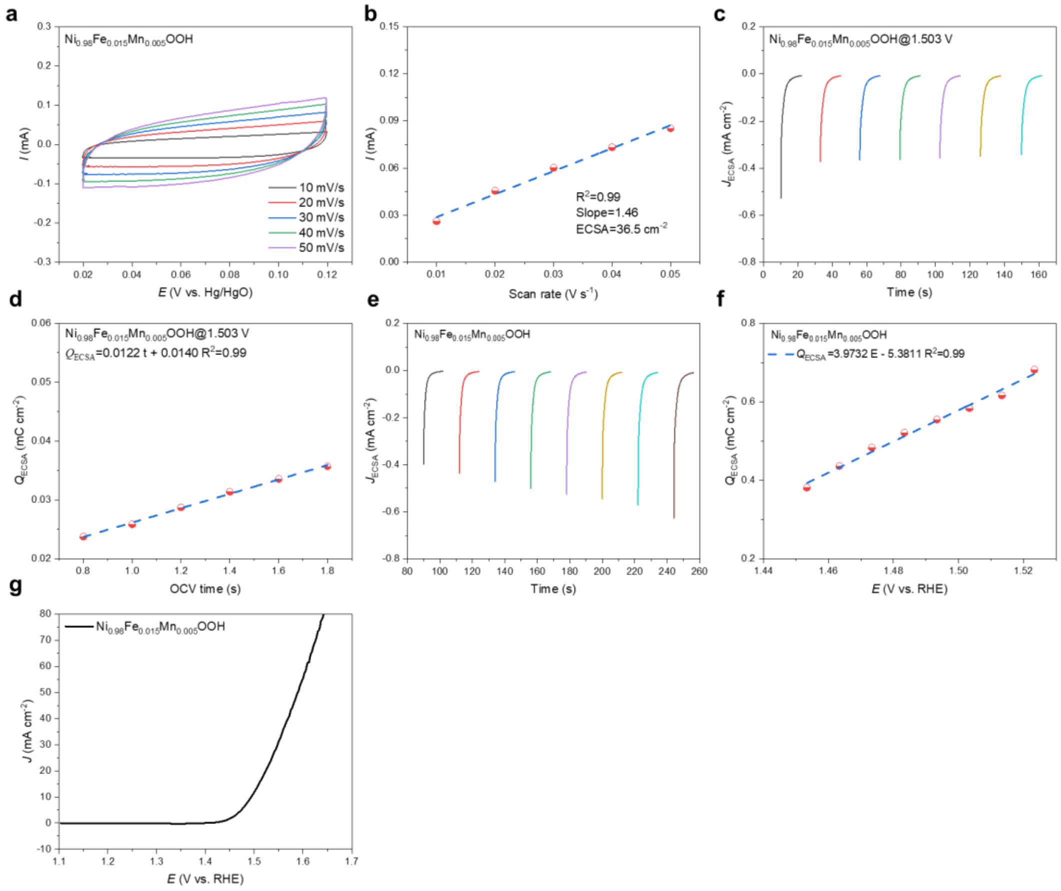

Supplementary Fig. 36. OCV–PV and PV measurements on $Ni_{0.98}Fe_{0.015}Mn_{0.005}OOH$. (a) The Cyclic voltammograms curves of the $Ni_{0.98}Fe_{0.015}Mn_{0.005}OOH$ and corresponding current (*I*) vs. scan rate plots (b). Plots of current vs. scan rate data were obtained from CV curves at 0.07 V vs. Hg/HgO. (c) The reductive pulse with the current response in OCV-PV test for $Ni_{0.98}Fe_{0.015}Mn_{0.005}OOH$ under charging bias of 1.503 V. (d) *OOH formation-related charge versus OCV duration. (e) The reductive pulse with the current response in PV test for $Ni_{0.98}Fe_{0.015}Mn_{0.005}OOH$. (f) *OH deprotonation-related charge versus charging bias. (g) LSV curve of $Ni_{0.98}Fe_{0.015}Mn_{0.005}OOH$ without iR compensation. Source data are provided as a Source Data file.

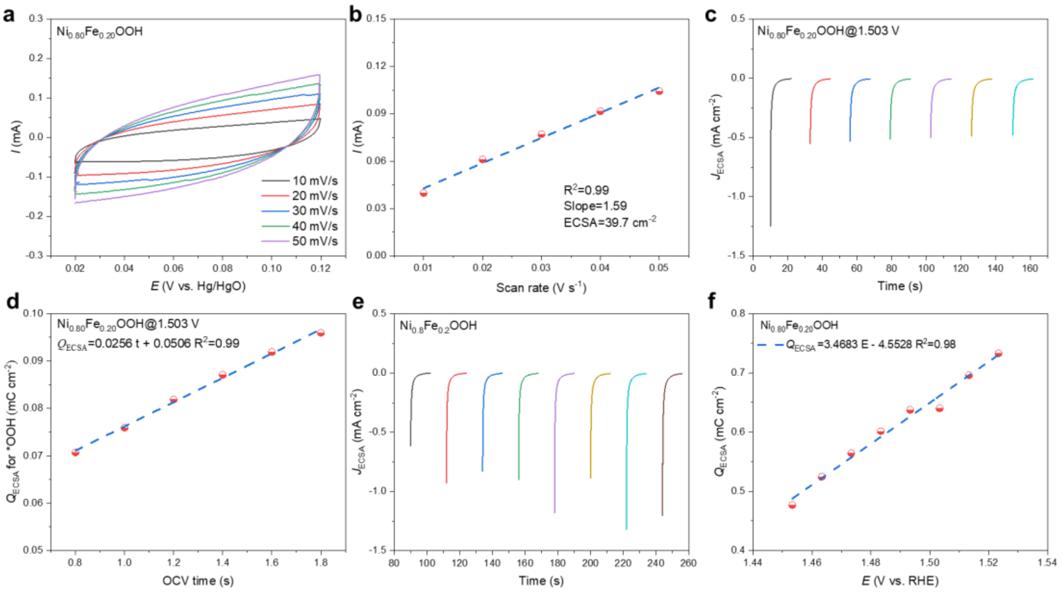

Supplementary Fig. 37. OCV–PV and PV measurements on $Ni_{0.80}Fe_{0.2}OOH$. (a) The Cyclic voltammograms curves of the $Ni_{0.80}Fe_{0.2}OOH$ and corresponding current (I) vs. scan rate plots (b). Plots of current vs. scan rate data were obtained from CV curves at 0.07 V vs. Hg/HgO. (c) The reductive pulse with the current response in OCV-PV test for $Ni_{0.80}Fe_{0.2}OOH$ under charging bias of 1.503 V. (d) *OOH formation-related charge versus OCV duration. (e) The reductive pulse with the current response in PV test for $Ni_{0.80}Fe_{0.2}OOH$. (f) *OH deprotonation-related charge versus charging bias. Source data are provided as a Source Data file.

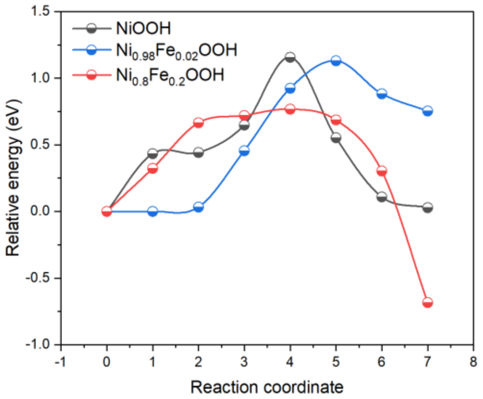

Supplementary Fig. 38. Simulation on the *OOH formation barriers for NiOOH, $Ni_{0.98}Fe_{0.02}OOH$, and $Ni_{0.8}Fe_{0.2}OOH$. Source data are provided as a Source Data file.

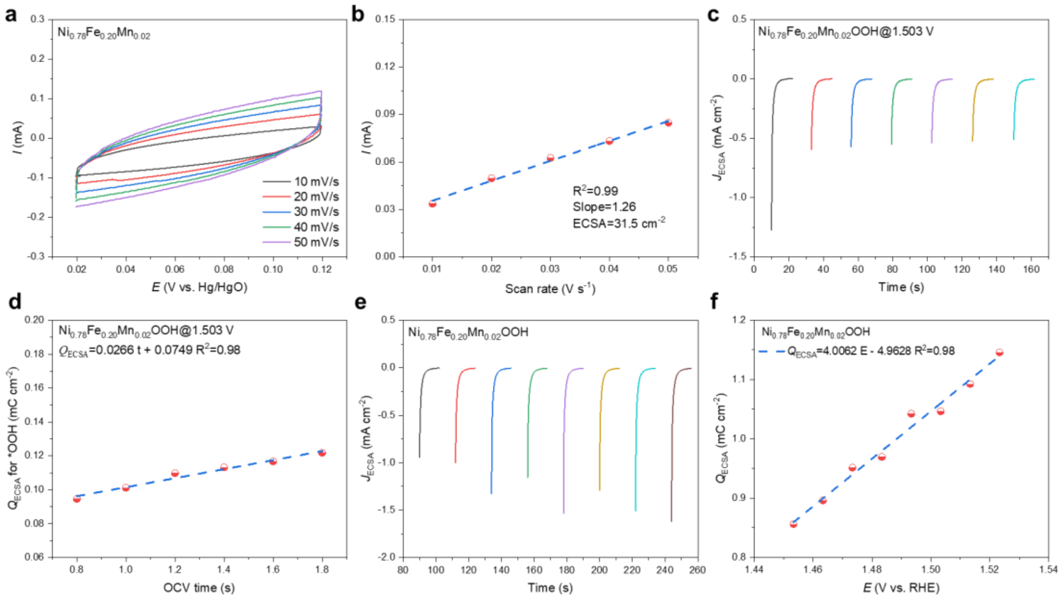

Supplementary Fig. 39. OCV–PV and PV measurements on $Ni_{0.78}Fe_{0.20}Mn_{0.02}OOH$. (a) The Cyclic voltammograms curves of the $Ni_{0.78}Fe_{0.20}Mn_{0.02}OOH$ and corresponding current (*I*) vs. scan rate plots (b). Plots of current vs. scan rate data were obtained from CV curves at 0.07 V vs. Hg/HgO. (c) The reductive pulse with the current response in OCV-PV test for $Ni_{0.78}Fe_{0.20}Mn_{0.02}OOH$ under charging bias of 1.503 V. (d) *OOH formation-related charge versus OCV duration. (e) The reductive pulse with the current response in PV test for $Ni_{0.78}Fe_{0.20}Mn_{0.02}OOH$. (f) *OH deprotonation-related charge versus charging bias. Source data are provided as a Source Data file.

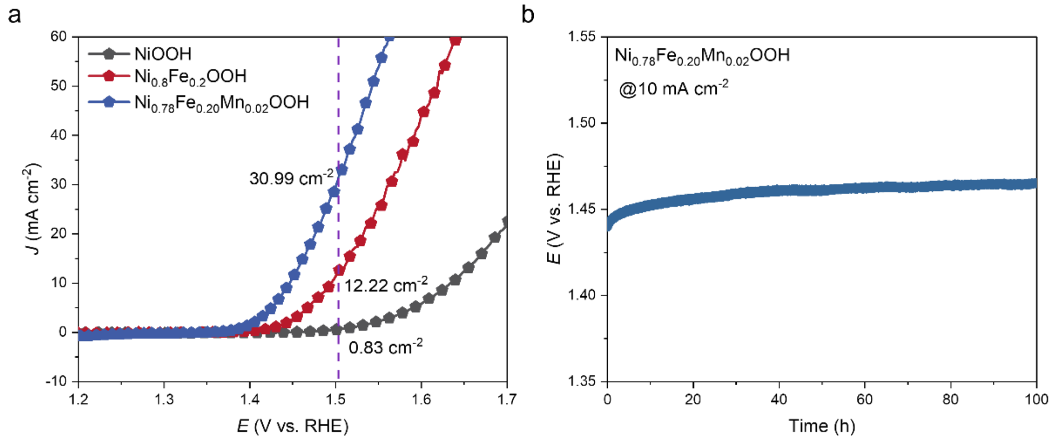

Supplementary Fig. 40. OER activity evaluation. (a) LSV curves of $Ni_{0.78}Fe_{0.20}Mn_{0.02}OOH$, $Ni_{0.78}Fe_{0.20}OOH$, and NiOOH without iR compensation. (b) Stability test of $Ni_{0.78}Fe_{0.20}Mn_{0.02}OOH$ at 10 mA cm^{-2} for 100 h. Source data are provided as a Source Data file.

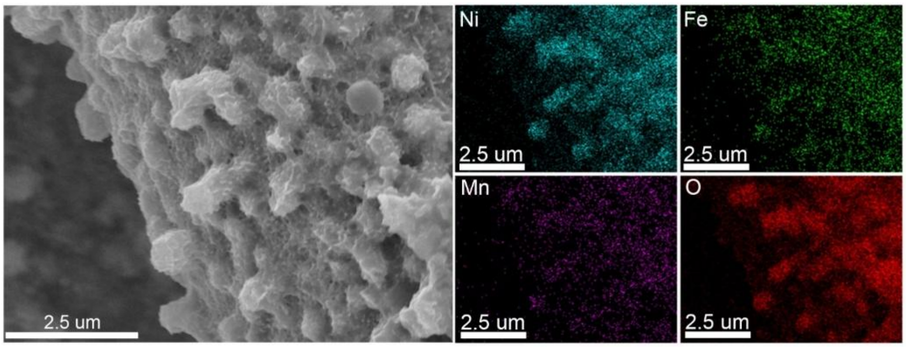

Supplementary Fig. 41. SEM-EDS images of $Ni_{0.78}Fe_{0.20}Mn_{0.02}$ sample before stability test.

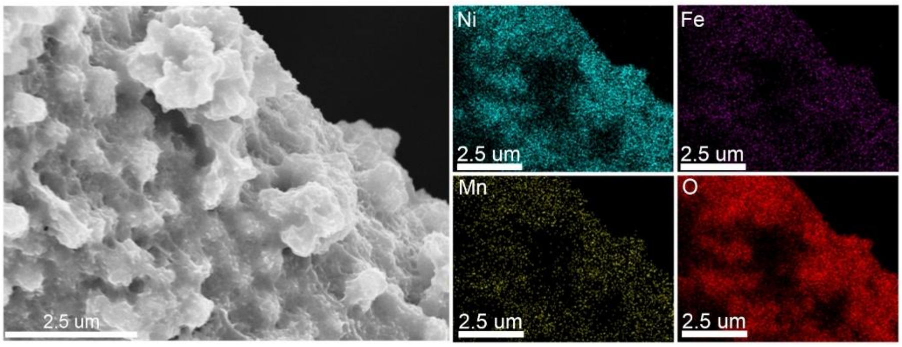

Supplementary Fig. 42. SEM-EDS images of $Ni_{0.78}Fe_{0.20}Mn_{0.02}$ sample after stability test.

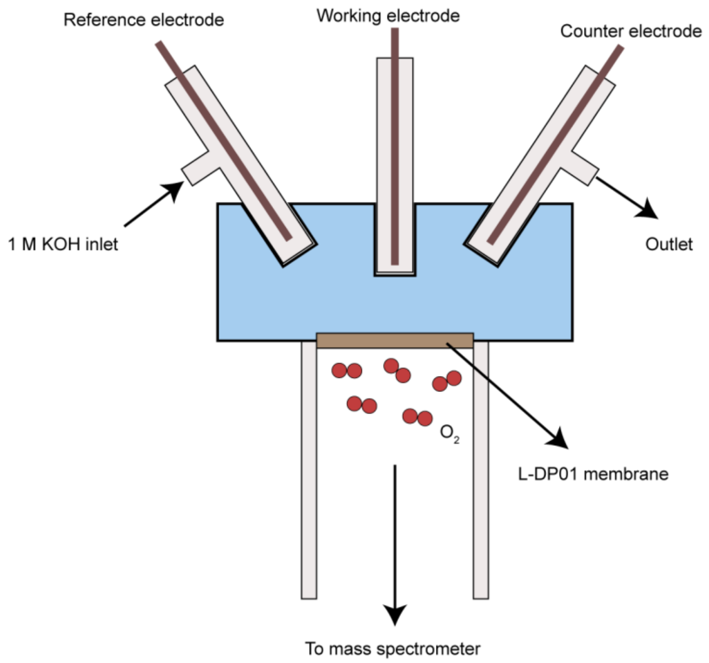

Supplementary Fig. 43. Schematic of DEMS electrochemical cell. The L-DP01 membrane from Shanghai Linglu is employed. The Ag/AgCl electrode was chosen as the reference electrode. The Pt wire was used as the counter electrode. The electrolyte is 1 M KOH.

Supplementary Table 1. ICP-determined elemental contents of $Ni_{0.78}Fe_{0.20}Mn_{0.02}$

Element	Ni<br>wt%	Fe<br>wt%	Mn<br>wt%
Pristine	78.8	19.3	1.9
After OER	78.6	19.4	2.0

Supplementary References:

1 Hao, Y. *et al.* Recognition of surface oxygen intermediates on NiFe oxyhydroxide oxygen-evolving catalysts by homogeneous oxidation reactivity. *Journal of the American Chemical Society* **143**, 1493-1502 (2021).
