# Supplementary material for: Decoupling electron transfer defines a quantitative kinetic framework for oxygen evolution catalysis
Source: Nat Commun. 2026 Jun 10;17:7377. doi: 10.1038/s41467-026-74392-3 (PMC13402610; doi:10.1038/s41467-026-74392-3)
Supplement: Supplementary file 2 — Description of Additional Supplementary Files [file 41467_2026_74392_MOESM2_ESM.pdf]

Description of Additional Supplementary Files

File Name: Supplementary Data 1

Description: CONTCAR of the optimized NiOOH models with different *O coverage or Fe dopant concentration.
