# Supplementary material for: Decoupling electron transfer defines a quantitative kinetic framework for oxygen evolution catalysis
Source: Nat Commun. 2026 Jun 10;17:7377. doi: 10.1038/s41467-026-74392-3 (PMC13402610; doi:10.1038/s41467-026-74392-3)
Supplement: Supplementary file 3 — Supplementary Data 1 [file 41467_2026_74392_MOESM3_ESM.docx]

**Appendix: All models: Optimized POSCARs (CONTCARs)**

NiOOH_17H ML CONTCAR

This file is generated by VASPKIT code

1.00000000000000

17.5560007098000028 0.0000000000000000 0.0000000000000000

-8.7889406573999977 15.4086551811000021 0.0000000000000000

0.0000000000000015 4.9977513337000028 24.4953563273999961

Ni O H

72 144 71

Selective dynamics

Direct

0.0341653786323661 0.3260019713917757 0.1960457408563343 F F F

0.0341653786323661 0.6593353047251114 0.1960457408563343 F F F

0.0341653786323661 0.9926686380584400 0.1960457408563343 F F F

0.2008320452990304 0.3260019713917757 0.1960457408563343 F F F

0.2008320452990304 0.6593353047251114 0.1960457408563343 F F F

0.2008320452990304 0.9926686380584400 0.1960457408563343 F F F

0.3674987119657018 0.3260019713917757 0.1960457408563343 F F F

0.3674987119657018 0.6593353047251114 0.1960457408563343 F F F

0.3674987119657018 0.9926686380584400 0.1960457408563343 F F F

0.5341653786323661 0.3260019713917757 0.1960457408563343 F F F

0.5341653786323661 0.6593353047251114 0.1960457408563343 F F F

0.5341653786323661 0.9926686380584400 0.1960457408563343 F F F

0.7008320452990304 0.3260019713917757 0.1960457408563343 F F F

0.7008320452990304 0.6593353047251114 0.1960457408563343 F F F

0.7008320452990304 0.9926686380584400 0.1960457408563343 F F F

0.8674987119657018 0.3260019713917757 0.1960457408563343 F F F

0.8674987119657018 0.6593353047251114 0.1960457408563343 F F F

0.8674987119657018 0.9926686380584400 0.1960457408563343 F F F

0.0351970056773183 0.1617665099525851 0.1949035160852901 F F F

0.0351970056773183 0.4950998432859137 0.1949035160852901 F F F

0.0351970056773183 0.8284331766192494 0.1949035160852901 F F F

0.2018636723439897 0.1617665099525851 0.1949035160852901 F F F

0.2018636723439897 0.4950998432859137 0.1949035160852901 F F F

0.2018636723439897 0.8284331766192494 0.1949035160852901 F F F

0.3685303390106540 0.1617665099525851 0.1949035160852901 F F F

0.3685303390106540 0.4950998432859137 0.1949035160852901 F F F

0.3685303390106540 0.8284331766192494 0.1949035160852901 F F F

0.5351970056773183 0.1617665099525851 0.1949035160852901 F F F

0.5351970056773183 0.4950998432859137 0.1949035160852901 F F F

0.5351970056773183 0.8284331766192494 0.1949035160852901 F F F

0.7018636723439897 0.1617665099525851 0.1949035160852901 F F F

0.7018636723439897 0.4950998432859137 0.1949035160852901 F F F

0.7018636723439897 0.8284331766192494 0.1949035160852901 F F F

0.8685303390106540 0.1617665099525851 0.1949035160852901 F F F

0.8685303390106540 0.4950998432859137 0.1949035160852901 F F F

0.8685303390106540 0.8284331766192494 0.1949035160852901 F F F

0.1330352507361495 0.0078329112382903 0.3796819534319712 T T T

0.1333950827604633 0.3415705328641743 0.3796252162413232 T T T

0.1338433545741490 0.6745118808426761 0.3797522605299911 T T T

0.2998855693278698 0.0079646773391363 0.3796482632723180 T T T

0.3000219522579123 0.3417760644710159 0.3794545655698756 T T T

0.3009614410559869 0.6744743062754213 0.3797280400047054 T T T

0.4666094616016245 0.0080772909411286 0.3796158925995157 T T T

0.4662529545303004 0.3419849221798028 0.3796866280620300 T T T

0.4689096019386161 0.6748362193958862 0.3771880686836258 T T T

0.6333383760034225 0.0085004255732639 0.3795973422785267 T T T

0.6328954026619715 0.3416941068431363 0.3797465155130192 T T T

0.6299035338543656 0.6743490887269816 0.3787316801856026 T T T

0.7998904418133914 0.0083998911044397 0.3795351823432732 T T T

0.7997323240908324 0.3416630521412011 0.3797176492598963 T T T

0.7986479072944120 0.6742613422885390 0.3803003242750372 T T T

0.9663046180014493 0.0078777490957783 0.3796320011867136 T T T

0.9665668727437069 0.3415747542324344 0.3797391541167783 T T T

0.9663831980661683 0.6746008274122286 0.3798161592068892 T T T

0.1320400008320601 0.1720318531178057 0.3806339640006341 T T T

0.1321619506359084 0.5053248117543154 0.3808769385668358 T T T

0.1321806711712736 0.8385062605128517 0.3806169031509853 T T T

0.2986542241334018 0.1720564684163350 0.3807608793844927 T T T

0.2993387421301079 0.5056942854633280 0.3796859231713254 T T T

0.2993899088701902 0.8386668038578061 0.3805525296192025 T T T

0.4653144879501849 0.1722177941743903 0.3808047585173366 T T T

0.4660441885972647 0.5078267154132564 0.3798264439114073 T T T

0.4667164800977738 0.8390320772835876 0.3805607023513948 T T T

0.6320365407605809 0.1723619795418629 0.3807586171464770 T T T

0.6314254171638713 0.5060322133919335 0.3806638259478195 T T T

0.6325581789561424 0.8396679825023393 0.3801276868272024 T T T

0.7988162558173654 0.1724038996353680 0.3806584704365306 T T T

0.7982263200605413 0.5056360150501945 0.3808128407430842 T T T

0.7979158448249946 0.8385394990360117 0.3805444119314789 T T T

0.9654883711121994 0.1722411967996376 0.3806047461406927 T T T

0.9652378527791544 0.5055236981097848 0.3808716094650522 T T T

0.9649031311391744 0.8383143291455406 0.3807572290260183 T T T

0.0826462090432543 0.0913673776135795 0.2306510691320725 F F F

0.0826462090432543 0.4247007109469081 0.2306510691320725 F F F

0.0826462090432543 0.7580340442802367 0.2306510691320725 F F F

0.2493128757099186 0.0913673776135795 0.2306510691320725 F F F

0.2493128757099186 0.4247007109469081 0.2306510691320725 F F F

0.2493128757099186 0.7580340442802367 0.2306510691320725 F F F

0.4159795423765829 0.0913673776135795 0.2306510691320725 F F F

0.4159795423765829 0.4247007109469081 0.2306510691320725 F F F

0.4159795423765829 0.7580340442802367 0.2306510691320725 F F F

0.5826462090432472 0.0913673776135795 0.2306510691320725 F F F

0.5826462090432472 0.4247007109469081 0.2306510691320725 F F F

0.5826462090432472 0.7580340442802367 0.2306510691320725 F F F

0.7493128757099186 0.0913673776135795 0.2306510691320725 F F F

0.7493128757099186 0.4247007109469081 0.2306510691320725 F F F

0.7493128757099186 0.7580340442802367 0.2306510691320725 F F F

0.9159795423765758 0.0913673776135795 0.2306510691320725 F F F

0.9159795423765758 0.4247007109469081 0.2306510691320725 F F F

0.9159795423765758 0.7580340442802367 0.2306510691320725 F F F

0.0902677041989364 0.2625098265214945 0.2403988745878252 F F F

0.0902677041989364 0.5958431598548302 0.2403988745878252 F F F

0.0902677041989364 0.9291764931881659 0.2403988745878252 F F F

0.2569343708656078 0.2625098265214945 0.2403988745878252 F F F

0.2569343708656078 0.5958431598548302 0.2403988745878252 F F F

0.2569343708656078 0.9291764931881659 0.2403988745878252 F F F

0.4236010375322721 0.2625098265214945 0.2403988745878252 F F F

0.4236010375322721 0.5958431598548302 0.2403988745878252 F F F

0.4236010375322721 0.9291764931881659 0.2403988745878252 F F F

0.5902677041989364 0.2625098265214945 0.2403988745878252 F F F

0.5902677041989364 0.5958431598548302 0.2403988745878252 F F F

0.5902677041989364 0.9291764931881659 0.2403988745878252 F F F

0.7569343708656078 0.2625098265214945 0.2403988745878252 F F F

0.7569343708656078 0.5958431598548302 0.2403988745878252 F F F

0.7569343708656078 0.9291764931881659 0.2403988745878252 F F F

0.9236010375322721 0.2625098265214945 0.2403988745878252 F F F

0.9236010375322721 0.5958431598548302 0.2403988745878252 F F F

0.9236010375322721 0.9291764931881659 0.2403988745878252 F F F

0.1450268573747806 0.0574030619262587 0.1502611028726761 F F F

0.1450268573747806 0.3907363952595873 0.1502611028726761 F F F

0.1450268573747806 0.7240697285929230 0.1502611028726761 F F F

0.3116935240414520 0.0574030619262587 0.1502611028726761 F F F

0.3116935240414520 0.3907363952595873 0.1502611028726761 F F F

0.3116935240414520 0.7240697285929230 0.1502611028726761 F F F

0.4783601907081163 0.0574030619262587 0.1502611028726761 F F F

0.4783601907081163 0.3907363952595873 0.1502611028726761 F F F

0.4783601907081163 0.7240697285929230 0.1502611028726761 F F F

0.6450268573747806 0.0574030619262587 0.1502611028726761 F F F

0.6450268573747806 0.3907363952595873 0.1502611028726761 F F F

0.6450268573747806 0.7240697285929230 0.1502611028726761 F F F

0.8116935240414520 0.0574030619262587 0.1502611028726761 F F F

0.8116935240414520 0.3907363952595873 0.1502611028726761 F F F

0.8116935240414520 0.7240697285929230 0.1502611028726761 F F F

0.9783601907081163 0.0574030619262587 0.1502611028726761 F F F

0.9783601907081163 0.3907363952595873 0.1502611028726761 F F F

0.9783601907081163 0.7240697285929230 0.1502611028726761 F F F

0.1540633143111236 0.2313171148045612 0.1594044809199673 F F F

0.1540633143111236 0.5646504481378969 0.1594044809199673 F F F

0.1540633143111236 0.8979837814712326 0.1594044809199673 F F F

0.3207299809777879 0.2313171148045612 0.1594044809199673 F F F

0.3207299809777879 0.5646504481378969 0.1594044809199673 F F F

0.3207299809777879 0.8979837814712326 0.1594044809199673 F F F

0.4873966476444522 0.2313171148045612 0.1594044809199673 F F F

0.4873966476444522 0.5646504481378969 0.1594044809199673 F F F

0.4873966476444522 0.8979837814712326 0.1594044809199673 F F F

0.6540633143111236 0.2313171148045612 0.1594044809199673 F F F

0.6540633143111236 0.5646504481378969 0.1594044809199673 F F F

0.6540633143111236 0.8979837814712326 0.1594044809199673 F F F

0.8207299809777879 0.2313171148045612 0.1594044809199673 F F F

0.8207299809777879 0.5646504481378969 0.1594044809199673 F F F

0.8207299809777879 0.8979837814712326 0.1594044809199673 F F F

0.9873966476444522 0.2313171148045612 0.1594044809199673 F F F

0.9873966476444522 0.5646504481378969 0.1594044809199673 F F F

0.9873966476444522 0.8979837814712326 0.1594044809199673 F F F

0.0131240716232894 0.1027164643107531 0.4156650754827880 T T T

0.0132517456216511 0.4360835871869111 0.4159353117611366 T T T

0.0133714829537822 0.7691583985021785 0.4157439020215759 T T T

0.1797180775292840 0.1025719587871941 0.4157398157155769 T T T

0.1806369059135023 0.4363735558963990 0.4156831310378667 T T T

0.1807169223749673 0.7692313224712304 0.4155153882116704 T T T

0.3463824225650327 0.1025885243942058 0.4157884437498474 T T T

0.3472878927382034 0.4376076065929378 0.4148485835175410 T T T

0.3478179062891653 0.7690998863752815 0.4152336268028508 T T T

0.5130589691163535 0.1026746464762772 0.4157672461215058 T T T

0.5126538792447340 0.4374093230579403 0.4154355865459136 T T T

0.5139841885861023 0.7683502739091703 0.4146076607514469 T T T

0.6798471378891318 0.1029690173246291 0.4157505204289382 T T T

0.6794154928664625 0.4363792287948682 0.4157908571448060 T T T

0.6787891309065452 0.7706496167064079 0.4147551000699699 T T T

0.8466224269711419 0.1029827869347162 0.4156695892537875 T T T

0.8463251547128956 0.4362283236813595 0.4158424520795129 T T T

0.8459173721607924 0.7689119498364652 0.4159901273475692 T T T

0.0226764494145514 0.2770149982748622 0.4247795367537361 T T T

0.0222621819496685 0.6101070763427958 0.4248248872025197 T T T

0.0222699324187703 0.9432633724595043 0.4247890071322032 T T T

0.1895266989125036 0.2773657123314113 0.4246592856029135 T T T

0.1900570462068275 0.6102306517974231 0.4251152263027290 T T T

0.1891720032283351 0.9432229489189378 0.4247480926853584 T T T

0.3557113424329479 0.2771592184270633 0.4249040533047036 T T T

0.3626045273826245 0.6125054095142306 0.4227622089738415 T T T

0.3559338574405845 0.9431919832306638 0.4247081771835369 T T T

0.5221670801997155 0.2771164265191830 0.4249283442605979 T T T

0.5189382410045188 0.6186907876098209 0.4184295665735960 T T T

0.5222346919932960 0.9428893899135573 0.4247270207152993 T T T

0.6889703053986129 0.2770391416859610 0.4248372597050516 T T T

0.6853701364010534 0.6092724766608850 0.4245410651714253 T T T

0.6889644453901141 0.9437279308967270 0.4244933842277218 T T T

0.8557675655538632 0.2769721210685118 0.4248032569692866 T T T

0.8547001181340094 0.6100845870080407 0.4248595944750064 T T T

0.8554334078717839 0.9434872340876124 0.4245950910479756 T T T

0.0767356865841538 0.0708740819645826 0.3362080251146415 T T T

0.0770027870878419 0.4047304325613313 0.3362594370697677 T T T

0.0776070276564268 0.7372707274806982 0.3363381904823685 T T T

0.2434025104886341 0.0708540328386447 0.3362387381655819 T T T

0.2433501269184688 0.4047088391237195 0.3358102922596380 T T T

0.2454984708517201 0.7369255385609441 0.3362252453916348 T T T

0.4101470114905477 0.0710744521345807 0.3362113756893167 T T T

0.4097105728608969 0.4041250103983927 0.3360006785319384 T T T

0.4143110700155460 0.7348511108872450 0.3364561742313449 T T T

0.5768035585113087 0.0712771323766393 0.3362218525879750 T T T

0.5765143915104197 0.4044629706334487 0.3362805478996870 T T T

0.5743557999966470 0.7396023677236010 0.3335047011138725 T T T

0.7437494350683648 0.0716942608283045 0.3361082252154816 T T T

0.7433847180131276 0.4046911795569064 0.3362881773677946 T T T

0.7420629279543576 0.7364633520900020 0.3367940973641304 T T T

0.9102871123815819 0.0712978253517277 0.3361055688219452 T T T

0.9102111403870102 0.4046564897381058 0.3362963686474583 T T T

0.9101081449998687 0.7374091335093470 0.3365474797880953 T T T

0.0848070967223383 0.2426193216520099 0.3457246263125569 T T T

0.0849261751807425 0.5757446980341825 0.3460150664160905 T T T

0.0845830085632645 0.9088051209909570 0.3458046848978067 T T T

0.2512936017037569 0.2423348543672920 0.3457645041354029 T T T

0.2517183888796259 0.5759814169063526 0.3459512397622762 T T T

0.2516588233356415 0.9089060663993194 0.3457153225763904 T T T

0.4179292366301131 0.2425442481914026 0.3459100714791413 T T T

0.4179310238947073 0.5768313075148509 0.3433401328041580 T T T

0.4184893919378985 0.9089952575672906 0.3456008452201472 T T T

0.5845649586376913 0.2426433266709740 0.3458594601869711 T T T

0.5835075670928508 0.5752551514057038 0.3448268355519729 T T T

0.5852424314824841 0.9099945099750464 0.3453923046415757 T T T

0.7513498259028407 0.2427607516029637 0.3457764226369710 T T T

0.7507885379606531 0.5760311216958988 0.3459107331849612 T T T

0.7513269124386874 0.9097351492001643 0.3453478238011552 T T T

0.9181713042162578 0.2427669946278715 0.3457354386625813 T T T

0.9178066600110949 0.5758778777863246 0.3459142175509091 T T T

0.9177766532073988 0.9088037019451219 0.3457835645978996 T T T

0.1397131416109474 0.0545266729236502 0.1118484288951365 F F F

0.1397131416109474 0.3878600062569788 0.1118484288951365 F F F

0.1397131416109474 0.7211933395903145 0.1118484288951365 F F F

0.3063798082776117 0.0545266729236502 0.1118484288951365 F F F

0.3063798082776117 0.3878600062569788 0.1118484288951365 F F F

0.3063798082776117 0.7211933395903145 0.1118484288951365 F F F

0.4730464749442831 0.0545266729236502 0.1118484288951365 F F F

0.4730464749442831 0.3878600062569788 0.1118484288951365 F F F

0.4730464749442831 0.7211933395903145 0.1118484288951365 F F F

0.6397131416109474 0.0545266729236502 0.1118484288951365 F F F

0.6397131416109474 0.3878600062569788 0.1118484288951365 F F F

0.6397131416109474 0.7211933395903145 0.1118484288951365 F F F

0.8063798082776117 0.0545266729236502 0.1118484288951365 F F F

0.8063798082776117 0.3878600062569788 0.1118484288951365 F F F

0.8063798082776117 0.7211933395903145 0.1118484288951365 F F F

0.9730464749442831 0.0545266729236502 0.1118484288951365 F F F

0.9730464749442831 0.3878600062569788 0.1118484288951365 F F F

0.9730464749442831 0.7211933395903145 0.1118484288951365 F F F

0.0904969084302252 0.2563302902655593 0.2814745639703204 F F F

0.0904969084302252 0.5896636235988879 0.2814745639703204 F F F

0.0904969084302252 0.9229969569322236 0.2814745639703204 F F F

0.2571635750968966 0.2563302902655593 0.2814745639703204 F F F

0.2571635750968966 0.5896636235988879 0.2814745639703204 F F F

0.2571635750968966 0.9229969569322236 0.2814745639703204 F F F

0.4238302417635609 0.2563302902655593 0.2814745639703204 F F F

0.4238302417635609 0.5896636235988879 0.2814745639703204 F F F

0.4238302417635609 0.9229969569322236 0.2814745639703204 F F F

0.5904969084302252 0.2563302902655593 0.2814745639703204 F F F

0.5904969084302252 0.5896636235988879 0.2814745639703204 F F F

0.5904969084302252 0.9229969569322236 0.2814745639703204 F F F

0.7571635750968895 0.2563302902655593 0.2814745639703204 F F F

0.7571635750968895 0.5896636235988879 0.2814745639703204 F F F

0.7571635750968895 0.9229969569322236 0.2814745639703204 F F F

0.9238302417635609 0.2563302902655593 0.2814745639703204 F F F

0.9238302417635609 0.5896636235988879 0.2814745639703204 F F F

0.9238302417635609 0.9229969569322236 0.2814745639703204 F F F

0.0764745799413895 0.0773464503994843 0.2949901967763967 T T T

0.0766861181283777 0.4111484718852681 0.2950368613525969 T T T

0.0772966034557990 0.7438733971218103 0.2951306508606544 T T T

0.2430976283623466 0.0773352101451747 0.2950221222424706 T T T

0.2429849286558958 0.4110760563430547 0.2945360102501243 T T T

0.2450165832226832 0.7434450029298909 0.2949602527710716 T T T

0.4098076802744882 0.0775477683525487 0.2949860957236976 T T T

0.4093361247082432 0.4102141541696417 0.2947840715784230 T T T

0.4147686761239620 0.7408683760072554 0.2948119836615960 T T T

0.5764810839964373 0.0775958009100775 0.2949866039589551 T T T

0.5762202199254042 0.4107730450008207 0.2950912802065666 T T T

0.5726822903674228 0.7443410926544937 0.2916639155736206 T T T

0.7434311524561021 0.0779771869691855 0.2948890170111361 T T T

0.7431266790585227 0.4110547052943868 0.2950907939184386 T T T

0.7427977576973817 0.7429455128911980 0.2956175567534072 T T T

0.9099829850942430 0.0777143082079586 0.2948830581392692 T T T

0.9098974611519537 0.4110409044398426 0.2950875683825460 T T T

0.9101090944185964 0.7439757665224874 0.2953943934034265 T T T

0.0281812098108929 0.2791421201836116 0.4632469061527595 T T T

0.0268845945878891 0.6120986291097839 0.4633484454117283 T T T

0.0277573836885430 0.9457625051963781 0.4632140966410732 T T T

0.1953888827490968 0.2803188807049465 0.4630135546699971 T T T

0.1952310364994445 0.6132454056378654 0.4635004774002532 T T T

0.1947441721267316 0.9456130688402673 0.4631835293514686 T T T

0.3615639569529431 0.2801407400801259 0.4632554102427363 T T T

0.3763871490608069 0.6137625809575726 0.4604860397897355 T T T

0.3616515355337979 0.9454364695144284 0.4631510622560120 T T T

0.5279014323029751 0.2794575754954940 0.4633565830625626 T T T

0.5275364490445408 0.9440566853965867 0.4633062221498909 T T T

0.6945075234878896 0.2792645208592277 0.4632931112887861 T T T

0.6853059293762801 0.6086437285902385 0.4634926909167703 T T T

0.6940245491863586 0.9442536135596901 0.4631521578131098 T T T

0.8612085854954710 0.2790302998986600 0.4632807830442536 T T T

0.8588393495791201 0.6120619299110431 0.4634056796024468 T T T

0.8607228354848012 0.9454736919345732 0.4630903826027137 T T T

0.00000000E+00 0.00000000E+00 0.00000000E+00

0.00000000E+00 0.00000000E+00 0.00000000E+00

0.00000000E+00 0.00000000E+00 0.00000000E+00

0.00000000E+00 0.00000000E+00 0.00000000E+00

0.00000000E+00 0.00000000E+00 0.00000000E+00

0.00000000E+00 0.00000000E+00 0.00000000E+00

0.00000000E+00 0.00000000E+00 0.00000000E+00

0.00000000E+00 0.00000000E+00 0.00000000E+00

0.00000000E+00 0.00000000E+00 0.00000000E+00

0.00000000E+00 0.00000000E+00 0.00000000E+00

0.00000000E+00 0.00000000E+00 0.00000000E+00

0.00000000E+00 0.00000000E+00 0.00000000E+00

0.00000000E+00 0.00000000E+00 0.00000000E+00

0.00000000E+00 0.00000000E+00 0.00000000E+00

0.00000000E+00 0.00000000E+00 0.00000000E+00

0.00000000E+00 0.00000000E+00 0.00000000E+00

0.00000000E+00 0.00000000E+00 0.00000000E+00

0.00000000E+00 0.00000000E+00 0.00000000E+00

0.00000000E+00 0.00000000E+00 0.00000000E+00

0.00000000E+00 0.00000000E+00 0.00000000E+00

0.00000000E+00 0.00000000E+00 0.00000000E+00

0.00000000E+00 0.00000000E+00 0.00000000E+00

0.00000000E+00 0.00000000E+00 0.00000000E+00

0.00000000E+00 0.00000000E+00 0.00000000E+00

0.00000000E+00 0.00000000E+00 0.00000000E+00

0.00000000E+00 0.00000000E+00 0.00000000E+00

0.00000000E+00 0.00000000E+00 0.00000000E+00

0.00000000E+00 0.00000000E+00 0.00000000E+00

0.00000000E+00 0.00000000E+00 0.00000000E+00

0.00000000E+00 0.00000000E+00 0.00000000E+00

0.00000000E+00 0.00000000E+00 0.00000000E+00

0.00000000E+00 0.00000000E+00 0.00000000E+00

0.00000000E+00 0.00000000E+00 0.00000000E+00

0.00000000E+00 0.00000000E+00 0.00000000E+00

0.00000000E+00 0.00000000E+00 0.00000000E+00

0.00000000E+00 0.00000000E+00 0.00000000E+00

0.00000000E+00 0.00000000E+00 0.00000000E+00

0.00000000E+00 0.00000000E+00 0.00000000E+00

0.00000000E+00 0.00000000E+00 0.00000000E+00

0.00000000E+00 0.00000000E+00 0.00000000E+00

0.00000000E+00 0.00000000E+00 0.00000000E+00

0.00000000E+00 0.00000000E+00 0.00000000E+00

0.00000000E+00 0.00000000E+00 0.00000000E+00

0.00000000E+00 0.00000000E+00 0.00000000E+00

0.00000000E+00 0.00000000E+00 0.00000000E+00

0.00000000E+00 0.00000000E+00 0.00000000E+00

0.00000000E+00 0.00000000E+00 0.00000000E+00

0.00000000E+00 0.00000000E+00 0.00000000E+00

0.00000000E+00 0.00000000E+00 0.00000000E+00

0.00000000E+00 0.00000000E+00 0.00000000E+00

0.00000000E+00 0.00000000E+00 0.00000000E+00

0.00000000E+00 0.00000000E+00 0.00000000E+00

0.00000000E+00 0.00000000E+00 0.00000000E+00

0.00000000E+00 0.00000000E+00 0.00000000E+00

0.00000000E+00 0.00000000E+00 0.00000000E+00

0.00000000E+00 0.00000000E+00 0.00000000E+00

0.00000000E+00 0.00000000E+00 0.00000000E+00

0.00000000E+00 0.00000000E+00 0.00000000E+00

0.00000000E+00 0.00000000E+00 0.00000000E+00

0.00000000E+00 0.00000000E+00 0.00000000E+00

0.00000000E+00 0.00000000E+00 0.00000000E+00

0.00000000E+00 0.00000000E+00 0.00000000E+00

0.00000000E+00 0.00000000E+00 0.00000000E+00

0.00000000E+00 0.00000000E+00 0.00000000E+00

0.00000000E+00 0.00000000E+00 0.00000000E+00

0.00000000E+00 0.00000000E+00 0.00000000E+00

0.00000000E+00 0.00000000E+00 0.00000000E+00

0.00000000E+00 0.00000000E+00 0.00000000E+00

0.00000000E+00 0.00000000E+00 0.00000000E+00

0.00000000E+00 0.00000000E+00 0.00000000E+00

0.00000000E+00 0.00000000E+00 0.00000000E+00

0.00000000E+00 0.00000000E+00 0.00000000E+00

0.00000000E+00 0.00000000E+00 0.00000000E+00

0.00000000E+00 0.00000000E+00 0.00000000E+00

0.00000000E+00 0.00000000E+00 0.00000000E+00

0.00000000E+00 0.00000000E+00 0.00000000E+00

0.00000000E+00 0.00000000E+00 0.00000000E+00

0.00000000E+00 0.00000000E+00 0.00000000E+00

0.00000000E+00 0.00000000E+00 0.00000000E+00

0.00000000E+00 0.00000000E+00 0.00000000E+00

0.00000000E+00 0.00000000E+00 0.00000000E+00

0.00000000E+00 0.00000000E+00 0.00000000E+00

0.00000000E+00 0.00000000E+00 0.00000000E+00

0.00000000E+00 0.00000000E+00 0.00000000E+00

0.00000000E+00 0.00000000E+00 0.00000000E+00

0.00000000E+00 0.00000000E+00 0.00000000E+00

0.00000000E+00 0.00000000E+00 0.00000000E+00

0.00000000E+00 0.00000000E+00 0.00000000E+00

0.00000000E+00 0.00000000E+00 0.00000000E+00

0.00000000E+00 0.00000000E+00 0.00000000E+00

0.00000000E+00 0.00000000E+00 0.00000000E+00

0.00000000E+00 0.00000000E+00 0.00000000E+00

0.00000000E+00 0.00000000E+00 0.00000000E+00

0.00000000E+00 0.00000000E+00 0.00000000E+00

0.00000000E+00 0.00000000E+00 0.00000000E+00

0.00000000E+00 0.00000000E+00 0.00000000E+00

0.00000000E+00 0.00000000E+00 0.00000000E+00

0.00000000E+00 0.00000000E+00 0.00000000E+00

0.00000000E+00 0.00000000E+00 0.00000000E+00

0.00000000E+00 0.00000000E+00 0.00000000E+00

0.00000000E+00 0.00000000E+00 0.00000000E+00

0.00000000E+00 0.00000000E+00 0.00000000E+00

0.00000000E+00 0.00000000E+00 0.00000000E+00

0.00000000E+00 0.00000000E+00 0.00000000E+00

0.00000000E+00 0.00000000E+00 0.00000000E+00

0.00000000E+00 0.00000000E+00 0.00000000E+00

0.00000000E+00 0.00000000E+00 0.00000000E+00

0.00000000E+00 0.00000000E+00 0.00000000E+00

0.00000000E+00 0.00000000E+00 0.00000000E+00

0.00000000E+00 0.00000000E+00 0.00000000E+00

0.00000000E+00 0.00000000E+00 0.00000000E+00

0.00000000E+00 0.00000000E+00 0.00000000E+00

0.00000000E+00 0.00000000E+00 0.00000000E+00

0.00000000E+00 0.00000000E+00 0.00000000E+00

0.00000000E+00 0.00000000E+00 0.00000000E+00

0.00000000E+00 0.00000000E+00 0.00000000E+00

0.00000000E+00 0.00000000E+00 0.00000000E+00

0.00000000E+00 0.00000000E+00 0.00000000E+00

0.00000000E+00 0.00000000E+00 0.00000000E+00

0.00000000E+00 0.00000000E+00 0.00000000E+00

0.00000000E+00 0.00000000E+00 0.00000000E+00

0.00000000E+00 0.00000000E+00 0.00000000E+00

0.00000000E+00 0.00000000E+00 0.00000000E+00

0.00000000E+00 0.00000000E+00 0.00000000E+00

0.00000000E+00 0.00000000E+00 0.00000000E+00

0.00000000E+00 0.00000000E+00 0.00000000E+00

0.00000000E+00 0.00000000E+00 0.00000000E+00

0.00000000E+00 0.00000000E+00 0.00000000E+00

0.00000000E+00 0.00000000E+00 0.00000000E+00

0.00000000E+00 0.00000000E+00 0.00000000E+00

0.00000000E+00 0.00000000E+00 0.00000000E+00

0.00000000E+00 0.00000000E+00 0.00000000E+00

0.00000000E+00 0.00000000E+00 0.00000000E+00

0.00000000E+00 0.00000000E+00 0.00000000E+00

0.00000000E+00 0.00000000E+00 0.00000000E+00

0.00000000E+00 0.00000000E+00 0.00000000E+00

0.00000000E+00 0.00000000E+00 0.00000000E+00

0.00000000E+00 0.00000000E+00 0.00000000E+00

0.00000000E+00 0.00000000E+00 0.00000000E+00

0.00000000E+00 0.00000000E+00 0.00000000E+00

0.00000000E+00 0.00000000E+00 0.00000000E+00

0.00000000E+00 0.00000000E+00 0.00000000E+00

0.00000000E+00 0.00000000E+00 0.00000000E+00

0.00000000E+00 0.00000000E+00 0.00000000E+00

0.00000000E+00 0.00000000E+00 0.00000000E+00

0.00000000E+00 0.00000000E+00 0.00000000E+00

0.00000000E+00 0.00000000E+00 0.00000000E+00

0.00000000E+00 0.00000000E+00 0.00000000E+00

0.00000000E+00 0.00000000E+00 0.00000000E+00

0.00000000E+00 0.00000000E+00 0.00000000E+00

0.00000000E+00 0.00000000E+00 0.00000000E+00

0.00000000E+00 0.00000000E+00 0.00000000E+00

0.00000000E+00 0.00000000E+00 0.00000000E+00

0.00000000E+00 0.00000000E+00 0.00000000E+00

0.00000000E+00 0.00000000E+00 0.00000000E+00

0.00000000E+00 0.00000000E+00 0.00000000E+00

0.00000000E+00 0.00000000E+00 0.00000000E+00

0.00000000E+00 0.00000000E+00 0.00000000E+00

0.00000000E+00 0.00000000E+00 0.00000000E+00

0.00000000E+00 0.00000000E+00 0.00000000E+00

0.00000000E+00 0.00000000E+00 0.00000000E+00

0.00000000E+00 0.00000000E+00 0.00000000E+00

0.00000000E+00 0.00000000E+00 0.00000000E+00

0.00000000E+00 0.00000000E+00 0.00000000E+00

0.00000000E+00 0.00000000E+00 0.00000000E+00

0.00000000E+00 0.00000000E+00 0.00000000E+00

0.00000000E+00 0.00000000E+00 0.00000000E+00

0.00000000E+00 0.00000000E+00 0.00000000E+00

0.00000000E+00 0.00000000E+00 0.00000000E+00

0.00000000E+00 0.00000000E+00 0.00000000E+00

0.00000000E+00 0.00000000E+00 0.00000000E+00

0.00000000E+00 0.00000000E+00 0.00000000E+00

0.00000000E+00 0.00000000E+00 0.00000000E+00

0.00000000E+00 0.00000000E+00 0.00000000E+00

0.00000000E+00 0.00000000E+00 0.00000000E+00

0.00000000E+00 0.00000000E+00 0.00000000E+00

0.00000000E+00 0.00000000E+00 0.00000000E+00

0.00000000E+00 0.00000000E+00 0.00000000E+00

0.00000000E+00 0.00000000E+00 0.00000000E+00

0.00000000E+00 0.00000000E+00 0.00000000E+00

0.00000000E+00 0.00000000E+00 0.00000000E+00

0.00000000E+00 0.00000000E+00 0.00000000E+00

0.00000000E+00 0.00000000E+00 0.00000000E+00

0.00000000E+00 0.00000000E+00 0.00000000E+00

0.00000000E+00 0.00000000E+00 0.00000000E+00

0.00000000E+00 0.00000000E+00 0.00000000E+00

0.00000000E+00 0.00000000E+00 0.00000000E+00

0.00000000E+00 0.00000000E+00 0.00000000E+00

0.00000000E+00 0.00000000E+00 0.00000000E+00

0.00000000E+00 0.00000000E+00 0.00000000E+00

0.00000000E+00 0.00000000E+00 0.00000000E+00

0.00000000E+00 0.00000000E+00 0.00000000E+00

0.00000000E+00 0.00000000E+00 0.00000000E+00

0.00000000E+00 0.00000000E+00 0.00000000E+00

0.00000000E+00 0.00000000E+00 0.00000000E+00

0.00000000E+00 0.00000000E+00 0.00000000E+00

0.00000000E+00 0.00000000E+00 0.00000000E+00

0.00000000E+00 0.00000000E+00 0.00000000E+00

0.00000000E+00 0.00000000E+00 0.00000000E+00

0.00000000E+00 0.00000000E+00 0.00000000E+00

0.00000000E+00 0.00000000E+00 0.00000000E+00

0.00000000E+00 0.00000000E+00 0.00000000E+00

0.00000000E+00 0.00000000E+00 0.00000000E+00

0.00000000E+00 0.00000000E+00 0.00000000E+00

0.00000000E+00 0.00000000E+00 0.00000000E+00

0.00000000E+00 0.00000000E+00 0.00000000E+00

0.00000000E+00 0.00000000E+00 0.00000000E+00

0.00000000E+00 0.00000000E+00 0.00000000E+00

0.00000000E+00 0.00000000E+00 0.00000000E+00

0.00000000E+00 0.00000000E+00 0.00000000E+00

0.00000000E+00 0.00000000E+00 0.00000000E+00

0.00000000E+00 0.00000000E+00 0.00000000E+00

0.00000000E+00 0.00000000E+00 0.00000000E+00

0.00000000E+00 0.00000000E+00 0.00000000E+00

0.00000000E+00 0.00000000E+00 0.00000000E+00

0.00000000E+00 0.00000000E+00 0.00000000E+00

0.00000000E+00 0.00000000E+00 0.00000000E+00

0.00000000E+00 0.00000000E+00 0.00000000E+00

0.00000000E+00 0.00000000E+00 0.00000000E+00

0.00000000E+00 0.00000000E+00 0.00000000E+00

0.00000000E+00 0.00000000E+00 0.00000000E+00

0.00000000E+00 0.00000000E+00 0.00000000E+00

0.00000000E+00 0.00000000E+00 0.00000000E+00

0.00000000E+00 0.00000000E+00 0.00000000E+00

0.00000000E+00 0.00000000E+00 0.00000000E+00

0.00000000E+00 0.00000000E+00 0.00000000E+00

0.00000000E+00 0.00000000E+00 0.00000000E+00

0.00000000E+00 0.00000000E+00 0.00000000E+00

0.00000000E+00 0.00000000E+00 0.00000000E+00

0.00000000E+00 0.00000000E+00 0.00000000E+00

0.00000000E+00 0.00000000E+00 0.00000000E+00

0.00000000E+00 0.00000000E+00 0.00000000E+00

0.00000000E+00 0.00000000E+00 0.00000000E+00

0.00000000E+00 0.00000000E+00 0.00000000E+00

0.00000000E+00 0.00000000E+00 0.00000000E+00

0.00000000E+00 0.00000000E+00 0.00000000E+00

0.00000000E+00 0.00000000E+00 0.00000000E+00

0.00000000E+00 0.00000000E+00 0.00000000E+00

0.00000000E+00 0.00000000E+00 0.00000000E+00

0.00000000E+00 0.00000000E+00 0.00000000E+00

0.00000000E+00 0.00000000E+00 0.00000000E+00

0.00000000E+00 0.00000000E+00 0.00000000E+00

0.00000000E+00 0.00000000E+00 0.00000000E+00

0.00000000E+00 0.00000000E+00 0.00000000E+00

0.00000000E+00 0.00000000E+00 0.00000000E+00

0.00000000E+00 0.00000000E+00 0.00000000E+00

0.00000000E+00 0.00000000E+00 0.00000000E+00

0.00000000E+00 0.00000000E+00 0.00000000E+00

0.00000000E+00 0.00000000E+00 0.00000000E+00

0.00000000E+00 0.00000000E+00 0.00000000E+00

0.00000000E+00 0.00000000E+00 0.00000000E+00

0.00000000E+00 0.00000000E+00 0.00000000E+00

0.00000000E+00 0.00000000E+00 0.00000000E+00

0.00000000E+00 0.00000000E+00 0.00000000E+00

0.00000000E+00 0.00000000E+00 0.00000000E+00

0.00000000E+00 0.00000000E+00 0.00000000E+00

0.00000000E+00 0.00000000E+00 0.00000000E+00

0.00000000E+00 0.00000000E+00 0.00000000E+00

0.00000000E+00 0.00000000E+00 0.00000000E+00

0.00000000E+00 0.00000000E+00 0.00000000E+00

0.00000000E+00 0.00000000E+00 0.00000000E+00

0.00000000E+00 0.00000000E+00 0.00000000E+00

0.00000000E+00 0.00000000E+00 0.00000000E+00

0.00000000E+00 0.00000000E+00 0.00000000E+00

0.00000000E+00 0.00000000E+00 0.00000000E+00

0.00000000E+00 0.00000000E+00 0.00000000E+00

0.00000000E+00 0.00000000E+00 0.00000000E+00

0.00000000E+00 0.00000000E+00 0.00000000E+00

0.00000000E+00 0.00000000E+00 0.00000000E+00

0.00000000E+00 0.00000000E+00 0.00000000E+00

0.00000000E+00 0.00000000E+00 0.00000000E+00

0.00000000E+00 0.00000000E+00 0.00000000E+00

0.00000000E+00 0.00000000E+00 0.00000000E+00

0.00000000E+00 0.00000000E+00 0.00000000E+00

0.00000000E+00 0.00000000E+00 0.00000000E+00

0.00000000E+00 0.00000000E+00 0.00000000E+00

0.00000000E+00 0.00000000E+00 0.00000000E+00

0.00000000E+00 0.00000000E+00 0.00000000E+00

0.00000000E+00 0.00000000E+00 0.00000000E+00

0.00000000E+00 0.00000000E+00 0.00000000E+00

0.00000000E+00 0.00000000E+00 0.00000000E+00

0.00000000E+00 0.00000000E+00 0.00000000E+00

0.00000000E+00 0.00000000E+00 0.00000000E+00

0.00000000E+00 0.00000000E+00 0.00000000E+00

0.00000000E+00 0.00000000E+00 0.00000000E+00

0.00000000E+00 0.00000000E+00 0.00000000E+00

0.00000000E+00 0.00000000E+00 0.00000000E+00

NiOOH_14H ML CONTCAR

This file is generated by VASPKIT code

1.00000000000000

17.5560007098000028 0.0000000000000000 0.0000000000000000

-8.7889406573999977 15.4086551811000021 0.0000000000000000

0.0000000000000015 4.9977513337000028 24.4953563273999961

Ni O H

72 144 68

Selective dynamics

Direct

0.0341653786323661 0.3260019713917757 0.1960457408563343 F F F

0.0341653786323661 0.6593353047251114 0.1960457408563343 F F F

0.0341653786323661 0.9926686380584400 0.1960457408563343 F F F

0.2008320452990304 0.3260019713917757 0.1960457408563343 F F F

0.2008320452990304 0.6593353047251114 0.1960457408563343 F F F

0.2008320452990304 0.9926686380584400 0.1960457408563343 F F F

0.3674987119657018 0.3260019713917757 0.1960457408563343 F F F

0.3674987119657018 0.6593353047251114 0.1960457408563343 F F F

0.3674987119657018 0.9926686380584400 0.1960457408563343 F F F

0.5341653786323661 0.3260019713917757 0.1960457408563343 F F F

0.5341653786323661 0.6593353047251114 0.1960457408563343 F F F

0.5341653786323661 0.9926686380584400 0.1960457408563343 F F F

0.7008320452990304 0.3260019713917757 0.1960457408563343 F F F

0.7008320452990304 0.6593353047251114 0.1960457408563343 F F F

0.7008320452990304 0.9926686380584400 0.1960457408563343 F F F

0.8674987119657018 0.3260019713917757 0.1960457408563343 F F F

0.8674987119657018 0.6593353047251114 0.1960457408563343 F F F

0.8674987119657018 0.9926686380584400 0.1960457408563343 F F F

0.0351970056773183 0.1617665099525851 0.1949035160852901 F F F

0.0351970056773183 0.4950998432859137 0.1949035160852901 F F F

0.0351970056773183 0.8284331766192494 0.1949035160852901 F F F

0.2018636723439897 0.1617665099525851 0.1949035160852901 F F F

0.2018636723439897 0.4950998432859137 0.1949035160852901 F F F

0.2018636723439897 0.8284331766192494 0.1949035160852901 F F F

0.3685303390106540 0.1617665099525851 0.1949035160852901 F F F

0.3685303390106540 0.4950998432859137 0.1949035160852901 F F F

0.3685303390106540 0.8284331766192494 0.1949035160852901 F F F

0.5351970056773183 0.1617665099525851 0.1949035160852901 F F F

0.5351970056773183 0.4950998432859137 0.1949035160852901 F F F

0.5351970056773183 0.8284331766192494 0.1949035160852901 F F F

0.7018636723439897 0.1617665099525851 0.1949035160852901 F F F

0.7018636723439897 0.4950998432859137 0.1949035160852901 F F F

0.7018636723439897 0.8284331766192494 0.1949035160852901 F F F

0.8685303390106540 0.1617665099525851 0.1949035160852901 F F F

0.8685303390106540 0.4950998432859137 0.1949035160852901 F F F

0.8685303390106540 0.8284331766192494 0.1949035160852901 F F F

0.1341778441284041 0.0083483795191240 0.3796593021734443 T T T

0.1342365481216406 0.3411238862389627 0.3799845496702871 T T T

0.1307033406230526 0.6740379053183976 0.3789941735124395 T T T

0.3008420458124753 0.0084035525575358 0.3799675734606978 T T T

0.3022718652951653 0.3417529898892487 0.3771267126422755 T T T

0.3001179014867854 0.6743207247475855 0.3802990749996052 T T T

0.4686388643505774 0.0082200262861912 0.3773875874334748 T T T

0.4631684153888586 0.3417596981685704 0.3790375643367427 T T T

0.4688424797392257 0.6756140972127407 0.3774913587218610 T T T

0.6298196367618785 0.0081769005980471 0.3789718500015214 T T T

0.6320978290558187 0.3417741508883915 0.3804293223744079 T T T

0.6304257562105738 0.6746775590313758 0.3788555486929139 T T T

0.7987581262463728 0.0079102588771231 0.3802495353875355 T T T

0.7999618406882957 0.3421489474774187 0.3796209394583628 T T T

0.7996034284008210 0.6739391412670717 0.3805407543808880 T T T

0.9665554894668290 0.0078742328552516 0.3796973513127305 T T T

0.9669636610306739 0.3415752739604461 0.3799465180981349 T T T

0.9689667977919568 0.6746936554741213 0.3775012831471319 T T T

0.1326180990590381 0.1721875891681026 0.3798572396243992 T T T

0.1324048142390286 0.5056365242163546 0.3809111435426145 T T T

0.1326257985605296 0.8393154069653701 0.3807009171502426 T T T

0.3000163920693297 0.1745965697928163 0.3800295699448740 T T T

0.3002326313778473 0.5058906531507084 0.3799836979616062 T T T

0.2988730043908092 0.8387568044032415 0.3798631981805577 T T T

0.4660688958860268 0.1731148160442565 0.3808936168660187 T T T

0.4662107140958510 0.5086645985180401 0.3796884774290027 T T T

0.4665742307272489 0.8412441396453616 0.3801790952422008 T T T

0.6320396632608875 0.1734302107046091 0.3804849367840303 T T T

0.6307908985603521 0.5060485720247663 0.3809390786906344 T T T

0.6319094471734397 0.8402266446957111 0.3803048031703901 T T T

0.7977917845130748 0.1722955398153959 0.3809336510115500 T T T

0.7984857654128703 0.5055678984819104 0.3800459829266587 T T T

0.7981420297929473 0.8387806053300082 0.3806295206978683 T T T

0.9649236116210114 0.1717800842012399 0.3811221308891987 T T T

0.9661803997622400 0.5076170980664290 0.3801054444022759 T T T

0.9661191430800046 0.8386930388661714 0.3809538703168899 T T T

0.0826462090432543 0.0913673776135795 0.2306510691320725 F F F

0.0826462090432543 0.4247007109469081 0.2306510691320725 F F F

0.0826462090432543 0.7580340442802367 0.2306510691320725 F F F

0.2493128757099186 0.0913673776135795 0.2306510691320725 F F F

0.2493128757099186 0.4247007109469081 0.2306510691320725 F F F

0.2493128757099186 0.7580340442802367 0.2306510691320725 F F F

0.4159795423765829 0.0913673776135795 0.2306510691320725 F F F

0.4159795423765829 0.4247007109469081 0.2306510691320725 F F F

0.4159795423765829 0.7580340442802367 0.2306510691320725 F F F

0.5826462090432472 0.0913673776135795 0.2306510691320725 F F F

0.5826462090432472 0.4247007109469081 0.2306510691320725 F F F

0.5826462090432472 0.7580340442802367 0.2306510691320725 F F F

0.7493128757099186 0.0913673776135795 0.2306510691320725 F F F

0.7493128757099186 0.4247007109469081 0.2306510691320725 F F F

0.7493128757099186 0.7580340442802367 0.2306510691320725 F F F

0.9159795423765758 0.0913673776135795 0.2306510691320725 F F F

0.9159795423765758 0.4247007109469081 0.2306510691320725 F F F

0.9159795423765758 0.7580340442802367 0.2306510691320725 F F F

0.0902677041989364 0.2625098265214945 0.2403988745878252 F F F

0.0902677041989364 0.5958431598548302 0.2403988745878252 F F F

0.0902677041989364 0.9291764931881659 0.2403988745878252 F F F

0.2569343708656078 0.2625098265214945 0.2403988745878252 F F F

0.2569343708656078 0.5958431598548302 0.2403988745878252 F F F

0.2569343708656078 0.9291764931881659 0.2403988745878252 F F F

0.4236010375322721 0.2625098265214945 0.2403988745878252 F F F

0.4236010375322721 0.5958431598548302 0.2403988745878252 F F F

0.4236010375322721 0.9291764931881659 0.2403988745878252 F F F

0.5902677041989364 0.2625098265214945 0.2403988745878252 F F F

0.5902677041989364 0.5958431598548302 0.2403988745878252 F F F

0.5902677041989364 0.9291764931881659 0.2403988745878252 F F F

0.7569343708656078 0.2625098265214945 0.2403988745878252 F F F

0.7569343708656078 0.5958431598548302 0.2403988745878252 F F F

0.7569343708656078 0.9291764931881659 0.2403988745878252 F F F

0.9236010375322721 0.2625098265214945 0.2403988745878252 F F F

0.9236010375322721 0.5958431598548302 0.2403988745878252 F F F

0.9236010375322721 0.9291764931881659 0.2403988745878252 F F F

0.1450268573747806 0.0574030619262587 0.1502611028726761 F F F

0.1450268573747806 0.3907363952595873 0.1502611028726761 F F F

0.1450268573747806 0.7240697285929230 0.1502611028726761 F F F

0.3116935240414520 0.0574030619262587 0.1502611028726761 F F F

0.3116935240414520 0.3907363952595873 0.1502611028726761 F F F

0.3116935240414520 0.7240697285929230 0.1502611028726761 F F F

0.4783601907081163 0.0574030619262587 0.1502611028726761 F F F

0.4783601907081163 0.3907363952595873 0.1502611028726761 F F F

0.4783601907081163 0.7240697285929230 0.1502611028726761 F F F

0.6450268573747806 0.0574030619262587 0.1502611028726761 F F F

0.6450268573747806 0.3907363952595873 0.1502611028726761 F F F

0.6450268573747806 0.7240697285929230 0.1502611028726761 F F F

0.8116935240414520 0.0574030619262587 0.1502611028726761 F F F

0.8116935240414520 0.3907363952595873 0.1502611028726761 F F F

0.8116935240414520 0.7240697285929230 0.1502611028726761 F F F

0.9783601907081163 0.0574030619262587 0.1502611028726761 F F F

0.9783601907081163 0.3907363952595873 0.1502611028726761 F F F

0.9783601907081163 0.7240697285929230 0.1502611028726761 F F F

0.1540633143111236 0.2313171148045612 0.1594044809199673 F F F

0.1540633143111236 0.5646504481378969 0.1594044809199673 F F F

0.1540633143111236 0.8979837814712326 0.1594044809199673 F F F

0.3207299809777879 0.2313171148045612 0.1594044809199673 F F F

0.3207299809777879 0.5646504481378969 0.1594044809199673 F F F

0.3207299809777879 0.8979837814712326 0.1594044809199673 F F F

0.4873966476444522 0.2313171148045612 0.1594044809199673 F F F

0.4873966476444522 0.5646504481378969 0.1594044809199673 F F F

0.4873966476444522 0.8979837814712326 0.1594044809199673 F F F

0.6540633143111236 0.2313171148045612 0.1594044809199673 F F F

0.6540633143111236 0.5646504481378969 0.1594044809199673 F F F

0.6540633143111236 0.8979837814712326 0.1594044809199673 F F F

0.8207299809777879 0.2313171148045612 0.1594044809199673 F F F

0.8207299809777879 0.5646504481378969 0.1594044809199673 F F F

0.8207299809777879 0.8979837814712326 0.1594044809199673 F F F

0.9873966476444522 0.2313171148045612 0.1594044809199673 F F F

0.9873966476444522 0.5646504481378969 0.1594044809199673 F F F

0.9873966476444522 0.8979837814712326 0.1594044809199673 F F F

0.0138739921410292 0.1026439377850292 0.4156910875323785 T T T

0.0137548072481369 0.4370770495774952 0.4154755934127711 T T T

0.0140664374873239 0.7681116886256099 0.4149658747092967 T T T

0.1814269381813113 0.1041686095422922 0.4147689402319874 T T T

0.1816230361406014 0.4360476150011088 0.4153836153272038 T T T

0.1799867821221534 0.7705804628266451 0.4148087936530001 T T T

0.3473826195244393 0.1037239304164086 0.4151863832433845 T T T

0.3475693478355635 0.4360567951758590 0.4140393420326966 T T T

0.3477246755062344 0.7701825675182334 0.4148228816866386 T T T

0.5134862190813145 0.1018871514007477 0.4148104185292923 T T T

0.5116646290765984 0.4390103868213907 0.4148273953638729 T T T

0.5134297897580387 0.7696340139224690 0.4146193150889478 T T T

0.6785440141507871 0.1043754902597086 0.4150253642617102 T T T

0.6795944237150749 0.4364270700804525 0.4161368195435703 T T T

0.6790287149166016 0.7709825229920474 0.4146322782879233 T T T

0.8459219669423540 0.1024264533316821 0.4161599279104355 T T T

0.8474681615466039 0.4376736068916961 0.4150155897383829 T T T

0.8471405811674152 0.7688953771169119 0.4156044782484501 T T T

0.0231225280631712 0.2770237983011297 0.4252971357831394 T T T

0.0194541479122720 0.6186947231757348 0.4185669774538105 T T T

0.0225270689389473 0.9433396967673963 0.4247105546651123 T T T

0.1960142693657825 0.2795271018947778 0.4228103120021570 T T T

0.1877778750902258 0.6096730031057433 0.4248053097604802 T T T

0.1898359083341132 0.9437332211909546 0.4252030538405843 T T T

0.3522001528175514 0.2856420715728844 0.4185085345693649 T T T

0.3624339142404209 0.6123781416803683 0.4231013364044227 T T T

0.3624229626418508 0.9459788552752781 0.4229081702413275 T T T

0.5187121174629477 0.2760698605988474 0.4248232692388658 T T T

0.5193034597260218 0.6196427100469686 0.4184645658497566 T T T

0.5186274246021011 0.9518875047472101 0.4186201867657476 T T T

0.6885245659447564 0.2779253288640384 0.4245496241120486 T T T

0.6864163105664738 0.6095564572803930 0.4247896163087512 T T T

0.6858517110059018 0.9430379899614988 0.4243576800404418 T T T

0.8553979446726518 0.2772754597885409 0.4248946450674813 T T T

0.8624080655774156 0.6128424824200046 0.4228819417709119 T T T

0.8551164316104591 0.9434380001403430 0.4245463479261105 T T T

0.0773586229022234 0.0707803824424576 0.3361364887841176 T T T

0.0789526445826998 0.4037058734256125 0.3365478190225386 T T T

0.0748382277726454 0.7393477969448520 0.3338925616292693 T T T

0.2453753850040420 0.0701894799342739 0.3362824842925021 T T T

0.2475463876163253 0.4016636450025378 0.3363557240874958 T T T

0.2432946693053643 0.7358706645908940 0.3367177555571887 T T T

0.4141841273600345 0.0683858403455880 0.3367476997858709 T T T

0.4076155254482218 0.4060888043970191 0.3335516363664125 T T T

0.4133007447676268 0.7344605512582327 0.3369510027463363 T T T

0.5742641554008310 0.0732760660814530 0.3337808219821694 T T T

0.5751318353083666 0.4036134462096198 0.3372116720031950 T T T

0.5743550429072798 0.7399229733035032 0.3337876125875158 T T T

0.7419976163068438 0.0704232971317805 0.3369790085162960 T T T

0.7433244954524200 0.4051134351557595 0.3363233807108198 T T T

0.7436904216040967 0.7358707766779187 0.3370198219597146 T T T

0.9101314475171750 0.0711541059381430 0.3366086391153042 T T T

0.9107578348165704 0.4039274060799644 0.3363864222320669 T T T

0.9140797802247580 0.7345895298375364 0.3370089920399897 T T T

0.0848582190109239 0.2425079491328234 0.3462207267440444 T T T

0.0841833829381459 0.5748621446056028 0.3453125606100269 T T T

0.0853576506915099 0.9094489052770501 0.3458941095205466 T T T

0.2512296899974936 0.2433493321824222 0.3434827535503292 T T T

0.2518112557814045 0.5757760478446224 0.3461973001868436 T T T

0.2517812757018157 0.9096787061289172 0.3459786344695430 T T T

0.4172454452225199 0.2419324125402784 0.3451259880349178 T T T

0.4182936216803664 0.5774406552445448 0.3435041689043911 T T T

0.4178407194349487 0.9098463582226988 0.3435284838020444 T T T

0.5845185387989353 0.2437068536908764 0.3458240045101693 T T T

0.5836933845595625 0.5759239618577302 0.3448358420002816 T T T

0.5836436021022506 0.9092436526253629 0.3449139704535592 T T T

0.7510011618414342 0.2432447506445542 0.3456482653803676 T T T

0.7509815349942508 0.5758966571871765 0.3462859990351762 T T T

0.7509493601426502 0.9098103453422162 0.3455571499729028 T T T

0.9180364279492538 0.2424430814122960 0.3462464225933858 T T T

0.9178215069826946 0.5766396285296439 0.3436648008803332 T T T

0.9182043327660971 0.9088639133093450 0.3458071880698503 T T T

0.1397131416109474 0.0545266729236502 0.1118484288951365 F F F

0.1397131416109474 0.3878600062569788 0.1118484288951365 F F F

0.1397131416109474 0.7211933395903145 0.1118484288951365 F F F

0.3063798082776117 0.0545266729236502 0.1118484288951365 F F F

0.3063798082776117 0.3878600062569788 0.1118484288951365 F F F

0.3063798082776117 0.7211933395903145 0.1118484288951365 F F F

0.4730464749442831 0.0545266729236502 0.1118484288951365 F F F

0.4730464749442831 0.3878600062569788 0.1118484288951365 F F F

0.4730464749442831 0.7211933395903145 0.1118484288951365 F F F

0.6397131416109474 0.0545266729236502 0.1118484288951365 F F F

0.6397131416109474 0.3878600062569788 0.1118484288951365 F F F

0.6397131416109474 0.7211933395903145 0.1118484288951365 F F F

0.8063798082776117 0.0545266729236502 0.1118484288951365 F F F

0.8063798082776117 0.3878600062569788 0.1118484288951365 F F F

0.8063798082776117 0.7211933395903145 0.1118484288951365 F F F

0.9730464749442831 0.0545266729236502 0.1118484288951365 F F F

0.9730464749442831 0.3878600062569788 0.1118484288951365 F F F

0.9730464749442831 0.7211933395903145 0.1118484288951365 F F F

0.0904969084302252 0.2563302902655593 0.2814745639703204 F F F

0.0904969084302252 0.5896636235988879 0.2814745639703204 F F F

0.0904969084302252 0.9229969569322236 0.2814745639703204 F F F

0.2571635750968966 0.2563302902655593 0.2814745639703204 F F F

0.2571635750968966 0.5896636235988879 0.2814745639703204 F F F

0.2571635750968966 0.9229969569322236 0.2814745639703204 F F F

0.4238302417635609 0.2563302902655593 0.2814745639703204 F F F

0.4238302417635609 0.5896636235988879 0.2814745639703204 F F F

0.4238302417635609 0.9229969569322236 0.2814745639703204 F F F

0.5904969084302252 0.2563302902655593 0.2814745639703204 F F F

0.5904969084302252 0.5896636235988879 0.2814745639703204 F F F

0.5904969084302252 0.9229969569322236 0.2814745639703204 F F F

0.7571635750968895 0.2563302902655593 0.2814745639703204 F F F

0.7571635750968895 0.5896636235988879 0.2814745639703204 F F F

0.7571635750968895 0.9229969569322236 0.2814745639703204 F F F

0.9238302417635609 0.2563302902655593 0.2814745639703204 F F F

0.9238302417635609 0.5896636235988879 0.2814745639703204 F F F

0.9238302417635609 0.9229969569322236 0.2814745639703204 F F F

0.0769978323231373 0.0771269488791722 0.2949185016344884 T T T

0.0785471746022823 0.4101559633009005 0.2953622280579146 T T T

0.0730569872814528 0.7442961315571061 0.2921113960189579 T T T

0.2448254515324907 0.0761633768687228 0.2950970959245297 T T T

0.2478484746207195 0.4075896641474582 0.2947210011205520 T T T

0.2435278116996160 0.7424026873277652 0.2955009069133217 T T T

0.4144381289785175 0.0741083795632347 0.2952185514830280 T T T

0.4060082833619123 0.4104205619465738 0.2917811492160777 T T T

0.4136986702740365 0.7400023342912271 0.2954769576104953 T T T

0.5727097480490122 0.0777952035863377 0.2920276856015602 T T T

0.5756471203108237 0.4097586867887050 0.2961113582100112 T T T

0.5724804175989040 0.7445467349850141 0.2920919323025503 T T T

0.7425579366564333 0.0767905699688736 0.2958564241675295 T T T

0.7430647645154448 0.4113377555633245 0.2952023635212148 T T T

0.7441911093729545 0.7422516138673061 0.2958499315323992 T T T

0.9098804348610483 0.0776234417787710 0.2954857459040660 T T T

0.9103646006042861 0.4101030509063449 0.2952372234191940 T T T

0.9146679434361246 0.7405931378367452 0.2955163900856589 T T T

0.0278145854135242 0.2795141042244542 0.4637755948414939 T T T

0.0270603227291180 0.9448639722762938 0.4633003443487653 T T T

0.2099433800568925 0.2823193421556500 0.4604317934555188 T T T

0.1889342834691584 0.6103150657598932 0.4636159979696853 T T T

0.1943463568776876 0.9451639227229586 0.4638152017031965 T T T

0.3758784304708719 0.6132535075243006 0.4609203617918943 T T T

0.3763549588308689 0.9474942932444461 0.4606056999636321 T T T

0.5190752689497595 0.2747348654314929 0.4638565572185855 T T T

0.6928665221107224 0.2785464123291020 0.4632503587549000 T T T

0.6856024867891662 0.6084273839711447 0.4638083894250881 T T T

0.6861148935504792 0.9407800588723960 0.4634758240429037 T T T

0.8600199332191245 0.2788308182626818 0.4634802706423484 T T T

0.8754978291096883 0.6152032836520052 0.4606676926469025 T T T

0.8594678674374724 0.9445689306815453 0.4631914228578991 T T T

0.00000000E+00 0.00000000E+00 0.00000000E+00

0.00000000E+00 0.00000000E+00 0.00000000E+00

0.00000000E+00 0.00000000E+00 0.00000000E+00

0.00000000E+00 0.00000000E+00 0.00000000E+00

0.00000000E+00 0.00000000E+00 0.00000000E+00

0.00000000E+00 0.00000000E+00 0.00000000E+00

0.00000000E+00 0.00000000E+00 0.00000000E+00

0.00000000E+00 0.00000000E+00 0.00000000E+00

0.00000000E+00 0.00000000E+00 0.00000000E+00

0.00000000E+00 0.00000000E+00 0.00000000E+00

0.00000000E+00 0.00000000E+00 0.00000000E+00

0.00000000E+00 0.00000000E+00 0.00000000E+00

0.00000000E+00 0.00000000E+00 0.00000000E+00

0.00000000E+00 0.00000000E+00 0.00000000E+00

0.00000000E+00 0.00000000E+00 0.00000000E+00

0.00000000E+00 0.00000000E+00 0.00000000E+00

0.00000000E+00 0.00000000E+00 0.00000000E+00

0.00000000E+00 0.00000000E+00 0.00000000E+00

0.00000000E+00 0.00000000E+00 0.00000000E+00

0.00000000E+00 0.00000000E+00 0.00000000E+00

0.00000000E+00 0.00000000E+00 0.00000000E+00

0.00000000E+00 0.00000000E+00 0.00000000E+00

0.00000000E+00 0.00000000E+00 0.00000000E+00

0.00000000E+00 0.00000000E+00 0.00000000E+00

0.00000000E+00 0.00000000E+00 0.00000000E+00

0.00000000E+00 0.00000000E+00 0.00000000E+00

0.00000000E+00 0.00000000E+00 0.00000000E+00

0.00000000E+00 0.00000000E+00 0.00000000E+00

0.00000000E+00 0.00000000E+00 0.00000000E+00

0.00000000E+00 0.00000000E+00 0.00000000E+00

0.00000000E+00 0.00000000E+00 0.00000000E+00

0.00000000E+00 0.00000000E+00 0.00000000E+00

0.00000000E+00 0.00000000E+00 0.00000000E+00

0.00000000E+00 0.00000000E+00 0.00000000E+00

0.00000000E+00 0.00000000E+00 0.00000000E+00

0.00000000E+00 0.00000000E+00 0.00000000E+00

0.00000000E+00 0.00000000E+00 0.00000000E+00

0.00000000E+00 0.00000000E+00 0.00000000E+00

0.00000000E+00 0.00000000E+00 0.00000000E+00

0.00000000E+00 0.00000000E+00 0.00000000E+00

0.00000000E+00 0.00000000E+00 0.00000000E+00

0.00000000E+00 0.00000000E+00 0.00000000E+00

0.00000000E+00 0.00000000E+00 0.00000000E+00

0.00000000E+00 0.00000000E+00 0.00000000E+00

0.00000000E+00 0.00000000E+00 0.00000000E+00

0.00000000E+00 0.00000000E+00 0.00000000E+00

0.00000000E+00 0.00000000E+00 0.00000000E+00

0.00000000E+00 0.00000000E+00 0.00000000E+00

0.00000000E+00 0.00000000E+00 0.00000000E+00

0.00000000E+00 0.00000000E+00 0.00000000E+00

0.00000000E+00 0.00000000E+00 0.00000000E+00

0.00000000E+00 0.00000000E+00 0.00000000E+00

0.00000000E+00 0.00000000E+00 0.00000000E+00

0.00000000E+00 0.00000000E+00 0.00000000E+00

0.00000000E+00 0.00000000E+00 0.00000000E+00

0.00000000E+00 0.00000000E+00 0.00000000E+00

0.00000000E+00 0.00000000E+00 0.00000000E+00

0.00000000E+00 0.00000000E+00 0.00000000E+00

0.00000000E+00 0.00000000E+00 0.00000000E+00

0.00000000E+00 0.00000000E+00 0.00000000E+00

0.00000000E+00 0.00000000E+00 0.00000000E+00

0.00000000E+00 0.00000000E+00 0.00000000E+00

0.00000000E+00 0.00000000E+00 0.00000000E+00

0.00000000E+00 0.00000000E+00 0.00000000E+00

0.00000000E+00 0.00000000E+00 0.00000000E+00

0.00000000E+00 0.00000000E+00 0.00000000E+00

0.00000000E+00 0.00000000E+00 0.00000000E+00

0.00000000E+00 0.00000000E+00 0.00000000E+00

0.00000000E+00 0.00000000E+00 0.00000000E+00

0.00000000E+00 0.00000000E+00 0.00000000E+00

0.00000000E+00 0.00000000E+00 0.00000000E+00

0.00000000E+00 0.00000000E+00 0.00000000E+00

0.00000000E+00 0.00000000E+00 0.00000000E+00

0.00000000E+00 0.00000000E+00 0.00000000E+00

0.00000000E+00 0.00000000E+00 0.00000000E+00

0.00000000E+00 0.00000000E+00 0.00000000E+00

0.00000000E+00 0.00000000E+00 0.00000000E+00

0.00000000E+00 0.00000000E+00 0.00000000E+00

0.00000000E+00 0.00000000E+00 0.00000000E+00

0.00000000E+00 0.00000000E+00 0.00000000E+00

0.00000000E+00 0.00000000E+00 0.00000000E+00

0.00000000E+00 0.00000000E+00 0.00000000E+00

0.00000000E+00 0.00000000E+00 0.00000000E+00

0.00000000E+00 0.00000000E+00 0.00000000E+00

0.00000000E+00 0.00000000E+00 0.00000000E+00

0.00000000E+00 0.00000000E+00 0.00000000E+00

0.00000000E+00 0.00000000E+00 0.00000000E+00

0.00000000E+00 0.00000000E+00 0.00000000E+00

0.00000000E+00 0.00000000E+00 0.00000000E+00

0.00000000E+00 0.00000000E+00 0.00000000E+00

0.00000000E+00 0.00000000E+00 0.00000000E+00

0.00000000E+00 0.00000000E+00 0.00000000E+00

0.00000000E+00 0.00000000E+00 0.00000000E+00

0.00000000E+00 0.00000000E+00 0.00000000E+00

0.00000000E+00 0.00000000E+00 0.00000000E+00

0.00000000E+00 0.00000000E+00 0.00000000E+00

0.00000000E+00 0.00000000E+00 0.00000000E+00

0.00000000E+00 0.00000000E+00 0.00000000E+00

0.00000000E+00 0.00000000E+00 0.00000000E+00

0.00000000E+00 0.00000000E+00 0.00000000E+00

0.00000000E+00 0.00000000E+00 0.00000000E+00

0.00000000E+00 0.00000000E+00 0.00000000E+00

0.00000000E+00 0.00000000E+00 0.00000000E+00

0.00000000E+00 0.00000000E+00 0.00000000E+00

0.00000000E+00 0.00000000E+00 0.00000000E+00

0.00000000E+00 0.00000000E+00 0.00000000E+00

0.00000000E+00 0.00000000E+00 0.00000000E+00

0.00000000E+00 0.00000000E+00 0.00000000E+00

0.00000000E+00 0.00000000E+00 0.00000000E+00

0.00000000E+00 0.00000000E+00 0.00000000E+00

0.00000000E+00 0.00000000E+00 0.00000000E+00

0.00000000E+00 0.00000000E+00 0.00000000E+00

0.00000000E+00 0.00000000E+00 0.00000000E+00

0.00000000E+00 0.00000000E+00 0.00000000E+00

0.00000000E+00 0.00000000E+00 0.00000000E+00

0.00000000E+00 0.00000000E+00 0.00000000E+00

0.00000000E+00 0.00000000E+00 0.00000000E+00

0.00000000E+00 0.00000000E+00 0.00000000E+00

0.00000000E+00 0.00000000E+00 0.00000000E+00

0.00000000E+00 0.00000000E+00 0.00000000E+00

0.00000000E+00 0.00000000E+00 0.00000000E+00

0.00000000E+00 0.00000000E+00 0.00000000E+00

0.00000000E+00 0.00000000E+00 0.00000000E+00

0.00000000E+00 0.00000000E+00 0.00000000E+00

0.00000000E+00 0.00000000E+00 0.00000000E+00

0.00000000E+00 0.00000000E+00 0.00000000E+00

0.00000000E+00 0.00000000E+00 0.00000000E+00

0.00000000E+00 0.00000000E+00 0.00000000E+00

0.00000000E+00 0.00000000E+00 0.00000000E+00

0.00000000E+00 0.00000000E+00 0.00000000E+00

0.00000000E+00 0.00000000E+00 0.00000000E+00

0.00000000E+00 0.00000000E+00 0.00000000E+00

0.00000000E+00 0.00000000E+00 0.00000000E+00

0.00000000E+00 0.00000000E+00 0.00000000E+00

0.00000000E+00 0.00000000E+00 0.00000000E+00

0.00000000E+00 0.00000000E+00 0.00000000E+00

0.00000000E+00 0.00000000E+00 0.00000000E+00

0.00000000E+00 0.00000000E+00 0.00000000E+00

0.00000000E+00 0.00000000E+00 0.00000000E+00

0.00000000E+00 0.00000000E+00 0.00000000E+00

0.00000000E+00 0.00000000E+00 0.00000000E+00

0.00000000E+00 0.00000000E+00 0.00000000E+00

0.00000000E+00 0.00000000E+00 0.00000000E+00

0.00000000E+00 0.00000000E+00 0.00000000E+00

0.00000000E+00 0.00000000E+00 0.00000000E+00

0.00000000E+00 0.00000000E+00 0.00000000E+00

0.00000000E+00 0.00000000E+00 0.00000000E+00

0.00000000E+00 0.00000000E+00 0.00000000E+00

0.00000000E+00 0.00000000E+00 0.00000000E+00

0.00000000E+00 0.00000000E+00 0.00000000E+00

0.00000000E+00 0.00000000E+00 0.00000000E+00

0.00000000E+00 0.00000000E+00 0.00000000E+00

0.00000000E+00 0.00000000E+00 0.00000000E+00

0.00000000E+00 0.00000000E+00 0.00000000E+00

0.00000000E+00 0.00000000E+00 0.00000000E+00

0.00000000E+00 0.00000000E+00 0.00000000E+00

0.00000000E+00 0.00000000E+00 0.00000000E+00

0.00000000E+00 0.00000000E+00 0.00000000E+00

0.00000000E+00 0.00000000E+00 0.00000000E+00

0.00000000E+00 0.00000000E+00 0.00000000E+00

0.00000000E+00 0.00000000E+00 0.00000000E+00

0.00000000E+00 0.00000000E+00 0.00000000E+00

0.00000000E+00 0.00000000E+00 0.00000000E+00

0.00000000E+00 0.00000000E+00 0.00000000E+00

0.00000000E+00 0.00000000E+00 0.00000000E+00

0.00000000E+00 0.00000000E+00 0.00000000E+00

0.00000000E+00 0.00000000E+00 0.00000000E+00

0.00000000E+00 0.00000000E+00 0.00000000E+00

0.00000000E+00 0.00000000E+00 0.00000000E+00

0.00000000E+00 0.00000000E+00 0.00000000E+00

0.00000000E+00 0.00000000E+00 0.00000000E+00

0.00000000E+00 0.00000000E+00 0.00000000E+00

0.00000000E+00 0.00000000E+00 0.00000000E+00

0.00000000E+00 0.00000000E+00 0.00000000E+00

0.00000000E+00 0.00000000E+00 0.00000000E+00

0.00000000E+00 0.00000000E+00 0.00000000E+00

0.00000000E+00 0.00000000E+00 0.00000000E+00

0.00000000E+00 0.00000000E+00 0.00000000E+00

0.00000000E+00 0.00000000E+00 0.00000000E+00

0.00000000E+00 0.00000000E+00 0.00000000E+00

0.00000000E+00 0.00000000E+00 0.00000000E+00

0.00000000E+00 0.00000000E+00 0.00000000E+00

0.00000000E+00 0.00000000E+00 0.00000000E+00

0.00000000E+00 0.00000000E+00 0.00000000E+00

0.00000000E+00 0.00000000E+00 0.00000000E+00

0.00000000E+00 0.00000000E+00 0.00000000E+00

0.00000000E+00 0.00000000E+00 0.00000000E+00

0.00000000E+00 0.00000000E+00 0.00000000E+00

0.00000000E+00 0.00000000E+00 0.00000000E+00

0.00000000E+00 0.00000000E+00 0.00000000E+00

0.00000000E+00 0.00000000E+00 0.00000000E+00

0.00000000E+00 0.00000000E+00 0.00000000E+00

0.00000000E+00 0.00000000E+00 0.00000000E+00

0.00000000E+00 0.00000000E+00 0.00000000E+00

0.00000000E+00 0.00000000E+00 0.00000000E+00

0.00000000E+00 0.00000000E+00 0.00000000E+00

0.00000000E+00 0.00000000E+00 0.00000000E+00

0.00000000E+00 0.00000000E+00 0.00000000E+00

0.00000000E+00 0.00000000E+00 0.00000000E+00

0.00000000E+00 0.00000000E+00 0.00000000E+00

0.00000000E+00 0.00000000E+00 0.00000000E+00

0.00000000E+00 0.00000000E+00 0.00000000E+00

0.00000000E+00 0.00000000E+00 0.00000000E+00

0.00000000E+00 0.00000000E+00 0.00000000E+00

0.00000000E+00 0.00000000E+00 0.00000000E+00

0.00000000E+00 0.00000000E+00 0.00000000E+00

0.00000000E+00 0.00000000E+00 0.00000000E+00

0.00000000E+00 0.00000000E+00 0.00000000E+00

0.00000000E+00 0.00000000E+00 0.00000000E+00

0.00000000E+00 0.00000000E+00 0.00000000E+00

0.00000000E+00 0.00000000E+00 0.00000000E+00

0.00000000E+00 0.00000000E+00 0.00000000E+00

0.00000000E+00 0.00000000E+00 0.00000000E+00

0.00000000E+00 0.00000000E+00 0.00000000E+00

0.00000000E+00 0.00000000E+00 0.00000000E+00

0.00000000E+00 0.00000000E+00 0.00000000E+00

0.00000000E+00 0.00000000E+00 0.00000000E+00

0.00000000E+00 0.00000000E+00 0.00000000E+00

0.00000000E+00 0.00000000E+00 0.00000000E+00

0.00000000E+00 0.00000000E+00 0.00000000E+00

0.00000000E+00 0.00000000E+00 0.00000000E+00

0.00000000E+00 0.00000000E+00 0.00000000E+00

0.00000000E+00 0.00000000E+00 0.00000000E+00

0.00000000E+00 0.00000000E+00 0.00000000E+00

0.00000000E+00 0.00000000E+00 0.00000000E+00

0.00000000E+00 0.00000000E+00 0.00000000E+00

0.00000000E+00 0.00000000E+00 0.00000000E+00

0.00000000E+00 0.00000000E+00 0.00000000E+00

0.00000000E+00 0.00000000E+00 0.00000000E+00

0.00000000E+00 0.00000000E+00 0.00000000E+00

0.00000000E+00 0.00000000E+00 0.00000000E+00

0.00000000E+00 0.00000000E+00 0.00000000E+00

0.00000000E+00 0.00000000E+00 0.00000000E+00

0.00000000E+00 0.00000000E+00 0.00000000E+00

0.00000000E+00 0.00000000E+00 0.00000000E+00

0.00000000E+00 0.00000000E+00 0.00000000E+00

0.00000000E+00 0.00000000E+00 0.00000000E+00

0.00000000E+00 0.00000000E+00 0.00000000E+00

0.00000000E+00 0.00000000E+00 0.00000000E+00

0.00000000E+00 0.00000000E+00 0.00000000E+00

0.00000000E+00 0.00000000E+00 0.00000000E+00

0.00000000E+00 0.00000000E+00 0.00000000E+00

0.00000000E+00 0.00000000E+00 0.00000000E+00

0.00000000E+00 0.00000000E+00 0.00000000E+00

0.00000000E+00 0.00000000E+00 0.00000000E+00

0.00000000E+00 0.00000000E+00 0.00000000E+00

0.00000000E+00 0.00000000E+00 0.00000000E+00

0.00000000E+00 0.00000000E+00 0.00000000E+00

0.00000000E+00 0.00000000E+00 0.00000000E+00

0.00000000E+00 0.00000000E+00 0.00000000E+00

0.00000000E+00 0.00000000E+00 0.00000000E+00

0.00000000E+00 0.00000000E+00 0.00000000E+00

0.00000000E+00 0.00000000E+00 0.00000000E+00

0.00000000E+00 0.00000000E+00 0.00000000E+00

0.00000000E+00 0.00000000E+00 0.00000000E+00

0.00000000E+00 0.00000000E+00 0.00000000E+00

0.00000000E+00 0.00000000E+00 0.00000000E+00

0.00000000E+00 0.00000000E+00 0.00000000E+00

0.00000000E+00 0.00000000E+00 0.00000000E+00

0.00000000E+00 0.00000000E+00 0.00000000E+00

0.00000000E+00 0.00000000E+00 0.00000000E+00

0.00000000E+00 0.00000000E+00 0.00000000E+00

0.00000000E+00 0.00000000E+00 0.00000000E+00

0.00000000E+00 0.00000000E+00 0.00000000E+00

0.00000000E+00 0.00000000E+00 0.00000000E+00

0.00000000E+00 0.00000000E+00 0.00000000E+00

0.00000000E+00 0.00000000E+00 0.00000000E+00

0.00000000E+00 0.00000000E+00 0.00000000E+00

0.00000000E+00 0.00000000E+00 0.00000000E+00

0.00000000E+00 0.00000000E+00 0.00000000E+00

0.00000000E+00 0.00000000E+00 0.00000000E+00

0.00000000E+00 0.00000000E+00 0.00000000E+00

0.00000000E+00 0.00000000E+00 0.00000000E+00

0.00000000E+00 0.00000000E+00 0.00000000E+00

0.00000000E+00 0.00000000E+00 0.00000000E+00

0.00000000E+00 0.00000000E+00 0.00000000E+00

0.00000000E+00 0.00000000E+00 0.00000000E+00

0.00000000E+00 0.00000000E+00 0.00000000E+00

0.00000000E+00 0.00000000E+00 0.00000000E+00

0.00000000E+00 0.00000000E+00 0.00000000E+00

0.00000000E+00 0.00000000E+00 0.00000000E+00

0.00000000E+00 0.00000000E+00 0.00000000E+00

0.00000000E+00 0.00000000E+00 0.00000000E+00

0.00000000E+00 0.00000000E+00 0.00000000E+00

NiOOH_11H ML CONTCAR

This file is generated by VASPKIT code

1.00000000000000

17.5560007098000028 0.0000000000000000 0.0000000000000000

-8.7889406573999977 15.4086551811000021 0.0000000000000000

0.0000000000000015 4.9977513337000028 24.4953563273999961

Ni O H

72 144 65

Selective dynamics

Direct

0.0341653786323661 0.3260019713917757 0.1960457408563343 F F F

0.0341653786323661 0.6593353047251114 0.1960457408563343 F F F

0.0341653786323661 0.9926686380584400 0.1960457408563343 F F F

0.2008320452990304 0.3260019713917757 0.1960457408563343 F F F

0.2008320452990304 0.6593353047251114 0.1960457408563343 F F F

0.2008320452990304 0.9926686380584400 0.1960457408563343 F F F

0.3674987119657018 0.3260019713917757 0.1960457408563343 F F F

0.3674987119657018 0.6593353047251114 0.1960457408563343 F F F

0.3674987119657018 0.9926686380584400 0.1960457408563343 F F F

0.5341653786323661 0.3260019713917757 0.1960457408563343 F F F

0.5341653786323661 0.6593353047251114 0.1960457408563343 F F F

0.5341653786323661 0.9926686380584400 0.1960457408563343 F F F

0.7008320452990304 0.3260019713917757 0.1960457408563343 F F F

0.7008320452990304 0.6593353047251114 0.1960457408563343 F F F

0.7008320452990304 0.9926686380584400 0.1960457408563343 F F F

0.8674987119657018 0.3260019713917757 0.1960457408563343 F F F

0.8674987119657018 0.6593353047251114 0.1960457408563343 F F F

0.8674987119657018 0.9926686380584400 0.1960457408563343 F F F

0.0351970056773183 0.1617665099525851 0.1949035160852901 F F F

0.0351970056773183 0.4950998432859137 0.1949035160852901 F F F

0.0351970056773183 0.8284331766192494 0.1949035160852901 F F F

0.2018636723439897 0.1617665099525851 0.1949035160852901 F F F

0.2018636723439897 0.4950998432859137 0.1949035160852901 F F F

0.2018636723439897 0.8284331766192494 0.1949035160852901 F F F

0.3685303390106540 0.1617665099525851 0.1949035160852901 F F F

0.3685303390106540 0.4950998432859137 0.1949035160852901 F F F

0.3685303390106540 0.8284331766192494 0.1949035160852901 F F F

0.5351970056773183 0.1617665099525851 0.1949035160852901 F F F

0.5351970056773183 0.4950998432859137 0.1949035160852901 F F F

0.5351970056773183 0.8284331766192494 0.1949035160852901 F F F

0.7018636723439897 0.1617665099525851 0.1949035160852901 F F F

0.7018636723439897 0.4950998432859137 0.1949035160852901 F F F

0.7018636723439897 0.8284331766192494 0.1949035160852901 F F F

0.8685303390106540 0.1617665099525851 0.1949035160852901 F F F

0.8685303390106540 0.4950998432859137 0.1949035160852901 F F F

0.8685303390106540 0.8284331766192494 0.1949035160852901 F F F

0.1306676750642433 0.0067782901050504 0.3789967646526820 T T T

0.1343854981349137 0.3417789709451741 0.3802790366142826 T T T

0.1330139167880508 0.6727419884978436 0.3766386466414149 T T T

0.2996641358810484 0.0073606579088278 0.3808686692174990 T T T

0.3027735431542011 0.3418673392347811 0.3771428244799775 T T T

0.2968374404962966 0.6737216308693309 0.3792344230542594 T T T

0.4688275287413805 0.0086088240891805 0.3773692499635915 T T T

0.4639206287749295 0.3411361052930768 0.3792946059280132 T T T

0.4679106834942992 0.6750891626650681 0.3781023502911240 T T T

0.6305012104377635 0.0088789045133440 0.3790490859551952 T T T

0.6348140453124710 0.3419703989678214 0.3782113372440539 T T T

0.6298488235169135 0.6746788908510722 0.3789961891949409 T T T

0.7996285398499009 0.0079069504983169 0.3806843609298211 T T T

0.7978355457624654 0.3436448966175332 0.3791471958621528 T T T

0.7991317369229891 0.6737683988585804 0.3810955423458402 T T T

0.9688639555383306 0.0075932216910077 0.3774619278229061 T T T

0.9673691109727183 0.3426747218930659 0.3807934716842979 T T T

0.9673396678171872 0.6725926554346232 0.3791963516417043 T T T

0.1328962194276020 0.1727027556959017 0.3795405290469315 T T T

0.1334507194961094 0.5082451751394491 0.3805009323950838 T T T

0.1323999109045696 0.8376297332645298 0.3808086085072823 T T T

0.2990223356422798 0.1737847265155789 0.3802211707616340 T T T

0.3002586858808896 0.5060739844209116 0.3801212309625142 T T T

0.2984161088040755 0.8383904248779226 0.3798184011406704 T T T

0.4662505243032434 0.1726182677868092 0.3802870422771271 T T T

0.4663327419851676 0.5085603345783948 0.3796565045224619 T T T

0.4657177135526543 0.8410007379991428 0.3803865398223124 T T T

0.6331297590561277 0.1760209561817641 0.3796033171098491 T T T

0.6312232757131119 0.5062657071782075 0.3811593095224419 T T T

0.6314423203968157 0.8401450181255489 0.3808540717093897 T T T

0.7984918083563622 0.1736952457826908 0.3807780631618037 T T T

0.7981983769465654 0.5070226263321039 0.3810554075168452 T T T

0.7983575603681571 0.8388661137303602 0.3800221790118231 T T T

0.9661392321492476 0.1724933145456303 0.3813703591862717 T T T

0.9669404426101706 0.5092912272126798 0.3785052192180188 T T T

0.9663797867735000 0.8396848831242721 0.3805096998494794 T T T

0.0826462090432543 0.0913673776135795 0.2306510691320725 F F F

0.0826462090432543 0.4247007109469081 0.2306510691320725 F F F

0.0826462090432543 0.7580340442802367 0.2306510691320725 F F F

0.2493128757099186 0.0913673776135795 0.2306510691320725 F F F

0.2493128757099186 0.4247007109469081 0.2306510691320725 F F F

0.2493128757099186 0.7580340442802367 0.2306510691320725 F F F

0.4159795423765829 0.0913673776135795 0.2306510691320725 F F F

0.4159795423765829 0.4247007109469081 0.2306510691320725 F F F

0.4159795423765829 0.7580340442802367 0.2306510691320725 F F F

0.5826462090432472 0.0913673776135795 0.2306510691320725 F F F

0.5826462090432472 0.4247007109469081 0.2306510691320725 F F F

0.5826462090432472 0.7580340442802367 0.2306510691320725 F F F

0.7493128757099186 0.0913673776135795 0.2306510691320725 F F F

0.7493128757099186 0.4247007109469081 0.2306510691320725 F F F

0.7493128757099186 0.7580340442802367 0.2306510691320725 F F F

0.9159795423765758 0.0913673776135795 0.2306510691320725 F F F

0.9159795423765758 0.4247007109469081 0.2306510691320725 F F F

0.9159795423765758 0.7580340442802367 0.2306510691320725 F F F

0.0902677041989364 0.2625098265214945 0.2403988745878252 F F F

0.0902677041989364 0.5958431598548302 0.2403988745878252 F F F

0.0902677041989364 0.9291764931881659 0.2403988745878252 F F F

0.2569343708656078 0.2625098265214945 0.2403988745878252 F F F

0.2569343708656078 0.5958431598548302 0.2403988745878252 F F F

0.2569343708656078 0.9291764931881659 0.2403988745878252 F F F

0.4236010375322721 0.2625098265214945 0.2403988745878252 F F F

0.4236010375322721 0.5958431598548302 0.2403988745878252 F F F

0.4236010375322721 0.9291764931881659 0.2403988745878252 F F F

0.5902677041989364 0.2625098265214945 0.2403988745878252 F F F

0.5902677041989364 0.5958431598548302 0.2403988745878252 F F F

0.5902677041989364 0.9291764931881659 0.2403988745878252 F F F

0.7569343708656078 0.2625098265214945 0.2403988745878252 F F F

0.7569343708656078 0.5958431598548302 0.2403988745878252 F F F

0.7569343708656078 0.9291764931881659 0.2403988745878252 F F F

0.9236010375322721 0.2625098265214945 0.2403988745878252 F F F

0.9236010375322721 0.5958431598548302 0.2403988745878252 F F F

0.9236010375322721 0.9291764931881659 0.2403988745878252 F F F

0.1450268573747806 0.0574030619262587 0.1502611028726761 F F F

0.1450268573747806 0.3907363952595873 0.1502611028726761 F F F

0.1450268573747806 0.7240697285929230 0.1502611028726761 F F F

0.3116935240414520 0.0574030619262587 0.1502611028726761 F F F

0.3116935240414520 0.3907363952595873 0.1502611028726761 F F F

0.3116935240414520 0.7240697285929230 0.1502611028726761 F F F

0.4783601907081163 0.0574030619262587 0.1502611028726761 F F F

0.4783601907081163 0.3907363952595873 0.1502611028726761 F F F

0.4783601907081163 0.7240697285929230 0.1502611028726761 F F F

0.6450268573747806 0.0574030619262587 0.1502611028726761 F F F

0.6450268573747806 0.3907363952595873 0.1502611028726761 F F F

0.6450268573747806 0.7240697285929230 0.1502611028726761 F F F

0.8116935240414520 0.0574030619262587 0.1502611028726761 F F F

0.8116935240414520 0.3907363952595873 0.1502611028726761 F F F

0.8116935240414520 0.7240697285929230 0.1502611028726761 F F F

0.9783601907081163 0.0574030619262587 0.1502611028726761 F F F

0.9783601907081163 0.3907363952595873 0.1502611028726761 F F F

0.9783601907081163 0.7240697285929230 0.1502611028726761 F F F

0.1540633143111236 0.2313171148045612 0.1594044809199673 F F F

0.1540633143111236 0.5646504481378969 0.1594044809199673 F F F

0.1540633143111236 0.8979837814712326 0.1594044809199673 F F F

0.3207299809777879 0.2313171148045612 0.1594044809199673 F F F

0.3207299809777879 0.5646504481378969 0.1594044809199673 F F F

0.3207299809777879 0.8979837814712326 0.1594044809199673 F F F

0.4873966476444522 0.2313171148045612 0.1594044809199673 F F F

0.4873966476444522 0.5646504481378969 0.1594044809199673 F F F

0.4873966476444522 0.8979837814712326 0.1594044809199673 F F F

0.6540633143111236 0.2313171148045612 0.1594044809199673 F F F

0.6540633143111236 0.5646504481378969 0.1594044809199673 F F F

0.6540633143111236 0.8979837814712326 0.1594044809199673 F F F

0.8207299809777879 0.2313171148045612 0.1594044809199673 F F F

0.8207299809777879 0.5646504481378969 0.1594044809199673 F F F

0.8207299809777879 0.8979837814712326 0.1594044809199673 F F F

0.9873966476444522 0.2313171148045612 0.1594044809199673 F F F

0.9873966476444522 0.5646504481378969 0.1594044809199673 F F F

0.9873966476444522 0.8979837814712326 0.1594044809199673 F F F

0.0146512498045195 0.1016498357939755 0.4147402905317055 T T T

0.0150190743538125 0.4411842692366868 0.4148439718808269 T T T

0.0145928458946289 0.7674191348151075 0.4149597170485508 T T T

0.1800024205324205 0.1046941766292167 0.4139840720834950 T T T

0.1817084410817889 0.4370435947571767 0.4154484357863518 T T T

0.1788625097350737 0.7659891192728761 0.4146748728187716 T T T

0.3472556492782483 0.1033363868904953 0.4156050234033183 T T T

0.3481902230854870 0.4362809549766073 0.4139350366671006 T T T

0.3463116685284099 0.7709717538445728 0.4142101887210851 T T T

0.5144689747778547 0.1030684218049449 0.4141579084669925 T T T

0.5120176190638390 0.4382845424364374 0.4143999090175203 T T T

0.5126602846302414 0.7691656751536458 0.4150559719388203 T T T

0.6793654308799718 0.1067474058901439 0.4143723450544923 T T T

0.6794454969541444 0.4351739222315530 0.4155067058775676 T T T

0.6790739530140911 0.7712233322291938 0.4148798241309410 T T T

0.8471143479023132 0.1030248428930555 0.4157932694503861 T T T

0.8482294681042778 0.4407482334339189 0.4151262251606948 T T T

0.8476057379815957 0.7701570744584242 0.4152471956664786 T T T

0.0227634011776159 0.2772394743693810 0.4257578915153218 T T T

0.0214599079625077 0.6040391048095843 0.4167978017406463 T T T

0.0192276171684553 0.9514269768101645 0.4184474975137136 T T T

0.1965470003577151 0.2801541609124025 0.4226623053620522 T T T

0.1842204811365406 0.6167367944094819 0.4179674307292063 T T T

0.1864174402756834 0.9415844409538674 0.4251584089814253 T T T

0.3527272640391252 0.2859078568944914 0.4185295471918012 T T T

0.3605681497734735 0.6111544968500704 0.4229709679305795 T T T

0.3622833173503781 0.9461782440859132 0.4228371942037543 T T T

0.5277229107310616 0.2785208372206408 0.4231603884519442 T T T

0.5189435886325380 0.6192781875836794 0.4186408252466267 T T T

0.5192254129628707 0.9528500813887891 0.4185103648364859 T T T

0.6865158143460027 0.2868260928419487 0.4187268414833716 T T T

0.6841983456679582 0.6088084773474379 0.4247360145619968 T T T

0.6867429908021864 0.9436835343107146 0.4249017643800301 T T T

0.8549604180132832 0.2798484335987550 0.4239531414592274 T T T

0.8524459192089001 0.6074190703906459 0.4250891569261028 T T T

0.8623729186389434 0.9459624067602322 0.4228355399797867 T T T

0.0745309756803231 0.0722551866296920 0.3336959490792105 T T T

0.0778367193850022 0.4034937026109482 0.3371374336320007 T T T

0.0843225571180197 0.7365713659340053 0.3353127991959126 T T T

0.2434891502477942 0.0683678619623138 0.3372119499127247 T T T

0.2478679528326444 0.4017279475558346 0.3365186246512936 T T T

0.2425855264538039 0.7391387971007473 0.3341353399583372 T T T

0.4137429010488621 0.0682783711670068 0.3369617790813834 T T T

0.4084008450190790 0.4054368677878360 0.3335774120447332 T T T

0.4117951657955948 0.7334901215919390 0.3377201484514278 T T T

0.5744142640153660 0.0732843094617850 0.3337166387232835 T T T

0.5787719711603205 0.4006292855470384 0.3377863155163378 T T T

0.5736875571460399 0.7400635835263533 0.3342622753010782 T T T

0.7438467767723272 0.0704016382678703 0.3372702520495792 T T T

0.7406497016225095 0.4073494327081956 0.3343126222431974 T T T

0.7427234316548775 0.7361407142767338 0.3371943076103031 T T T

0.9140306541649889 0.0682573191785136 0.3372616488820038 T T T

0.9135034017450312 0.4119214373876339 0.3378198195964522 T T T

0.9126403570490790 0.7349008899980788 0.3375110912802535 T T T

0.0852145530005734 0.2432432832055910 0.3463032292107182 T T T

0.0835206178895642 0.5756573871765490 0.3421248306148143 T T T

0.0837582102636656 0.9071211431970283 0.3454821006443405 T T T

0.2512351002770440 0.2434152748034219 0.3434138259681017 T T T

0.2504415359053009 0.5742908429922235 0.3456361925509907 T T T

0.2514691326635548 0.9092189033577615 0.3464376096523716 T T T

0.4175503748134675 0.2416169188472067 0.3458834640750136 T T T

0.4181864795922735 0.5773760345449210 0.3435488875661615 T T T

0.4175469600514589 0.9100929315245863 0.3436210764744355 T T T

0.5848790356782734 0.2443399131881962 0.3437430922551764 T T T

0.5834033981036735 0.5756555121473642 0.3446980728597066 T T T

0.5837274379579425 0.9093625887433744 0.3454194820246596 T T T

0.7519202328203501 0.2448804206228536 0.3449444880330768 T T T

0.7504543330195185 0.5761548849868873 0.3461625726299570 T T T

0.7509841801913441 0.9100228239103807 0.3461521814738500 T T T

0.9185871953148642 0.2434943554658439 0.3464761471681650 T T T

0.9171681226169455 0.5756247235536953 0.3442832356910404 T T T

0.9178341441592800 0.9095106841166941 0.3437146748533811 T T T

0.1397131416109474 0.0545266729236502 0.1118484288951365 F F F

0.1397131416109474 0.3878600062569788 0.1118484288951365 F F F

0.1397131416109474 0.7211933395903145 0.1118484288951365 F F F

0.3063798082776117 0.0545266729236502 0.1118484288951365 F F F

0.3063798082776117 0.3878600062569788 0.1118484288951365 F F F

0.3063798082776117 0.7211933395903145 0.1118484288951365 F F F

0.4730464749442831 0.0545266729236502 0.1118484288951365 F F F

0.4730464749442831 0.3878600062569788 0.1118484288951365 F F F

0.4730464749442831 0.7211933395903145 0.1118484288951365 F F F

0.6397131416109474 0.0545266729236502 0.1118484288951365 F F F

0.6397131416109474 0.3878600062569788 0.1118484288951365 F F F

0.6397131416109474 0.7211933395903145 0.1118484288951365 F F F

0.8063798082776117 0.0545266729236502 0.1118484288951365 F F F

0.8063798082776117 0.3878600062569788 0.1118484288951365 F F F

0.8063798082776117 0.7211933395903145 0.1118484288951365 F F F

0.9730464749442831 0.0545266729236502 0.1118484288951365 F F F

0.9730464749442831 0.3878600062569788 0.1118484288951365 F F F

0.9730464749442831 0.7211933395903145 0.1118484288951365 F F F

0.0904969084302252 0.2563302902655593 0.2814745639703204 F F F

0.0904969084302252 0.5896636235988879 0.2814745639703204 F F F

0.0904969084302252 0.9229969569322236 0.2814745639703204 F F F

0.2571635750968966 0.2563302902655593 0.2814745639703204 F F F

0.2571635750968966 0.5896636235988879 0.2814745639703204 F F F

0.2571635750968966 0.9229969569322236 0.2814745639703204 F F F

0.4238302417635609 0.2563302902655593 0.2814745639703204 F F F

0.4238302417635609 0.5896636235988879 0.2814745639703204 F F F

0.4238302417635609 0.9229969569322236 0.2814745639703204 F F F

0.5904969084302252 0.2563302902655593 0.2814745639703204 F F F

0.5904969084302252 0.5896636235988879 0.2814745639703204 F F F

0.5904969084302252 0.9229969569322236 0.2814745639703204 F F F

0.7571635750968895 0.2563302902655593 0.2814745639703204 F F F

0.7571635750968895 0.5896636235988879 0.2814745639703204 F F F

0.7571635750968895 0.9229969569322236 0.2814745639703204 F F F

0.9238302417635609 0.2563302902655593 0.2814745639703204 F F F

0.9238302417635609 0.5896636235988879 0.2814745639703204 F F F

0.9238302417635609 0.9229969569322236 0.2814745639703204 F F F

0.0727072037349988 0.0772989003214236 0.2918994292816633 T T T

0.0771635774331946 0.4093270304552685 0.2959700329291731 T T T

0.0853682174758606 0.7426023394178668 0.2938381861426016 T T T

0.2439295429922678 0.0746588602828405 0.2960866080352119 T T T

0.2481479906770598 0.4076096188779703 0.2949438106083411 T T T

0.2413963700649183 0.7452527066539747 0.2924812747088418 T T T

0.4142728750795520 0.0741127689561496 0.2955013094856431 T T T

0.4064287608380950 0.4100948534358610 0.2918051996799756 T T T

0.4130713876903844 0.7393893716164389 0.2963212179957839 T T T

0.5725656148826472 0.0774624230505413 0.2920796682819850 T T T

0.5799474857534971 0.4064451703627699 0.2963736339568739 T T T

0.5719541922013086 0.7445260933547474 0.2926762160996111 T T T

0.7440812603522995 0.0763746736276863 0.2961751312711796 T T T

0.7389642395599807 0.4114972293966029 0.2926898080713282 T T T

0.7433636020744008 0.7425805260011069 0.2960542252081528 T T T

0.9144258838998603 0.0741377739995374 0.2958315497530581 T T T

0.9150388831748500 0.4211780801244720 0.2963126056028687 T T T

0.9129410841057490 0.7415865813779857 0.2963388938970801 T T T

0.0269995991542592 0.2776452554341214 0.4645107546916081 T T T

0.2100478243287735 0.2811735679821176 0.4604860675327274 T T T

0.1861145089665697 0.9403924213611948 0.4641998179512060 T T T

0.3710230400341953 0.6097271615372976 0.4613516926179378 T T T

0.3749551136789291 0.9464203737962913 0.4608290169982335 T T T

0.5385185281577944 0.2775507108538336 0.4614751530467801 T T T

0.6825018383260867 0.6054932547515898 0.4639795182262039 T T T

0.6865139525623742 0.9411767952808704 0.4640769672101560 T T T

0.8559612196593258 0.2809694512939741 0.4627287185477123 T T T

0.8547484999073123 0.6019874860781900 0.4643269605020405 T T T

0.8752602978539520 0.9462463534265088 0.4607911077995756 T T T

0.00000000E+00 0.00000000E+00 0.00000000E+00

0.00000000E+00 0.00000000E+00 0.00000000E+00

0.00000000E+00 0.00000000E+00 0.00000000E+00

0.00000000E+00 0.00000000E+00 0.00000000E+00

0.00000000E+00 0.00000000E+00 0.00000000E+00

0.00000000E+00 0.00000000E+00 0.00000000E+00

0.00000000E+00 0.00000000E+00 0.00000000E+00

0.00000000E+00 0.00000000E+00 0.00000000E+00

0.00000000E+00 0.00000000E+00 0.00000000E+00

0.00000000E+00 0.00000000E+00 0.00000000E+00

0.00000000E+00 0.00000000E+00 0.00000000E+00

0.00000000E+00 0.00000000E+00 0.00000000E+00

0.00000000E+00 0.00000000E+00 0.00000000E+00

0.00000000E+00 0.00000000E+00 0.00000000E+00

0.00000000E+00 0.00000000E+00 0.00000000E+00

0.00000000E+00 0.00000000E+00 0.00000000E+00

0.00000000E+00 0.00000000E+00 0.00000000E+00

0.00000000E+00 0.00000000E+00 0.00000000E+00

0.00000000E+00 0.00000000E+00 0.00000000E+00

0.00000000E+00 0.00000000E+00 0.00000000E+00

0.00000000E+00 0.00000000E+00 0.00000000E+00

0.00000000E+00 0.00000000E+00 0.00000000E+00

0.00000000E+00 0.00000000E+00 0.00000000E+00

0.00000000E+00 0.00000000E+00 0.00000000E+00

0.00000000E+00 0.00000000E+00 0.00000000E+00

0.00000000E+00 0.00000000E+00 0.00000000E+00

0.00000000E+00 0.00000000E+00 0.00000000E+00

0.00000000E+00 0.00000000E+00 0.00000000E+00

0.00000000E+00 0.00000000E+00 0.00000000E+00

0.00000000E+00 0.00000000E+00 0.00000000E+00

0.00000000E+00 0.00000000E+00 0.00000000E+00

0.00000000E+00 0.00000000E+00 0.00000000E+00

0.00000000E+00 0.00000000E+00 0.00000000E+00

0.00000000E+00 0.00000000E+00 0.00000000E+00

0.00000000E+00 0.00000000E+00 0.00000000E+00

0.00000000E+00 0.00000000E+00 0.00000000E+00

0.00000000E+00 0.00000000E+00 0.00000000E+00

0.00000000E+00 0.00000000E+00 0.00000000E+00

0.00000000E+00 0.00000000E+00 0.00000000E+00

0.00000000E+00 0.00000000E+00 0.00000000E+00

0.00000000E+00 0.00000000E+00 0.00000000E+00

0.00000000E+00 0.00000000E+00 0.00000000E+00

0.00000000E+00 0.00000000E+00 0.00000000E+00

0.00000000E+00 0.00000000E+00 0.00000000E+00

0.00000000E+00 0.00000000E+00 0.00000000E+00

0.00000000E+00 0.00000000E+00 0.00000000E+00

0.00000000E+00 0.00000000E+00 0.00000000E+00

0.00000000E+00 0.00000000E+00 0.00000000E+00

0.00000000E+00 0.00000000E+00 0.00000000E+00

0.00000000E+00 0.00000000E+00 0.00000000E+00

0.00000000E+00 0.00000000E+00 0.00000000E+00

0.00000000E+00 0.00000000E+00 0.00000000E+00

0.00000000E+00 0.00000000E+00 0.00000000E+00

0.00000000E+00 0.00000000E+00 0.00000000E+00

0.00000000E+00 0.00000000E+00 0.00000000E+00

0.00000000E+00 0.00000000E+00 0.00000000E+00

0.00000000E+00 0.00000000E+00 0.00000000E+00

0.00000000E+00 0.00000000E+00 0.00000000E+00

0.00000000E+00 0.00000000E+00 0.00000000E+00

0.00000000E+00 0.00000000E+00 0.00000000E+00

0.00000000E+00 0.00000000E+00 0.00000000E+00

0.00000000E+00 0.00000000E+00 0.00000000E+00

0.00000000E+00 0.00000000E+00 0.00000000E+00

0.00000000E+00 0.00000000E+00 0.00000000E+00

0.00000000E+00 0.00000000E+00 0.00000000E+00

0.00000000E+00 0.00000000E+00 0.00000000E+00

0.00000000E+00 0.00000000E+00 0.00000000E+00

0.00000000E+00 0.00000000E+00 0.00000000E+00

0.00000000E+00 0.00000000E+00 0.00000000E+00

0.00000000E+00 0.00000000E+00 0.00000000E+00

0.00000000E+00 0.00000000E+00 0.00000000E+00

0.00000000E+00 0.00000000E+00 0.00000000E+00

0.00000000E+00 0.00000000E+00 0.00000000E+00

0.00000000E+00 0.00000000E+00 0.00000000E+00

0.00000000E+00 0.00000000E+00 0.00000000E+00

0.00000000E+00 0.00000000E+00 0.00000000E+00

0.00000000E+00 0.00000000E+00 0.00000000E+00

0.00000000E+00 0.00000000E+00 0.00000000E+00

0.00000000E+00 0.00000000E+00 0.00000000E+00

0.00000000E+00 0.00000000E+00 0.00000000E+00

0.00000000E+00 0.00000000E+00 0.00000000E+00

0.00000000E+00 0.00000000E+00 0.00000000E+00

0.00000000E+00 0.00000000E+00 0.00000000E+00

0.00000000E+00 0.00000000E+00 0.00000000E+00

0.00000000E+00 0.00000000E+00 0.00000000E+00

0.00000000E+00 0.00000000E+00 0.00000000E+00

0.00000000E+00 0.00000000E+00 0.00000000E+00

0.00000000E+00 0.00000000E+00 0.00000000E+00

0.00000000E+00 0.00000000E+00 0.00000000E+00

0.00000000E+00 0.00000000E+00 0.00000000E+00

0.00000000E+00 0.00000000E+00 0.00000000E+00

0.00000000E+00 0.00000000E+00 0.00000000E+00

0.00000000E+00 0.00000000E+00 0.00000000E+00

0.00000000E+00 0.00000000E+00 0.00000000E+00

0.00000000E+00 0.00000000E+00 0.00000000E+00

0.00000000E+00 0.00000000E+00 0.00000000E+00

0.00000000E+00 0.00000000E+00 0.00000000E+00

0.00000000E+00 0.00000000E+00 0.00000000E+00

0.00000000E+00 0.00000000E+00 0.00000000E+00

0.00000000E+00 0.00000000E+00 0.00000000E+00

0.00000000E+00 0.00000000E+00 0.00000000E+00

0.00000000E+00 0.00000000E+00 0.00000000E+00

0.00000000E+00 0.00000000E+00 0.00000000E+00

0.00000000E+00 0.00000000E+00 0.00000000E+00

0.00000000E+00 0.00000000E+00 0.00000000E+00

0.00000000E+00 0.00000000E+00 0.00000000E+00

0.00000000E+00 0.00000000E+00 0.00000000E+00

0.00000000E+00 0.00000000E+00 0.00000000E+00

0.00000000E+00 0.00000000E+00 0.00000000E+00

0.00000000E+00 0.00000000E+00 0.00000000E+00

0.00000000E+00 0.00000000E+00 0.00000000E+00

0.00000000E+00 0.00000000E+00 0.00000000E+00

0.00000000E+00 0.00000000E+00 0.00000000E+00

0.00000000E+00 0.00000000E+00 0.00000000E+00

0.00000000E+00 0.00000000E+00 0.00000000E+00

0.00000000E+00 0.00000000E+00 0.00000000E+00

0.00000000E+00 0.00000000E+00 0.00000000E+00

0.00000000E+00 0.00000000E+00 0.00000000E+00

0.00000000E+00 0.00000000E+00 0.00000000E+00

0.00000000E+00 0.00000000E+00 0.00000000E+00

0.00000000E+00 0.00000000E+00 0.00000000E+00

0.00000000E+00 0.00000000E+00 0.00000000E+00

0.00000000E+00 0.00000000E+00 0.00000000E+00

0.00000000E+00 0.00000000E+00 0.00000000E+00

0.00000000E+00 0.00000000E+00 0.00000000E+00

0.00000000E+00 0.00000000E+00 0.00000000E+00

0.00000000E+00 0.00000000E+00 0.00000000E+00

0.00000000E+00 0.00000000E+00 0.00000000E+00

0.00000000E+00 0.00000000E+00 0.00000000E+00

0.00000000E+00 0.00000000E+00 0.00000000E+00

0.00000000E+00 0.00000000E+00 0.00000000E+00

0.00000000E+00 0.00000000E+00 0.00000000E+00

0.00000000E+00 0.00000000E+00 0.00000000E+00

0.00000000E+00 0.00000000E+00 0.00000000E+00

0.00000000E+00 0.00000000E+00 0.00000000E+00

0.00000000E+00 0.00000000E+00 0.00000000E+00

0.00000000E+00 0.00000000E+00 0.00000000E+00

0.00000000E+00 0.00000000E+00 0.00000000E+00

0.00000000E+00 0.00000000E+00 0.00000000E+00

0.00000000E+00 0.00000000E+00 0.00000000E+00

0.00000000E+00 0.00000000E+00 0.00000000E+00

0.00000000E+00 0.00000000E+00 0.00000000E+00

0.00000000E+00 0.00000000E+00 0.00000000E+00

0.00000000E+00 0.00000000E+00 0.00000000E+00

0.00000000E+00 0.00000000E+00 0.00000000E+00

0.00000000E+00 0.00000000E+00 0.00000000E+00

0.00000000E+00 0.00000000E+00 0.00000000E+00

0.00000000E+00 0.00000000E+00 0.00000000E+00

0.00000000E+00 0.00000000E+00 0.00000000E+00

0.00000000E+00 0.00000000E+00 0.00000000E+00

0.00000000E+00 0.00000000E+00 0.00000000E+00

0.00000000E+00 0.00000000E+00 0.00000000E+00

0.00000000E+00 0.00000000E+00 0.00000000E+00

0.00000000E+00 0.00000000E+00 0.00000000E+00

0.00000000E+00 0.00000000E+00 0.00000000E+00

0.00000000E+00 0.00000000E+00 0.00000000E+00

0.00000000E+00 0.00000000E+00 0.00000000E+00

0.00000000E+00 0.00000000E+00 0.00000000E+00

0.00000000E+00 0.00000000E+00 0.00000000E+00

0.00000000E+00 0.00000000E+00 0.00000000E+00

0.00000000E+00 0.00000000E+00 0.00000000E+00

0.00000000E+00 0.00000000E+00 0.00000000E+00

0.00000000E+00 0.00000000E+00 0.00000000E+00

0.00000000E+00 0.00000000E+00 0.00000000E+00

0.00000000E+00 0.00000000E+00 0.00000000E+00

0.00000000E+00 0.00000000E+00 0.00000000E+00

0.00000000E+00 0.00000000E+00 0.00000000E+00

0.00000000E+00 0.00000000E+00 0.00000000E+00

0.00000000E+00 0.00000000E+00 0.00000000E+00

0.00000000E+00 0.00000000E+00 0.00000000E+00

0.00000000E+00 0.00000000E+00 0.00000000E+00

0.00000000E+00 0.00000000E+00 0.00000000E+00

0.00000000E+00 0.00000000E+00 0.00000000E+00

0.00000000E+00 0.00000000E+00 0.00000000E+00

0.00000000E+00 0.00000000E+00 0.00000000E+00

0.00000000E+00 0.00000000E+00 0.00000000E+00

0.00000000E+00 0.00000000E+00 0.00000000E+00

0.00000000E+00 0.00000000E+00 0.00000000E+00

0.00000000E+00 0.00000000E+00 0.00000000E+00

0.00000000E+00 0.00000000E+00 0.00000000E+00

0.00000000E+00 0.00000000E+00 0.00000000E+00

0.00000000E+00 0.00000000E+00 0.00000000E+00

0.00000000E+00 0.00000000E+00 0.00000000E+00

0.00000000E+00 0.00000000E+00 0.00000000E+00

0.00000000E+00 0.00000000E+00 0.00000000E+00

0.00000000E+00 0.00000000E+00 0.00000000E+00

0.00000000E+00 0.00000000E+00 0.00000000E+00

0.00000000E+00 0.00000000E+00 0.00000000E+00

0.00000000E+00 0.00000000E+00 0.00000000E+00

0.00000000E+00 0.00000000E+00 0.00000000E+00

0.00000000E+00 0.00000000E+00 0.00000000E+00

0.00000000E+00 0.00000000E+00 0.00000000E+00

0.00000000E+00 0.00000000E+00 0.00000000E+00

0.00000000E+00 0.00000000E+00 0.00000000E+00

0.00000000E+00 0.00000000E+00 0.00000000E+00

0.00000000E+00 0.00000000E+00 0.00000000E+00

0.00000000E+00 0.00000000E+00 0.00000000E+00

0.00000000E+00 0.00000000E+00 0.00000000E+00

0.00000000E+00 0.00000000E+00 0.00000000E+00

0.00000000E+00 0.00000000E+00 0.00000000E+00

0.00000000E+00 0.00000000E+00 0.00000000E+00

0.00000000E+00 0.00000000E+00 0.00000000E+00

0.00000000E+00 0.00000000E+00 0.00000000E+00

0.00000000E+00 0.00000000E+00 0.00000000E+00

0.00000000E+00 0.00000000E+00 0.00000000E+00

0.00000000E+00 0.00000000E+00 0.00000000E+00

0.00000000E+00 0.00000000E+00 0.00000000E+00

0.00000000E+00 0.00000000E+00 0.00000000E+00

0.00000000E+00 0.00000000E+00 0.00000000E+00

0.00000000E+00 0.00000000E+00 0.00000000E+00

0.00000000E+00 0.00000000E+00 0.00000000E+00

0.00000000E+00 0.00000000E+00 0.00000000E+00

0.00000000E+00 0.00000000E+00 0.00000000E+00

0.00000000E+00 0.00000000E+00 0.00000000E+00

0.00000000E+00 0.00000000E+00 0.00000000E+00

0.00000000E+00 0.00000000E+00 0.00000000E+00

0.00000000E+00 0.00000000E+00 0.00000000E+00

0.00000000E+00 0.00000000E+00 0.00000000E+00

0.00000000E+00 0.00000000E+00 0.00000000E+00

0.00000000E+00 0.00000000E+00 0.00000000E+00

0.00000000E+00 0.00000000E+00 0.00000000E+00

0.00000000E+00 0.00000000E+00 0.00000000E+00

0.00000000E+00 0.00000000E+00 0.00000000E+00

0.00000000E+00 0.00000000E+00 0.00000000E+00

0.00000000E+00 0.00000000E+00 0.00000000E+00

0.00000000E+00 0.00000000E+00 0.00000000E+00

0.00000000E+00 0.00000000E+00 0.00000000E+00

0.00000000E+00 0.00000000E+00 0.00000000E+00

0.00000000E+00 0.00000000E+00 0.00000000E+00

0.00000000E+00 0.00000000E+00 0.00000000E+00

0.00000000E+00 0.00000000E+00 0.00000000E+00

0.00000000E+00 0.00000000E+00 0.00000000E+00

0.00000000E+00 0.00000000E+00 0.00000000E+00

0.00000000E+00 0.00000000E+00 0.00000000E+00

0.00000000E+00 0.00000000E+00 0.00000000E+00

0.00000000E+00 0.00000000E+00 0.00000000E+00

0.00000000E+00 0.00000000E+00 0.00000000E+00

0.00000000E+00 0.00000000E+00 0.00000000E+00

0.00000000E+00 0.00000000E+00 0.00000000E+00

0.00000000E+00 0.00000000E+00 0.00000000E+00

0.00000000E+00 0.00000000E+00 0.00000000E+00

0.00000000E+00 0.00000000E+00 0.00000000E+00

0.00000000E+00 0.00000000E+00 0.00000000E+00

0.00000000E+00 0.00000000E+00 0.00000000E+00

0.00000000E+00 0.00000000E+00 0.00000000E+00

0.00000000E+00 0.00000000E+00 0.00000000E+00

0.00000000E+00 0.00000000E+00 0.00000000E+00

0.00000000E+00 0.00000000E+00 0.00000000E+00

0.00000000E+00 0.00000000E+00 0.00000000E+00

0.00000000E+00 0.00000000E+00 0.00000000E+00

0.00000000E+00 0.00000000E+00 0.00000000E+00

0.00000000E+00 0.00000000E+00 0.00000000E+00

0.00000000E+00 0.00000000E+00 0.00000000E+00

0.00000000E+00 0.00000000E+00 0.00000000E+00

0.00000000E+00 0.00000000E+00 0.00000000E+00

0.00000000E+00 0.00000000E+00 0.00000000E+00

0.00000000E+00 0.00000000E+00 0.00000000E+00

0.00000000E+00 0.00000000E+00 0.00000000E+00

0.00000000E+00 0.00000000E+00 0.00000000E+00

0.00000000E+00 0.00000000E+00 0.00000000E+00

0.00000000E+00 0.00000000E+00 0.00000000E+00

0.00000000E+00 0.00000000E+00 0.00000000E+00

0.00000000E+00 0.00000000E+00 0.00000000E+00

0.00000000E+00 0.00000000E+00 0.00000000E+00

0.00000000E+00 0.00000000E+00 0.00000000E+00

0.00000000E+00 0.00000000E+00 0.00000000E+00

0.00000000E+00 0.00000000E+00 0.00000000E+00

0.00000000E+00 0.00000000E+00 0.00000000E+00

0.00000000E+00 0.00000000E+00 0.00000000E+00

0.00000000E+00 0.00000000E+00 0.00000000E+00

0.00000000E+00 0.00000000E+00 0.00000000E+00

0.00000000E+00 0.00000000E+00 0.00000000E+00

0.00000000E+00 0.00000000E+00 0.00000000E+00

0.00000000E+00 0.00000000E+00 0.00000000E+00

0.00000000E+00 0.00000000E+00 0.00000000E+00

0.00000000E+00 0.00000000E+00 0.00000000E+00

0.00000000E+00 0.00000000E+00 0.00000000E+00

0.00000000E+00 0.00000000E+00 0.00000000E+00

0.00000000E+00 0.00000000E+00 0.00000000E+00

0.00000000E+00 0.00000000E+00 0.00000000E+00

0.00000000E+00 0.00000000E+00 0.00000000E+00

NiOOH_9H ML CONTCAR

This file is generated by VASPKIT code

1.00000000000000

17.5560007098000028 0.0000000000000000 0.0000000000000000

-8.7889406573999977 15.4086551811000021 0.0000000000000000

0.0000000000000015 4.9977513337000028 24.4953563273999961

Ni O H

72 144 63

Selective dynamics

Direct

0.0341653786323661 0.3260019713917757 0.1960457408563343 F F F

0.0341653786323661 0.6593353047251114 0.1960457408563343 F F F

0.0341653786323661 0.9926686380584400 0.1960457408563343 F F F

0.2008320452990304 0.3260019713917757 0.1960457408563343 F F F

0.2008320452990304 0.6593353047251114 0.1960457408563343 F F F

0.2008320452990304 0.9926686380584400 0.1960457408563343 F F F

0.3674987119657018 0.3260019713917757 0.1960457408563343 F F F

0.3674987119657018 0.6593353047251114 0.1960457408563343 F F F

0.3674987119657018 0.9926686380584400 0.1960457408563343 F F F

0.5341653786323661 0.3260019713917757 0.1960457408563343 F F F

0.5341653786323661 0.6593353047251114 0.1960457408563343 F F F

0.5341653786323661 0.9926686380584400 0.1960457408563343 F F F

0.7008320452990304 0.3260019713917757 0.1960457408563343 F F F

0.7008320452990304 0.6593353047251114 0.1960457408563343 F F F

0.7008320452990304 0.9926686380584400 0.1960457408563343 F F F

0.8674987119657018 0.3260019713917757 0.1960457408563343 F F F

0.8674987119657018 0.6593353047251114 0.1960457408563343 F F F

0.8674987119657018 0.9926686380584400 0.1960457408563343 F F F

0.0351970056773183 0.1617665099525851 0.1949035160852901 F F F

0.0351970056773183 0.4950998432859137 0.1949035160852901 F F F

0.0351970056773183 0.8284331766192494 0.1949035160852901 F F F

0.2018636723439897 0.1617665099525851 0.1949035160852901 F F F

0.2018636723439897 0.4950998432859137 0.1949035160852901 F F F

0.2018636723439897 0.8284331766192494 0.1949035160852901 F F F

0.3685303390106540 0.1617665099525851 0.1949035160852901 F F F

0.3685303390106540 0.4950998432859137 0.1949035160852901 F F F

0.3685303390106540 0.8284331766192494 0.1949035160852901 F F F

0.5351970056773183 0.1617665099525851 0.1949035160852901 F F F

0.5351970056773183 0.4950998432859137 0.1949035160852901 F F F

0.5351970056773183 0.8284331766192494 0.1949035160852901 F F F

0.7018636723439897 0.1617665099525851 0.1949035160852901 F F F

0.7018636723439897 0.4950998432859137 0.1949035160852901 F F F

0.7018636723439897 0.8284331766192494 0.1949035160852901 F F F

0.8685303390106540 0.1617665099525851 0.1949035160852901 F F F

0.8685303390106540 0.4950998432859137 0.1949035160852901 F F F

0.8685303390106540 0.8284331766192494 0.1949035160852901 F F F

0.1304233639789720 0.0037816994995475 0.3792502896813248 T T T

0.1318208640930161 0.3399915011198366 0.3790254625620781 T T T

0.1338011306186236 0.6712634065155845 0.3796676814554285 T T T

0.3018812376779277 0.0068447708182139 0.3779895645407753 T T T

0.3017801445713194 0.3402108577040046 0.3778111275400154 T T T

0.2970656515082780 0.6707938857333755 0.3791535045202432 T T T

0.4639365660826169 0.0070571132711577 0.3791745060168029 T T T

0.4637226356240510 0.3411560995931771 0.3793638797324996 T T T

0.4683975282149602 0.6743826949492481 0.3779283069380028 T T T

0.6342772879856213 0.0083475302315004 0.3781783885737477 T T T

0.6353157508398667 0.3429333854700560 0.3781609335750942 T T T

0.6299806346754970 0.6740822298426392 0.3800891825414062 T T T

0.7968372345815905 0.0090036784233503 0.3797368487246557 T T T

0.7968599811874345 0.3408472921587619 0.3805671474925940 T T T

0.8001536159677480 0.6745131324668387 0.3800102397674155 T T T

0.9673251452688886 0.0062918240374821 0.3801262453944873 T T T

0.9671004324908117 0.3400580430554567 0.3803376150353733 T T T

0.9646131541663368 0.6741450366845029 0.3790150802951449 T T T

0.1319568719112326 0.1710461578659490 0.3798891078588301 T T T

0.1343664030344497 0.5071841896601269 0.3790424190440370 T T T

0.1321705030701112 0.8367481924654653 0.3804604862906394 T T T

0.2986159805347940 0.1724586779795769 0.3807347114356541 T T T

0.2993531253550895 0.5045057542798307 0.3802268652509824 T T T

0.2989169270675856 0.8384991767614863 0.3801719965552285 T T T

0.4656736513597056 0.1731333650946018 0.3795710919308536 T T T

0.4657927197430500 0.5078006447078184 0.3798847760473960 T T T

0.4649278988241482 0.8378823655105511 0.3806394612572249 T T T

0.6319788807565768 0.1747790764669788 0.3801210123907027 T T T

0.6315226110168881 0.5072351105957563 0.3811259306923724 T T T

0.6323941499863134 0.8416902010199215 0.3800232906028802 T T T

0.7975921974550002 0.1742734598634246 0.3810977546934802 T T T

0.7991848853827356 0.5091912930716790 0.3789789379714224 T T T

0.7992282490001487 0.8401540549458362 0.3811750869092110 T T T

0.9651115106238165 0.1738853378483189 0.3796901087796585 T T T

0.9659208957347030 0.5058808312406151 0.3811123193671312 T T T

0.9675288478113716 0.8415145801362865 0.3792095265520892 T T T

0.0826462090432543 0.0913673776135795 0.2306510691320725 F F F

0.0826462090432543 0.4247007109469081 0.2306510691320725 F F F

0.0826462090432543 0.7580340442802367 0.2306510691320725 F F F

0.2493128757099186 0.0913673776135795 0.2306510691320725 F F F

0.2493128757099186 0.4247007109469081 0.2306510691320725 F F F

0.2493128757099186 0.7580340442802367 0.2306510691320725 F F F

0.4159795423765829 0.0913673776135795 0.2306510691320725 F F F

0.4159795423765829 0.4247007109469081 0.2306510691320725 F F F

0.4159795423765829 0.7580340442802367 0.2306510691320725 F F F

0.5826462090432472 0.0913673776135795 0.2306510691320725 F F F

0.5826462090432472 0.4247007109469081 0.2306510691320725 F F F

0.5826462090432472 0.7580340442802367 0.2306510691320725 F F F

0.7493128757099186 0.0913673776135795 0.2306510691320725 F F F

0.7493128757099186 0.4247007109469081 0.2306510691320725 F F F

0.7493128757099186 0.7580340442802367 0.2306510691320725 F F F

0.9159795423765758 0.0913673776135795 0.2306510691320725 F F F

0.9159795423765758 0.4247007109469081 0.2306510691320725 F F F

0.9159795423765758 0.7580340442802367 0.2306510691320725 F F F

0.0902677041989364 0.2625098265214945 0.2403988745878252 F F F

0.0902677041989364 0.5958431598548302 0.2403988745878252 F F F

0.0902677041989364 0.9291764931881659 0.2403988745878252 F F F

0.2569343708656078 0.2625098265214945 0.2403988745878252 F F F

0.2569343708656078 0.5958431598548302 0.2403988745878252 F F F

0.2569343708656078 0.9291764931881659 0.2403988745878252 F F F

0.4236010375322721 0.2625098265214945 0.2403988745878252 F F F

0.4236010375322721 0.5958431598548302 0.2403988745878252 F F F

0.4236010375322721 0.9291764931881659 0.2403988745878252 F F F

0.5902677041989364 0.2625098265214945 0.2403988745878252 F F F

0.5902677041989364 0.5958431598548302 0.2403988745878252 F F F

0.5902677041989364 0.9291764931881659 0.2403988745878252 F F F

0.7569343708656078 0.2625098265214945 0.2403988745878252 F F F

0.7569343708656078 0.5958431598548302 0.2403988745878252 F F F

0.7569343708656078 0.9291764931881659 0.2403988745878252 F F F

0.9236010375322721 0.2625098265214945 0.2403988745878252 F F F

0.9236010375322721 0.5958431598548302 0.2403988745878252 F F F

0.9236010375322721 0.9291764931881659 0.2403988745878252 F F F

0.1450268573747806 0.0574030619262587 0.1502611028726761 F F F

0.1450268573747806 0.3907363952595873 0.1502611028726761 F F F

0.1450268573747806 0.7240697285929230 0.1502611028726761 F F F

0.3116935240414520 0.0574030619262587 0.1502611028726761 F F F

0.3116935240414520 0.3907363952595873 0.1502611028726761 F F F

0.3116935240414520 0.7240697285929230 0.1502611028726761 F F F

0.4783601907081163 0.0574030619262587 0.1502611028726761 F F F

0.4783601907081163 0.3907363952595873 0.1502611028726761 F F F

0.4783601907081163 0.7240697285929230 0.1502611028726761 F F F

0.6450268573747806 0.0574030619262587 0.1502611028726761 F F F

0.6450268573747806 0.3907363952595873 0.1502611028726761 F F F

0.6450268573747806 0.7240697285929230 0.1502611028726761 F F F

0.8116935240414520 0.0574030619262587 0.1502611028726761 F F F

0.8116935240414520 0.3907363952595873 0.1502611028726761 F F F

0.8116935240414520 0.7240697285929230 0.1502611028726761 F F F

0.9783601907081163 0.0574030619262587 0.1502611028726761 F F F

0.9783601907081163 0.3907363952595873 0.1502611028726761 F F F

0.9783601907081163 0.7240697285929230 0.1502611028726761 F F F

0.1540633143111236 0.2313171148045612 0.1594044809199673 F F F

0.1540633143111236 0.5646504481378969 0.1594044809199673 F F F

0.1540633143111236 0.8979837814712326 0.1594044809199673 F F F

0.3207299809777879 0.2313171148045612 0.1594044809199673 F F F

0.3207299809777879 0.5646504481378969 0.1594044809199673 F F F

0.3207299809777879 0.8979837814712326 0.1594044809199673 F F F

0.4873966476444522 0.2313171148045612 0.1594044809199673 F F F

0.4873966476444522 0.5646504481378969 0.1594044809199673 F F F

0.4873966476444522 0.8979837814712326 0.1594044809199673 F F F

0.6540633143111236 0.2313171148045612 0.1594044809199673 F F F

0.6540633143111236 0.5646504481378969 0.1594044809199673 F F F

0.6540633143111236 0.8979837814712326 0.1594044809199673 F F F

0.8207299809777879 0.2313171148045612 0.1594044809199673 F F F

0.8207299809777879 0.5646504481378969 0.1594044809199673 F F F

0.8207299809777879 0.8979837814712326 0.1594044809199673 F F F

0.9873966476444522 0.2313171148045612 0.1594044809199673 F F F

0.9873966476444522 0.5646504481378969 0.1594044809199673 F F F

0.9873966476444522 0.8979837814712326 0.1594044809199673 F F F

0.0135039684751137 0.1033211949482975 0.4153447464187527 T T T

0.0183334241730414 0.4378154360581173 0.4156692811101816 T T T

0.0125325229266487 0.7714658870634594 0.4154670333771650 T T T

0.1784445652003655 0.1017319349065340 0.4148424398372857 T T T

0.1799154639973095 0.4373164898338763 0.4150658414820770 T T T

0.1804357808841598 0.7667997128958268 0.4151046203075611 T T T

0.3473303053647693 0.1018920846414131 0.4147498807792996 T T T

0.3474039720864597 0.4350122913361329 0.4143696356080713 T T T

0.3450447123270101 0.7682543152911269 0.4154028586727690 T T T

0.5132797586761075 0.1052089790625805 0.4136815039953151 T T T

0.5121291976865030 0.4382679480302738 0.4144107260325793 T T T

0.5135047105848162 0.7685361061476457 0.4146616607891717 T T T

0.6789672864719031 0.1028970722587658 0.4149225642359531 T T T

0.6815655388186187 0.4379192055292659 0.4148382998917842 T T T

0.6792003761970115 0.7726762204828598 0.4148582545928555 T T T

0.8459681684965369 0.1063129103695887 0.4153087171528339 T T T

0.8457876030641454 0.4401246184492276 0.4146703295288467 T T T

0.8512007164141965 0.7719318636655762 0.4156700211834796 T T T

0.0176146363547808 0.2732926803884454 0.4177272152511715 T T T

0.0192985675660442 0.6069538535356823 0.4236242854014650 T T T

0.0181162744371842 0.9323852555845155 0.4194932250233732 T T T

0.1946619851111905 0.2779833442580239 0.4225515639383526 T T T

0.1843939741471150 0.5982393251066194 0.4192677991834763 T T T

0.1952158450222335 0.9432002498664801 0.4226746552448716 T T T

0.3524167296246063 0.2849338579219309 0.4189751071940527 T T T

0.3612149677798155 0.6100515163329044 0.4225205690516668 T T T

0.3532210595681669 0.9518777798633716 0.4187750992033701 T T T

0.5289300387428020 0.2797829192483717 0.4228826311785965 T T T

0.5188769410221981 0.6187485023408397 0.4188798964821230 T T T

0.5272757717898011 0.9447704923700432 0.4231484685437009 T T T

0.6854214006183343 0.2866883095945900 0.4188929990046616 T T T

0.6818904003832680 0.6059497537930647 0.4254644198783447 T T T

0.6857073050549745 0.9529816594098468 0.4189517444902559 T T T

0.8480560282969112 0.2731055982790311 0.4257570801153833 T T T

0.8507686062545525 0.6076071015227870 0.4173605019270104 T T T

0.8546548877406667 0.9436039023111956 0.4235905301609939 T T T

0.0773697306716510 0.0712185483269029 0.3354112848361475 T T T

0.0870473166580247 0.4159711201914163 0.3356861603654864 T T T

0.0763480913709689 0.7348657614447355 0.3374100953152943 T T T

0.2457114662233053 0.0659533603554807 0.3377135735132459 T T T

0.2464486848830768 0.3998638707005693 0.3372823813289841 T T T

0.2455024399437750 0.7376513635284792 0.3351764546458920 T T T

0.4078645080922147 0.0720702676220189 0.3339085549172319 T T T

0.4075540439284054 0.4045605996828184 0.3339522722119864 T T T

0.4123748171987877 0.7334769586905910 0.3374671770765350 T T T

0.5786228871472807 0.0668241966551048 0.3375030166915761 T T T

0.5791474284038854 0.4014025387296350 0.3376593037682122 T T T

0.5745467003720129 0.7394695541506884 0.3342751455282586 T T T

0.7403274258086416 0.0734562273550229 0.3344461868577637 T T T

0.7439970559803780 0.4113164117555112 0.3353119120117083 T T T

0.7443974889699113 0.7348066913179616 0.3389321462635375 T T T

0.9121312752926937 0.0761617653524486 0.3385406092208148 T T T

0.9112839799554355 0.4005656803335117 0.3391770032304186 T T T

0.9195190930052961 0.7509187129287993 0.3357976896228748 T T T

0.0840408169956558 0.2412494161032613 0.3449267473336876 T T T

0.0848349336150924 0.5739875634850820 0.3444994820852942 T T T

0.0842123087986208 0.9064174190584411 0.3446312157778499 T T T

0.2509137786486694 0.2417319080095551 0.3439391329452867 T T T

0.2509344387397921 0.5730851311504410 0.3444033248418955 T T T

0.2513728567620802 0.9082379611338689 0.3440531330297427 T T T

0.4172640759233582 0.2419626026014182 0.3459486918402874 T T T

0.4179526263707000 0.5762051820044825 0.3435965057810024 T T T

0.4173121127925754 0.9074788509173446 0.3459032349761162 T T T

0.5849841105382453 0.2451070652483968 0.3434779955056856 T T T

0.5832216007041096 0.5756449884847850 0.3449112346429527 T T T

0.5843011989750553 0.9101465538934153 0.3440988636655428 T T T

0.7498679961847058 0.2431771699386417 0.3453536672110369 T T T

0.7507581767432394 0.5773646600895440 0.3444550646908116 T T T

0.7511578259318258 0.9102947861847758 0.3452516222737945 T T T

0.9169464333466099 0.2427848791563912 0.3448997286426857 T T T

0.9162907715750866 0.5751625123536096 0.3440770074465387 T T T

0.9186034398359640 0.9093248510064686 0.3446417371253841 T T T

0.1397131416109474 0.0545266729236502 0.1118484288951365 F F F

0.1397131416109474 0.3878600062569788 0.1118484288951365 F F F

0.1397131416109474 0.7211933395903145 0.1118484288951365 F F F

0.3063798082776117 0.0545266729236502 0.1118484288951365 F F F

0.3063798082776117 0.3878600062569788 0.1118484288951365 F F F

0.3063798082776117 0.7211933395903145 0.1118484288951365 F F F

0.4730464749442831 0.0545266729236502 0.1118484288951365 F F F

0.4730464749442831 0.3878600062569788 0.1118484288951365 F F F

0.4730464749442831 0.7211933395903145 0.1118484288951365 F F F

0.6397131416109474 0.0545266729236502 0.1118484288951365 F F F

0.6397131416109474 0.3878600062569788 0.1118484288951365 F F F

0.6397131416109474 0.7211933395903145 0.1118484288951365 F F F

0.8063798082776117 0.0545266729236502 0.1118484288951365 F F F

0.8063798082776117 0.3878600062569788 0.1118484288951365 F F F

0.8063798082776117 0.7211933395903145 0.1118484288951365 F F F

0.9730464749442831 0.0545266729236502 0.1118484288951365 F F F

0.9730464749442831 0.3878600062569788 0.1118484288951365 F F F

0.9730464749442831 0.7211933395903145 0.1118484288951365 F F F

0.0904969084302252 0.2563302902655593 0.2814745639703204 F F F

0.0904969084302252 0.5896636235988879 0.2814745639703204 F F F

0.0904969084302252 0.9229969569322236 0.2814745639703204 F F F

0.2571635750968966 0.2563302902655593 0.2814745639703204 F F F

0.2571635750968966 0.5896636235988879 0.2814745639703204 F F F

0.2571635750968966 0.9229969569322236 0.2814745639703204 F F F

0.4238302417635609 0.2563302902655593 0.2814745639703204 F F F

0.4238302417635609 0.5896636235988879 0.2814745639703204 F F F

0.4238302417635609 0.9229969569322236 0.2814745639703204 F F F

0.5904969084302252 0.2563302902655593 0.2814745639703204 F F F

0.5904969084302252 0.5896636235988879 0.2814745639703204 F F F

0.5904969084302252 0.9229969569322236 0.2814745639703204 F F F

0.7571635750968895 0.2563302902655593 0.2814745639703204 F F F

0.7571635750968895 0.5896636235988879 0.2814745639703204 F F F

0.7571635750968895 0.9229969569322236 0.2814745639703204 F F F

0.9238302417635609 0.2563302902655593 0.2814745639703204 F F F

0.9238302417635609 0.5896636235988879 0.2814745639703204 F F F

0.9238302417635609 0.9229969569322236 0.2814745639703204 F F F

0.0765663088911083 0.0780126625142508 0.2941488246182017 T T T

0.0889299111230404 0.4267947217815283 0.2943105432990781 T T T

0.0765700309078171 0.7429980213814551 0.2961446736962538 T T T

0.2470803054483777 0.0727365356507399 0.2962532679282230 T T T

0.2473950257193092 0.4067608853607579 0.2956595152590618 T T T

0.2449941024086087 0.7447566225801053 0.2940034087673170 T T T

0.4060629852443115 0.0771100269113432 0.2921982159942512 T T T

0.4058770829946147 0.4094424315335941 0.2922208308199981 T T T

0.4136016629812534 0.7401473700752338 0.2959673182099284 T T T

0.5798610998917710 0.0724247013102225 0.2960779716231229 T T T

0.5802049269899734 0.4068664823983960 0.2962487215326531 T T T

0.5727543888803562 0.7440055585954223 0.2927016609269926 T T T

0.7386181533340992 0.0776059062733854 0.2928957730287867 T T T

0.7431880499549439 0.4180515954983606 0.2932278351536315 T T T

0.7455735134535860 0.7408779028290654 0.2977645688652705 T T T

0.9136804372848845 0.0858554649349609 0.2970843726677531 T T T

0.9125115758776526 0.4068344104773732 0.2979802873082795 T T T

0.9218252061680958 0.7617671347405005 0.2944901860569087 T T T

0.0197647431427098 0.5991404068293760 0.4630828998042349 T T T

0.2047486724629863 0.2761995927905148 0.4610154607274499 T T T

0.2048873002129816 0.9414911365344335 0.4611860696141616 T T T

0.3702991784447212 0.6084606295109946 0.4611022901373976 T T T

0.5399935298806180 0.2765050368125339 0.4612506126459859 T T T

0.5377439104103798 0.9442842234952360 0.4615040610676516 T T T

0.6777193489621132 0.5963368721549706 0.4650783173455508 T T T

0.8431114756368212 0.2623761793333097 0.4654120247481986 T T T

0.8580350051739308 0.9382797520469001 0.4628183786411083 T T T

0.00000000E+00 0.00000000E+00 0.00000000E+00

0.00000000E+00 0.00000000E+00 0.00000000E+00

0.00000000E+00 0.00000000E+00 0.00000000E+00

0.00000000E+00 0.00000000E+00 0.00000000E+00

0.00000000E+00 0.00000000E+00 0.00000000E+00

0.00000000E+00 0.00000000E+00 0.00000000E+00

0.00000000E+00 0.00000000E+00 0.00000000E+00

0.00000000E+00 0.00000000E+00 0.00000000E+00

0.00000000E+00 0.00000000E+00 0.00000000E+00

0.00000000E+00 0.00000000E+00 0.00000000E+00

0.00000000E+00 0.00000000E+00 0.00000000E+00

0.00000000E+00 0.00000000E+00 0.00000000E+00

0.00000000E+00 0.00000000E+00 0.00000000E+00

0.00000000E+00 0.00000000E+00 0.00000000E+00

0.00000000E+00 0.00000000E+00 0.00000000E+00

0.00000000E+00 0.00000000E+00 0.00000000E+00

0.00000000E+00 0.00000000E+00 0.00000000E+00

0.00000000E+00 0.00000000E+00 0.00000000E+00

0.00000000E+00 0.00000000E+00 0.00000000E+00

0.00000000E+00 0.00000000E+00 0.00000000E+00

0.00000000E+00 0.00000000E+00 0.00000000E+00

0.00000000E+00 0.00000000E+00 0.00000000E+00

0.00000000E+00 0.00000000E+00 0.00000000E+00

0.00000000E+00 0.00000000E+00 0.00000000E+00

0.00000000E+00 0.00000000E+00 0.00000000E+00

0.00000000E+00 0.00000000E+00 0.00000000E+00

0.00000000E+00 0.00000000E+00 0.00000000E+00

0.00000000E+00 0.00000000E+00 0.00000000E+00

0.00000000E+00 0.00000000E+00 0.00000000E+00

0.00000000E+00 0.00000000E+00 0.00000000E+00

0.00000000E+00 0.00000000E+00 0.00000000E+00

0.00000000E+00 0.00000000E+00 0.00000000E+00

0.00000000E+00 0.00000000E+00 0.00000000E+00

0.00000000E+00 0.00000000E+00 0.00000000E+00

0.00000000E+00 0.00000000E+00 0.00000000E+00

0.00000000E+00 0.00000000E+00 0.00000000E+00

0.00000000E+00 0.00000000E+00 0.00000000E+00

0.00000000E+00 0.00000000E+00 0.00000000E+00

0.00000000E+00 0.00000000E+00 0.00000000E+00

0.00000000E+00 0.00000000E+00 0.00000000E+00

0.00000000E+00 0.00000000E+00 0.00000000E+00

0.00000000E+00 0.00000000E+00 0.00000000E+00

0.00000000E+00 0.00000000E+00 0.00000000E+00

0.00000000E+00 0.00000000E+00 0.00000000E+00

0.00000000E+00 0.00000000E+00 0.00000000E+00

0.00000000E+00 0.00000000E+00 0.00000000E+00

0.00000000E+00 0.00000000E+00 0.00000000E+00

0.00000000E+00 0.00000000E+00 0.00000000E+00

0.00000000E+00 0.00000000E+00 0.00000000E+00

0.00000000E+00 0.00000000E+00 0.00000000E+00

0.00000000E+00 0.00000000E+00 0.00000000E+00

0.00000000E+00 0.00000000E+00 0.00000000E+00

0.00000000E+00 0.00000000E+00 0.00000000E+00

0.00000000E+00 0.00000000E+00 0.00000000E+00

0.00000000E+00 0.00000000E+00 0.00000000E+00

0.00000000E+00 0.00000000E+00 0.00000000E+00

0.00000000E+00 0.00000000E+00 0.00000000E+00

0.00000000E+00 0.00000000E+00 0.00000000E+00

0.00000000E+00 0.00000000E+00 0.00000000E+00

0.00000000E+00 0.00000000E+00 0.00000000E+00

0.00000000E+00 0.00000000E+00 0.00000000E+00

0.00000000E+00 0.00000000E+00 0.00000000E+00

0.00000000E+00 0.00000000E+00 0.00000000E+00

0.00000000E+00 0.00000000E+00 0.00000000E+00

0.00000000E+00 0.00000000E+00 0.00000000E+00

0.00000000E+00 0.00000000E+00 0.00000000E+00

0.00000000E+00 0.00000000E+00 0.00000000E+00

0.00000000E+00 0.00000000E+00 0.00000000E+00

0.00000000E+00 0.00000000E+00 0.00000000E+00

0.00000000E+00 0.00000000E+00 0.00000000E+00

0.00000000E+00 0.00000000E+00 0.00000000E+00

0.00000000E+00 0.00000000E+00 0.00000000E+00

0.00000000E+00 0.00000000E+00 0.00000000E+00

0.00000000E+00 0.00000000E+00 0.00000000E+00

0.00000000E+00 0.00000000E+00 0.00000000E+00

0.00000000E+00 0.00000000E+00 0.00000000E+00

0.00000000E+00 0.00000000E+00 0.00000000E+00

0.00000000E+00 0.00000000E+00 0.00000000E+00

0.00000000E+00 0.00000000E+00 0.00000000E+00

0.00000000E+00 0.00000000E+00 0.00000000E+00

0.00000000E+00 0.00000000E+00 0.00000000E+00

0.00000000E+00 0.00000000E+00 0.00000000E+00

0.00000000E+00 0.00000000E+00 0.00000000E+00

0.00000000E+00 0.00000000E+00 0.00000000E+00

0.00000000E+00 0.00000000E+00 0.00000000E+00

0.00000000E+00 0.00000000E+00 0.00000000E+00

0.00000000E+00 0.00000000E+00 0.00000000E+00

0.00000000E+00 0.00000000E+00 0.00000000E+00

0.00000000E+00 0.00000000E+00 0.00000000E+00

0.00000000E+00 0.00000000E+00 0.00000000E+00

0.00000000E+00 0.00000000E+00 0.00000000E+00

0.00000000E+00 0.00000000E+00 0.00000000E+00

0.00000000E+00 0.00000000E+00 0.00000000E+00

0.00000000E+00 0.00000000E+00 0.00000000E+00

0.00000000E+00 0.00000000E+00 0.00000000E+00

0.00000000E+00 0.00000000E+00 0.00000000E+00

0.00000000E+00 0.00000000E+00 0.00000000E+00

0.00000000E+00 0.00000000E+00 0.00000000E+00

0.00000000E+00 0.00000000E+00 0.00000000E+00

0.00000000E+00 0.00000000E+00 0.00000000E+00

0.00000000E+00 0.00000000E+00 0.00000000E+00

0.00000000E+00 0.00000000E+00 0.00000000E+00

0.00000000E+00 0.00000000E+00 0.00000000E+00

0.00000000E+00 0.00000000E+00 0.00000000E+00

0.00000000E+00 0.00000000E+00 0.00000000E+00

0.00000000E+00 0.00000000E+00 0.00000000E+00

0.00000000E+00 0.00000000E+00 0.00000000E+00

0.00000000E+00 0.00000000E+00 0.00000000E+00

0.00000000E+00 0.00000000E+00 0.00000000E+00

0.00000000E+00 0.00000000E+00 0.00000000E+00

0.00000000E+00 0.00000000E+00 0.00000000E+00

0.00000000E+00 0.00000000E+00 0.00000000E+00

0.00000000E+00 0.00000000E+00 0.00000000E+00

0.00000000E+00 0.00000000E+00 0.00000000E+00

0.00000000E+00 0.00000000E+00 0.00000000E+00

0.00000000E+00 0.00000000E+00 0.00000000E+00

0.00000000E+00 0.00000000E+00 0.00000000E+00

0.00000000E+00 0.00000000E+00 0.00000000E+00

0.00000000E+00 0.00000000E+00 0.00000000E+00

0.00000000E+00 0.00000000E+00 0.00000000E+00

0.00000000E+00 0.00000000E+00 0.00000000E+00

0.00000000E+00 0.00000000E+00 0.00000000E+00

0.00000000E+00 0.00000000E+00 0.00000000E+00

0.00000000E+00 0.00000000E+00 0.00000000E+00

0.00000000E+00 0.00000000E+00 0.00000000E+00

0.00000000E+00 0.00000000E+00 0.00000000E+00

0.00000000E+00 0.00000000E+00 0.00000000E+00

0.00000000E+00 0.00000000E+00 0.00000000E+00

0.00000000E+00 0.00000000E+00 0.00000000E+00

0.00000000E+00 0.00000000E+00 0.00000000E+00

0.00000000E+00 0.00000000E+00 0.00000000E+00

0.00000000E+00 0.00000000E+00 0.00000000E+00

0.00000000E+00 0.00000000E+00 0.00000000E+00

0.00000000E+00 0.00000000E+00 0.00000000E+00

0.00000000E+00 0.00000000E+00 0.00000000E+00

0.00000000E+00 0.00000000E+00 0.00000000E+00

0.00000000E+00 0.00000000E+00 0.00000000E+00

0.00000000E+00 0.00000000E+00 0.00000000E+00

0.00000000E+00 0.00000000E+00 0.00000000E+00

0.00000000E+00 0.00000000E+00 0.00000000E+00

0.00000000E+00 0.00000000E+00 0.00000000E+00

0.00000000E+00 0.00000000E+00 0.00000000E+00

0.00000000E+00 0.00000000E+00 0.00000000E+00

0.00000000E+00 0.00000000E+00 0.00000000E+00

0.00000000E+00 0.00000000E+00 0.00000000E+00

0.00000000E+00 0.00000000E+00 0.00000000E+00

0.00000000E+00 0.00000000E+00 0.00000000E+00

0.00000000E+00 0.00000000E+00 0.00000000E+00

0.00000000E+00 0.00000000E+00 0.00000000E+00

0.00000000E+00 0.00000000E+00 0.00000000E+00

0.00000000E+00 0.00000000E+00 0.00000000E+00

0.00000000E+00 0.00000000E+00 0.00000000E+00

0.00000000E+00 0.00000000E+00 0.00000000E+00

0.00000000E+00 0.00000000E+00 0.00000000E+00

0.00000000E+00 0.00000000E+00 0.00000000E+00

0.00000000E+00 0.00000000E+00 0.00000000E+00

0.00000000E+00 0.00000000E+00 0.00000000E+00

0.00000000E+00 0.00000000E+00 0.00000000E+00

0.00000000E+00 0.00000000E+00 0.00000000E+00

0.00000000E+00 0.00000000E+00 0.00000000E+00

0.00000000E+00 0.00000000E+00 0.00000000E+00

0.00000000E+00 0.00000000E+00 0.00000000E+00

0.00000000E+00 0.00000000E+00 0.00000000E+00

0.00000000E+00 0.00000000E+00 0.00000000E+00

0.00000000E+00 0.00000000E+00 0.00000000E+00

0.00000000E+00 0.00000000E+00 0.00000000E+00

0.00000000E+00 0.00000000E+00 0.00000000E+00

0.00000000E+00 0.00000000E+00 0.00000000E+00

0.00000000E+00 0.00000000E+00 0.00000000E+00

0.00000000E+00 0.00000000E+00 0.00000000E+00

0.00000000E+00 0.00000000E+00 0.00000000E+00

0.00000000E+00 0.00000000E+00 0.00000000E+00

0.00000000E+00 0.00000000E+00 0.00000000E+00

0.00000000E+00 0.00000000E+00 0.00000000E+00

0.00000000E+00 0.00000000E+00 0.00000000E+00

0.00000000E+00 0.00000000E+00 0.00000000E+00

0.00000000E+00 0.00000000E+00 0.00000000E+00

0.00000000E+00 0.00000000E+00 0.00000000E+00

0.00000000E+00 0.00000000E+00 0.00000000E+00

0.00000000E+00 0.00000000E+00 0.00000000E+00

0.00000000E+00 0.00000000E+00 0.00000000E+00

0.00000000E+00 0.00000000E+00 0.00000000E+00

0.00000000E+00 0.00000000E+00 0.00000000E+00

0.00000000E+00 0.00000000E+00 0.00000000E+00

0.00000000E+00 0.00000000E+00 0.00000000E+00

0.00000000E+00 0.00000000E+00 0.00000000E+00

0.00000000E+00 0.00000000E+00 0.00000000E+00

0.00000000E+00 0.00000000E+00 0.00000000E+00

0.00000000E+00 0.00000000E+00 0.00000000E+00

0.00000000E+00 0.00000000E+00 0.00000000E+00

0.00000000E+00 0.00000000E+00 0.00000000E+00

0.00000000E+00 0.00000000E+00 0.00000000E+00

0.00000000E+00 0.00000000E+00 0.00000000E+00

0.00000000E+00 0.00000000E+00 0.00000000E+00

0.00000000E+00 0.00000000E+00 0.00000000E+00

0.00000000E+00 0.00000000E+00 0.00000000E+00

0.00000000E+00 0.00000000E+00 0.00000000E+00

0.00000000E+00 0.00000000E+00 0.00000000E+00

0.00000000E+00 0.00000000E+00 0.00000000E+00

0.00000000E+00 0.00000000E+00 0.00000000E+00

0.00000000E+00 0.00000000E+00 0.00000000E+00

0.00000000E+00 0.00000000E+00 0.00000000E+00

0.00000000E+00 0.00000000E+00 0.00000000E+00

0.00000000E+00 0.00000000E+00 0.00000000E+00

0.00000000E+00 0.00000000E+00 0.00000000E+00

0.00000000E+00 0.00000000E+00 0.00000000E+00

0.00000000E+00 0.00000000E+00 0.00000000E+00

0.00000000E+00 0.00000000E+00 0.00000000E+00

0.00000000E+00 0.00000000E+00 0.00000000E+00

0.00000000E+00 0.00000000E+00 0.00000000E+00

0.00000000E+00 0.00000000E+00 0.00000000E+00

0.00000000E+00 0.00000000E+00 0.00000000E+00

0.00000000E+00 0.00000000E+00 0.00000000E+00

0.00000000E+00 0.00000000E+00 0.00000000E+00

0.00000000E+00 0.00000000E+00 0.00000000E+00

0.00000000E+00 0.00000000E+00 0.00000000E+00

0.00000000E+00 0.00000000E+00 0.00000000E+00

0.00000000E+00 0.00000000E+00 0.00000000E+00

0.00000000E+00 0.00000000E+00 0.00000000E+00

0.00000000E+00 0.00000000E+00 0.00000000E+00

0.00000000E+00 0.00000000E+00 0.00000000E+00

0.00000000E+00 0.00000000E+00 0.00000000E+00

0.00000000E+00 0.00000000E+00 0.00000000E+00

0.00000000E+00 0.00000000E+00 0.00000000E+00

0.00000000E+00 0.00000000E+00 0.00000000E+00

0.00000000E+00 0.00000000E+00 0.00000000E+00

0.00000000E+00 0.00000000E+00 0.00000000E+00

0.00000000E+00 0.00000000E+00 0.00000000E+00

0.00000000E+00 0.00000000E+00 0.00000000E+00

0.00000000E+00 0.00000000E+00 0.00000000E+00

0.00000000E+00 0.00000000E+00 0.00000000E+00

0.00000000E+00 0.00000000E+00 0.00000000E+00

0.00000000E+00 0.00000000E+00 0.00000000E+00

0.00000000E+00 0.00000000E+00 0.00000000E+00

0.00000000E+00 0.00000000E+00 0.00000000E+00

0.00000000E+00 0.00000000E+00 0.00000000E+00

0.00000000E+00 0.00000000E+00 0.00000000E+00

0.00000000E+00 0.00000000E+00 0.00000000E+00

0.00000000E+00 0.00000000E+00 0.00000000E+00

0.00000000E+00 0.00000000E+00 0.00000000E+00

0.00000000E+00 0.00000000E+00 0.00000000E+00

0.00000000E+00 0.00000000E+00 0.00000000E+00

0.00000000E+00 0.00000000E+00 0.00000000E+00

0.00000000E+00 0.00000000E+00 0.00000000E+00

0.00000000E+00 0.00000000E+00 0.00000000E+00

0.00000000E+00 0.00000000E+00 0.00000000E+00

0.00000000E+00 0.00000000E+00 0.00000000E+00

0.00000000E+00 0.00000000E+00 0.00000000E+00

0.00000000E+00 0.00000000E+00 0.00000000E+00

0.00000000E+00 0.00000000E+00 0.00000000E+00

0.00000000E+00 0.00000000E+00 0.00000000E+00

0.00000000E+00 0.00000000E+00 0.00000000E+00

0.00000000E+00 0.00000000E+00 0.00000000E+00

0.00000000E+00 0.00000000E+00 0.00000000E+00

0.00000000E+00 0.00000000E+00 0.00000000E+00

0.00000000E+00 0.00000000E+00 0.00000000E+00

0.00000000E+00 0.00000000E+00 0.00000000E+00

0.00000000E+00 0.00000000E+00 0.00000000E+00

0.00000000E+00 0.00000000E+00 0.00000000E+00

0.00000000E+00 0.00000000E+00 0.00000000E+00

0.00000000E+00 0.00000000E+00 0.00000000E+00

0.00000000E+00 0.00000000E+00 0.00000000E+00

0.00000000E+00 0.00000000E+00 0.00000000E+00

0.00000000E+00 0.00000000E+00 0.00000000E+00

0.00000000E+00 0.00000000E+00 0.00000000E+00

0.00000000E+00 0.00000000E+00 0.00000000E+00

0.00000000E+00 0.00000000E+00 0.00000000E+00

0.00000000E+00 0.00000000E+00 0.00000000E+00

0.00000000E+00 0.00000000E+00 0.00000000E+00

0.00000000E+00 0.00000000E+00 0.00000000E+00

0.00000000E+00 0.00000000E+00 0.00000000E+00

0.00000000E+00 0.00000000E+00 0.00000000E+00

0.00000000E+00 0.00000000E+00 0.00000000E+00

0.00000000E+00 0.00000000E+00 0.00000000E+00

0.00000000E+00 0.00000000E+00 0.00000000E+00

0.00000000E+00 0.00000000E+00 0.00000000E+00

0.00000000E+00 0.00000000E+00 0.00000000E+00

0.00000000E+00 0.00000000E+00 0.00000000E+00

0.00000000E+00 0.00000000E+00 0.00000000E+00

NiOOH_6H ML CONTCAR

This file is generated by VASPKIT code

1.00000000000000

17.5560007098000028 0.0000000000000000 0.0000000000000000

-8.7889406573999977 15.4086551811000021 0.0000000000000000

0.0000000000000015 4.9977513337000028 24.4953563273999961

Ni O H

72 144 60

Selective dynamics

Direct

0.0341653786323661 0.3260019713917757 0.1960457408563343 F F F

0.0341653786323661 0.6593353047251114 0.1960457408563343 F F F

0.0341653786323661 0.9926686380584400 0.1960457408563343 F F F

0.2008320452990304 0.3260019713917757 0.1960457408563343 F F F

0.2008320452990304 0.6593353047251114 0.1960457408563343 F F F

0.2008320452990304 0.9926686380584400 0.1960457408563343 F F F

0.3674987119657018 0.3260019713917757 0.1960457408563343 F F F

0.3674987119657018 0.6593353047251114 0.1960457408563343 F F F

0.3674987119657018 0.9926686380584400 0.1960457408563343 F F F

0.5341653786323661 0.3260019713917757 0.1960457408563343 F F F

0.5341653786323661 0.6593353047251114 0.1960457408563343 F F F

0.5341653786323661 0.9926686380584400 0.1960457408563343 F F F

0.7008320452990304 0.3260019713917757 0.1960457408563343 F F F

0.7008320452990304 0.6593353047251114 0.1960457408563343 F F F

0.7008320452990304 0.9926686380584400 0.1960457408563343 F F F

0.8674987119657018 0.3260019713917757 0.1960457408563343 F F F

0.8674987119657018 0.6593353047251114 0.1960457408563343 F F F

0.8674987119657018 0.9926686380584400 0.1960457408563343 F F F

0.0351970056773183 0.1617665099525851 0.1949035160852901 F F F

0.0351970056773183 0.4950998432859137 0.1949035160852901 F F F

0.0351970056773183 0.8284331766192494 0.1949035160852901 F F F

0.2018636723439897 0.1617665099525851 0.1949035160852901 F F F

0.2018636723439897 0.4950998432859137 0.1949035160852901 F F F

0.2018636723439897 0.8284331766192494 0.1949035160852901 F F F

0.3685303390106540 0.1617665099525851 0.1949035160852901 F F F

0.3685303390106540 0.4950998432859137 0.1949035160852901 F F F

0.3685303390106540 0.8284331766192494 0.1949035160852901 F F F

0.5351970056773183 0.1617665099525851 0.1949035160852901 F F F

0.5351970056773183 0.4950998432859137 0.1949035160852901 F F F

0.5351970056773183 0.8284331766192494 0.1949035160852901 F F F

0.7018636723439897 0.1617665099525851 0.1949035160852901 F F F

0.7018636723439897 0.4950998432859137 0.1949035160852901 F F F

0.7018636723439897 0.8284331766192494 0.1949035160852901 F F F

0.8685303390106540 0.1617665099525851 0.1949035160852901 F F F

0.8685303390106540 0.4950998432859137 0.1949035160852901 F F F

0.8685303390106540 0.8284331766192494 0.1949035160852901 F F F

0.1302613793178158 0.0043607458860006 0.3794654159516198 T T T

0.1294423489732341 0.3389022081926093 0.3808537223445274 T T T

0.1360012589278594 0.6733300728639322 0.3809987709092964 T T T

0.3024498163870841 0.0068109587822898 0.3794480442956606 T T T

0.3002718137559789 0.3386636554156022 0.3811619233615536 T T T

0.3012254852418783 0.6752983594020803 0.3767397455707741 T T T

0.4688932938276483 0.0089757848382054 0.3764388527585853 T T T

0.4675049433823051 0.3424216080016503 0.3772146639667686 T T T

0.4623277812285299 0.6730541179134667 0.3764747311075970 T T T

0.6317584830159945 0.0091417815182680 0.3768557119843021 T T T

0.6319771483112980 0.3439042208420099 0.3771194908909322 T T T

0.6273698042984219 0.6723042614906908 0.3799237506946099 T T T

0.7945999394658831 0.0068064420208921 0.3794077895483075 T T T

0.7960841457120438 0.3424713217633422 0.3801642471165277 T T T

0.7989321687449967 0.6737861089838167 0.3794173563226964 T T T

0.9650649251966941 0.0041176410683753 0.3780500258371631 T T T

0.9673667868830893 0.3410988787139607 0.3784581409660910 T T T

0.9641789243945527 0.6732715051949987 0.3801881760594508 T T T

0.1306846868797555 0.1709706056473074 0.3816105838454024 T T T

0.1325603063584043 0.5078478036764225 0.3804470439821111 T T T

0.1326581795771091 0.8387860235216380 0.3819593694732615 T T T

0.3000919338104340 0.1735466977317335 0.3805724246019952 T T T

0.2980010151419649 0.5073427315963276 0.3801409487108082 T T T

0.3028455260182801 0.8420490942590544 0.3790059308762090 T T T

0.4677586120310093 0.1755934241911521 0.3790417976204576 T T T

0.4645497304535747 0.5081819228699176 0.3784305136843927 T T T

0.4672357564809286 0.8420906420789119 0.3796506454845514 T T T

0.6328021957154791 0.1763739977229979 0.3800939683625751 T T T

0.6310223164793022 0.5077685745905945 0.3805681580362800 T T T

0.6297820806270804 0.8399685702862005 0.3802804352924108 T T T

0.7980417857575736 0.1737047465728361 0.3795007213440484 T T T

0.7982429544729415 0.5094284478018851 0.3785333189841107 T T T

0.7964958360226136 0.8374203650295540 0.3808556023720331 T T T

0.9644648288704214 0.1726058391504406 0.3809855377821417 T T T

0.9652029016064395 0.5068188913951348 0.3814801934886747 T T T

0.9651757248439335 0.8409649304779854 0.3783527869879325 T T T

0.0826462090432543 0.0913673776135795 0.2306510691320725 F F F

0.0826462090432543 0.4247007109469081 0.2306510691320725 F F F

0.0826462090432543 0.7580340442802367 0.2306510691320725 F F F

0.2493128757099186 0.0913673776135795 0.2306510691320725 F F F

0.2493128757099186 0.4247007109469081 0.2306510691320725 F F F

0.2493128757099186 0.7580340442802367 0.2306510691320725 F F F

0.4159795423765829 0.0913673776135795 0.2306510691320725 F F F

0.4159795423765829 0.4247007109469081 0.2306510691320725 F F F

0.4159795423765829 0.7580340442802367 0.2306510691320725 F F F

0.5826462090432472 0.0913673776135795 0.2306510691320725 F F F

0.5826462090432472 0.4247007109469081 0.2306510691320725 F F F

0.5826462090432472 0.7580340442802367 0.2306510691320725 F F F

0.7493128757099186 0.0913673776135795 0.2306510691320725 F F F

0.7493128757099186 0.4247007109469081 0.2306510691320725 F F F

0.7493128757099186 0.7580340442802367 0.2306510691320725 F F F

0.9159795423765758 0.0913673776135795 0.2306510691320725 F F F

0.9159795423765758 0.4247007109469081 0.2306510691320725 F F F

0.9159795423765758 0.7580340442802367 0.2306510691320725 F F F

0.0902677041989364 0.2625098265214945 0.2403988745878252 F F F

0.0902677041989364 0.5958431598548302 0.2403988745878252 F F F

0.0902677041989364 0.9291764931881659 0.2403988745878252 F F F

0.2569343708656078 0.2625098265214945 0.2403988745878252 F F F

0.2569343708656078 0.5958431598548302 0.2403988745878252 F F F

0.2569343708656078 0.9291764931881659 0.2403988745878252 F F F

0.4236010375322721 0.2625098265214945 0.2403988745878252 F F F

0.4236010375322721 0.5958431598548302 0.2403988745878252 F F F

0.4236010375322721 0.9291764931881659 0.2403988745878252 F F F

0.5902677041989364 0.2625098265214945 0.2403988745878252 F F F

0.5902677041989364 0.5958431598548302 0.2403988745878252 F F F

0.5902677041989364 0.9291764931881659 0.2403988745878252 F F F

0.7569343708656078 0.2625098265214945 0.2403988745878252 F F F

0.7569343708656078 0.5958431598548302 0.2403988745878252 F F F

0.7569343708656078 0.9291764931881659 0.2403988745878252 F F F

0.9236010375322721 0.2625098265214945 0.2403988745878252 F F F

0.9236010375322721 0.5958431598548302 0.2403988745878252 F F F

0.9236010375322721 0.9291764931881659 0.2403988745878252 F F F

0.1450268573747806 0.0574030619262587 0.1502611028726761 F F F

0.1450268573747806 0.3907363952595873 0.1502611028726761 F F F

0.1450268573747806 0.7240697285929230 0.1502611028726761 F F F

0.3116935240414520 0.0574030619262587 0.1502611028726761 F F F

0.3116935240414520 0.3907363952595873 0.1502611028726761 F F F

0.3116935240414520 0.7240697285929230 0.1502611028726761 F F F

0.4783601907081163 0.0574030619262587 0.1502611028726761 F F F

0.4783601907081163 0.3907363952595873 0.1502611028726761 F F F

0.4783601907081163 0.7240697285929230 0.1502611028726761 F F F

0.6450268573747806 0.0574030619262587 0.1502611028726761 F F F

0.6450268573747806 0.3907363952595873 0.1502611028726761 F F F

0.6450268573747806 0.7240697285929230 0.1502611028726761 F F F

0.8116935240414520 0.0574030619262587 0.1502611028726761 F F F

0.8116935240414520 0.3907363952595873 0.1502611028726761 F F F

0.8116935240414520 0.7240697285929230 0.1502611028726761 F F F

0.9783601907081163 0.0574030619262587 0.1502611028726761 F F F

0.9783601907081163 0.3907363952595873 0.1502611028726761 F F F

0.9783601907081163 0.7240697285929230 0.1502611028726761 F F F

0.1540633143111236 0.2313171148045612 0.1594044809199673 F F F

0.1540633143111236 0.5646504481378969 0.1594044809199673 F F F

0.1540633143111236 0.8979837814712326 0.1594044809199673 F F F

0.3207299809777879 0.2313171148045612 0.1594044809199673 F F F

0.3207299809777879 0.5646504481378969 0.1594044809199673 F F F

0.3207299809777879 0.8979837814712326 0.1594044809199673 F F F

0.4873966476444522 0.2313171148045612 0.1594044809199673 F F F

0.4873966476444522 0.5646504481378969 0.1594044809199673 F F F

0.4873966476444522 0.8979837814712326 0.1594044809199673 F F F

0.6540633143111236 0.2313171148045612 0.1594044809199673 F F F

0.6540633143111236 0.5646504481378969 0.1594044809199673 F F F

0.6540633143111236 0.8979837814712326 0.1594044809199673 F F F

0.8207299809777879 0.2313171148045612 0.1594044809199673 F F F

0.8207299809777879 0.5646504481378969 0.1594044809199673 F F F

0.8207299809777879 0.8979837814712326 0.1594044809199673 F F F

0.9873966476444522 0.2313171148045612 0.1594044809199673 F F F

0.9873966476444522 0.5646504481378969 0.1594044809199673 F F F

0.9873966476444522 0.8979837814712326 0.1594044809199673 F F F

0.0114250704338781 0.0978285561426159 0.4153175615873557 T T T

0.0134716753079408 0.4353732301366542 0.4152996449645404 T T T

0.0103699480530531 0.7713726252507007 0.4157110436643776 T T T

0.1783872443237970 0.1007547121463185 0.4144878425600607 T T T

0.1795516305107058 0.4387410173057275 0.4145016842487949 T T T

0.1866892508878546 0.7726505983920315 0.4153402028871603 T T T

0.3511135914249941 0.1048032450943158 0.4137796115107800 T T T

0.3502033091330360 0.4395277914544125 0.4144747836858286 T T T

0.3504806572731118 0.7716195751854902 0.4131923579837443 T T T

0.5142862681362653 0.1040266341233062 0.4132804182300687 T T T

0.5129582374587167 0.4381513187752577 0.4140397334308101 T T T

0.5115230840132252 0.7680274693770887 0.4134872983698856 T T T

0.6793623967026755 0.1043626994054770 0.4135916814709280 T T T

0.6801425148673204 0.4390535077708568 0.4139654209122076 T T T

0.6754982353570540 0.7681776864787524 0.4153187797084250 T T T

0.8452589589169368 0.1043617436520006 0.4140460698122344 T T T

0.8445923100425540 0.4406992513728988 0.4146096688179756 T T T

0.8497298490084493 0.7716413629944966 0.4149743235573685 T T T

0.0171265027639018 0.2843964906010774 0.4192749030631260 T T T

0.0156349596072644 0.6054142403150571 0.4258105016596093 T T T

0.0095490230282855 0.9371541630529210 0.4170255511983662 T T T

0.1814572080074447 0.2702094823173025 0.4264203402797005 T T T

0.1949323780831356 0.6192158358253004 0.4185165635851853 T T T

0.1826470435060938 0.9392288125626274 0.4247073804810549 T T T

0.3603649667409549 0.2844431087066145 0.4184405999748959 T T T

0.3531468189757581 0.6178914836218315 0.4170912350900172 T T T

0.3600022298656302 0.9466067666408261 0.4170839893130031 T T T

0.5222027904414333 0.2874282419639804 0.4175517786893665 T T T

0.5053945695712314 0.6056495519533640 0.4168640452082166 T T T

0.5218130932433580 0.9523818223960842 0.4173121568867584 T T T

0.6827684362336062 0.2873254944474338 0.4183102502084339 T T T

0.6788183994104218 0.6063838605437434 0.4246509630449366 T T T

0.6820346543869623 0.9524788527941571 0.4183902325575082 T T T

0.8595153798452078 0.2793427640318780 0.4230407945493360 T T T

0.8479184129525156 0.6072661154520856 0.4170490979042786 T T T

0.8575098181573353 0.9425243110872209 0.4226337231645707 T T T

0.0725409811313851 0.0677283344563188 0.3352832482364967 T T T

0.0736294843338387 0.4049064251811013 0.3348859136090619 T T T

0.0797171442205069 0.7335075426491083 0.3399468376442580 T T T

0.2462522814877607 0.0669870358865331 0.3381154704590392 T T T

0.2441990517895273 0.3984629523966859 0.3395737868214896 T T T

0.2562088785365547 0.7428779930266145 0.3352375598827517 T T T

0.4216777674482582 0.0731368723509491 0.3343251228845128 T T T

0.4220376238548457 0.4115443659195512 0.3359984831284845 T T T

0.4141542592148094 0.7363409170789619 0.3346132292386931 T T T

0.5804514409112013 0.0711156348959734 0.3350002582935697 T T T

0.5801582262814520 0.4050196071320112 0.3351404376220981 T T T

0.5741030013529644 0.7365955199154047 0.3352137615487578 T T T

0.7421838064346542 0.0734772341505760 0.3341111724189522 T T T

0.7453735185159504 0.4133312685926382 0.3354787925216540 T T T

0.7444850680126075 0.7342412285342655 0.3383583672858038 T T T

0.9117208838989102 0.0649640892389504 0.3381873454748854 T T T

0.9118720517682782 0.4012458994974913 0.3384020923860742 T T T

0.9208151209973964 0.7510464170070702 0.3356035741604538 T T T

0.0822335439771932 0.2401312867129682 0.3463226690863587 T T T

0.0839235110981590 0.5754688652809180 0.3457746898748129 T T T

0.0824998777727405 0.9070458340563184 0.3445162934746479 T T T

0.2497498915002755 0.2408634792872553 0.3458070384582250 T T T

0.2505853866227789 0.5770263675523114 0.3432014262645045 T T T

0.2513979842365911 0.9085559292104006 0.3449425368282203 T T T

0.4187770945019197 0.2448248416635762 0.3434527086314606 T T T

0.4149737049688645 0.5751040682053589 0.3424812088465788 T T T

0.4193842656044242 0.9111318130094263 0.3426013773316632 T T T

0.5851791939254704 0.2464227487906003 0.3433880015452584 T T T

0.5816168323331337 0.5761815748691044 0.3443337315864484 T T T

0.5835305698564591 0.9105733617594981 0.3435857788370695 T T T

0.7505524549062278 0.2446589338189172 0.3457113831340667 T T T

0.7494123683397039 0.5769665026749138 0.3438864958804421 T T T

0.7479909506365643 0.9069641246923270 0.3461898576806001 T T T

0.9170105284141462 0.2433332436673581 0.3438756649466059 T T T

0.9165667478237383 0.5765896211577562 0.3442183176502905 T T T

0.9154670016454117 0.9073801820374884 0.3429180607732090 T T T

0.1397131416109474 0.0545266729236502 0.1118484288951365 F F F

0.1397131416109474 0.3878600062569788 0.1118484288951365 F F F

0.1397131416109474 0.7211933395903145 0.1118484288951365 F F F

0.3063798082776117 0.0545266729236502 0.1118484288951365 F F F

0.3063798082776117 0.3878600062569788 0.1118484288951365 F F F

0.3063798082776117 0.7211933395903145 0.1118484288951365 F F F

0.4730464749442831 0.0545266729236502 0.1118484288951365 F F F

0.4730464749442831 0.3878600062569788 0.1118484288951365 F F F

0.4730464749442831 0.7211933395903145 0.1118484288951365 F F F

0.6397131416109474 0.0545266729236502 0.1118484288951365 F F F

0.6397131416109474 0.3878600062569788 0.1118484288951365 F F F

0.6397131416109474 0.7211933395903145 0.1118484288951365 F F F

0.8063798082776117 0.0545266729236502 0.1118484288951365 F F F

0.8063798082776117 0.3878600062569788 0.1118484288951365 F F F

0.8063798082776117 0.7211933395903145 0.1118484288951365 F F F

0.9730464749442831 0.0545266729236502 0.1118484288951365 F F F

0.9730464749442831 0.3878600062569788 0.1118484288951365 F F F

0.9730464749442831 0.7211933395903145 0.1118484288951365 F F F

0.0904969084302252 0.2563302902655593 0.2814745639703204 F F F

0.0904969084302252 0.5896636235988879 0.2814745639703204 F F F

0.0904969084302252 0.9229969569322236 0.2814745639703204 F F F

0.2571635750968966 0.2563302902655593 0.2814745639703204 F F F

0.2571635750968966 0.5896636235988879 0.2814745639703204 F F F

0.2571635750968966 0.9229969569322236 0.2814745639703204 F F F

0.4238302417635609 0.2563302902655593 0.2814745639703204 F F F

0.4238302417635609 0.5896636235988879 0.2814745639703204 F F F

0.4238302417635609 0.9229969569322236 0.2814745639703204 F F F

0.5904969084302252 0.2563302902655593 0.2814745639703204 F F F

0.5904969084302252 0.5896636235988879 0.2814745639703204 F F F

0.5904969084302252 0.9229969569322236 0.2814745639703204 F F F

0.7571635750968895 0.2563302902655593 0.2814745639703204 F F F

0.7571635750968895 0.5896636235988879 0.2814745639703204 F F F

0.7571635750968895 0.9229969569322236 0.2814745639703204 F F F

0.9238302417635609 0.2563302902655593 0.2814745639703204 F F F

0.9238302417635609 0.5896636235988879 0.2814745639703204 F F F

0.9238302417635609 0.9229969569322236 0.2814745639703204 F F F

0.0719170018627122 0.0743559305895458 0.2935290597345608 T T T

0.0717186245265701 0.4097536626597928 0.2934054865071695 T T T

0.0805244041296766 0.7401653130069716 0.2988558472543308 T T T

0.2476622082791111 0.0741276134255705 0.2968557524249348 T T T

0.2450977686394193 0.4043966409433376 0.2985023482939382 T T T

0.2575730644135570 0.7490776165635871 0.2935985346181198 T T T

0.4218885685716906 0.0775074321280317 0.2929993300626444 T T T

0.4244320159410991 0.4193559977765605 0.2941064403210188 T T T

0.4140136337020923 0.7408455663174962 0.2924310650435108 T T T

0.5798489919907249 0.0755776691507078 0.2929527886080411 T T T

0.5798040742918725 0.4087560632599070 0.2931999985446478 T T T

0.5747530374258994 0.7428505041943916 0.2935705079905820 T T T

0.7414018191031030 0.0785471154714807 0.2926098614490735 T T T

0.7458413535032933 0.4207203357664993 0.2935385730998012 T T T

0.7467085429166918 0.7411700619228055 0.2970680458601411 T T T

0.9139625412564412 0.0718273590145809 0.2966575918605253 T T T

0.9131504972960461 0.4069542449616083 0.2970872910508701 T T T

0.9236374122857689 0.7626258607629610 0.2941910920681075 T T T

0.0088203445300641 0.5943385466296380 0.4654701444816262 T T T

0.1779047979468093 0.2612273701536783 0.4660281051305481 T T T

0.1782049153100621 0.9317424172455606 0.4642551914218406 T T T

0.6726589041148312 0.5955990936612977 0.4643170820367750 T T T

0.8692591444297749 0.2749419153787591 0.4616254291928514 T T T

0.8673027799946631 0.9387012932017966 0.4612081591898358 T T T

0.00000000E+00 0.00000000E+00 0.00000000E+00

0.00000000E+00 0.00000000E+00 0.00000000E+00

0.00000000E+00 0.00000000E+00 0.00000000E+00

0.00000000E+00 0.00000000E+00 0.00000000E+00

0.00000000E+00 0.00000000E+00 0.00000000E+00

0.00000000E+00 0.00000000E+00 0.00000000E+00

0.00000000E+00 0.00000000E+00 0.00000000E+00

0.00000000E+00 0.00000000E+00 0.00000000E+00

0.00000000E+00 0.00000000E+00 0.00000000E+00

0.00000000E+00 0.00000000E+00 0.00000000E+00

0.00000000E+00 0.00000000E+00 0.00000000E+00

0.00000000E+00 0.00000000E+00 0.00000000E+00

0.00000000E+00 0.00000000E+00 0.00000000E+00

0.00000000E+00 0.00000000E+00 0.00000000E+00

0.00000000E+00 0.00000000E+00 0.00000000E+00

0.00000000E+00 0.00000000E+00 0.00000000E+00

0.00000000E+00 0.00000000E+00 0.00000000E+00

0.00000000E+00 0.00000000E+00 0.00000000E+00

0.00000000E+00 0.00000000E+00 0.00000000E+00

0.00000000E+00 0.00000000E+00 0.00000000E+00

0.00000000E+00 0.00000000E+00 0.00000000E+00

0.00000000E+00 0.00000000E+00 0.00000000E+00

0.00000000E+00 0.00000000E+00 0.00000000E+00

0.00000000E+00 0.00000000E+00 0.00000000E+00

0.00000000E+00 0.00000000E+00 0.00000000E+00

0.00000000E+00 0.00000000E+00 0.00000000E+00

0.00000000E+00 0.00000000E+00 0.00000000E+00

0.00000000E+00 0.00000000E+00 0.00000000E+00

0.00000000E+00 0.00000000E+00 0.00000000E+00

0.00000000E+00 0.00000000E+00 0.00000000E+00

0.00000000E+00 0.00000000E+00 0.00000000E+00

0.00000000E+00 0.00000000E+00 0.00000000E+00

0.00000000E+00 0.00000000E+00 0.00000000E+00

0.00000000E+00 0.00000000E+00 0.00000000E+00

0.00000000E+00 0.00000000E+00 0.00000000E+00

0.00000000E+00 0.00000000E+00 0.00000000E+00

0.00000000E+00 0.00000000E+00 0.00000000E+00

0.00000000E+00 0.00000000E+00 0.00000000E+00

0.00000000E+00 0.00000000E+00 0.00000000E+00

0.00000000E+00 0.00000000E+00 0.00000000E+00

0.00000000E+00 0.00000000E+00 0.00000000E+00

0.00000000E+00 0.00000000E+00 0.00000000E+00

0.00000000E+00 0.00000000E+00 0.00000000E+00

0.00000000E+00 0.00000000E+00 0.00000000E+00

0.00000000E+00 0.00000000E+00 0.00000000E+00

0.00000000E+00 0.00000000E+00 0.00000000E+00

0.00000000E+00 0.00000000E+00 0.00000000E+00

0.00000000E+00 0.00000000E+00 0.00000000E+00

0.00000000E+00 0.00000000E+00 0.00000000E+00

0.00000000E+00 0.00000000E+00 0.00000000E+00

0.00000000E+00 0.00000000E+00 0.00000000E+00

0.00000000E+00 0.00000000E+00 0.00000000E+00

0.00000000E+00 0.00000000E+00 0.00000000E+00

0.00000000E+00 0.00000000E+00 0.00000000E+00

0.00000000E+00 0.00000000E+00 0.00000000E+00

0.00000000E+00 0.00000000E+00 0.00000000E+00

0.00000000E+00 0.00000000E+00 0.00000000E+00

0.00000000E+00 0.00000000E+00 0.00000000E+00

0.00000000E+00 0.00000000E+00 0.00000000E+00

0.00000000E+00 0.00000000E+00 0.00000000E+00

0.00000000E+00 0.00000000E+00 0.00000000E+00

0.00000000E+00 0.00000000E+00 0.00000000E+00

0.00000000E+00 0.00000000E+00 0.00000000E+00

0.00000000E+00 0.00000000E+00 0.00000000E+00

0.00000000E+00 0.00000000E+00 0.00000000E+00

0.00000000E+00 0.00000000E+00 0.00000000E+00

0.00000000E+00 0.00000000E+00 0.00000000E+00

0.00000000E+00 0.00000000E+00 0.00000000E+00

0.00000000E+00 0.00000000E+00 0.00000000E+00

0.00000000E+00 0.00000000E+00 0.00000000E+00

0.00000000E+00 0.00000000E+00 0.00000000E+00

0.00000000E+00 0.00000000E+00 0.00000000E+00

0.00000000E+00 0.00000000E+00 0.00000000E+00

0.00000000E+00 0.00000000E+00 0.00000000E+00

0.00000000E+00 0.00000000E+00 0.00000000E+00

0.00000000E+00 0.00000000E+00 0.00000000E+00

0.00000000E+00 0.00000000E+00 0.00000000E+00

0.00000000E+00 0.00000000E+00 0.00000000E+00

0.00000000E+00 0.00000000E+00 0.00000000E+00

0.00000000E+00 0.00000000E+00 0.00000000E+00

0.00000000E+00 0.00000000E+00 0.00000000E+00

0.00000000E+00 0.00000000E+00 0.00000000E+00

0.00000000E+00 0.00000000E+00 0.00000000E+00

0.00000000E+00 0.00000000E+00 0.00000000E+00

0.00000000E+00 0.00000000E+00 0.00000000E+00

0.00000000E+00 0.00000000E+00 0.00000000E+00

0.00000000E+00 0.00000000E+00 0.00000000E+00

0.00000000E+00 0.00000000E+00 0.00000000E+00

0.00000000E+00 0.00000000E+00 0.00000000E+00

0.00000000E+00 0.00000000E+00 0.00000000E+00

0.00000000E+00 0.00000000E+00 0.00000000E+00

0.00000000E+00 0.00000000E+00 0.00000000E+00

0.00000000E+00 0.00000000E+00 0.00000000E+00

0.00000000E+00 0.00000000E+00 0.00000000E+00

0.00000000E+00 0.00000000E+00 0.00000000E+00

0.00000000E+00 0.00000000E+00 0.00000000E+00

0.00000000E+00 0.00000000E+00 0.00000000E+00

0.00000000E+00 0.00000000E+00 0.00000000E+00

0.00000000E+00 0.00000000E+00 0.00000000E+00

0.00000000E+00 0.00000000E+00 0.00000000E+00

0.00000000E+00 0.00000000E+00 0.00000000E+00

0.00000000E+00 0.00000000E+00 0.00000000E+00

0.00000000E+00 0.00000000E+00 0.00000000E+00

0.00000000E+00 0.00000000E+00 0.00000000E+00

0.00000000E+00 0.00000000E+00 0.00000000E+00

0.00000000E+00 0.00000000E+00 0.00000000E+00

0.00000000E+00 0.00000000E+00 0.00000000E+00

0.00000000E+00 0.00000000E+00 0.00000000E+00

0.00000000E+00 0.00000000E+00 0.00000000E+00

0.00000000E+00 0.00000000E+00 0.00000000E+00

0.00000000E+00 0.00000000E+00 0.00000000E+00

0.00000000E+00 0.00000000E+00 0.00000000E+00

0.00000000E+00 0.00000000E+00 0.00000000E+00

0.00000000E+00 0.00000000E+00 0.00000000E+00

0.00000000E+00 0.00000000E+00 0.00000000E+00

0.00000000E+00 0.00000000E+00 0.00000000E+00

0.00000000E+00 0.00000000E+00 0.00000000E+00

0.00000000E+00 0.00000000E+00 0.00000000E+00

0.00000000E+00 0.00000000E+00 0.00000000E+00

0.00000000E+00 0.00000000E+00 0.00000000E+00

0.00000000E+00 0.00000000E+00 0.00000000E+00

0.00000000E+00 0.00000000E+00 0.00000000E+00

0.00000000E+00 0.00000000E+00 0.00000000E+00

0.00000000E+00 0.00000000E+00 0.00000000E+00

0.00000000E+00 0.00000000E+00 0.00000000E+00

0.00000000E+00 0.00000000E+00 0.00000000E+00

0.00000000E+00 0.00000000E+00 0.00000000E+00

0.00000000E+00 0.00000000E+00 0.00000000E+00

0.00000000E+00 0.00000000E+00 0.00000000E+00

0.00000000E+00 0.00000000E+00 0.00000000E+00

0.00000000E+00 0.00000000E+00 0.00000000E+00

0.00000000E+00 0.00000000E+00 0.00000000E+00

0.00000000E+00 0.00000000E+00 0.00000000E+00

0.00000000E+00 0.00000000E+00 0.00000000E+00

0.00000000E+00 0.00000000E+00 0.00000000E+00

0.00000000E+00 0.00000000E+00 0.00000000E+00

0.00000000E+00 0.00000000E+00 0.00000000E+00

0.00000000E+00 0.00000000E+00 0.00000000E+00

0.00000000E+00 0.00000000E+00 0.00000000E+00

0.00000000E+00 0.00000000E+00 0.00000000E+00

0.00000000E+00 0.00000000E+00 0.00000000E+00

0.00000000E+00 0.00000000E+00 0.00000000E+00

0.00000000E+00 0.00000000E+00 0.00000000E+00

0.00000000E+00 0.00000000E+00 0.00000000E+00

0.00000000E+00 0.00000000E+00 0.00000000E+00

0.00000000E+00 0.00000000E+00 0.00000000E+00

0.00000000E+00 0.00000000E+00 0.00000000E+00

0.00000000E+00 0.00000000E+00 0.00000000E+00

0.00000000E+00 0.00000000E+00 0.00000000E+00

0.00000000E+00 0.00000000E+00 0.00000000E+00

0.00000000E+00 0.00000000E+00 0.00000000E+00

0.00000000E+00 0.00000000E+00 0.00000000E+00

0.00000000E+00 0.00000000E+00 0.00000000E+00

0.00000000E+00 0.00000000E+00 0.00000000E+00

0.00000000E+00 0.00000000E+00 0.00000000E+00

0.00000000E+00 0.00000000E+00 0.00000000E+00

0.00000000E+00 0.00000000E+00 0.00000000E+00

0.00000000E+00 0.00000000E+00 0.00000000E+00

0.00000000E+00 0.00000000E+00 0.00000000E+00

0.00000000E+00 0.00000000E+00 0.00000000E+00

0.00000000E+00 0.00000000E+00 0.00000000E+00

0.00000000E+00 0.00000000E+00 0.00000000E+00

0.00000000E+00 0.00000000E+00 0.00000000E+00

0.00000000E+00 0.00000000E+00 0.00000000E+00

0.00000000E+00 0.00000000E+00 0.00000000E+00

0.00000000E+00 0.00000000E+00 0.00000000E+00

0.00000000E+00 0.00000000E+00 0.00000000E+00

0.00000000E+00 0.00000000E+00 0.00000000E+00

0.00000000E+00 0.00000000E+00 0.00000000E+00

0.00000000E+00 0.00000000E+00 0.00000000E+00

0.00000000E+00 0.00000000E+00 0.00000000E+00

0.00000000E+00 0.00000000E+00 0.00000000E+00

0.00000000E+00 0.00000000E+00 0.00000000E+00

0.00000000E+00 0.00000000E+00 0.00000000E+00

0.00000000E+00 0.00000000E+00 0.00000000E+00

0.00000000E+00 0.00000000E+00 0.00000000E+00

0.00000000E+00 0.00000000E+00 0.00000000E+00

0.00000000E+00 0.00000000E+00 0.00000000E+00

0.00000000E+00 0.00000000E+00 0.00000000E+00

0.00000000E+00 0.00000000E+00 0.00000000E+00

0.00000000E+00 0.00000000E+00 0.00000000E+00

0.00000000E+00 0.00000000E+00 0.00000000E+00

0.00000000E+00 0.00000000E+00 0.00000000E+00

0.00000000E+00 0.00000000E+00 0.00000000E+00

0.00000000E+00 0.00000000E+00 0.00000000E+00

0.00000000E+00 0.00000000E+00 0.00000000E+00

0.00000000E+00 0.00000000E+00 0.00000000E+00

0.00000000E+00 0.00000000E+00 0.00000000E+00

0.00000000E+00 0.00000000E+00 0.00000000E+00

0.00000000E+00 0.00000000E+00 0.00000000E+00

0.00000000E+00 0.00000000E+00 0.00000000E+00

0.00000000E+00 0.00000000E+00 0.00000000E+00

0.00000000E+00 0.00000000E+00 0.00000000E+00

0.00000000E+00 0.00000000E+00 0.00000000E+00

0.00000000E+00 0.00000000E+00 0.00000000E+00

0.00000000E+00 0.00000000E+00 0.00000000E+00

0.00000000E+00 0.00000000E+00 0.00000000E+00

0.00000000E+00 0.00000000E+00 0.00000000E+00

0.00000000E+00 0.00000000E+00 0.00000000E+00

0.00000000E+00 0.00000000E+00 0.00000000E+00

0.00000000E+00 0.00000000E+00 0.00000000E+00

0.00000000E+00 0.00000000E+00 0.00000000E+00

0.00000000E+00 0.00000000E+00 0.00000000E+00

0.00000000E+00 0.00000000E+00 0.00000000E+00

0.00000000E+00 0.00000000E+00 0.00000000E+00

0.00000000E+00 0.00000000E+00 0.00000000E+00

0.00000000E+00 0.00000000E+00 0.00000000E+00

0.00000000E+00 0.00000000E+00 0.00000000E+00

0.00000000E+00 0.00000000E+00 0.00000000E+00

0.00000000E+00 0.00000000E+00 0.00000000E+00

0.00000000E+00 0.00000000E+00 0.00000000E+00

0.00000000E+00 0.00000000E+00 0.00000000E+00

0.00000000E+00 0.00000000E+00 0.00000000E+00

0.00000000E+00 0.00000000E+00 0.00000000E+00

0.00000000E+00 0.00000000E+00 0.00000000E+00

0.00000000E+00 0.00000000E+00 0.00000000E+00

0.00000000E+00 0.00000000E+00 0.00000000E+00

0.00000000E+00 0.00000000E+00 0.00000000E+00

0.00000000E+00 0.00000000E+00 0.00000000E+00

0.00000000E+00 0.00000000E+00 0.00000000E+00

0.00000000E+00 0.00000000E+00 0.00000000E+00

0.00000000E+00 0.00000000E+00 0.00000000E+00

0.00000000E+00 0.00000000E+00 0.00000000E+00

0.00000000E+00 0.00000000E+00 0.00000000E+00

0.00000000E+00 0.00000000E+00 0.00000000E+00

0.00000000E+00 0.00000000E+00 0.00000000E+00

0.00000000E+00 0.00000000E+00 0.00000000E+00

0.00000000E+00 0.00000000E+00 0.00000000E+00

0.00000000E+00 0.00000000E+00 0.00000000E+00

0.00000000E+00 0.00000000E+00 0.00000000E+00

0.00000000E+00 0.00000000E+00 0.00000000E+00

0.00000000E+00 0.00000000E+00 0.00000000E+00

0.00000000E+00 0.00000000E+00 0.00000000E+00

0.00000000E+00 0.00000000E+00 0.00000000E+00

0.00000000E+00 0.00000000E+00 0.00000000E+00

0.00000000E+00 0.00000000E+00 0.00000000E+00

0.00000000E+00 0.00000000E+00 0.00000000E+00

0.00000000E+00 0.00000000E+00 0.00000000E+00

0.00000000E+00 0.00000000E+00 0.00000000E+00

0.00000000E+00 0.00000000E+00 0.00000000E+00

0.00000000E+00 0.00000000E+00 0.00000000E+00

0.00000000E+00 0.00000000E+00 0.00000000E+00

0.00000000E+00 0.00000000E+00 0.00000000E+00

0.00000000E+00 0.00000000E+00 0.00000000E+00

0.00000000E+00 0.00000000E+00 0.00000000E+00

0.00000000E+00 0.00000000E+00 0.00000000E+00

0.00000000E+00 0.00000000E+00 0.00000000E+00

0.00000000E+00 0.00000000E+00 0.00000000E+00

0.00000000E+00 0.00000000E+00 0.00000000E+00

0.00000000E+00 0.00000000E+00 0.00000000E+00

0.00000000E+00 0.00000000E+00 0.00000000E+00

0.00000000E+00 0.00000000E+00 0.00000000E+00

0.00000000E+00 0.00000000E+00 0.00000000E+00

0.00000000E+00 0.00000000E+00 0.00000000E+00

0.00000000E+00 0.00000000E+00 0.00000000E+00

0.00000000E+00 0.00000000E+00 0.00000000E+00

0.00000000E+00 0.00000000E+00 0.00000000E+00

0.00000000E+00 0.00000000E+00 0.00000000E+00

0.00000000E+00 0.00000000E+00 0.00000000E+00

0.00000000E+00 0.00000000E+00 0.00000000E+00

0.00000000E+00 0.00000000E+00 0.00000000E+00

0.00000000E+00 0.00000000E+00 0.00000000E+00

0.00000000E+00 0.00000000E+00 0.00000000E+00

0.00000000E+00 0.00000000E+00 0.00000000E+00

0.00000000E+00 0.00000000E+00 0.00000000E+00

0.00000000E+00 0.00000000E+00 0.00000000E+00

0.00000000E+00 0.00000000E+00 0.00000000E+00

0.00000000E+00 0.00000000E+00 0.00000000E+00

0.00000000E+00 0.00000000E+00 0.00000000E+00

0.00000000E+00 0.00000000E+00 0.00000000E+00

0.00000000E+00 0.00000000E+00 0.00000000E+00

0.00000000E+00 0.00000000E+00 0.00000000E+00

0.00000000E+00 0.00000000E+00 0.00000000E+00

0.00000000E+00 0.00000000E+00 0.00000000E+00

0.00000000E+00 0.00000000E+00 0.00000000E+00

0.00000000E+00 0.00000000E+00 0.00000000E+00

NiOOH_3H_H_2_O ML CONTCAR

#======================================================================

# CRYSTAL DATA

#----------------------------------------------------------------------

data_VESTA_phase_1

_chemical_name_common 'This file is generated by VASPKIT code'

_cell_length_a 17.55600

_cell_length_b 17.73900

_cell_length_c 25.00000

_cell_angle_alpha 80.00000

_cell_angle_beta 90

_cell_angle_gamma 119.70000

_space_group_name_H-M_alt 'P 1'

_space_group_IT_number 1

loop_

_space_group_symop_operation_xyz

'x, y, z'

loop_

_atom_site_label

_atom_site_occupancy

_atom_site_fract_x

_atom_site_fract_y

_atom_site_fract_z

_atom_site_adp_type

_atom_site_B_iso_or_equiv

_atom_site_type_symbol

Ni1 1.0 0.034165 0.326002 0.196046 Biso 1.000000 Ni

Ni2 1.0 0.034165 0.659335 0.196046 Biso 1.000000 Ni

Ni3 1.0 0.034165 0.992669 0.196046 Biso 1.000000 Ni

Ni4 1.0 0.200832 0.326002 0.196046 Biso 1.000000 Ni

Ni5 1.0 0.200832 0.659335 0.196046 Biso 1.000000 Ni

Ni6 1.0 0.200832 0.992669 0.196046 Biso 1.000000 Ni

Ni7 1.0 0.367499 0.326002 0.196046 Biso 1.000000 Ni

Ni8 1.0 0.367499 0.659335 0.196046 Biso 1.000000 Ni

Ni9 1.0 0.367499 0.992669 0.196046 Biso 1.000000 Ni

Ni10 1.0 0.534165 0.326002 0.196046 Biso 1.000000 Ni

Ni11 1.0 0.534165 0.659335 0.196046 Biso 1.000000 Ni

Ni12 1.0 0.534165 0.992669 0.196046 Biso 1.000000 Ni

Ni13 1.0 0.700832 0.326002 0.196046 Biso 1.000000 Ni

Ni14 1.0 0.700832 0.659335 0.196046 Biso 1.000000 Ni

Ni15 1.0 0.700832 0.992669 0.196046 Biso 1.000000 Ni

Ni16 1.0 0.867499 0.326002 0.196046 Biso 1.000000 Ni

Ni17 1.0 0.867499 0.659335 0.196046 Biso 1.000000 Ni

Ni18 1.0 0.867499 0.992669 0.196046 Biso 1.000000 Ni

Ni19 1.0 0.035197 0.161767 0.194904 Biso 1.000000 Ni

Ni20 1.0 0.035197 0.495100 0.194904 Biso 1.000000 Ni

Ni21 1.0 0.035197 0.828433 0.194904 Biso 1.000000 Ni

Ni22 1.0 0.201864 0.161767 0.194904 Biso 1.000000 Ni

Ni23 1.0 0.201864 0.495100 0.194904 Biso 1.000000 Ni

Ni24 1.0 0.201864 0.828433 0.194904 Biso 1.000000 Ni

Ni25 1.0 0.368530 0.161767 0.194904 Biso 1.000000 Ni

Ni26 1.0 0.368530 0.495100 0.194904 Biso 1.000000 Ni

Ni27 1.0 0.368530 0.828433 0.194904 Biso 1.000000 Ni

Ni28 1.0 0.535197 0.161767 0.194904 Biso 1.000000 Ni

Ni29 1.0 0.535197 0.495100 0.194904 Biso 1.000000 Ni

Ni30 1.0 0.535197 0.828433 0.194904 Biso 1.000000 Ni

Ni31 1.0 0.701864 0.161767 0.194904 Biso 1.000000 Ni

Ni32 1.0 0.701864 0.495100 0.194904 Biso 1.000000 Ni

Ni33 1.0 0.701864 0.828433 0.194904 Biso 1.000000 Ni

Ni34 1.0 0.868530 0.161767 0.194904 Biso 1.000000 Ni

Ni35 1.0 0.868530 0.495100 0.194904 Biso 1.000000 Ni

Ni36 1.0 0.868530 0.828433 0.194904 Biso 1.000000 Ni

Ni37 1.0 0.133154 0.009989 0.376795 Biso 1.000000 Ni

Ni38 1.0 0.132828 0.343161 0.376556 Biso 1.000000 Ni

Ni39 1.0 0.136128 0.675471 0.381459 Biso 1.000000 Ni

Ni40 1.0 0.298298 0.006376 0.379395 Biso 1.000000 Ni

Ni41 1.0 0.296699 0.340377 0.378998 Biso 1.000000 Ni

Ni42 1.0 0.303488 0.677849 0.377204 Biso 1.000000 Ni

Ni43 1.0 0.466932 0.005512 0.377546 Biso 1.000000 Ni

Ni44 1.0 0.464261 0.337056 0.377758 Biso 1.000000 Ni

Ni45 1.0 0.466436 0.675073 0.377208 Biso 1.000000 Ni

Ni46 1.0 0.632733 0.009621 0.376837 Biso 1.000000 Ni

Ni47 1.0 0.630023 0.341796 0.377011 Biso 1.000000 Ni

Ni48 1.0 0.630898 0.674028 0.377184 Biso 1.000000 Ni

Ni49 1.0 0.797233 0.008812 0.379359 Biso 1.000000 Ni

Ni50 1.0 0.795483 0.342311 0.380139 Biso 1.000000 Ni

Ni51 1.0 0.795603 0.674580 0.376861 Biso 1.000000 Ni

Ni52 1.0 0.970133 0.008567 0.378135 Biso 1.000000 Ni

Ni53 1.0 0.969685 0.342064 0.378408 Biso 1.000000 Ni

Ni54 1.0 0.961678 0.673541 0.380720 Biso 1.000000 Ni

Ni55 1.0 0.133496 0.176704 0.380271 Biso 1.000000 Ni

Ni56 1.0 0.134533 0.510608 0.380371 Biso 1.000000 Ni

Ni57 1.0 0.133346 0.842191 0.380862 Biso 1.000000 Ni

Ni58 1.0 0.299902 0.175138 0.378103 Biso 1.000000 Ni

Ni59 1.0 0.298988 0.509203 0.379137 Biso 1.000000 Ni

Ni60 1.0 0.304364 0.843769 0.378426 Biso 1.000000 Ni

Ni61 1.0 0.465500 0.173141 0.380007 Biso 1.000000 Ni

Ni62 1.0 0.463830 0.506694 0.379592 Biso 1.000000 Ni

Ni63 1.0 0.469087 0.842950 0.379526 Biso 1.000000 Ni

Ni64 1.0 0.631645 0.174823 0.380852 Biso 1.000000 Ni

Ni65 1.0 0.629733 0.507365 0.380037 Biso 1.000000 Ni

Ni66 1.0 0.633336 0.842230 0.380205 Biso 1.000000 Ni

Ni67 1.0 0.798581 0.174232 0.379890 Biso 1.000000 Ni

Ni68 1.0 0.795996 0.508630 0.379463 Biso 1.000000 Ni

Ni69 1.0 0.798828 0.839810 0.380220 Biso 1.000000 Ni

Ni70 1.0 0.966459 0.175348 0.380030 Biso 1.000000 Ni

Ni71 1.0 0.965044 0.507676 0.382038 Biso 1.000000 Ni

Ni72 1.0 0.965003 0.840997 0.379448 Biso 1.000000 Ni

O1 1.0 0.082646 0.091367 0.230651 Biso 1.000000 O

O2 1.0 0.082646 0.424701 0.230651 Biso 1.000000 O

O3 1.0 0.082646 0.758034 0.230651 Biso 1.000000 O

O4 1.0 0.249313 0.091367 0.230651 Biso 1.000000 O

O5 1.0 0.249313 0.424701 0.230651 Biso 1.000000 O

O6 1.0 0.249313 0.758034 0.230651 Biso 1.000000 O

O7 1.0 0.415980 0.091367 0.230651 Biso 1.000000 O

O8 1.0 0.415980 0.424701 0.230651 Biso 1.000000 O

O9 1.0 0.415980 0.758034 0.230651 Biso 1.000000 O

O10 1.0 0.582646 0.091367 0.230651 Biso 1.000000 O

O11 1.0 0.582646 0.424701 0.230651 Biso 1.000000 O

O12 1.0 0.582646 0.758034 0.230651 Biso 1.000000 O

O13 1.0 0.749313 0.091367 0.230651 Biso 1.000000 O

O14 1.0 0.749313 0.424701 0.230651 Biso 1.000000 O

O15 1.0 0.749313 0.758034 0.230651 Biso 1.000000 O

O16 1.0 0.915980 0.091367 0.230651 Biso 1.000000 O

O17 1.0 0.915980 0.424701 0.230651 Biso 1.000000 O

O18 1.0 0.915980 0.758034 0.230651 Biso 1.000000 O

O19 1.0 0.090268 0.262510 0.240399 Biso 1.000000 O

O20 1.0 0.090268 0.595843 0.240399 Biso 1.000000 O

O21 1.0 0.090268 0.929177 0.240399 Biso 1.000000 O

O22 1.0 0.256934 0.262510 0.240399 Biso 1.000000 O

O23 1.0 0.256934 0.595843 0.240399 Biso 1.000000 O

O24 1.0 0.256934 0.929177 0.240399 Biso 1.000000 O

O25 1.0 0.423601 0.262510 0.240399 Biso 1.000000 O

O26 1.0 0.423601 0.595843 0.240399 Biso 1.000000 O

O27 1.0 0.423601 0.929177 0.240399 Biso 1.000000 O

O28 1.0 0.590268 0.262510 0.240399 Biso 1.000000 O

O29 1.0 0.590268 0.595843 0.240399 Biso 1.000000 O

O30 1.0 0.590268 0.929177 0.240399 Biso 1.000000 O

O31 1.0 0.756934 0.262510 0.240399 Biso 1.000000 O

O32 1.0 0.756934 0.595843 0.240399 Biso 1.000000 O

O33 1.0 0.756934 0.929177 0.240399 Biso 1.000000 O

O34 1.0 0.923601 0.262510 0.240399 Biso 1.000000 O

O35 1.0 0.923601 0.595843 0.240399 Biso 1.000000 O

O36 1.0 0.923601 0.929177 0.240399 Biso 1.000000 O

O37 1.0 0.145027 0.057403 0.150261 Biso 1.000000 O

O38 1.0 0.145027 0.390736 0.150261 Biso 1.000000 O

O39 1.0 0.145027 0.724070 0.150261 Biso 1.000000 O

O40 1.0 0.311694 0.057403 0.150261 Biso 1.000000 O

O41 1.0 0.311694 0.390736 0.150261 Biso 1.000000 O

O42 1.0 0.311694 0.724070 0.150261 Biso 1.000000 O

O43 1.0 0.478360 0.057403 0.150261 Biso 1.000000 O

O44 1.0 0.478360 0.390736 0.150261 Biso 1.000000 O

O45 1.0 0.478360 0.724070 0.150261 Biso 1.000000 O

O46 1.0 0.645027 0.057403 0.150261 Biso 1.000000 O

O47 1.0 0.645027 0.390736 0.150261 Biso 1.000000 O

O48 1.0 0.645027 0.724070 0.150261 Biso 1.000000 O

O49 1.0 0.811694 0.057403 0.150261 Biso 1.000000 O

O50 1.0 0.811694 0.390736 0.150261 Biso 1.000000 O

O51 1.0 0.811694 0.724070 0.150261 Biso 1.000000 O

O52 1.0 0.978360 0.057403 0.150261 Biso 1.000000 O

O53 1.0 0.978360 0.390736 0.150261 Biso 1.000000 O

O54 1.0 0.978360 0.724070 0.150261 Biso 1.000000 O

O55 1.0 0.154063 0.231317 0.159404 Biso 1.000000 O

O56 1.0 0.154063 0.564650 0.159404 Biso 1.000000 O

O57 1.0 0.154063 0.897984 0.159404 Biso 1.000000 O

O58 1.0 0.320730 0.231317 0.159404 Biso 1.000000 O

O59 1.0 0.320730 0.564650 0.159404 Biso 1.000000 O

O60 1.0 0.320730 0.897984 0.159404 Biso 1.000000 O

O61 1.0 0.487397 0.231317 0.159404 Biso 1.000000 O

O62 1.0 0.487397 0.564650 0.159404 Biso 1.000000 O

O63 1.0 0.487397 0.897984 0.159404 Biso 1.000000 O

O64 1.0 0.654063 0.231317 0.159404 Biso 1.000000 O

O65 1.0 0.654063 0.564650 0.159404 Biso 1.000000 O

O66 1.0 0.654063 0.897984 0.159404 Biso 1.000000 O

O67 1.0 0.820730 0.231317 0.159404 Biso 1.000000 O

O68 1.0 0.820730 0.564650 0.159404 Biso 1.000000 O

O69 1.0 0.820730 0.897984 0.159404 Biso 1.000000 O

O70 1.0 0.987397 0.231317 0.159404 Biso 1.000000 O

O71 1.0 0.987397 0.564650 0.159404 Biso 1.000000 O

O72 1.0 0.987397 0.897984 0.159404 Biso 1.000000 O

O73 1.0 0.015739 0.104274 0.413893 Biso 1.000000 O

O74 1.0 0.015949 0.436910 0.414578 Biso 1.000000 O

O75 1.0 0.010828 0.771815 0.414684 Biso 1.000000 O

O76 1.0 0.182137 0.106624 0.413423 Biso 1.000000 O

O77 1.0 0.180831 0.439060 0.412740 Biso 1.000000 O

O78 1.0 0.188687 0.776680 0.415005 Biso 1.000000 O

O79 1.0 0.346232 0.105072 0.413350 Biso 1.000000 O

O80 1.0 0.345893 0.437923 0.413405 Biso 1.000000 O

O81 1.0 0.351824 0.774143 0.413390 Biso 1.000000 O

O82 1.0 0.511843 0.100907 0.414130 Biso 1.000000 O

O83 1.0 0.509786 0.433372 0.414238 Biso 1.000000 O

O84 1.0 0.515013 0.771059 0.413024 Biso 1.000000 O

O85 1.0 0.679333 0.104005 0.414011 Biso 1.000000 O

O86 1.0 0.676669 0.437479 0.413441 Biso 1.000000 O

O87 1.0 0.679441 0.769472 0.413434 Biso 1.000000 O

O88 1.0 0.847181 0.105954 0.414037 Biso 1.000000 O

O89 1.0 0.843756 0.439772 0.414777 Biso 1.000000 O

O90 1.0 0.845164 0.769023 0.413609 Biso 1.000000 O

O91 1.0 0.023247 0.286605 0.417787 Biso 1.000000 O

O92 1.0 0.015083 0.607000 0.426219 Biso 1.000000 O

O93 1.0 0.024498 0.953890 0.417673 Biso 1.000000 O

O94 1.0 0.183361 0.286571 0.417651 Biso 1.000000 O

O95 1.0 0.196269 0.621677 0.418499 Biso 1.000000 O

O96 1.0 0.184042 0.952709 0.417150 Biso 1.000000 O

O97 1.0 0.354620 0.266146 0.417979 Biso 1.000000 O

O98 1.0 0.356465 0.620961 0.417397 Biso 1.000000 O

O99 1.0 0.356846 0.934475 0.418024 Biso 1.000000 O

O100 1.0 0.519935 0.283393 0.417471 Biso 1.000000 O

O101 1.0 0.520553 0.618509 0.417916 Biso 1.000000 O

O102 1.0 0.522478 0.951839 0.417297 Biso 1.000000 O

O103 1.0 0.681885 0.286684 0.418486 Biso 1.000000 O

O104 1.0 0.684604 0.618250 0.417642 Biso 1.000000 O

O105 1.0 0.684135 0.953743 0.418092 Biso 1.000000 O

O106 1.0 0.862738 0.280828 0.423181 Biso 1.000000 O

O107 1.0 0.844019 0.615099 0.417781 Biso 1.000000 O

O108 1.0 0.863532 0.946618 0.423023 Biso 1.000000 O

O109 1.0 0.078324 0.069870 0.334888 Biso 1.000000 O

O110 1.0 0.077656 0.403541 0.334980 Biso 1.000000 O

O111 1.0 0.079247 0.734723 0.340382 Biso 1.000000 O

O112 1.0 0.246167 0.079268 0.335307 Biso 1.000000 O

O113 1.0 0.242310 0.406988 0.333979 Biso 1.000000 O

O114 1.0 0.258661 0.748152 0.336265 Biso 1.000000 O

O115 1.0 0.413875 0.066935 0.337273 Biso 1.000000 O

O116 1.0 0.412245 0.399257 0.337240 Biso 1.000000 O

O117 1.0 0.416146 0.737236 0.335012 Biso 1.000000 O

O118 1.0 0.579126 0.070488 0.335482 Biso 1.000000 O

O119 1.0 0.576368 0.402286 0.335439 Biso 1.000000 O

O120 1.0 0.579226 0.735711 0.335355 Biso 1.000000 O

O121 1.0 0.743035 0.074632 0.334231 Biso 1.000000 O

O122 1.0 0.741552 0.408441 0.334981 Biso 1.000000 O

O123 1.0 0.743514 0.735673 0.335173 Biso 1.000000 O

O124 1.0 0.913738 0.067035 0.337928 Biso 1.000000 O

O125 1.0 0.912769 0.401228 0.338764 Biso 1.000000 O

O126 1.0 0.907023 0.738713 0.334805 Biso 1.000000 O

O127 1.0 0.084123 0.244261 0.343424 Biso 1.000000 O

O128 1.0 0.084682 0.577709 0.346045 Biso 1.000000 O

O129 1.0 0.084643 0.911279 0.343553 Biso 1.000000 O

O130 1.0 0.250258 0.242588 0.344313 Biso 1.000000 O

O131 1.0 0.252199 0.580234 0.343324 Biso 1.000000 O

O132 1.0 0.252474 0.909101 0.344378 Biso 1.000000 O

O133 1.0 0.416287 0.241192 0.342554 Biso 1.000000 O

O134 1.0 0.416860 0.576977 0.343558 Biso 1.000000 O

O135 1.0 0.419428 0.910127 0.342205 Biso 1.000000 O

O136 1.0 0.582795 0.243056 0.343824 Biso 1.000000 O

O137 1.0 0.581716 0.575783 0.343894 Biso 1.000000 O

O138 1.0 0.585217 0.911485 0.343186 Biso 1.000000 O

O139 1.0 0.749865 0.243729 0.346325 Biso 1.000000 O

O140 1.0 0.747490 0.576905 0.343156 Biso 1.000000 O

O141 1.0 0.751037 0.909711 0.345961 Biso 1.000000 O

O142 1.0 0.918016 0.244352 0.343893 Biso 1.000000 O

O143 1.0 0.915320 0.576367 0.345603 Biso 1.000000 O

O144 1.0 0.918099 0.910515 0.344015 Biso 1.000000 O

O145 1.0 0.524579 0.502075 0.513954 Biso 1.000000 O

H1 1.0 0.525783 0.553588 0.490968 Biso 1.000000 H

H2 1.0 0.514218 0.461630 0.488954 Biso 1.000000 H

H3 1.0 0.139713 0.054527 0.111848 Biso 1.000000 H

H4 1.0 0.139713 0.387860 0.111848 Biso 1.000000 H

H5 1.0 0.139713 0.721193 0.111848 Biso 1.000000 H

H6 1.0 0.306380 0.054527 0.111848 Biso 1.000000 H

H7 1.0 0.306380 0.387860 0.111848 Biso 1.000000 H

H8 1.0 0.306380 0.721193 0.111848 Biso 1.000000 H

H9 1.0 0.473046 0.054527 0.111848 Biso 1.000000 H

H10 1.0 0.473046 0.387860 0.111848 Biso 1.000000 H

H11 1.0 0.473046 0.721193 0.111848 Biso 1.000000 H

H12 1.0 0.639713 0.054527 0.111848 Biso 1.000000 H

H13 1.0 0.639713 0.387860 0.111848 Biso 1.000000 H

H14 1.0 0.639713 0.721193 0.111848 Biso 1.000000 H

H15 1.0 0.806380 0.054527 0.111848 Biso 1.000000 H

H16 1.0 0.806380 0.387860 0.111848 Biso 1.000000 H

H17 1.0 0.806380 0.721193 0.111848 Biso 1.000000 H

H18 1.0 0.973046 0.054527 0.111848 Biso 1.000000 H

H19 1.0 0.973046 0.387860 0.111848 Biso 1.000000 H

H20 1.0 0.973046 0.721193 0.111848 Biso 1.000000 H

H21 1.0 0.090497 0.256330 0.281475 Biso 1.000000 H

H22 1.0 0.090497 0.589664 0.281475 Biso 1.000000 H

H23 1.0 0.090497 0.922997 0.281475 Biso 1.000000 H

H24 1.0 0.257164 0.256330 0.281475 Biso 1.000000 H

H25 1.0 0.257164 0.589664 0.281475 Biso 1.000000 H

H26 1.0 0.257164 0.922997 0.281475 Biso 1.000000 H

H27 1.0 0.423830 0.256330 0.281475 Biso 1.000000 H

H28 1.0 0.423830 0.589664 0.281475 Biso 1.000000 H

H29 1.0 0.423830 0.922997 0.281475 Biso 1.000000 H

H30 1.0 0.590497 0.256330 0.281475 Biso 1.000000 H

H31 1.0 0.590497 0.589664 0.281475 Biso 1.000000 H

H32 1.0 0.590497 0.922997 0.281475 Biso 1.000000 H

H33 1.0 0.757164 0.256330 0.281475 Biso 1.000000 H

H34 1.0 0.757164 0.589664 0.281475 Biso 1.000000 H

H35 1.0 0.757164 0.922997 0.281475 Biso 1.000000 H

H36 1.0 0.923830 0.256330 0.281475 Biso 1.000000 H

H37 1.0 0.923830 0.589664 0.281475 Biso 1.000000 H

H38 1.0 0.923830 0.922997 0.281475 Biso 1.000000 H

H39 1.0 0.077128 0.073675 0.292591 Biso 1.000000 H

H40 1.0 0.076431 0.407271 0.292842 Biso 1.000000 H

H41 1.0 0.080924 0.741097 0.299343 Biso 1.000000 H

H42 1.0 0.246297 0.087259 0.293334 Biso 1.000000 H

H43 1.0 0.241179 0.411445 0.292506 Biso 1.000000 H

H44 1.0 0.260468 0.755747 0.294612 Biso 1.000000 H

H45 1.0 0.414449 0.072807 0.295778 Biso 1.000000 H

H46 1.0 0.413187 0.405148 0.295759 Biso 1.000000 H

H47 1.0 0.414809 0.740716 0.293079 Biso 1.000000 H

H48 1.0 0.578980 0.074943 0.293565 Biso 1.000000 H

H49 1.0 0.577098 0.407202 0.293411 Biso 1.000000 H

H50 1.0 0.578695 0.739745 0.293470 Biso 1.000000 H

H51 1.0 0.741977 0.079353 0.292799 Biso 1.000000 H

H52 1.0 0.741657 0.414386 0.293353 Biso 1.000000 H

H53 1.0 0.744267 0.740433 0.293049 Biso 1.000000 H

H54 1.0 0.914303 0.072383 0.296593 Biso 1.000000 H

H55 1.0 0.913574 0.406617 0.297559 Biso 1.000000 H

H56 1.0 0.907141 0.743934 0.293358 Biso 1.000000 H

H57 1.0 0.008815 0.597237 0.465860 Biso 1.000000 H

H58 1.0 0.872819 0.277402 0.461743 Biso 1.000000 H

H59 1.0 0.873617 0.945067 0.461544 Biso 1.000000 H

Ni_0.98_Fe_0.02__H_2_O ML CONTCAR

#======================================================================

# CRYSTAL DATA

#----------------------------------------------------------------------

data_VESTA_phase_1

_chemical_name_common 'This file is generated by VASPKIT code'

_cell_length_a 17.556000

_cell_length_b 17.739000

_cell_length_c 25.000000

_cell_angle_alpha 80.000000

_cell_angle_beta 90.000000

_cell_angle_gamma 119.699997

_cell_volume 6626.345312

_space_group_name_H-M_alt 'P 1'

_space_group_IT_number 1

loop_

_space_group_symop_operation_xyz

'x, y, z'

loop_

_atom_site_label

_atom_site_occupancy

_atom_site_fract_x

_atom_site_fract_y

_atom_site_fract_z

_atom_site_adp_type

_atom_site_B_iso_or_equiv

_atom_site_type_symbol

Fe1 1.0 0.465390 0.507789 0.382808 Biso 1.000000 Fe

Fe2 1.0 0.796660 0.502530 0.383820 Biso 1.000000 Ni

Fe3 1.0 0.701915 0.829237 0.194966 Biso 1.000000 Fe

Fe4 1.0 0.201828 0.162495 0.194822 Biso 1.000000 Ni

Ni1 1.0 0.034086 0.326701 0.196066 Biso 1.000000 Ni

Ni2 1.0 0.034143 0.660079 0.196082 Biso 1.000000 Ni

Ni3 1.0 0.034127 0.993354 0.196082 Biso 1.000000 Ni

Ni4 1.0 0.200714 0.326649 0.195957 Biso 1.000000 Ni

Ni5 1.0 0.200823 0.660061 0.196089 Biso 1.000000 Ni

Ni6 1.0 0.200802 0.993349 0.196094 Biso 1.000000 Ni

Ni7 1.0 0.367416 0.326609 0.195724 Biso 1.000000 Ni

Ni8 1.0 0.367477 0.660066 0.196084 Biso 1.000000 Ni

Ni9 1.0 0.367472 0.993344 0.196127 Biso 1.000000 Ni

Ni10 1.0 0.534170 0.326703 0.195940 Biso 1.000000 Ni

Ni11 1.0 0.534170 0.660053 0.196184 Biso 1.000000 Ni

Ni12 1.0 0.534148 0.993363 0.196123 Biso 1.000000 Ni

Ni13 1.0 0.700823 0.326700 0.196064 Biso 1.000000 Ni

Ni14 1.0 0.700835 0.660085 0.196123 Biso 1.000000 Ni

Ni15 1.0 0.700835 0.993410 0.196090 Biso 1.000000 Ni

Ni16 1.0 0.867456 0.326708 0.196111 Biso 1.000000 Ni

Ni17 1.0 0.867487 0.660091 0.196067 Biso 1.000000 Ni

Ni18 1.0 0.867479 0.993396 0.196084 Biso 1.000000 Ni

Ni19 1.0 0.035163 0.162534 0.194889 Biso 1.000000 Ni

Ni20 1.0 0.035225 0.495915 0.194921 Biso 1.000000 Ni

Ni21 1.0 0.035242 0.829231 0.194928 Biso 1.000000 Ni

Ni22 1.0 0.201866 0.495866 0.194879 Biso 1.000000 Ni

Ni23 1.0 0.201906 0.829215 0.194963 Biso 1.000000 Ni

Ni24 1.0 0.368530 0.162492 0.194826 Biso 1.000000 Ni

Ni25 1.0 0.368481 0.495792 0.194840 Biso 1.000000 Ni

Ni26 1.0 0.368572 0.829190 0.195000 Biso 1.000000 Ni

Ni27 1.0 0.535219 0.162535 0.194904 Biso 1.000000 Ni

Ni28 1.0 0.535212 0.495806 0.194863 Biso 1.000000 Ni

Ni29 1.0 0.535245 0.829214 0.194984 Biso 1.000000 Ni

Ni30 1.0 0.701880 0.162552 0.194937 Biso 1.000000 Ni

Ni31 1.0 0.701899 0.495881 0.194900 Biso 1.000000 Ni

Ni32 1.0 0.868533 0.162567 0.194931 Biso 1.000000 Ni

Ni33 1.0 0.868558 0.495910 0.194926 Biso 1.000000 Ni

Ni34 1.0 0.868561 0.829222 0.194950 Biso 1.000000 Ni

Ni35 1.0 0.131440 0.007604 0.379624 Biso 1.000000 Ni

Ni36 1.0 0.131848 0.341696 0.379628 Biso 1.000000 Ni

Ni37 1.0 0.134159 0.674917 0.379607 Biso 1.000000 Ni

Ni38 1.0 0.298205 0.006964 0.379724 Biso 1.000000 Ni

Ni39 1.0 0.298397 0.341804 0.379495 Biso 1.000000 Ni

Ni40 1.0 0.301020 0.674375 0.380276 Biso 1.000000 Ni

Ni41 1.0 0.465664 0.008038 0.379544 Biso 1.000000 Ni

Ni42 1.0 0.463479 0.340373 0.381096 Biso 1.000000 Ni

Ni43 1.0 0.469655 0.676238 0.379212 Biso 1.000000 Ni

Ni44 1.0 0.632639 0.009298 0.379206 Biso 1.000000 Ni

Ni45 1.0 0.628351 0.339871 0.381590 Biso 1.000000 Ni

Ni46 1.0 0.633292 0.677651 0.376858 Biso 1.000000 Ni

Ni47 1.0 0.799321 0.009425 0.379123 Biso 1.000000 Ni

Ni48 1.0 0.799173 0.340565 0.380413 Biso 1.000000 Ni

Ni49 1.0 0.799257 0.676134 0.379053 Biso 1.000000 Ni

Ni50 1.0 0.965389 0.008570 0.379512 Biso 1.000000 Ni

Ni51 1.0 0.965455 0.341618 0.379479 Biso 1.000000 Ni

Ni52 1.0 0.967936 0.675491 0.379136 Biso 1.000000 Ni

Ni53 1.0 0.129207 0.172603 0.380524 Biso 1.000000 Ni

Ni54 1.0 0.132070 0.505870 0.380934 Biso 1.000000 Ni

Ni55 1.0 0.133165 0.838587 0.380590 Biso 1.000000 Ni

Ni56 1.0 0.294907 0.170661 0.379880 Biso 1.000000 Ni

Ni57 1.0 0.297717 0.505887 0.381608 Biso 1.000000 Ni

Ni58 1.0 0.300295 0.838473 0.380762 Biso 1.000000 Ni

Ni59 1.0 0.461119 0.171165 0.380550 Biso 1.000000 Ni

Ni60 1.0 0.467588 0.839424 0.380619 Biso 1.000000 Ni

Ni61 1.0 0.628677 0.172776 0.380509 Biso 1.000000 Ni

Ni62 1.0 0.631466 0.508404 0.380635 Biso 1.000000 Ni

Ni63 1.0 0.634427 0.841002 0.379921 Biso 1.000000 Ni

Ni64 1.0 0.795674 0.172529 0.380669 Biso 1.000000 Ni

Ni65 1.0 0.799814 0.840226 0.380102 Biso 1.000000 Ni

Ni66 1.0 0.962464 0.172633 0.380659 Biso 1.000000 Ni

Ni67 1.0 0.966614 0.505736 0.381086 Biso 1.000000 Ni

Ni68 1.0 0.966365 0.839215 0.380361 Biso 1.000000 Ni

O1 1.0 0.082552 0.092106 0.230633 Biso 1.000000 O

O2 1.0 0.082582 0.425453 0.230638 Biso 1.000000 O

O3 1.0 0.082594 0.758794 0.230664 Biso 1.000000 O

O4 1.0 0.249226 0.092106 0.230625 Biso 1.000000 O

O5 1.0 0.249180 0.425349 0.230544 Biso 1.000000 O

O6 1.0 0.249256 0.758744 0.230707 Biso 1.000000 O

O7 1.0 0.415911 0.092115 0.230654 Biso 1.000000 O

O8 1.0 0.415775 0.425148 0.230340 Biso 1.000000 O

O9 1.0 0.415907 0.758710 0.230735 Biso 1.000000 O

O10 1.0 0.582573 0.092113 0.230673 Biso 1.000000 O

O11 1.0 0.582581 0.425361 0.230568 Biso 1.000000 O

O12 1.0 0.582621 0.758814 0.230709 Biso 1.000000 O

O13 1.0 0.749258 0.092142 0.230663 Biso 1.000000 O

O14 1.0 0.749237 0.425440 0.230644 Biso 1.000000 O

O15 1.0 0.749235 0.758747 0.230699 Biso 1.000000 O

O16 1.0 0.915904 0.092136 0.230652 Biso 1.000000 O

O17 1.0 0.915917 0.425480 0.230667 Biso 1.000000 O

O18 1.0 0.915929 0.758779 0.230659 Biso 1.000000 O

O19 1.0 0.090363 0.263195 0.240366 Biso 1.000000 O

O20 1.0 0.090425 0.596573 0.240422 Biso 1.000000 O

O21 1.0 0.090406 0.929854 0.240445 Biso 1.000000 O

O22 1.0 0.257022 0.263124 0.240180 Biso 1.000000 O

O23 1.0 0.257068 0.596507 0.240435 Biso 1.000000 O

O24 1.0 0.257059 0.929851 0.240475 Biso 1.000000 O

O25 1.0 0.423686 0.263207 0.240204 Biso 1.000000 O

O26 1.0 0.423659 0.596489 0.240502 Biso 1.000000 O

O27 1.0 0.423749 0.929858 0.240481 Biso 1.000000 O

O28 1.0 0.590389 0.263241 0.240365 Biso 1.000000 O

O29 1.0 0.590403 0.596443 0.240613 Biso 1.000000 O

O30 1.0 0.590459 0.929900 0.240470 Biso 1.000000 O

O31 1.0 0.757047 0.263198 0.240455 Biso 1.000000 O

O32 1.0 0.757048 0.596575 0.240438 Biso 1.000000 O

O33 1.0 0.757102 0.929919 0.240452 Biso 1.000000 O

O34 1.0 0.923719 0.263210 0.240455 Biso 1.000000 O

O35 1.0 0.923719 0.596585 0.240413 Biso 1.000000 O

O36 1.0 0.923744 0.929891 0.240430 Biso 1.000000 O

O37 1.0 0.144727 0.058024 0.150278 Biso 1.000000 O

O38 1.0 0.144664 0.391426 0.150218 Biso 1.000000 O

O39 1.0 0.144793 0.724794 0.150303 Biso 1.000000 O

O40 1.0 0.311425 0.058022 0.150304 Biso 1.000000 O

O41 1.0 0.311304 0.391375 0.150087 Biso 1.000000 O

O42 1.0 0.311467 0.724813 0.150300 Biso 1.000000 O

O43 1.0 0.478103 0.058061 0.150321 Biso 1.000000 O

O44 1.0 0.478241 0.391457 0.150051 Biso 1.000000 O

O45 1.0 0.478163 0.724792 0.150346 Biso 1.000000 O

O46 1.0 0.644770 0.058110 0.150304 Biso 1.000000 O

O47 1.0 0.644873 0.391458 0.150207 Biso 1.000000 O

O48 1.0 0.644775 0.724734 0.150349 Biso 1.000000 O

O49 1.0 0.811460 0.058148 0.150275 Biso 1.000000 O

O50 1.0 0.811465 0.391442 0.150289 Biso 1.000000 O

O51 1.0 0.811477 0.724823 0.150290 Biso 1.000000 O

O52 1.0 0.978096 0.058090 0.150274 Biso 1.000000 O

O53 1.0 0.978069 0.391457 0.150294 Biso 1.000000 O

O54 1.0 0.978134 0.724809 0.150262 Biso 1.000000 O

O55 1.0 0.153977 0.232011 0.159285 Biso 1.000000 O

O56 1.0 0.154123 0.565446 0.159392 Biso 1.000000 O

O57 1.0 0.154135 0.898733 0.159402 Biso 1.000000 O

O58 1.0 0.320688 0.231895 0.159170 Biso 1.000000 O

O59 1.0 0.320779 0.565449 0.159374 Biso 1.000000 O

O60 1.0 0.320791 0.898713 0.159457 Biso 1.000000 O

O61 1.0 0.487483 0.232039 0.159288 Biso 1.000000 O

O62 1.0 0.487470 0.565444 0.159451 Biso 1.000000 O

O63 1.0 0.487448 0.898715 0.159460 Biso 1.000000 O

O64 1.0 0.654134 0.232074 0.159382 Biso 1.000000 O

O65 1.0 0.654148 0.565477 0.159424 Biso 1.000000 O

O66 1.0 0.654144 0.898769 0.159426 Biso 1.000000 O

O67 1.0 0.820772 0.232110 0.159415 Biso 1.000000 O

O68 1.0 0.820777 0.565442 0.159398 Biso 1.000000 O

O69 1.0 0.820791 0.898755 0.159414 Biso 1.000000 O

O70 1.0 0.987372 0.232087 0.159375 Biso 1.000000 O

O71 1.0 0.987447 0.565460 0.159398 Biso 1.000000 O

O72 1.0 0.987451 0.898733 0.159395 Biso 1.000000 O

O73 1.0 0.010754 0.103088 0.415650 Biso 1.000000 O

O74 1.0 0.014578 0.436190 0.415790 Biso 1.000000 O

O75 1.0 0.014233 0.769382 0.415374 Biso 1.000000 O

O76 1.0 0.177051 0.102816 0.415499 Biso 1.000000 O

O77 1.0 0.180718 0.436299 0.416144 Biso 1.000000 O

O78 1.0 0.181789 0.768946 0.415614 Biso 1.000000 O

O79 1.0 0.342830 0.101182 0.415738 Biso 1.000000 O

O80 1.0 0.347059 0.436712 0.418004 Biso 1.000000 O

O81 1.0 0.349137 0.769206 0.415811 Biso 1.000000 O

O82 1.0 0.511034 0.102677 0.415592 Biso 1.000000 O

O83 1.0 0.516313 0.447921 0.420222 Biso 1.000000 O

O84 1.0 0.518126 0.771678 0.414955 Biso 1.000000 O

O85 1.0 0.677638 0.103359 0.415591 Biso 1.000000 O

O86 1.0 0.673675 0.435078 0.418551 Biso 1.000000 O

O87 1.0 0.680924 0.770145 0.414443 Biso 1.000000 O

O88 1.0 0.844389 0.103259 0.415669 Biso 1.000000 O

O89 1.0 0.850510 0.433518 0.417886 Biso 1.000000 O

O90 1.0 0.847817 0.770489 0.414804 Biso 1.000000 O

O91 1.0 0.011675 0.277140 0.424688 Biso 1.000000 O

O92 1.0 0.017112 0.611098 0.424811 Biso 1.000000 O

O93 1.0 0.021075 0.943145 0.424661 Biso 1.000000 O

O94 1.0 0.177340 0.276541 0.424746 Biso 1.000000 O

O95 1.0 0.182702 0.609433 0.424995 Biso 1.000000 O

O96 1.0 0.187759 0.942548 0.424890 Biso 1.000000 O

O97 1.0 0.342706 0.276491 0.424058 Biso 1.000000 O

O98 1.0 0.350009 0.609565 0.425085 Biso 1.000000 O

O99 1.0 0.354981 0.942442 0.424940 Biso 1.000000 O

O100 1.0 0.510080 0.269747 0.426599 Biso 1.000000 O

O101 1.0 0.524731 0.618670 0.418890 Biso 1.000000 O

O102 1.0 0.522684 0.944077 0.424460 Biso 1.000000 O

O103 1.0 0.679366 0.277297 0.424790 Biso 1.000000 O

O104 1.0 0.680909 0.617106 0.420576 Biso 1.000000 O

O105 1.0 0.689245 0.944463 0.424150 Biso 1.000000 O

O106 1.0 0.845587 0.275553 0.425072 Biso 1.000000 O

O107 1.0 0.847646 0.607754 0.425448 Biso 1.000000 O

O108 1.0 0.854898 0.943686 0.424281 Biso 1.000000 O

O109 1.0 0.074949 0.071255 0.336178 Biso 1.000000 O

O110 1.0 0.084618 0.405175 0.336132 Biso 1.000000 O

O111 1.0 0.083878 0.738740 0.335982 Biso 1.000000 O

O112 1.0 0.240871 0.069264 0.336234 Biso 1.000000 O

O113 1.0 0.251142 0.405172 0.336667 Biso 1.000000 O

O114 1.0 0.251018 0.737189 0.336481 Biso 1.000000 O

O115 1.0 0.407972 0.069221 0.336348 Biso 1.000000 O

O116 1.0 0.418699 0.412936 0.335433 Biso 1.000000 O

O117 1.0 0.418896 0.737155 0.336805 Biso 1.000000 O

O118 1.0 0.575972 0.072007 0.335913 Biso 1.000000 O

O119 1.0 0.582558 0.405407 0.338181 Biso 1.000000 O

O120 1.0 0.589180 0.740533 0.333851 Biso 1.000000 O

O121 1.0 0.742638 0.072460 0.335833 Biso 1.000000 O

O122 1.0 0.750878 0.407125 0.337140 Biso 1.000000 O

O123 1.0 0.744183 0.738075 0.335353 Biso 1.000000 O

O124 1.0 0.909156 0.072155 0.335928 Biso 1.000000 O

O125 1.0 0.917126 0.404444 0.336342 Biso 1.000000 O

O126 1.0 0.913550 0.736297 0.336264 Biso 1.000000 O

O127 1.0 0.080911 0.243023 0.345574 Biso 1.000000 O

O128 1.0 0.084292 0.576404 0.345766 Biso 1.000000 O

O129 1.0 0.084682 0.908659 0.345564 Biso 1.000000 O

O130 1.0 0.247643 0.243417 0.344954 Biso 1.000000 O

O131 1.0 0.250754 0.576052 0.346043 Biso 1.000000 O

O132 1.0 0.251680 0.908153 0.345730 Biso 1.000000 O

O133 1.0 0.410732 0.236252 0.344604 Biso 1.000000 O

O134 1.0 0.416583 0.575694 0.345680 Biso 1.000000 O

O135 1.0 0.418732 0.908786 0.345688 Biso 1.000000 O

O136 1.0 0.581435 0.243489 0.345481 Biso 1.000000 O

O137 1.0 0.584782 0.578998 0.343111 Biso 1.000000 O

O138 1.0 0.586027 0.910364 0.345160 Biso 1.000000 O

O139 1.0 0.747967 0.243277 0.345708 Biso 1.000000 O

O140 1.0 0.748313 0.577413 0.345106 Biso 1.000000 O

O141 1.0 0.752644 0.910928 0.344759 Biso 1.000000 O

O142 1.0 0.914464 0.242871 0.345602 Biso 1.000000 O

O143 1.0 0.920487 0.577263 0.345514 Biso 1.000000 O

O144 1.0 0.918429 0.909644 0.345270 Biso 1.000000 O

O145 1.0 0.572835 0.550963 0.510311 Biso 1.000000 O

H1 1.0 0.547067 0.583778 0.488771 Biso 1.000000 H

H2 1.0 0.546614 0.496496 0.495456 Biso 1.000000 H

H3 1.0 0.138786 0.055168 0.111906 Biso 1.000000 H

H4 1.0 0.138656 0.388475 0.111861 Biso 1.000000 H

H5 1.0 0.138847 0.721897 0.111935 Biso 1.000000 H

H6 1.0 0.305516 0.055186 0.111929 Biso 1.000000 H

H7 1.0 0.305273 0.388493 0.111723 Biso 1.000000 H

H8 1.0 0.305532 0.721951 0.111928 Biso 1.000000 H

H9 1.0 0.472192 0.055223 0.111944 Biso 1.000000 H

H10 1.0 0.472629 0.388596 0.111663 Biso 1.000000 H

H11 1.0 0.472369 0.722051 0.111951 Biso 1.000000 H

H12 1.0 0.638864 0.055261 0.111929 Biso 1.000000 H

H13 1.0 0.639030 0.388516 0.111840 Biso 1.000000 H

H14 1.0 0.638894 0.721929 0.111963 Biso 1.000000 H

H15 1.0 0.805545 0.055298 0.111899 Biso 1.000000 H

H16 1.0 0.805544 0.388526 0.111922 Biso 1.000000 H

H17 1.0 0.805645 0.721919 0.111917 Biso 1.000000 H

H18 1.0 0.972182 0.055280 0.111894 Biso 1.000000 H

H19 1.0 0.972138 0.388525 0.111930 Biso 1.000000 H

H20 1.0 0.972267 0.721896 0.111891 Biso 1.000000 H

H21 1.0 0.090682 0.256431 0.281474 Biso 1.000000 H

H22 1.0 0.090735 0.589803 0.281531 Biso 1.000000 H

H23 1.0 0.090690 0.923023 0.281566 Biso 1.000000 H

H24 1.0 0.257108 0.255961 0.281299 Biso 1.000000 H

H25 1.0 0.257484 0.589825 0.281501 Biso 1.000000 H

H26 1.0 0.257346 0.923011 0.281593 Biso 1.000000 H

H27 1.0 0.423879 0.256285 0.281309 Biso 1.000000 H

H28 1.0 0.424030 0.590202 0.281432 Biso 1.000000 H

H29 1.0 0.424046 0.923080 0.281593 Biso 1.000000 H

H30 1.0 0.590656 0.256504 0.281480 Biso 1.000000 H

H31 1.0 0.591118 0.590199 0.281528 Biso 1.000000 H

H32 1.0 0.590780 0.923238 0.281582 Biso 1.000000 H

H33 1.0 0.757349 0.256507 0.281572 Biso 1.000000 H

H34 1.0 0.757318 0.589907 0.281521 Biso 1.000000 H

H35 1.0 0.757481 0.923304 0.281563 Biso 1.000000 H

H36 1.0 0.924020 0.256510 0.281576 Biso 1.000000 H

H37 1.0 0.924018 0.589821 0.281525 Biso 1.000000 H

H38 1.0 0.924054 0.923136 0.281546 Biso 1.000000 H

H39 1.0 0.073984 0.077396 0.295047 Biso 1.000000 H

H40 1.0 0.086355 0.411216 0.294884 Biso 1.000000 H

H41 1.0 0.084096 0.744760 0.294768 Biso 1.000000 H

H42 1.0 0.241328 0.076240 0.295013 Biso 1.000000 H

H43 1.0 0.253258 0.411285 0.295460 Biso 1.000000 H

H44 1.0 0.251675 0.743696 0.295256 Biso 1.000000 H

H45 1.0 0.407927 0.076490 0.295114 Biso 1.000000 H

H46 1.0 0.418966 0.419328 0.294429 Biso 1.000000 H

H47 1.0 0.419719 0.743176 0.295579 Biso 1.000000 H

H48 1.0 0.575705 0.078408 0.294657 Biso 1.000000 H

H49 1.0 0.585060 0.411203 0.297227 Biso 1.000000 H

H50 1.0 0.588239 0.743636 0.292295 Biso 1.000000 H

H51 1.0 0.742341 0.078934 0.294514 Biso 1.000000 H

H52 1.0 0.752184 0.413716 0.295809 Biso 1.000000 H

H53 1.0 0.744119 0.742832 0.293662 Biso 1.000000 H

H54 1.0 0.908955 0.078597 0.294641 Biso 1.000000 H

H55 1.0 0.918586 0.410410 0.295111 Biso 1.000000 H

H56 1.0 0.914379 0.742874 0.294878 Biso 1.000000 H

H57 1.0 0.007584 0.279183 0.463125 Biso 1.000000 H

H58 1.0 0.018672 0.615610 0.463032 Biso 1.000000 H

H59 1.0 0.027453 -0.054971 0.463099 Biso 1.000000 H

H60 1.0 0.173162 0.278030 0.463272 Biso 1.000000 H

H61 1.0 0.181147 0.610072 0.463747 Biso 1.000000 H

H62 1.0 0.194433 -0.055106 0.463262 Biso 1.000000 H

H63 1.0 0.336586 0.276183 0.462758 Biso 1.000000 H

H64 1.0 0.348438 0.609285 0.463968 Biso 1.000000 H

H65 1.0 0.360927 -0.056040 0.463441 Biso 1.000000 H

H66 1.0 0.507852 0.261798 0.466040 Biso 1.000000 H

H67 1.0 0.529229 -0.053117 0.462791 Biso 1.000000 H

H68 1.0 0.675033 0.276043 0.463725 Biso 1.000000 H

H69 1.0 0.658426 0.603533 0.460246 Biso 1.000000 H

H70 1.0 0.695075 -0.054548 0.462719 Biso 1.000000 H

H71 1.0 0.843443 0.279906 0.463202 Biso 1.000000 H

H72 1.0 0.847035 0.609147 0.464122 Biso 1.000000 H

H73 1.0 0.860643 -0.055393 0.462862 Biso 1.000000 H

Ni_0.8_Fe_0.2__H_2_O ML CONTCAR

#======================================================================

# CRYSTAL DATA

#----------------------------------------------------------------------

data_VESTA_phase_1

_chemical_name_common 'This file is generated by VASPKIT code'

_cell_length_a 17.55600

_cell_length_b 17.73900

_cell_length_c 25.00000

_cell_angle_alpha 80.00000

_cell_angle_beta 90

_cell_angle_gamma 119.70000

_space_group_name_H-M_alt 'P 1'

_space_group_IT_number 1

loop_

_space_group_symop_operation_xyz

'x, y, z'

loop_

_atom_site_label

_atom_site_occupancy

_atom_site_fract_x

_atom_site_fract_y

_atom_site_fract_z

_atom_site_adp_type

_atom_site_B_iso_or_equiv

_atom_site_type_symbol

Fe1 1.0 0.467163 0.510189 0.385377 Biso 1.000000 Fe

Fe2 1.0 0.798224 0.167383 0.383149 Biso 1.000000 Fe

Fe3 1.0 0.701915 0.829237 0.194966 Biso 1.000000 Fe

Fe4 1.0 0.201828 0.162495 0.194822 Biso 1.000000 Fe

Fe5 1.0 0.201866 0.495866 0.194879 Biso 1.000000 Fe

Fe6 1.0 0.201906 0.829215 0.194963 Biso 1.000000 Fe

Fe7 1.0 0.701880 0.162552 0.194937 Biso 1.000000 Fe

Fe8 1.0 0.701899 0.495881 0.194900 Biso 1.000000 Fe

Fe9 1.0 0.297148 0.168893 0.383044 Biso 1.000000 Fe

Fe10 1.0 0.296919 0.834169 0.381888 Biso 1.000000 Fe

Fe11 1.0 0.797071 0.502307 0.383104 Biso 1.000000 Fe

Fe12 1.0 0.798142 0.837000 0.381126 Biso 1.000000 Fe

Fe13 1.0 0.367472 0.993344 0.196127 Biso 1.000000 Fe

Fe14 1.0 0.466328 0.006988 0.382256 Biso 1.000000 Fe

Ni1 1.0 0.034086 0.326701 0.196066 Biso 1.000000 Ni

Ni2 1.0 0.034143 0.660079 0.196082 Biso 1.000000 Ni

Ni3 1.0 0.034127 0.993354 0.196082 Biso 1.000000 Ni

Ni4 1.0 0.200714 0.326649 0.195957 Biso 1.000000 Ni

Ni5 1.0 0.200823 0.660061 0.196089 Biso 1.000000 Ni

Ni6 1.0 0.200802 0.993349 0.196094 Biso 1.000000 Ni

Ni7 1.0 0.367416 0.326609 0.195724 Biso 1.000000 Ni

Ni8 1.0 0.367477 0.660066 0.196084 Biso 1.000000 Ni

Ni9 1.0 0.534170 0.326703 0.195940 Biso 1.000000 Ni

Ni10 1.0 0.534170 0.660053 0.196184 Biso 1.000000 Ni

Ni11 1.0 0.534148 0.993363 0.196123 Biso 1.000000 Ni

Ni12 1.0 0.700823 0.326700 0.196064 Biso 1.000000 Ni

Ni13 1.0 0.700835 0.660085 0.196123 Biso 1.000000 Ni

Ni14 1.0 0.700835 0.993410 0.196090 Biso 1.000000 Ni

Ni15 1.0 0.867456 0.326708 0.196111 Biso 1.000000 Ni

Ni16 1.0 0.867487 0.660091 0.196067 Biso 1.000000 Ni

Ni17 1.0 0.867479 0.993396 0.196084 Biso 1.000000 Ni

Ni18 1.0 0.035163 0.162534 0.194889 Biso 1.000000 Ni

Ni19 1.0 0.035225 0.495915 0.194921 Biso 1.000000 Ni

Ni20 1.0 0.035242 0.829231 0.194928 Biso 1.000000 Ni

Ni21 1.0 0.368530 0.162492 0.194826 Biso 1.000000 Ni

Ni22 1.0 0.368481 0.495792 0.194840 Biso 1.000000 Ni

Ni23 1.0 0.368572 0.829190 0.195000 Biso 1.000000 Ni

Ni24 1.0 0.535219 0.162535 0.194904 Biso 1.000000 Ni

Ni25 1.0 0.535212 0.495806 0.194863 Biso 1.000000 Ni

Ni26 1.0 0.535245 0.829214 0.194984 Biso 1.000000 Ni

Ni27 1.0 0.868533 0.162567 0.194931 Biso 1.000000 Ni

Ni28 1.0 0.868558 0.495910 0.194926 Biso 1.000000 Ni

Ni29 1.0 0.868561 0.829222 0.194950 Biso 1.000000 Ni

Ni30 1.0 0.131566 0.004528 0.380632 Biso 1.000000 Ni

Ni31 1.0 0.133046 0.339696 0.379942 Biso 1.000000 Ni

Ni32 1.0 0.131691 0.670119 0.380212 Biso 1.000000 Ni

Ni33 1.0 0.298043 0.004698 0.380512 Biso 1.000000 Ni

Ni34 1.0 0.299727 0.340418 0.380961 Biso 1.000000 Ni

Ni35 1.0 0.298344 0.668730 0.381216 Biso 1.000000 Ni

Ni36 1.0 0.465586 0.340907 0.383115 Biso 1.000000 Ni

Ni37 1.0 0.468118 0.673656 0.380122 Biso 1.000000 Ni

Ni38 1.0 0.632264 0.006062 0.380599 Biso 1.000000 Ni

Ni39 1.0 0.631061 0.340754 0.380896 Biso 1.000000 Ni

Ni40 1.0 0.632118 0.675783 0.379259 Biso 1.000000 Ni

Ni41 1.0 0.800105 0.004773 0.380767 Biso 1.000000 Ni

Ni42 1.0 0.799851 0.340052 0.379638 Biso 1.000000 Ni

Ni43 1.0 0.798817 0.673351 0.381246 Biso 1.000000 Ni

Ni44 1.0 0.966447 0.004885 0.380586 Biso 1.000000 Ni

Ni45 1.0 0.967515 0.339525 0.379665 Biso 1.000000 Ni

Ni46 1.0 0.966659 0.672537 0.380186 Biso 1.000000 Ni

Ni47 1.0 0.132689 0.171407 0.381674 Biso 1.000000 Ni

Ni48 1.0 0.131123 0.502852 0.381307 Biso 1.000000 Ni

Ni49 1.0 0.129833 0.835205 0.380174 Biso 1.000000 Ni

Ni50 1.0 0.297112 0.503302 0.382005 Biso 1.000000 Ni

Ni51 1.0 0.464867 0.171593 0.382553 Biso 1.000000 Ni

Ni52 1.0 0.465475 0.835126 0.380987 Biso 1.000000 Ni

Ni53 1.0 0.631914 0.173044 0.381454 Biso 1.000000 Ni

Ni54 1.0 0.632056 0.507548 0.381227 Biso 1.000000 Ni

Ni55 1.0 0.632327 0.837652 0.380085 Biso 1.000000 Ni

Ni56 1.0 0.967146 0.169736 0.381928 Biso 1.000000 Ni

Ni57 1.0 0.965793 0.502765 0.381277 Biso 1.000000 Ni

Ni58 1.0 0.963776 0.836060 0.380865 Biso 1.000000 Ni

O1 1.0 0.082552 0.092106 0.230633 Biso 1.000000 O

O2 1.0 0.082582 0.425453 0.230638 Biso 1.000000 O

O3 1.0 0.082594 0.758794 0.230664 Biso 1.000000 O

O4 1.0 0.249226 0.092106 0.230625 Biso 1.000000 O

O5 1.0 0.249180 0.425349 0.230544 Biso 1.000000 O

O6 1.0 0.249256 0.758744 0.230707 Biso 1.000000 O

O7 1.0 0.415911 0.092115 0.230654 Biso 1.000000 O

O8 1.0 0.415775 0.425148 0.230340 Biso 1.000000 O

O9 1.0 0.415907 0.758710 0.230735 Biso 1.000000 O

O10 1.0 0.582573 0.092113 0.230673 Biso 1.000000 O

O11 1.0 0.582581 0.425361 0.230568 Biso 1.000000 O

O12 1.0 0.582621 0.758814 0.230709 Biso 1.000000 O

O13 1.0 0.749258 0.092142 0.230663 Biso 1.000000 O

O14 1.0 0.749237 0.425440 0.230644 Biso 1.000000 O

O15 1.0 0.749235 0.758747 0.230699 Biso 1.000000 O

O16 1.0 0.915904 0.092136 0.230652 Biso 1.000000 O

O17 1.0 0.915917 0.425480 0.230667 Biso 1.000000 O

O18 1.0 0.915929 0.758779 0.230659 Biso 1.000000 O

O19 1.0 0.090363 0.263195 0.240366 Biso 1.000000 O

O20 1.0 0.090425 0.596573 0.240422 Biso 1.000000 O

O21 1.0 0.090406 0.929854 0.240445 Biso 1.000000 O

O22 1.0 0.257022 0.263124 0.240180 Biso 1.000000 O

O23 1.0 0.257068 0.596507 0.240435 Biso 1.000000 O

O24 1.0 0.257059 0.929851 0.240475 Biso 1.000000 O

O25 1.0 0.423686 0.263207 0.240204 Biso 1.000000 O

O26 1.0 0.423659 0.596489 0.240502 Biso 1.000000 O

O27 1.0 0.423749 0.929858 0.240481 Biso 1.000000 O

O28 1.0 0.590389 0.263241 0.240365 Biso 1.000000 O

O29 1.0 0.590403 0.596443 0.240613 Biso 1.000000 O

O30 1.0 0.590459 0.929900 0.240470 Biso 1.000000 O

O31 1.0 0.757047 0.263198 0.240455 Biso 1.000000 O

O32 1.0 0.757048 0.596575 0.240438 Biso 1.000000 O

O33 1.0 0.757102 0.929919 0.240452 Biso 1.000000 O

O34 1.0 0.923719 0.263210 0.240455 Biso 1.000000 O

O35 1.0 0.923719 0.596585 0.240413 Biso 1.000000 O

O36 1.0 0.923744 0.929891 0.240430 Biso 1.000000 O

O37 1.0 0.144727 0.058024 0.150278 Biso 1.000000 O

O38 1.0 0.144664 0.391426 0.150218 Biso 1.000000 O

O39 1.0 0.144793 0.724794 0.150303 Biso 1.000000 O

O40 1.0 0.311425 0.058022 0.150304 Biso 1.000000 O

O41 1.0 0.311304 0.391375 0.150087 Biso 1.000000 O

O42 1.0 0.311467 0.724813 0.150300 Biso 1.000000 O

O43 1.0 0.478103 0.058061 0.150321 Biso 1.000000 O

O44 1.0 0.478241 0.391457 0.150051 Biso 1.000000 O

O45 1.0 0.478163 0.724792 0.150346 Biso 1.000000 O

O46 1.0 0.644770 0.058110 0.150304 Biso 1.000000 O

O47 1.0 0.644873 0.391458 0.150207 Biso 1.000000 O

O48 1.0 0.644775 0.724734 0.150349 Biso 1.000000 O

O49 1.0 0.811460 0.058148 0.150275 Biso 1.000000 O

O50 1.0 0.811465 0.391442 0.150289 Biso 1.000000 O

O51 1.0 0.811477 0.724823 0.150290 Biso 1.000000 O

O52 1.0 0.978096 0.058090 0.150274 Biso 1.000000 O

O53 1.0 0.978069 0.391457 0.150294 Biso 1.000000 O

O54 1.0 0.978134 0.724809 0.150262 Biso 1.000000 O

O55 1.0 0.153977 0.232011 0.159285 Biso 1.000000 O

O56 1.0 0.154123 0.565446 0.159392 Biso 1.000000 O

O57 1.0 0.154135 0.898733 0.159402 Biso 1.000000 O

O58 1.0 0.320688 0.231895 0.159170 Biso 1.000000 O

O59 1.0 0.320779 0.565449 0.159374 Biso 1.000000 O

O60 1.0 0.320791 0.898713 0.159457 Biso 1.000000 O

O61 1.0 0.487483 0.232039 0.159288 Biso 1.000000 O

O62 1.0 0.487470 0.565444 0.159451 Biso 1.000000 O

O63 1.0 0.487448 0.898715 0.159460 Biso 1.000000 O

O64 1.0 0.654134 0.232074 0.159382 Biso 1.000000 O

O65 1.0 0.654148 0.565477 0.159424 Biso 1.000000 O

O66 1.0 0.654144 0.898769 0.159426 Biso 1.000000 O

O67 1.0 0.820772 0.232110 0.159415 Biso 1.000000 O

O68 1.0 0.820777 0.565442 0.159398 Biso 1.000000 O

O69 1.0 0.820791 0.898755 0.159414 Biso 1.000000 O

O70 1.0 0.987372 0.232087 0.159375 Biso 1.000000 O

O71 1.0 0.987447 0.565460 0.159398 Biso 1.000000 O

O72 1.0 0.987451 0.898733 0.159395 Biso 1.000000 O

O73 1.0 0.014057 0.100222 0.417343 Biso 1.000000 O

O74 1.0 0.012618 0.432992 0.416529 Biso 1.000000 O

O75 1.0 0.011886 0.766120 0.416367 Biso 1.000000 O

O76 1.0 0.185699 0.108613 0.419590 Biso 1.000000 O

O77 1.0 0.179048 0.433620 0.416642 Biso 1.000000 O

O78 1.0 0.178737 0.765910 0.416049 Biso 1.000000 O

O79 1.0 0.344668 0.098246 0.418831 Biso 1.000000 O

O80 1.0 0.345899 0.434517 0.418417 Biso 1.000000 O

O81 1.0 0.345501 0.763565 0.416409 Biso 1.000000 O

O82 1.0 0.516848 0.108013 0.418844 Biso 1.000000 O

O83 1.0 0.516652 0.447549 0.421045 Biso 1.000000 O

O84 1.0 0.515730 0.769243 0.416632 Biso 1.000000 O

O85 1.0 0.675351 0.099414 0.418089 Biso 1.000000 O

O86 1.0 0.675159 0.433870 0.418596 Biso 1.000000 O

O87 1.0 0.679427 0.768846 0.416239 Biso 1.000000 O

O88 1.0 0.850767 0.098507 0.418685 Biso 1.000000 O

O89 1.0 0.849135 0.432363 0.417880 Biso 1.000000 O

O90 1.0 0.846615 0.769304 0.416942 Biso 1.000000 O

O91 1.0 0.022223 0.275258 0.425241 Biso 1.000000 O

O92 1.0 0.019911 0.607266 0.425217 Biso 1.000000 O

O93 1.0 0.013536 0.937929 0.425481 Biso 1.000000 O

O94 1.0 0.188122 0.275851 0.424992 Biso 1.000000 O

O95 1.0 0.183423 0.603587 0.425881 Biso 1.000000 O

O96 1.0 0.185526 0.938141 0.424387 Biso 1.000000 O

O97 1.0 0.348120 0.270066 0.428141 Biso 1.000000 O

O98 1.0 0.348433 0.603310 0.426022 Biso 1.000000 O

O99 1.0 0.350048 0.939214 0.429203 Biso 1.000000 O

O100 1.0 0.517913 0.273130 0.426367 Biso 1.000000 O

O101 1.0 0.518936 0.613884 0.421533 Biso 1.000000 O

O102 1.0 0.517354 0.937760 0.426737 Biso 1.000000 O

O103 1.0 0.688298 0.278254 0.424384 Biso 1.000000 O

O104 1.0 0.686674 0.610370 0.423294 Biso 1.000000 O

O105 1.0 0.687417 0.944054 0.424358 Biso 1.000000 O

O106 1.0 0.853645 0.271767 0.425279 Biso 1.000000 O

O107 1.0 0.852311 0.604487 0.426336 Biso 1.000000 O

O108 1.0 0.846495 0.931000 0.424768 Biso 1.000000 O

O109 1.0 0.081316 0.072722 0.337370 Biso 1.000000 O

O110 1.0 0.077935 0.403351 0.336427 Biso 1.000000 O

O111 1.0 0.077889 0.734911 0.336563 Biso 1.000000 O

O112 1.0 0.249257 0.072980 0.336260 Biso 1.000000 O

O113 1.0 0.244149 0.403435 0.336978 Biso 1.000000 O

O114 1.0 0.243575 0.729237 0.336672 Biso 1.000000 O

O115 1.0 0.411749 0.069045 0.337209 Biso 1.000000 O

O116 1.0 0.411322 0.405871 0.338387 Biso 1.000000 O

O117 1.0 0.414410 0.732328 0.338119 Biso 1.000000 O

O118 1.0 0.581196 0.071100 0.336232 Biso 1.000000 O

O119 1.0 0.580600 0.406470 0.338152 Biso 1.000000 O

O120 1.0 0.583912 0.743679 0.334325 Biso 1.000000 O

O121 1.0 0.748674 0.073561 0.336439 Biso 1.000000 O

O122 1.0 0.746792 0.407271 0.336565 Biso 1.000000 O

O123 1.0 0.747542 0.742249 0.337756 Biso 1.000000 O

O124 1.0 0.912030 0.066487 0.338111 Biso 1.000000 O

O125 1.0 0.910837 0.401483 0.336590 Biso 1.000000 O

O126 1.0 0.909214 0.733707 0.337604 Biso 1.000000 O

O127 1.0 0.084618 0.240319 0.345980 Biso 1.000000 O

O128 1.0 0.083287 0.572378 0.345664 Biso 1.000000 O

O129 1.0 0.079691 0.900507 0.344762 Biso 1.000000 O

O130 1.0 0.252414 0.242946 0.344918 Biso 1.000000 O

O131 1.0 0.249953 0.572460 0.345612 Biso 1.000000 O

O132 1.0 0.251789 0.908239 0.344915 Biso 1.000000 O

O133 1.0 0.412139 0.234795 0.345943 Biso 1.000000 O

O134 1.0 0.416425 0.573385 0.345620 Biso 1.000000 O

O135 1.0 0.415497 0.903080 0.345262 Biso 1.000000 O

O136 1.0 0.584290 0.243522 0.345831 Biso 1.000000 O

O137 1.0 0.584863 0.577232 0.344195 Biso 1.000000 O

O138 1.0 0.584726 0.908567 0.345634 Biso 1.000000 O

O139 1.0 0.749240 0.242679 0.344685 Biso 1.000000 O

O140 1.0 0.748920 0.574819 0.344822 Biso 1.000000 O

O141 1.0 0.750628 0.908302 0.345075 Biso 1.000000 O

O142 1.0 0.922079 0.241697 0.345286 Biso 1.000000 O

O143 1.0 0.920125 0.574698 0.345521 Biso 1.000000 O

O144 1.0 0.917447 0.908043 0.344831 Biso 1.000000 O

O145 1.0 0.569294 0.534996 0.512921 Biso 1.000000 O

H1 1.0 0.547315 0.573202 0.494447 Biso 1.000000 H

H2 1.0 0.546364 0.488129 0.491224 Biso 1.000000 H

H3 1.0 0.138786 0.055168 0.111906 Biso 1.000000 H

H4 1.0 0.138656 0.388475 0.111861 Biso 1.000000 H

H5 1.0 0.138847 0.721897 0.111935 Biso 1.000000 H

H6 1.0 0.305516 0.055186 0.111929 Biso 1.000000 H

H7 1.0 0.305273 0.388493 0.111723 Biso 1.000000 H

H8 1.0 0.305532 0.721951 0.111928 Biso 1.000000 H

H9 1.0 0.472192 0.055223 0.111944 Biso 1.000000 H

H10 1.0 0.472629 0.388596 0.111663 Biso 1.000000 H

H11 1.0 0.472369 0.722051 0.111951 Biso 1.000000 H

H12 1.0 0.638864 0.055261 0.111929 Biso 1.000000 H

H13 1.0 0.639030 0.388516 0.111840 Biso 1.000000 H

H14 1.0 0.638894 0.721929 0.111963 Biso 1.000000 H

H15 1.0 0.805545 0.055298 0.111899 Biso 1.000000 H

H16 1.0 0.805544 0.388526 0.111922 Biso 1.000000 H

H17 1.0 0.805645 0.721919 0.111917 Biso 1.000000 H

H18 1.0 0.972182 0.055280 0.111894 Biso 1.000000 H

H19 1.0 0.972138 0.388525 0.111930 Biso 1.000000 H

H20 1.0 0.972267 0.721896 0.111891 Biso 1.000000 H

H21 1.0 0.090682 0.256431 0.281474 Biso 1.000000 H

H22 1.0 0.090735 0.589803 0.281531 Biso 1.000000 H

H23 1.0 0.090690 0.923023 0.281566 Biso 1.000000 H

H24 1.0 0.257108 0.255961 0.281299 Biso 1.000000 H

H25 1.0 0.257484 0.589825 0.281501 Biso 1.000000 H

H26 1.0 0.257346 0.923011 0.281593 Biso 1.000000 H

H27 1.0 0.423879 0.256285 0.281309 Biso 1.000000 H

H28 1.0 0.424030 0.590202 0.281432 Biso 1.000000 H

H29 1.0 0.424046 0.923080 0.281593 Biso 1.000000 H

H30 1.0 0.590656 0.256504 0.281480 Biso 1.000000 H

H31 1.0 0.591118 0.590199 0.281528 Biso 1.000000 H

H32 1.0 0.590780 0.923238 0.281582 Biso 1.000000 H

H33 1.0 0.757349 0.256507 0.281572 Biso 1.000000 H

H34 1.0 0.757318 0.589907 0.281521 Biso 1.000000 H

H35 1.0 0.757481 0.923304 0.281563 Biso 1.000000 H

H36 1.0 0.924020 0.256510 0.281576 Biso 1.000000 H

H37 1.0 0.924018 0.589821 0.281525 Biso 1.000000 H

H38 1.0 0.924054 0.923136 0.281546 Biso 1.000000 H

H39 1.0 0.080353 0.079562 0.296816 Biso 1.000000 H

H40 1.0 0.075931 0.409323 0.295523 Biso 1.000000 H

H41 1.0 0.075976 0.741764 0.295663 Biso 1.000000 H

H42 1.0 0.251488 0.081068 0.295255 Biso 1.000000 H

H43 1.0 0.244636 0.410165 0.295903 Biso 1.000000 H

H44 1.0 0.244169 0.736567 0.295436 Biso 1.000000 H

H45 1.0 0.411951 0.077983 0.295924 Biso 1.000000 H

H46 1.0 0.411118 0.411827 0.297409 Biso 1.000000 H

H47 1.0 0.416801 0.739700 0.296841 Biso 1.000000 H

H48 1.0 0.581797 0.077182 0.295092 Biso 1.000000 H

H49 1.0 0.581890 0.412778 0.297476 Biso 1.000000 H

H50 1.0 0.584048 0.750297 0.293442 Biso 1.000000 H

H51 1.0 0.749117 0.080133 0.295354 Biso 1.000000 H

H52 1.0 0.747106 0.414028 0.295421 Biso 1.000000 H

H53 1.0 0.748813 0.751605 0.296636 Biso 1.000000 H

H54 1.0 0.912240 0.073238 0.297019 Biso 1.000000 H

H55 1.0 0.910806 0.409168 0.295395 Biso 1.000000 H

H56 1.0 0.909428 0.741519 0.296467 Biso 1.000000 H

H57 1.0 0.028109 0.278701 0.463533 Biso 1.000000 H

H58 1.0 0.025527 0.610549 0.463536 Biso 1.000000 H

H59 1.0 0.007728 0.934169 0.464606 Biso 1.000000 H

H60 1.0 0.192414 0.276477 0.463680 Biso 1.000000 H

H61 1.0 0.185417 0.600402 0.464963 Biso 1.000000 H

H62 1.0 0.188420 0.934969 0.463450 Biso 1.000000 H

H63 1.0 0.339882 0.260854 0.467580 Biso 1.000000 H

H64 1.0 0.347567 0.599093 0.465271 Biso 1.000000 H

H65 1.0 0.348981 0.941464 0.467709 Biso 1.000000 H

H66 1.0 0.521083 0.267422 0.465511 Biso 1.000000 H

H67 1.0 0.521724 0.935230 0.465660 Biso 1.000000 H

H68 1.0 0.692422 0.280235 0.462952 Biso 1.000000 H

H69 1.0 0.671359 0.597509 0.463252 Biso 1.000000 H

H70 1.0 0.692133 0.947505 0.462742 Biso 1.000000 H

H71 1.0 0.857862 0.274000 0.463778 Biso 1.000000 H

H72 1.0 0.853759 0.603540 0.465278 Biso 1.000000 H

H73 1.0 0.839984 0.914540 0.464308 Biso 1.000000 H
