# Supplementary material for: Decoupling electron transfer defines a quantitative kinetic framework for oxygen evolution catalysis
Source: Nat Commun. 2026 Jun 10;17:7377. doi: 10.1038/s41467-026-74392-3 (PMC13402610; doi:10.1038/s41467-026-74392-3)
Supplement: Supplementary file 4 — Transparent Peer Review File [file 41467_2026_74392_MOESM4_ESM.pdf]

nature portfolio

Peer Review File

Decoupling electron transfer defines a quantitative kinetic framework for oxygen evolution catalysis

Corresponding Author: Professor Junmin Xue

Parts of this Peer Review File have been redacted as indicated to remove third-party material.

This file contains all reviewer reports in order by version, followed by all author rebuttals in order by version.

Version 0:

Reviewer comments:

Reviewer #1

(Remarks to the Author)
This manuscript presents a novel and potentially impactful electrochemical methodology, termed Open-Circuit Voltage Pulse Voltammetry (OCV-PV), to quantitatively decouple the electron transfer steps in the oxygen evolution reaction (OER). By isolating the rate of *OOH formation that widely regarded as the rate-determining step (RDS) from the total current, the authors propose a new kinetic descriptor. The combination of this kinetic analysis with in situ XAS and ATR-SEIRAS provides a compelling narrative that clarifies the distinct roles of Fe and Mn dopants in Ni-based oxyhydroxides, leading to the rational design of a ternary NiFeMn catalyst. However, several critical issues must be addressed before it could be reconsidered for publication on the high-level journal like Nature Communications
1.The core premise of the OCV-PV method is that the reduction of Ni^{4+} during the OCV period is driven exclusively by electron transfer from electrolyte OH^- ions to form *OOH. However, the authors used Carbon Cloth as the substrate. It is well-known that high-valence nickel species can oxidize carbon substrates under OER conditions, acting as a self-discharge pathway. The authors must rigorously rule out the contribution of carbon oxidation to the charge transfer during OCV.
2.Ni K-edge XAS in transmission mode probes the bulk oxidation state. However, OER and the proposed *OOH formation are surface-confined processes. During OCV, the reduction of bulk Ni^{4+} to Ni^{3+} might be limited by proton diffusion from the electrolyte into the lattice, rather than solely by surface electron transfer kinetics. The authors need to discuss the influence of proton diffusion coefficients
3.While the manuscript focuses on kinetics, the rational design claim culminates in the NiFeMn catalyst. However, kinetic insights must translate into robust performance, while current manuscript lacks sufficient stability data.
4.Is it possible that radical recombination (e.g., 2 *OH = H_2O + *O) contributes to the charge transfer during OCV?
5.Please provide more details in the Methods section regarding the calibration of the DEMS signal. How was the claimed negligible contribution of O_2 evolution quantified in terms of molar sensitivity?
6.Please supply XRD patterns for the doped samples (NiFe, NiMn, etc.) in the Supplementary Information to confirm that 2% doping did not induce phase segregation or impurity phases.

Reviewer #2

(Remarks to the Author)
In the paper entitled “Decoupling electron transfer defines a quantitative kinetic framework for oxygen evolution catalysis”, the authors have presented a nice, conceptually strong work. It presents a clear and well-designed OCV–PV strategy to quantify *OOH formation kinetics, and the approach is experimentally grounded in understanding the OER kinetics. There are some queries to be addressed before proceeding with publication.
Comment 1: What is the thermodynamic driving force for continued *OOH formation under open-circuit conditions? How can it be compared to the *OOH formation under applied-bias OER?
Comment 2: Even after voltage shutdown, residual charge or the slow discharge electrode during OCV might be assisting/contributing to *OOH formation.
Comment 3: The 1163 cm^{-1} peak from ATR-SEIRAS, assigned to *OOH intermediate formed during OER, can also have an overlap with C-O stretching, O-H bending, or electrolyte-related vibrational modes. How do the authors rule out the

interference?
Comment 4: Would the same OCV-driven *OOH formation be observed in electrolytes other than 1 M KOH or in other transition-metal oxyhydroxides?
Comment 5: The authors claim that "each *OOH formation event corresponds to the acceptance of two electrons by the Ni^{4+} sites during the OCV process." This statement, although scientifically deep, requires a clearer explanation of how the redistribution of electrons internally ($Ni^{4+} \rightarrow Ni^{3+}$) under OCV occurs.
Comment 6: Authors state that OCV–PV and PV measurements decouple electrolyte → catalyst and catalyst → circuit electron transfer. However, the dopants may alter the mechanisms and, hence, the later steps by altering the conductivity, introducing defects, or altering the catalyst's surface reconstruction. How is this approach valid for all dopants? Also, are all dopants (V, Cr, Mn, Fe, Co, Cu, Zn, Ce) truly substitutional?
Comment 7: On what basis is the potential of 1.503 used to perform the analysis?
Comment 8: In Figure 1, the captions for Figures d and e are missing.

Reviewer #3

(Remarks to the Author)
This study introduces a novel electrochemical methodology (OCV-PV) to quantitatively decouple the kinetics of elementary steps in the oxygen evolution reaction (OER): the deprotonation of OH* and the formation of OOH* intermediates. By applying this approach, the authors systematically elucidate how distinct dopant elements modulate the rates of these elementary steps on the NiOOH catalyst surface. Notably, Fe doping predominantly accelerates OOH* formation, whereas Mn doping enhances OH* deprotonation. Leveraging these mechanistic insights, the authors rationally design a high-performance FeMn co-doped NiOOH catalyst that enables targeted kinetic regulation of both elementary steps, resulting in substantially improved OER activity. These findings may arouse interest in related fields, but several issues need to be considered:

1. How can the influence of deprotonation of OOH*, and OH^- (or O_2) mass transfer limitations on OER kinetics be excluded?
2. Does the proposed OCV method under investigation enable accurate quantification of the OOH* formation rate? Specifically, can it adequately account for the contributions of OH^- adsorption and O_2* desorption to this kinetic step? Given that surface O* concentration serves as a critical descriptor in contemporary mechanistic studies, to what extent might the lattice oxygen participation mechanism influence the overall reaction pathway?
3. The DFT calculations presented in Figure 1 do not substantiate the efficacy of the proposed OCV method. In my opinion, they primarily elucidate the dependence of the theoretical activation energy (Ea) for elementary reaction steps on the surface coverage of O*. Furthermore, when the O* coverage exceeds 0.5, the variation in Ea becomes marginal.
4. What is the fundamental mechanism by which distinct dopant elements selectively facilitate specific elementary steps in the OER process? This mechanistic aspect remains insufficiently elucidated in current version.
5. Although macroscopic performance data suggest that Fe primarily facilitates the formation of OOH*, while Mn enhances the deprotonation of OH*, the precise OER mechanism in NiOOH catalysts co-doped with Fe and Mn remains incompletely elucidated. Key mechanistic questions persist, including the exact location of active sites responsible for O* and OOH* formation, as well as the atomic-scale structural configuration of the Fe-Mn dopant motif.
6. What impact does the increase in Fe content have on the structure of the catalyst? Will the increase in Mn content also enhance the OER activity?
7. The electrical double layer plays a pivotal role in governing electrochemical reaction mechanisms. For example, the structural arrangement of interfacial water molecules significantly influences both the kinetics of water dissociation and the mass transport of key species (OH^- and O_2). The structural impact of metal dopant incorporation on interfacial water remains unconfirmed.

Minor comment:
The captions for Figures 1d and 1e are missing.

Version 1:

Reviewer comments:

Reviewer #1

(Remarks to the Author)
The authors have well addressed the reviewers'concerns. Now it is ready for publication.

Reviewer #2

(Remarks to the Author)
Although authors have performed the revision carefully, to make the manuscript complete, a few additional data may be provided in the revised manuscript. The specific comments are:
1.The authors should add the standard diffraction data card in the PXRD data presented in the supplementary file.
2. Authors have explained the *OO evolution in addition to Ni^{4+} to Ni^{3+} conversion. It is still unclear from the scheme and also from the text how this step is performed for calculation. Provide some detailed explanation of the relevant steps.

3. In figure 3b, how did the author explain the initial discharging current. What does it contribute to? An explanation will be better to visualize the results obtained.
4. What is the calculated (actual) Faradaic efficiency of the proposed method? A detailing would be beneficial for the readers. Please include it.

Reviewer #3

(Remarks to the Author)
The authors have addressed all the comments.

Open Access This Peer Review File is licensed under a Creative Commons Attribution 4.0 International License, which permits use, sharing, adaptation, distribution and reproduction in any medium or format, as long as you give appropriate credit to the original author(s) and the source, provide a link to the Creative Commons license, and indicate if changes were made.
In cases where reviewers are anonymous, credit should be given to 'Anonymous Referee' and the source.
The images or other third party material in this Peer Review File are included in the article's Creative Commons license, unless indicated otherwise in a credit line to the material. If material is not included in the article's Creative Commons license and your intended use is not permitted by statutory regulation or exceeds the permitted use, you will need to obtain permission directly from the copyright holder.
To view a copy of this license, visit https://creativecommons.org/licenses/by/4.0/

Response to referees

We thank the three referees for taking the time to carefully review the manuscript and for giving many useful suggestions. The manuscript has been revised according to their comments and the changes have been highlighted in the revised version. We feel the quality of the paper has been improved greatly thanks to the input from the referees. Below is a point-by-point response.

Reviewer #1

Remarks to the Author: This manuscript presents a novel and potentially impactful electrochemical methodology, termed Open-Circuit Voltage Pulse Voltammetry (OCV-PV), to quantitatively decouple the electron transfer steps in the oxygen evolution reaction (OER). By isolating the rate of *OOH formation that widely regarded as the rate-determining step (RDS) from the total current, the authors propose a new kinetic descriptor. The combination of this kinetic analysis with in situ XAS and ATR-SEIRAS provides a compelling narrative that clarifies the distinct roles of Fe and Mn dopants in Ni-based oxyhydroxides, leading to the rational design of a ternary NiFeMn catalyst. However, several critical issues must be addressed before it could be reconsidered for publication on the high-level journal like Nature Communications

1.The core premise of the OCV-PV method is that the reduction of Ni4+ during the OCV period is driven exclusively by electron transfer from electrolyte OH- ions to form *OOH. However, the authors used Carbon Cloth as the substrate. It is well-known that high-valence nickel species can oxidize carbon substrates under OER conditions, acting as a self-discharge pathway. The authors must rigorously rule out the contribution of carbon oxidation to the charge transfer during OCV.

Response: We thank the reviewer for this important comment. We agree that, in principle, high-valent Ni species may oxidize carbon substrates under sustained OER conditions and thereby provide a possible self-discharge pathway. However, such carbon oxidation is typically associated with prolonged anodic polarization at high current density and high potential. Typically, Yu et al demonstrated that with applied potential at 1.59 V vs. RHE, the ≈97% Faradaic efficiency of the N, S-CC electrode indicated that the anodic current mainly came from water oxidation rather than carbon oxidation. (*10.1002/advs.201800760*) In

contrast, the OCV–PV analysis in this work is performed under fundamentally different conditions: the external circuit is disconnected, no sustained current flows, and the OCV window is very short (0.8–1.2 s). Under such transient relaxation conditions, carbon oxidation is unlikely to make a measurable contribution to the observed charge transfer.

More importantly, we directly examined the carbon substrate by comparing the Raman spectra before and after the OCV–PV protocol (Figure R1-1). No discernible change was observed, indicating that the electronic structure of the carbon cloth remains essentially unchanged during the OCV process. This provides direct experimental evidence that the carbon substrate is not measurably oxidized under the conditions used here.

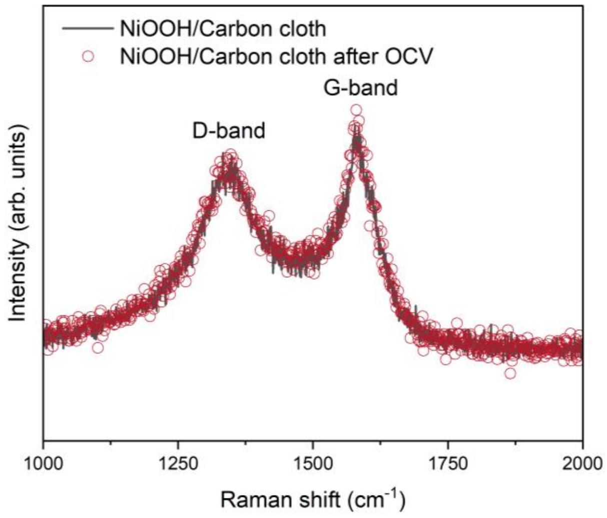

Figure R1-1. Raman spectra of NiOOH/Carbon cloth before and after OCV. (Supplementary Figure 16 in revised manuscript)

Taken together, the short OCV time scale, the absence of sustained anodic current, and the unchanged D-band/G-band in Raman spectra of carbon support that carbon oxidation does not contribute significantly to the OCV charge transfer in our measurements.

To address the comment, we have revised our manuscript as follows (line 257, page 8, highlighted in yellow): In addition, possible self-discharge through oxidation of the carbon-cloth substrate was examined by Raman spectroscopy before and after the OCV–PV test, and no detectable change was observed (Supplementary Figure 16), supporting that the measured OCV charge is not significantly contributed by carbon oxidation.

2.Ni K-edge XAS in transmission mode probes the bulk oxidation state. However, OER and the proposed *OOH formation are surface-confined processes. During OCV, the reduction of bulk Ni4+ to Ni3+ might be limited by proton diffusion from the electrolyte into the lattice, rather than solely by surface electron transfer kinetics. The authors need to discuss the influence of proton diffusion coefficients.

Response: We appreciate the reviewer's important point that Ni K-edge XAS in transmission mode reflects a bulk-averaged oxidation state, whereas OER and the proposed *OOH chemistry are surface-confined. In our workflow, the Ni K-edge signal is used to quantify the overall relaxation of oxidized Ni species during OCV, while the surface nature of the process is independently established by surface-sensitive probes, including in situ O K-edge XAS and ATR-SEIRAS.

We also acknowledge that proton diffusion/transport has been discussed in prior studies in the NiOOH/Ni$(OH)_2$ conversion process (*J. Electrochem. Soc. 117 729*, *J. Electrochem. Soc. 142 1401*). In the present case, however, our data do not support bulk proton diffusion as the dominant origin of the short-time Ni^{4+} to Ni^{3+} relaxation measured in OCV–PV. Specifically, even after 12 min of OCV relaxation, the bulk-sensitive EXAFS spectra show negligible structural change (Figure R1-2), indicating that bulk structural relaxation/proton-coupled conversion is highly sluggish under our conditions. If bulk proton diffusion were the dominant process governing the observed Ni^{4+} to Ni^{3+} relaxation during OCV, a more pronounced bulk structural response *i.e.* decrease of Ni-O bond length, would be expected on this much longer timescale. Its absence argues against bulk proton diffusion being the dominant rate-limiting process for the much shorter 1-2 s OCV window used for kinetic analysis.

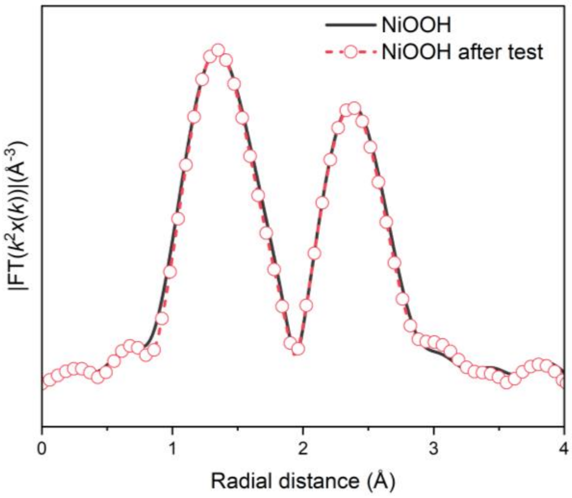

Figure R1-2. EXAFS spectra of NiOOH before and after in-situ Ni K-edge XAS characterization. (Supplementary Figure 11 in revised manuscript)

We therefore conclude that, although limited local proton redistribution during long-time relaxation cannot be completely excluded, the short-time OCV–PV kinetics mainly reflect an interfacial chemical relaxation process associated with surface *OOH formation, rather than proton-diffusion controlled bulk reduction.

To address the comment, we have revised our manuscript as follows (line 217, page 7, highlighted in yellow): In addition, although the OCV relaxation is accompanied by a decrease in the bulk-averaged Ni valence, no obvious structural change was observed in the bulk-sensitive EXAFS spectra even after prolonged OCV relaxation during in situ Ni K-edge characterization (Supplementary Figure 11), suggesting that bulk proton diffusion is unlikely to dominate the much shorter 1-2 s OCV window used for kinetic analysis.

3.While the manuscript focuses on kinetics, the rational design claim culminates in the NiFeMn catalyst. However, kinetic insights must translate into robust performance, while current manuscript lacks sufficient stability data.

Response: We thank the reviewer for this comment. We agree that kinetic insights should ultimately translate into robust catalytic performance. To address this point, we performed an additional stability test on the $Ni_{0.78}Fe_{0.20}Mn_{0.02}OOH$ catalyst, which is the optimized composition identified in our step-resolved design framework. As shown in Figure R1-3, the $Ni_{0.78}Fe_{0.20}Mn_{0.02}OOH$ catalyst maintains stable chronopotentiometric performance at 10 mA cm^{-2} over 100 h, supporting that the kinetically guided catalyst

design also delivers robust OER stability.

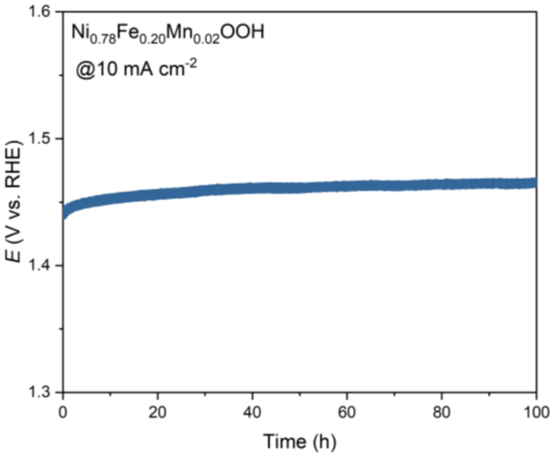

Figure R1-3. Stability test of $Ni_{0.78}Fe_{0.20}Mn_{0.02}OOH$ at 10 mA cm^{-2} for 100 h. (Supplementary Figure 39b in revised manuscript)

In addition, SEM-EDS analyses before and after the stability test show that the overall morphology and elemental distributions remain well preserved, while ICP analysis indicates that the Ni, Fe, and Mn contents change only negligibly after OER operation. These results collectively support the structural and compositional stability of $Ni_{0.78}Fe_{0.20}Mn_{0.02}OOH$ under the tested conditions.

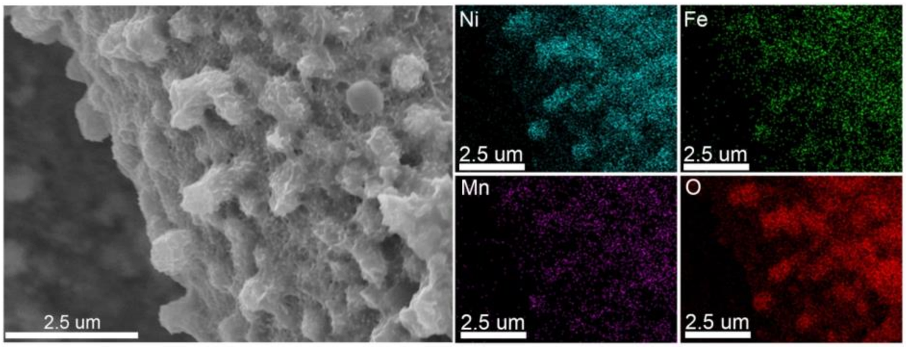

Figure R1-4. SEM-EDS images of $Ni_{0.78}Fe_{0.20}Mn_{0.02}$ sample before stability test. (Supplementary Figure 40 in revised manuscript)

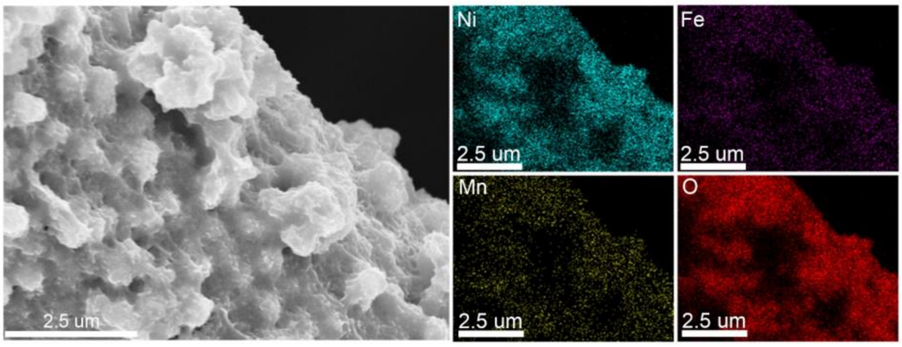

Figure R1-5. SEM-EDS images of $Ni_{0.78}Fe_{0.20}Mn_{0.02}$ sample after stability test. (Supplementary Figure 41 in revised manuscript)

Table R1-1. ICP-determined elemental contents of $Ni_{0.78}Fe_{0.20}Mn_{0.02}$ (Supplementary Table 1 in revised manuscript)

Element	Ni wt%	Fe wt%	Mn wt%
Pristine	78.8	19.3	1.9
After OER	78.6	19.4	2.0

To address the comment, we have revised our manuscript as follows (line 363, page 11, highlighted in yellow):

The $Ni_{0.78}Fe_{0.20}Mn_{0.02}OOH$ catalyst maintains stable chronopotentiometric performance at 10 mA cm^{-2} over 100 h without element segregation or dissolution, supporting that the kinetically guided catalyst design also delivers robust OER stability (Supplementary Figure 39b, 40-41 and Table 1).

4.Is it possible that radical recombination (e.g., 2 *OH = H2O + *O) contributes to the charge transfer during OCV?

Response: We appreciate the reviewer's suggestion regarding potential radical recombination pathways (e.g., 2*OH → H2O + *O). Such a process represents a surface chemical rearrangement and does not involve net electron transfer from the electrolyte to the catalyst. Therefore, it cannot account for the OCV-induced reduction of oxidized Ni species quantified by the OCV–PV method.

Moreover, our surface-sensitive measurements during OCV are inconsistent with this pathway being dominant: *in situ* O K-edge XAS shows a progressive decrease of the $^{*}O$-related feature, indicating consumption of $^{*}O$ rather than generation, while ATR-SEIRAS reveals an increasing $^{*}OOH$-related band during OCV. These observations support that the dominant OCV-period process is electrolyte-derived electron transfer associated with $^{*}OOH$ formation ($^{*}O + OH^{-} \rightarrow {}^{*}OOH$), rather than radical recombination among adsorbates.

To address the comment, we have revised our manuscript as follows (line 255, page 8, highlighted in yellow): Possible adsorbate rearrangement pathways, such as $2^{*}OH \rightarrow H_2O + {}^{*}O$, are not expected to contribute to the measured OCV charge because they do not involve net electron transfer to oxidized Ni sites.

5.Please provide more details in the Methods section regarding the calibration of the DEMS signal. How was the claimed negligible contribution of O_2 evolution quantified in terms of molar sensitivity?

Response: We thank the reviewer for this comment. We agree that the original Methods section did not provide sufficient information regarding DEMS calibration and therefore did not adequately support quantitative assessment of the O_2 contribution during OCV. In the original measurements, the DEMS signal was used mainly as a qualitative indicator of O_2-related behavior. To address the reviewer's concern, we have now performed additional calibration measurements to establish an effective molar sensitivity for the m/z = 32 signal and reanalyzed the OCV-period DEMS data accordingly.

Specifically, after electrochemical activation, the catalyst was held at 1.503 V under steady OER conditions, and the electrochemical charge passed during the steady-state interval was converted to the corresponding O_2 amount using Faraday's law $n(O_2) = Q_{cell}/4F$ (Figure R1-6). The simultaneously recorded, baseline-corrected DEMS m/z = 32 signal was integrated over the same time window to obtain the detector response, from which an effective molar sensitivity was determined as k=3.7 mol/C. This calibration factor was then applied to the OCV-period DEMS signal to estimate the amount of O_2 detected within the OCV window.

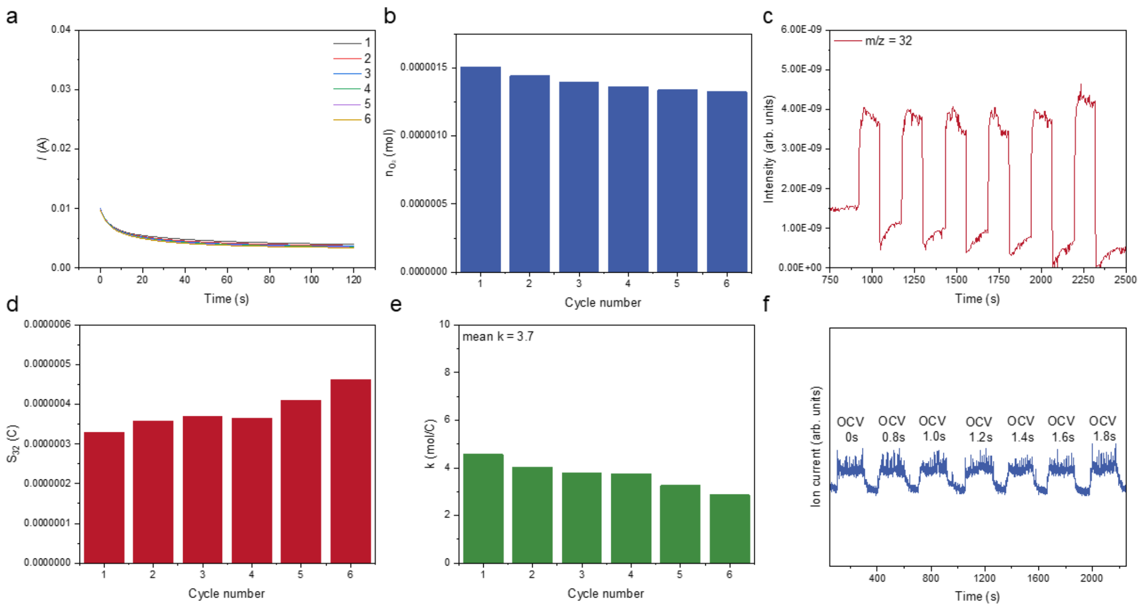

Figure R1-6. Calibration of the DEMS response and OCV-time-dependent DEMS measurements. (a) *I-t* curves recorded at 1.503 V vss. RHE for six repeated calibration cycles. (b) O_2 amount ($n(O_2)$) estimated from the integrated electrochemical charge in each calibration cycle. (c) Simultaneously recorded DEMS signal at m/z=32 during the calibration cycles. (d) Integrated baseline-corrected DEMS m/z=32 signal (S_{32}) for each calibration cycle. (e) Effective DEMS calibration factor k obtained from the corresponding electrochemical and DEMS integrals, with an average value of k=3.7 mol/C (f) DEMS m/z=32 signal recorded under identical pre-charging conditions while varying only the OCV duration (0, 0.8, 1.0, 1.2, 1.4, 1.6, and 1.8 s), used to evaluate the additional O_2 signal develops with increasing OCV time. (Supplementary Figure 15 in revised manuscript)

Using this calibration factor, we estimated the amount of O_2 detected within the OCV window at different OCV durations and converted it into the corresponding charge contribution associated with the *OO evolution. Comparison with the total OCV-induced charge quantified by OCV–PV, it shows that this contribution is much smaller and does not dominate the extracted kinetics (Figure R1-7).

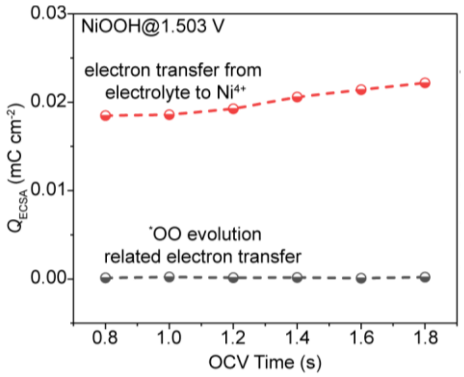

Figure R1-7. Estimated transferred charge normalized to the electrochemical surface area (ECSA) during the OCV procedure. The contribution of oxygen evolution to charge transfer was derived from *in situ* DEMS measurements. (Figure 3c in revised manuscript)

To address the comment, we have revised our manuscript as follows

(line 452, page 13, highlighted in yellow): **DEMS measurement.** Differential Electrochemical Mass Spectrometry (DEMS) was used to quantify the evolved oxygen molecules within the OCV window at different OCV durations during the OCV-PV measurement. Specifically, after electrochemical activation, the catalyst was held at 1.503 V under steady OER conditions, and the electrochemical charge passed during the steady-state interval was converted to the corresponding O_2 amount using Faraday's law $n(O_2) = Q_{cell}/4F$ (Supplementary Figure 15). The simultaneously recorded, baseline-corrected DEMS m/z = 32 signal was integrated over the same time window to obtain the detector response, from which an effective molar sensitivity was determined as k=3.7 mol/C. This calibration factor was then applied to the OCV-period DEMS signal to estimate the amount of O_2 detected within the OCV window.

(line 247, page 8, highlighted in yellow): Moreover, the contribution of the *OO evolution step was determined using *in situ* DEMS (details on the DEMS procedure are provided in the Methods section). As shown in Figure 3c and Supplementary Figures 15, compared with the total charge obtained from the OCV–PV measurement, the recorded charge associated with *OO evolution is significantly lower and hence can be ignored

6.Please supply XRD patterns for the doped samples (NiFe, NiMn, etc.) in the Supplementary Information to confirm that 2% doping did not induce phase segregation or impurity phases.

Response: We thank the reviewer for this comment. We have now included the XRD patterns of all doped

samples (NiFe, NiMn, NiCo, NiCr, NiV, etc.) in the Supplementary Information (Figure R1-8). All samples exhibit diffraction features consistent with the parent $Ni(OH)_2$ phase. No additional diffraction peaks corresponding to possible impurity phases (e.g., FeOx, MnOx, CoOx, etc.) are detected. Meanwhile, no evidence of phase segregation is observed. The absence of peak splitting or new reflections indicates that 2% dopants are structurally incorporated without forming secondary crystalline phases.

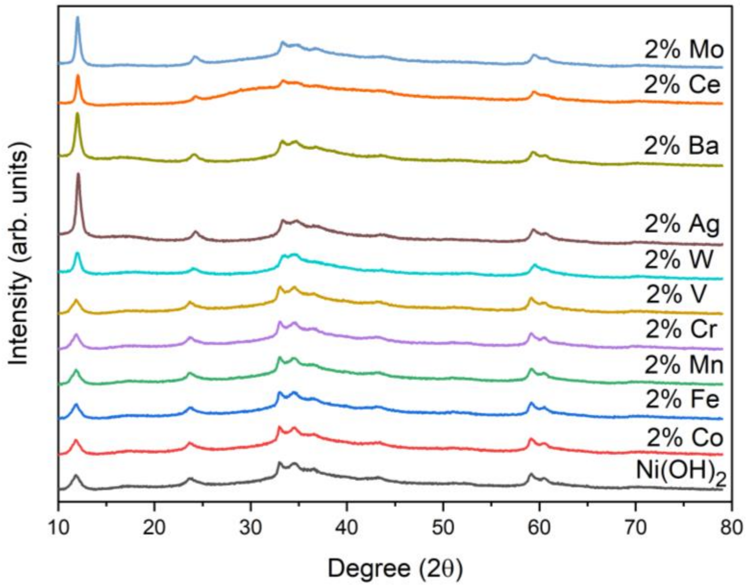

Figure R1-8. XRD patterns of $Ni(OH)_2$-based catalysts with various cation dopants (2 at%).

(Supplementary Figure 21 in revised manuscript)

To address the comment, we have revised our manuscript as follows (line 308, page 9, highlighted in yellow):

XRD patterns of all 2 at% doped samples show diffraction features consistent with the parent $Ni(OH)_2$ phase, without detectable impurity peaks or phase segregation (Supplementary Figure 21).

Reviewer #2

Remarks to the Author: In the paper entitled “Decoupling electron transfer defines a quantitative kinetic framework for oxygen evolution catalysis”, the authors have presented a nice, conceptually strong work. It presents a clear and well-designed OCV–PV strategy to quantify *OOH formation kinetics, and the approach is experimentally grounded in understanding the OER kinetics. There are some queries to be addressed before proceeding with publication.

Comment 1: What is the thermodynamic driving force for continued *OOH formation under open-circuit conditions? How can it be compared to the *OOH formation under applied-bias OER?

Response: We thank the reviewer for this important question. The *OOH formation observed under OCV does not originate from an external electrochemical potential, but from the intrinsic free-energy difference between a highly oxidized, pre-charged NiOOH surface and its relaxed state. In the OCV–PV protocol, the preceding charging step deliberately generates a metastable surface enriched in Ni^{4+} species (Figure R2-1b). These oxidized Ni centers possess an elevated chemical potential and are thermodynamically driven to relax toward Ni^{3+}. Under open-circuit conditions, this relaxation cannot proceed through the external circuit and therefore occurs via electrolyte-coupled interfacial reactions. In particular, the reaction $^*O + OH^- \rightarrow {}^*OOH$ provides an electron that is locally accommodated by neighboring Ni^{4+} sites, reducing them to Ni^{3+}. Thus, the thermodynamic driving force for *OOH formation under OCV is the spontaneous reduction of high-valence Ni species generated during the pre-charging step.

Our DFT calculations further support this mechanism, showing that the charged NiOOH surface generates an internal electric field that lowers the free-energy barrier for *OOH formation, rendering the process thermodynamically and kinetically accessible even in the absence of sustained applied bias (Figure R2-1d and e).

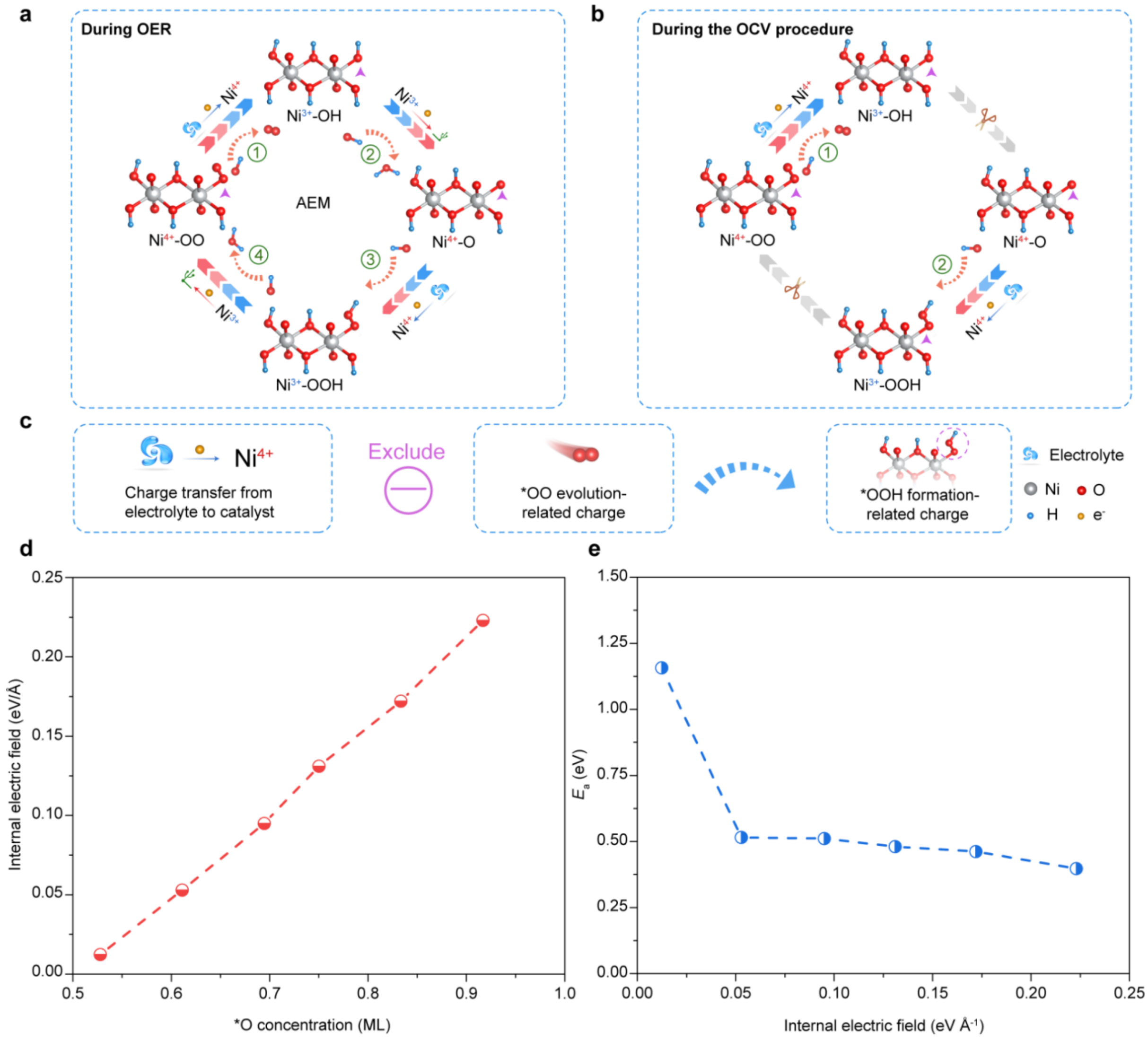

Figure R2-1. Theoretical analysis for quantifying the *OOH formation rate. **a** Schematic of the adsorbate evolution mechanism (AEM) pathway, in which a series of surface-bound oxygen intermediates such as *OH, *O, *OOH, and *OO are sequentially formed. **b** Schematic of electron transfer upon conducting an open-circuit voltage (OCV) procedure. The scissor icon indicates that electron transfer from the electrocatalyst to the external circuit is blocked. **c** Schematic of the method used to selectively isolate the *OOH formation-related charge from the total charge transferred from the electrolyte to the catalyst. **d** The effect of *O concentration on internal electric field. **e** The effect of internal electric field on *OOH formation energy barrier. (Figure 1 in revised manuscript)

We emphasize that *OOH formation under OCV is fundamentally distinct from steady-state OER under applied bias. Applied-bias OER is driven by an external electrochemical potential and proceeds under steady-state conditions, whereas OCV probes the short-time relaxation of a pre-charged, metastable surface. By

restricting the OCV window to 1–2 s, we operate in the initial-rate regime (t → 0 limit) of the relaxation process, where the measured rate reflects the intrinsic interfacial kinetics of $^{*}O + OH^{-} \rightarrow {}^{*}OOH$ prior to significant mass transport or bulk re-equilibration. The linear dependence of ΔQ on OCV duration within this window confirms reaction-controlled behavior. Although the absolute rates under OCV are not directly comparable to steady-state OER currents, the short-time approximation enables meaningful comparison of intrinsic $^{*}OOH$ formation kinetics and their dependence on catalyst composition and pre-charging potential.

To address the comment, we have revised our manuscript as follows

(line 133, page 4, highlighted in yellow):

Therefore, the continued $^{*}OOH$ formation under OCV is thermodynamically driven by the spontaneous relaxation of the pre-charged, highly oxidized NiOOH surface toward a lower-energy state with reduced Ni valence, rather than by a sustained external bias.

(line 237, page 7, highlighted in yellow):

Here, the $^{*}OOH$ formation rate measured under OCV should be understood as the initial relaxation kinetics of a pre-charged metastable surface under defined initial conditions, rather than the steady-state $^{*}OOH$ formation rate under continuously applied bias.

Comment 2: Even after voltage shutdown, residual charge or the slow discharge electrode during OCV might be assisting/contributing to *OOH formation.

Response: We thank the reviewer for this important point. We agree that residual double-layer charge or slow electrode discharge could, in principle, contribute to transient relaxation immediately after voltage shutdown. However, several considerations indicate that the OCV-induced $^{*}OOH$ formation observed in our experiments is not driven by residual discharge.

First, $^{*}OOH$ formation under alkaline conditions involves electron transfer from electrolyte-derived OH^{-} species to oxidized Ni^{4+} sites, corresponding to electron flow into the electrode. In contrast, residual capacitive discharge corresponds to electron release from the electrode. The direction of electron flow required for $^{*}OOH$ formation is therefore opposite to that associated with electrode discharge. Residual discharge cannot supply the electrons required for the observed reaction.

Second, if capacitive discharge were dominant, the accumulated charge during OCV would follow an

exponential RC-type decay. Instead, the experimentally measured ΔQ increases linearly with OCV duration within the 1–2 s window, consistent with a reaction-controlled process rather than passive discharge.

Third, the magnitude of ΔQ depends systematically on the pre-charging potential and catalyst composition, indicating that the relaxation kinetics are governed by the oxidized surface state rather than by non-specific capacitive effects.

Taken together, these observations support that the OCV-induced charge reflects chemical relaxation of the pre-charged NiOOH surface via interfacial *OOH formation, rather than slow electrode discharge.

To address the comment, we have revised our manuscript as follows (line 266, page 8, highlighted in yellow):

Notably, the approximately linear increase of Q_{ECSA} with OCV duration within this short time window is inconsistent with a simple residual capacitive-discharge process and instead supports a reaction-controlled chemical relaxation of the pre-charged NiOOH surface.

Comment 3: The 1163 cm^{-1} peak from ATR-SEIRAS, assigned to *OOH intermediate formed during OER, can also have an overlap with C-O stretching, O-H bending, or electrolyte-related vibrational modes. How do the authors rule out the interference?

Response: We thank the reviewer for the important comment. To address the potential overlap in the 1000–1200 cm^{-1} region, we performed multiple control analyses to rule out interference from substrate or electrolyte-related vibrations.

First, under identical ATR-SEIRAS conditions, the initial state (without applied bias) does not exhibit a discernible band at 1163 cm^{-1} (Fig.R2-2a). Meanwhile, no intermediate evolution is observed for bare carbon cloth under applied bias. These observations exclude a static C–O vibration of the carbon support or a background electrolyte absorption as the origin of this band (Fig.R2-2b).

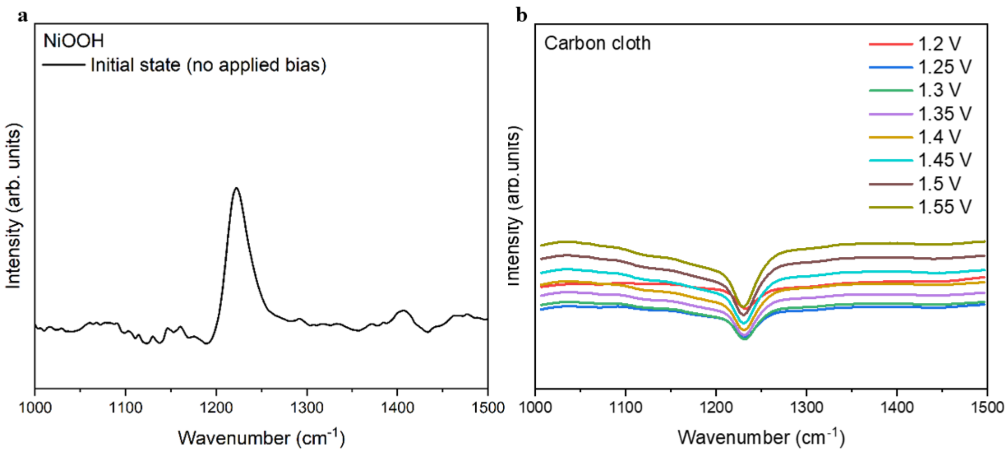

Figure R2-2. ATR-SEIRAS characterization. **a** NiOOH initial state without applied bias. **b** Bare carbon cloth. (Supplementary Figure 10 in revised manuscript)

Second, the 1163 cm^{-1} feature does not correspond to O–H stretching, which typically appears in the 3000–3700 cm^{-1} region (as evidenced by the *OH stretching band at 3729 cm^{-1} in Figure R2-3).

[FIGURE REDACTED]

Figure R2-3. Operando synchrotron FTIR spectra recorded in the potential range of 1.2–1.6 V versus RHE for 12Ru/MnO_2 (Figure 6b, *Nature Catalysis volume 4, pages1012–1023 (2021)*)

Importantly, the evolution of the 1163 cm^{-1} band correlates with the concurrent consumption of *O species to form *OOH observed by in situ O K-edge XAS and with the reduction of oxidized Ni species during OCV (Figure R2-4 and R2-5). The synchronized evolution across independent spectroscopic probes strongly supports assignment of the 1163 cm^{-1} feature to *OOH intermediate.

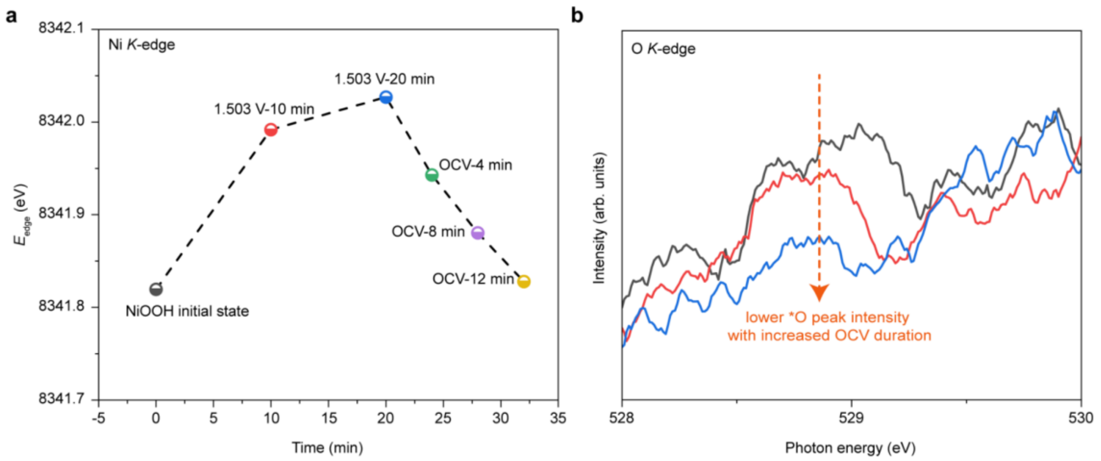

Figure R2-4. Continued formation of *OOH during the OCV. **a** Photon energy of the half-height position (E_{edge}) of NiOOH obtained from the normalized *in situ* Ni *K*-edge XAS spectra (Supplementary Figure 6) under charging and OCV procedures. **b** *In situ* O *K*-edge XAS spectra of the change in *O peak intensity for NiOOH during the OCV procedure. (Figure 2 in revised manuscript)

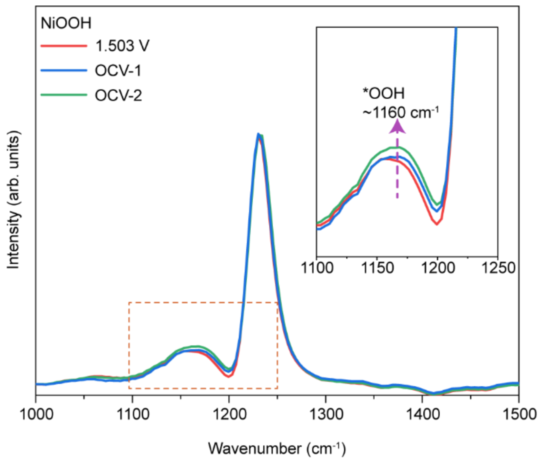

Figure R2-5. *In situ* ATR-SEIRAS characterization on NiOOH under applied potentials of 1.403 V, 1.503 V, and following OCV procedure. (Supplementary Figure 9 in revised manuscript)

Taken together, these control experiments and cross-validation results indicate that the 1163 cm^{-1} band is predominantly associated with a surface *OOH-related intermediate rather than *OH or substrate/electrolyte-derived vibrations.

To address the comment, we have revised our manuscript as follows (line 180, page 6, highlighted in yellow):

A prominent peak at 1163 cm^{-1} was detected in the ATR-SEIRAS spectra by charging NiOOH at 1.503 V vs. RHE, which can be attributed to *OOH intermediate formed during OER (Supplementary Figure 9). Upon

introducing the OCV procedure, the $^*OOH$-related peak intensity progressively increased, providing compelling evidence for the continuous formation and accumulation of $^*OOH$ intermediates under open-circuit conditions. Notably, this feature is absent in the initial state without applied bias or in the bare carbon cloth under applied bias (Supplementary Figure 10), excluding a static contribution from the substrate or background electrolyte. Therefore, combining the *in situ* Ni *K*-edge, O *K*-edge and ATR-SEIRAS results, we successfully reveal that $^*OOH$ can form and persist on charged NiOOH by accepting electrons from OH^- during the OCV procedure.

Comment 4: Would the same OCV-driven *OOH formation be observed in electrolytes other than 1 M KOH or in other transition-metal oxyhydroxides?

Response: We thank the reviewer for raising this important question regarding generality. The OCV-driven $^*OOH$ formation observed in our study originates from chemical relaxation of a pre-charged, high-valent oxyhydroxide surface under alkaline conditions.

In our NiOOH system under alkaline conditions, this process is understood within the adsorbate evolution mechanism (AEM), in which $^*OOH$ is a required reaction intermediate. From this perspective, the OCV-driven $^*OOH$ formation is not specific to 1 M KOH itself, but rather linked to catalytic systems that operate through an AEM-type pathway and can reach oxidized surface states after anodic polarization. Therefore, for other catalysts that also follow AEM pathway—such as acidic IrO_2-based OER catalysts—an analogous OCV-driven relaxation through $^*OOH$ formation should in principle also be possible.

Regarding catalyst generality, the OCV-driven relaxation is not specific to NiOOH, but to systems capable of forming high-valent metal–oxygen species under anodic polarization within an AEM framework. Transition-metal oxyhydroxides such as CoOOH, which also exhibit reversible high-valent surface states and follow AEM-type pathways, are therefore expected to display analogous behavior. To examine generality, we performed OCV–PV measurements on CoOOH with the charging bias at 1.503 V vs. RHE (Figure R2-6). Similar qualitative OCV-induced charge evolution was observed, supporting that the relaxation mechanism is not unique to NiOOH. Therefore, while the quantitative kinetics may vary across systems, the underlying OCV-induced relaxation framework should be broadly applicable to catalysts operating via AEM-type mechanism.

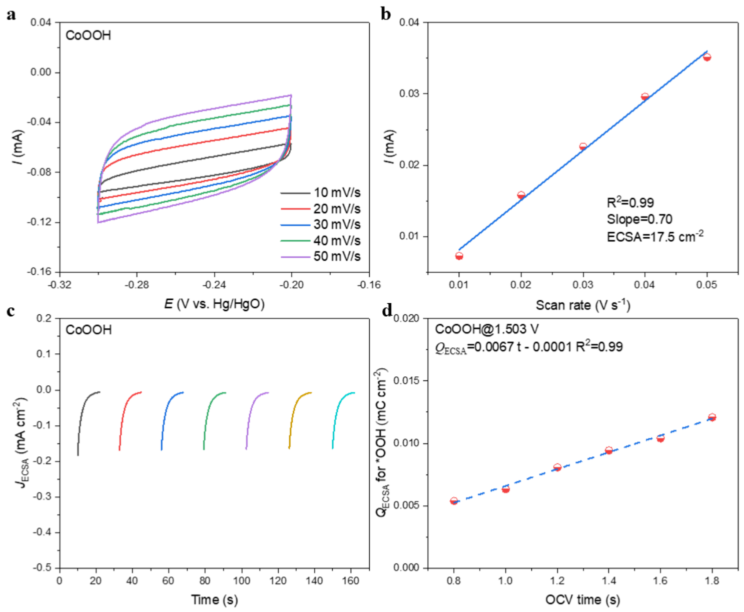

Figure R2-6. OCV–PV measurement on CoOOH. (a) The Cyclic voltammograms curves of the CoOOH and corresponding current (*I*) vs. scan rate plots (b). Plots of current vs. scan rate data were obtained from CV curves at -0.25 V vs. Hg/HgO. (c) The reductive pulse with the current response in OCV-PV test for CoOOH under charging bias of 1.503 V. (d) *OOH formation-related charge versus OCV duration. (Supplementary Figure 20 in revised manuscript)

To address the comment, we have revised our manuscript as follows (line 284, page 8, highlighted in yellow): To preliminarily examine the generality of the OCV-driven relaxation framework, we further performed OCV–PV measurements on CoOOH under an identical charging bias. A similar qualitative OCV-induced charge evolution was observed (Supplementary Figure 20), suggesting that the present framework is not unique to NiOOH and may be applicable to other transition-metal oxyhydroxides capable of forming high-valent surface states under OER conditions.

Comment 5: The authors claim that "each *OOH formation event corresponds to the acceptance of two electrons by the Ni4+ sites during the OCV process." This statement, although scientifically deep, requires a clearer explanation of how the redistribution of electrons internally (Ni4+ → Ni3+) under OCV occurs.

Response: We thank the reviewer for the important comment.

Under alkaline AEM conditions, *OOH formation proceeds via a proton-coupled electron-transfer step:

$$^{*}O + OH^{-} \rightarrow {}^{*}OOH + e^{-}$$

During applied bias, the generated electron can be transported through the external circuit. However, under open-circuit conditions, electron flow to the external circuit is blocked. Consequently, the electron produced during *OOH formation must be accommodated locally within the catalyst framework.

Prior anodic polarization generates a population of high-valent Ni^{4+} sites. These oxidized sites serve as internal electron acceptors during OCV relaxation. The electron generated from the interfacial PCET step is therefore captured by neighboring Ni^{4+} species, reducing them to Ni^{3+}. In this sense, *OOH formation under OCV is directly coupled to the reduction of high-valent Ni sites within the oxyhydroxide lattice. The statement that each *OOH formation event corresponds to electron acceptance by Ni^{4+} refers to this local redox compensation mechanism, rather than to electron transfer through the external circuit. We have revised the manuscript to clarify that the electron redistribution occurs internally within the Ni–O network during OCV relaxation.

To address the reviewer's concern, we have revised the relevant text in the manuscript as follows (line 228, page 7, highlighted in yellow):

Generally, *OOH formation proceeds via the reaction $^{*}O + OH^{-} \rightarrow {}^{*}OOH + e^{-}$, in which one electron is generated during the interfacial proton-coupled electron-transfer step. Under an applied bias, this electron can be transferred to the external circuit[7,8]. Under OCV conditions, however, electron transfer to the external circuit is blocked. Therefore, the electron generated from *OOH formation must be redistributed locally within the catalyst and can be accommodated by nearby high-valent Ni^{4+} species, reducing them to Ni^{3+}. In this sense, *OOH formation under OCV is coupled to an internal redox-compensation process. Accordingly, one *OOH formation event during OCV is reflected electrochemically as a two-electron reduction of Ni^{4+} species. Consequently, half of the charge difference between the two PV cycles $[(Q_1\text{-}Q_2)/2)]$ correlates to the amount of *OOH formed.

Comment 6: Authors state that OCV–PV and PV measurements decouple electrolyte → catalyst and catalyst → circuit electron transfer. However, the dopants may alter the mechanisms and, hence, the later steps by altering the conductivity, introducing defects, or altering the catalyst's surface reconstruction. How is this approach valid for all dopants? Also, are all dopants (V, Cr, Mn, Fe, Co, Cu, Zn, Ce) truly substitutional?

Response: We thank the reviewer for this insightful comment. We fully agree that dopants may influence

conductivity, defect density, or surface reconstruction, which in turn can modify overall catalytic performance. However, the validity of the OCV–PV/PV framework does not require identical electronic transport properties or reconstruction behavior across dopants.

The OCV–PV methodology relies on a more fundamental condition: the separation of electron flow directions under open-circuit versus applied-bias conditions. During OCV, electron transfer to the external circuit is suppressed, and any interfacial PCET step must be compensated internally within the catalyst lattice. During PV, electron transfer proceeds through the external circuit. As long as the reaction proceeds via surface adsorbate intermediates (i.e., an AEM-type pathway), the directional decoupling of electrolyte → catalyst and catalyst → circuit electron transfer remains physically well-defined.

Here, our study deliberately employs dilute doping (~2 at%) to minimize the likelihood of introducing a new dominant reaction framework. While dopants can, in principle, alter the reaction pathway, previous computational studies have shown that substantial modification of oxygen electronic structure and a transition toward lattice oxygen participation typically require significantly higher dopant concentrations. For example, Xu et al. reported that in CoZn systems, lattice oxygen mechanism (LOM) dominance emerges only at elevated Zn contents (~20%), where sufficient nonbonding oxygen states are introduced near the Fermi level. (*Nature Energy, 2019, 4(4): 329-338.*) In contrast, the dilute doping level used here is an order of magnitude lower and is therefore less likely to induce a mechanistic shift of this nature. While we cannot fully exclude minor contributions from alternative pathways under certain conditions, the electrochemical behavior and the step-resolved trends extracted from OCV–PV and PV remain internally consistent across the dopant series, supporting the applicability of the approach for comparative kinetics within an AEM-type framework.

Regarding dopant incorporation, XRD shows no additional reflections attributable to secondary crystalline phases, consistent with predominantly substitutional/dilute incorporation at this low concentration (within the detection limit of laboratory XRD).

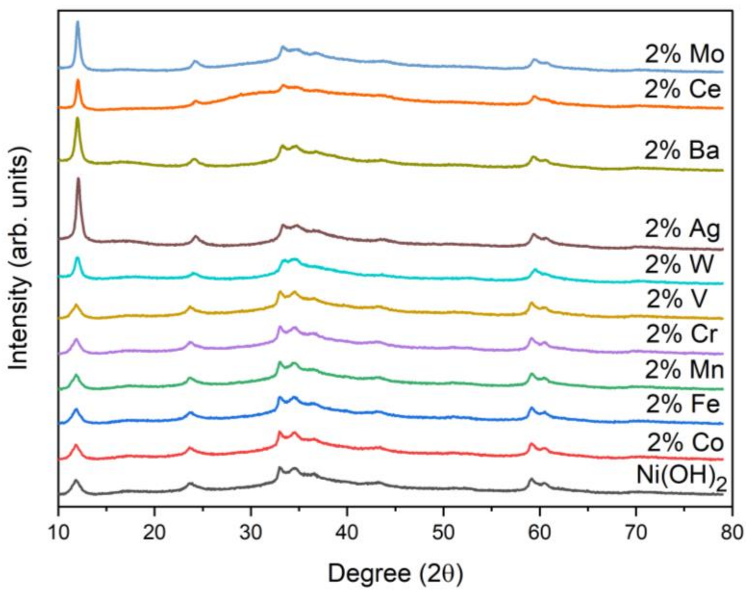

Figure R2-7. XRD patterns of $Ni(OH)_2$-based catalysts with various cation dopants (2 at%).

(Supplementary Figure 21 in revised manuscript)

To address the comment, we have revised our manuscript as follows (line 308, page 9, highlighted in yellow): XRD patterns of all 2 at% doped samples show diffraction features consistent with the parent $Ni(OH)_2$ phase, without detectable impurity peaks or phase segregation (Supplementary Figure 21). We note that different dopants may also influence conductivity, defect density, and surface reconstruction behavior. Therefore, the present OCV–PV/PV framework is not intended to assume identical microscopic properties across all doped catalysts. Rather, it is used to compare the apparent step-specific kinetics under identical testing conditions.

Comment 7: On what basis is the potential of 1.503 used to perform the analysis?

Response: We thank the reviewer for this question. The potential of 1.503 V was selected as a fixed comparison point for the dopant series rather than as a unique mechanistic threshold. At this potential, all doped NiOOH catalysts are in the OER-relevant region and the NiOOH surface is sufficiently oxidized, which allows the OCV–PV measurements to resolve dopant-dependent differences in *OOH formation kinetics under identical electrochemical conditions. Using the same potential for all samples is important because it ensures that the extracted differences arise primarily from catalyst-dependent kinetic behavior, rather than from variations in the applied potential. Therefore, 1.503 V was chosen as a representative and stable operating point for systematic comparison across the dopant series.

To address the comment, we have revised our manuscript as follows (line 314, page 9, highlighted in yellow): A fixed potential of 1.503 V was chosen here as a representative comparison point because it lies in the OER-relevant region where all of the doped NiOOH catalysts are electrochemically active, while still providing a sufficiently oxidized surface state for resolving dopant-dependent differences in step-specific kinetics under identical conditions.

Comment 8: In Figure 1, the captions for Figures d and e are missing.

Response: We thank the reviewer for this comment. We have revised this in the manuscript. (line 109, page 4, highlighted in yellow): **d** The effect of $^{*}O$ concentration on internal electric field. **e** The effect of internal electric field on $^{*}OOH$ formation energy barrier

Reviewer #3

Remarks to the Author: This study introduces a novel electrochemical methodology (OCV-PV) to quantitatively decouple the kinetics of elementary steps in the oxygen evolution reaction (OER): the deprotonation of OH* and the formation of OOH* intermediates. By applying this approach, the authors systematically elucidate how distinct dopant elements modulate the rates of these elementary steps on the NiOOH catalyst surface. Notably, Fe doping predominantly accelerates OOH* formation, whereas Mn doping enhances OH* deprotonation. Leveraging these mechanistic insights, the authors rationally design a high-performance FeMn co-doped NiOOH catalyst that enables targeted kinetic regulation of both elementary steps, resulting in substantially improved OER activity. These findings may arouse interest in related fields, but several issues need to be considered:

1. How can the influence of deprotonation of OOH*, and OH- (or O2) mass transfer limitations on OER kinetics be excluded?

Response: We thank the reviewer for this important question.

First, the OCV–PV analysis probes the formation of *OOH rather than its subsequent deprotonation. The *OOH → *OO step requires electron transfer through the external circuit under anodic bias. During OCV, the external circuit is disconnected and electron flow to the circuit is blocked. Therefore, the deprotonation of *OOH cannot proceed as a sustained electrochemical step under OCV conditions.

Second, mass-transport limitations of OH^- or O_2 are unlikely under the present conditions. The use of 1 M KOH provides abundant OH^- supply, minimizing the possibility of reactant depletion near the electrode surface. Meanwhile, the electrolyte was continuously stirred during the measurements, which further helps suppress concentration gradients near the electrode/electrolyte interface. In addition, mass-transport limitations typically arise under sustained OER currents where O_2 accumulation occurs. In contrast, the OCV window corresponds to a transient relaxation regime without continuous oxygen evolution, making significant O_2 buildup unlikely. During OCV-PV test, the short OCV time window (0.8-1.8 s) ensures that the measured charge evolution reflects initial reaction kinetics rather than diffusion-limited processes. Experimentally, the transferred charge increases approximately linearly with time within this window, which is characteristic of a reaction-controlled regime rather than diffusion-limited behavior.

Taken together, these considerations indicate that the kinetics extracted from the OCV–PV protocol primarily reflect the intrinsic interfacial $^*OOH$ formation step rather than deprotonation or mass transport limitations.

To address the comment, we have revised our manuscript as follows

(line 263, page 8, highlighted in yellow): In addition, because all measurements were conducted in 1 M KOH and within a short OCV window, significant OH^- depletion or O_2 accumulation is unlikely under the present conditions, supporting that the extracted kinetics are not dominated by mass-transport limitation.

(line 263, page 8, highlighted in yellow): The electrolyte was continuously stirred during the measurements.

2. Does the proposed OCV method under investigation enable accurate quantification of the OOH* formation rate? Specifically, can it adequately account for the contributions of OH- adsorption and O2* desorption to this kinetic step? Given that surface O* concentration serves as a critical descriptor in contemporary mechanistic studies, to what extent might the lattice oxygen participation mechanism influence the overall reaction pathway?

Response: We thank the reviewer for this comment.

In our work, the OCV–PV protocol is designed to extract the initial-rate ($t \rightarrow 0$) charge consumption associated with chemical relaxation of a pre-charged, oxidized surface. It therefore provides an apparent OOH formation rate under a defined initial surface condition, rather than an absolute elementary-step rate constant independent of surface coverage. During the OCV–PV test, the pre-charging step establishes a reproducible oxidized surface state with a high population of high-valent Ni sites. By restricting the OCV window to 0.8–1.8 s, we operate in the initial-rate regime where ΔQ increases linearly with time, indicating reaction-controlled behavior. Under these conditions, the extracted rate reflects the interfacial PCET process leading to $^*OOH$ accumulation. This mechanistic interpretation is consistent with independent operando spectroscopy (ATR-SEIRAS and XAS), which verifies the presence of $^*OOH$-related intermediates and the concurrent reduction of oxidized Ni species during OCV relaxation.

Accordingly, we have revised our manuscript as follows (line 237, page 7, highlighted in yellow): Here, the

*OOH formation rate measured under OCV should be understood as the initial relaxation kinetics of a pre-charged metastable surface under defined initial conditions, rather than the steady-state *OOH formation rate under continuously applied bias.

Role of OH^{-} adsorption and O_2 desorption. The OH^{-} adsorption is intrinsically coupled to *OOH formation on a real surface and therefore is not treated as a fully separable process in the present OCV–PV measurement. In this sense, the OCV–PV protocol does not isolate a purely coverage-independent elementary rate constant, but rather probes the apparent initial kinetics of *OOH accumulation under a defined pre-charged surface state, including the interfacial OH^{-} attack/adsorption involved in the $^{*}O + OH^{-} \rightarrow {}^{*}OOH$ step. Moreover, O_2 desorption is associated with oxygen evolution and requires sustained electrochemical driving; under open circuit, continuous O_2 release is not expected to dominate within the short OCV window, which has also been validated through DEMS characterization (Figure R3-1). Therefore, while these processes may exist to some extent, they do not account for the dominant ΔQ signal used to quantify the initial relaxation kinetics.

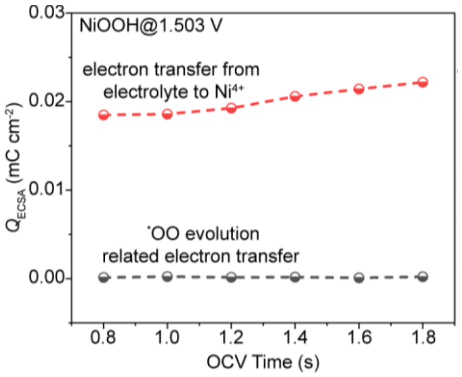

Figure R3-1. Estimated transferred charge normalized to the electrochemical surface area (ECSA) during the OCV procedure. The contribution of oxygen evolution to charge transfer was derived from *in situ* DEMS measurements. (Figure 3c in revised manuscript)

Accordingly, we have revised our manuscript as follows (line 252, page 8, highlighted in yellow):

We note that the extracted OCV charge should therefore be understood as the apparent initial kinetics of *OOH formation, which intrinsically includes the interfacial OH^{-} attack/adsorption involved in the $^{*}O + OH^{-} \rightarrow {}^{*}OOH$ step, whereas the contribution from the *OO evolution is negligible under the present conditions.

Given that surface O* concentration serves as a critical descriptor in contemporary mechanistic studies, to what extent might the lattice oxygen participation mechanism influence the overall reaction pathway?

We agree that surface *O coverage is an important mechanistic descriptor. Regarding the possible influence of lattice oxygen participation, we note that the OCV–PV framework developed here is based on a Ni-centered metal-redox/AEM description, in which the Ni valence changes between Ni^{3+} and Ni^{4+} are explicitly used to track the elementary charge-transfer processes. Therefore, in theory, the current OCV–PV method is not designed to independently resolve or quantify the contribution from an oxygen-redox/lattice-oxygen pathway, which follows a fundamentally different reaction picture from the Ni-centered adsorbate evolution mechanism. In addition, activation of oxygen redox generally requires the emergence of non-bonding oxygen states near the Fermi level. Based on the current understanding of NiOOH, the OER pathway is more commonly interpreted within a Ni-centered redox framework where the metal states is around the Fermi level (*Nature, 2022, 611(7937): 702-708.*); therefore, extensive oxygen-redox/lattice-oxygen participation is considered unlikely under the present conditions. Under the present conditions, we therefore interpret the measured kinetics within the AEM framework.

Accordingly, we have revised our manuscript as follows (line 280, page 8, highlighted in yellow): We note that the present OCV–PV analysis is interpreted within a Ni-centered AEM framework, consistent with the current understanding of NiOOH-based OER, and is not designed to independently resolve contributions from oxygen-redox pathways, which would require dedicated *operando* probes for direct identification.

3. The DFT calculations presented in Figure 1 do not substantiate the efficacy of the proposed OCV method. In my opinion, they primarily elucidate the dependence of the theoretical activation energy (Ea) for elementary reaction steps on the surface coverage of O*. Furthermore, when the O* coverage exceeds 0.5, the variation in Ea becomes marginal.

Response: We thank the reviewer for this comment. We agree that the DFT calculations in Figure 1 are not intended to directly validate the OCV method itself. Their main purpose is to provide theoretical support for the chemical plausibility of the proposed process, namely that charged Ni sites are favorable for *OOH formation during the OCV period. In particular, the DFT results show that increasing surface *O coverage, which reflects a more highly charged NiOOH surface, lowers the energy barrier for *OOH formation and thus suggests that *OOH formation can remain kinetically accessible on charged NiOOH under OCV conditions.

The basis for proposing and validating the OCV method comes from the operando experimental evidence rather than from DFT alone. Specifically, the in situ Ni K-edge XAS shows that the Ni valence decreases during OCV, indicating continued electron transfer from OH^- to charged Ni sites. Meanwhile, the in situ O K-edge XAS reveals the gradual consumption of $^*O$ during OCV, and ATR-SEIRAS further shows an increasing $^*OOH$-related signal under OCV. Taken together, these results directly support the conclusion that $^*OOH$ continues to form and accumulate during the OCV process, which is the key experimental foundation for the OCV–PV method.

We agree that the Ea variation becomes less pronounced in the high-*O-coverage region. However, the key point of Figure 1e is that, with the emergence of the internal electric field on the charged NiOOH surface, the $^*OOH$ formation barrier drops rapidly to below 0.75 eV and then decreases more gradually at higher field strength. Thus, the physically relevant implication is that $^*OOH$ formation becomes kinetically accessible once a sufficiently charged surface state is established.

To address the reviewer's concern, we have revised the relevant text in the manuscript as follows (line 143, page 4, highlighted in yellow):

These results provide a theoretical basis for the proposed OCV concept.

4. What is the fundamental mechanism by which distinct dopant elements selectively facilitate specific elementary steps in the OER process? This mechanistic aspect remains insufficiently elucidated in current version.

Response: We thank the reviewer for this important comment. We agree that the previous version did not sufficiently clarify the mechanistic basis by which different dopant elements selectively facilitate different elementary steps in OER.

In the present work, the primary role of the OCV–PV/PV methodology is to resolve step-specific kinetic fingerprints across a broad dopant series, rather than to assign a fully atomically resolved mechanism to every individual dopant. Nevertheless, the results reveal a clear mechanistic principle: different dopants

selectively perturb different parts of the Ni–O reaction coordinate within the NiOOH lattice, and therefore influence different elementary steps to different extents.

Specifically, our results show that Fe predominantly accelerates the $^*O \rightarrow {}^*OOH$ step, whereas Mn more effectively facilitates the $^*OH \rightarrow {}^*O$ deprotonation step. For Fe, this interpretation is supported by the observed enhancement of $^*OOH$ formation kinetics together with our previous understanding that higher Fe incorporation induces stronger e_g^* band broadening (*Nature Communications, 2023, 14(1): 7488.*), which is associated with more favorable electronic structure for electron transfer from the electrolyte to the catalyst. In addition, the DFT calculations on $Ni_{1-x}Fe_xOOH$ show that the $^*OOH$ formation barrier decreases with increasing Fe content (Figure R3-2). By contrast, Mn does not produce the strongest enhancement in $^*OOH$ formation, but more effectively improves the $^*OH$ deprotonation kinetics, indicating that its primary effect is on a different elementary reaction coordinate.

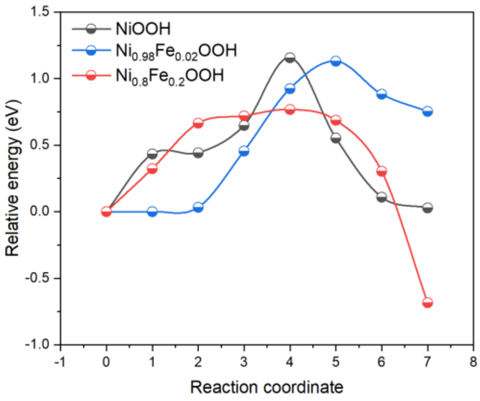

Figure R3-2. Simulation on the $^*OOH$ formation barriers for NiOOH, $Ni_{0.98}Fe_{0.02}OOH$, and $Ni_{0.8}Fe_{0.2}OOH$. (Supplementary Figure 37 in revised manuscript)

Therefore, the mechanistic picture emerging from this work is that dopants act as selective perturbations to step-specific kinetics within a Ni-centered oxyhydroxide lattice, rather than as uniform promoters of the overall OER. We have revised the manuscript accordingly to clarify that this study establishes a dopant-selective kinetic framework, while direct atomic-scale identification of the microscopic mechanism for each dopant remains beyond the scope of the present work.

To address the reviewer's concern, we have revised the relevant text in the manuscript as follows (line 326, page 10, highlighted in yellow):

These results suggest that different dopants selectively perturb different parts of the Ni–O reaction coordinate within the NiOOH lattice, thereby facilitating different elementary OER steps to different extents.

5. Although macroscopic performance data suggest that Fe primarily facilitates the formation of OOH*, while Mn enhances the deprotonation of OH*, the precise OER mechanism in NiOOH catalysts co-doped with Fe and Mn remains incompletely elucidated. Key mechanistic questions persist, including the exact location of active sites responsible for O* and OOH* formation, as well as the atomic-scale structural configuration of the Fe-Mn dopant motif.

Response: We thank the reviewer for this important comment. We agree that the precise atomic-scale OER mechanism in Fe/Mn co-doped NiOOH, including the exact active-site locations for *O and *OOH formation and the detailed Fe–Mn dopant configuration, is not yet fully resolved.

In the present work, our intention is not to assign the OER activity to a unique atomically identified Fe, Mn, or Ni site. Rather, based on the OCV–PV/PV framework, we resolve the apparent step-specific kinetic roles of the dopants under identical conditions. Within this experimentally derived framework, Fe is identified to predominantly promote *OOH formation, whereas Mn more effectively facilitates *OH deprotonation, and their combination enables simultaneous improvement of the two key kinetic processes in OER.

At the mechanistic level, these results suggest that Fe and Mn likely act by selectively modifying the local electronic environment of the Ni–O framework, thereby perturbing different parts of the reaction coordinate to different extents. In this sense, the mechanistic conclusion of the present work is made at the level of experimentally measured step-specific kinetic behavior, rather than atomically resolved active-site structure. This interpretation is also consistent with the Ni-centered redox framework used throughout the manuscript to describe OER in NiOOH-based catalysts. Direct clarification of the local Fe–Mn motif and the exact sites for *O and *OOH formation would require dedicated operando structural studies. We have therefore revised the manuscript to clarify that our conclusions are made at the level of experimentally resolved step-specific kinetics rather than atomically identified active-site structures.

We have revised the relevant text in the manuscript as follows (line 345, page 10, highlighted in yellow):

Here, the roles of Fe and Mn are discussed at the level of experimentally resolved apparent step-specific kinetics, rather than at the level of atomically identified active-site motifs. The present results therefore do not directly resolve the local Fe–Mn dopant configuration or the exact sites for $^*O$ and $^*OOH$ formation, which would require dedicated operando structural characterization.

6. What impact does the increase in Fe content have on the structure of the catalyst? Will the increase in Mn content also enhance the OER activity?

Response: We thank the reviewer for this comment. In our previous work, we have systematically investigated the Fe dopant effect on Ni (oxy)hydroxides (*Energy & Environmental Science, 2023, 16(2): 641-652.; Nature Communications, 2023, 14(1): 7488.; Applied Catalysis B: Environment and Energy, 2024, 344: 123674.*).

It is shown that the main structural difference is the degree of NiO_6 octahedron distortion, leading to various extent of e_g^* band broadening around the Fermi level with the order: $Ni_{0.8}Fe_{0.2}OOH$ > $Ni_{0.95}Fe_{0.05}OOH$ > NiOOH (Figure R3-3, *Nature Communications, 2023, 14(1): 7488.*).

[FIGURE REDACTED]

Figure R3-3. **a** Models of NiOOH with and without Fe dopant to show the Ni-O bond extension along *z* axis. **b** The calculated e_g^* band Ni partial density of states (PDOS) of $Ni_{1-x}Fe_xOOH$ ($x = 0, 0.05, 0,2$). **c** Raman spectra of the $Ni_{1-x}Fe_xOOH$ ($x = 0, 0.05, 0,2$) to compare the intensity ratio between peak at 472 $cm^{-1}$and 555 cm^{-1} (I_{472}/I_{555}). **d** FT-EXAFS spectra of Ni *K* edges of $Ni_{1-x}Fe_x(OH)_2$ (x = 0, 0.05, 0.2), inset showing the enlarged FT results within the range of 1.5 to 1.75 Å. **e** The correlation between the Ni *K*-edge white line intensity with the energy difference of $d_{x^2-y^2}$ and d_{z^2} orbitals for $Ni_{1-x}Fe_xOOH$ ($x = 0, 0.05, 0.2$). It should be noted that the white line intensity of $Ni_{1-x}Fe_x(OH)_2$ (x = 0, 0.05, 0.2) extracted from Ni *K*-edge XAS (*Energy & Environmental Science, 2023, 16(2): 641-652.*) is used to evaluate the e_g^* band broadening for $Ni_{1-x}Fe_xOOH$ (x = 0, 0.05, 0.2) to avoid the possible effects on the XAS results caused by the self-discharge of Ni^{3+}.

Theoretically, the stronger e_g^* band broadening would trigger the emergence of more electronic states around the Fermi level. This effect would notably facilitate the electron transfer between electrolyte and electrocatalysts, which has been validated through the OCV-PV test. To further support this, we conduct DFT calculations to investigate the *OOH formation energy barriers on $Ni_{1-x}Fe_xOOH$ (x = 0, 0.02, 0.2). We find that the energy barrier for *OOH formation is greatly decreased with higher Fe dopant concentration, showing the order: NiOOH> $Ni_{0.98}Fe_{0.02}OOH$ > $Ni_{0.8}Fe_{0.2}OOH$. (Figure R3-4)

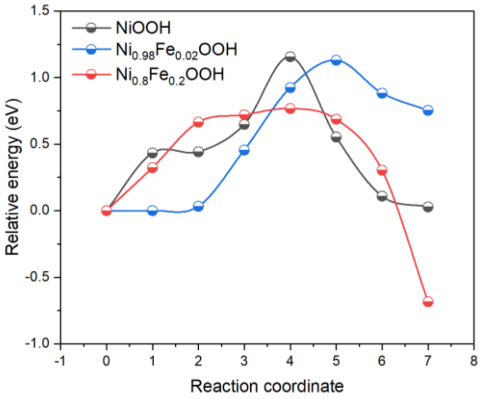

Figure R3-4. Simulation on the *OOH formation barriers for NiOOH, $Ni_{0.98}Fe_{0.02}OOH$, and $Ni_{0.8}Fe_{0.2}OOH$. (Supplementary Figure 37 in revised manuscript)

Based on our step-resolved kinetic framework, a further increase in Mn content may in principle provide additional enhancement in OER activity, because Mn primarily facilitates the *OH deprotonation process. However, such an improvement is expected to be limited, rather than necessarily monotonic, because *OOH formation is identified here as the RDS, whereas *OH deprotonation acts only as a secondary kinetic process under certain conditions. Therefore, once *OH deprotonation has been sufficiently accelerated, further increasing Mn content may provide only marginal benefit unless the *OOH formation step is simultaneously optimized.

To address this comment, we have revised our manuscript as follows (line 352, page 10, highlighted in yellow):

Our previous work reveals that the increase in Fe content can lead to stronger NiO_6 distortion, which is associated with enhanced e_g^* band broadening. Such electronic-structure modulation is expected to increase the density of states near the Fermi level and thereby facilitate electron transfer between the electrolyte and the electrocatalyst. To further support this interpretation, we performed DFT calculations on $Ni_{1-x}Fe_xOOH$

(x = 0, 0.02, and 0.2) to evaluate the energy barrier for *OOH formation. The calculated barrier decreases with increasing Fe content, following the order NiOOH > $Ni_{0.98}Fe_{0.02}OOH$ > $Ni_{0.8}Fe_{0.2}OOH$ (Supplementary Figure 37). This trend agrees with the OCV–PV results, supporting that higher Fe incorporation facilitates *OOH formation in NiFeOOH.

(line 366, page 11, highlighted in yellow): In principle, further increasing Mn content may provide additional benefit; however, such improvement is expected to be limited once *OOH formation remains the dominant RDS in the present framework, whereas *OH deprotonation serves as a secondary kinetic process.

7. The electrical double layer plays a pivotal role in governing electrochemical reaction mechanisms. For example, the structural arrangement of interfacial water molecules significantly influences both the kinetics of water dissociation and the mass transport of key species (OH- and O2). The structural impact of metal dopant incorporation on interfacial water remains unconfirmed.

Response: We thank the reviewer for highlighting the possible influence of interfacial water structure. We agree that the organization of interfacial water molecules can influence proton-coupled electron transfer and thus influence both water dissociation kinetics and species transport.

Regarding possible mass-transport contributions, we minimized their influence through the design of the OCV–PV protocol. In our measurements, the OCV interval is intentionally kept short (0.8–1.8 s), so that the extracted charge evolution primarily reflects the initial kinetic response rather than a fully developed diffusion-limited regime. Consistently, the approximately linear ΔQ–t relationship within this short time window supports the use of this region for kinetic analysis. In addition, all measurements were performed in 1 M KOH, where OH^- is abundant, and the brief OCV interval corresponds to a transient relaxation period without sustained anodic oxygen evolution, making significant O_2 accumulation unlikely under the present conditions.

We also agree that metal dopants may modify the interfacial environment, including the structure of interfacial water. However, the present work does not directly probe interfacial water structure, and therefore we do not assign the dopant effect to this specific microscopic origin. Rather, our intention is to use the OCV–PV/PV approach to provide a comparative, step-resolved kinetic analysis under identical electrolyte conditions. In this sense, any dopant-induced interfacial-water effect, if present, would be incorporated into

the measured apparent kinetics. Therefore, our conclusions are limited to the experimentally observed differences in step-specific OER kinetics among doped catalysts, rather than to a direct identification of interfacial-water restructuring as the dominant origin.

To avoid overstating this point, we have revised the manuscript to clarify that the dopant-dependent kinetic differences revealed by OCV–PV/PV may include contributions from changes in the interfacial environment, and that direct verification of interfacial-water restructuring would require dedicated operando spectroscopic investigation in future work.

We have revised our manuscript as follows (line 331, page 10, highlighted in yellow): We emphasize that these dopant-dependent differences are identified at the level of experimentally measured apparent step-specific kinetics. The present results do not distinguish whether such effects originate predominantly from catalyst electronic-structure modulation, from changes in the interfacial solvation/electrical-double-layer environment, or from their combined contributions. Direct verification of dopant-induced restructuring of interfacial water would require dedicated *operando* interfacial spectroscopy and is beyond the scope of the present work.

Minor comment:

The captions for Figures 1d and 1e are missing.

Response: We thank the reviewer for this comment. The captions have been added in the revised manuscript.

To address the comment, we have revised our manuscript as follows (line 109, page 4, highlighted in yellow): **d** The effect of *O concentration on internal electric field. **e** The effect of internal electric field on *OOH formation energy barrier

Response to referees

We thank the three referees for taking the time to carefully review the manuscript and for giving many useful suggestions. The manuscript has been revised according to their comments and the changes have been highlighted in the revised version. We feel the quality of the paper has been improved greatly thanks to the input from the referees. Below is a point-by-point response.

Reviewer #2

Although authors have performed the revision carefully, to make the manuscript complete, a few additional data may be provided in the revised manuscript. The specific comments are:

1.The authors should add the standard diffraction data card in the PXRD data presented in the supplementary file.

Response: We thank the reviewer for this comment. We have now added the standard diffraction data card of the parent $Ni(OH)_2$ phase (PDF #38-0715) to the PXRD patterns in the Supplementary Information, as shown in Figure R2-1 (Supplementary Fig. S21 in the revised manuscript). The reference diffraction lines are displayed at the bottom of the figure, together with the indexed major reflections of $Ni(OH)_2$. We note that slight peak shifts relative to the standard diffraction card are observed across the entire sample series, including the undoped $Ni(OH)_2$ reference. Such deviations are common for substrate-supported hydroxide films and do not indicate impurity phase formation. Importantly, no additional reflections or peak splitting associated with secondary crystalline phases are observed.

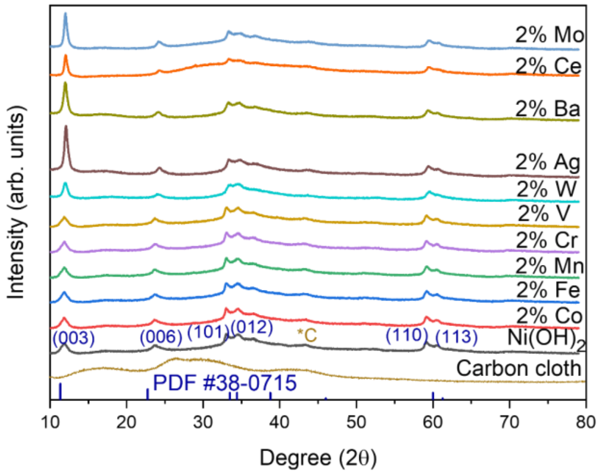

Fig. R2-1. XRD patterns of $Ni(OH)_2$-based catalysts with various cation dopants (2 at%). (Supplementary Figure 22 in revised manuscript)

To address the comment, we have revised our manuscript as follows:

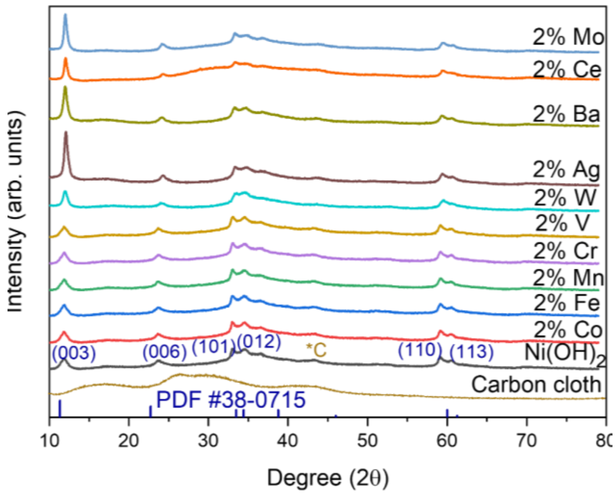

Supplementary Figure 22. XRD patterns of $Ni(OH)_2$-based catalysts with various cation dopants (2 at%).

We note that slight peak shifts relative to the standard diffraction card are observed across the entire sample series, including the undoped $Ni(OH)_2$ reference. Such deviations are common for substrate-supported hydroxide films and do not indicate impurity phase formation. Importantly, no additional reflections or peak splitting associated with secondary crystalline phases are observed.

2. Authors have explained the *OO evolution in addition to Ni4+ to Ni3+ conversion. It is still unclear from the scheme and also from the text how this step is performed for calculation. Provide some detailed explanation of the relevant steps.

Response: We thank the reviewer for this comment. We agree that the previous version did not explain clearly enough how the *OO evolution contribution was incorporated into the calculation.

In our OCV-PV framework, the total OCV-induced charge is obtained from the charge difference between the two PV cycles, (Q_1-Q_2). As discussed in the manuscript, this total charge reflects the apparent sum of all electrolyte-to-catalyst electron-transfer events occurring during OCV. To express the result on an event-equivalent basis, (Q_1-Q_2) is divided by 2. This gives the total apparent number of reaction events contributing during the OCV period.

Within this framework, the *OO evolution pathway is treated as a local OCV-period relaxation step in which a pre-formed *OO related species is released as O_2 during OCV. Each such *OO evolution event is

accompanied by one local electron transfer to a neighboring Ni^{4+} site, reducing it to Ni^{3+}. Therefore, the O_2 amount detected by DEMS during OCV is converted to the corresponding *OO-evolution-related contribution on the same event-equivalent basis as $(Q_1$-$Q_2)/2$, *i.e.*, $Q(^*OO)= F\ n_{O2}$, where n_{O2} is the O_2 amount detected within the OCV window, obtained from the calibrated DEMS signal (Fig.R2-2).

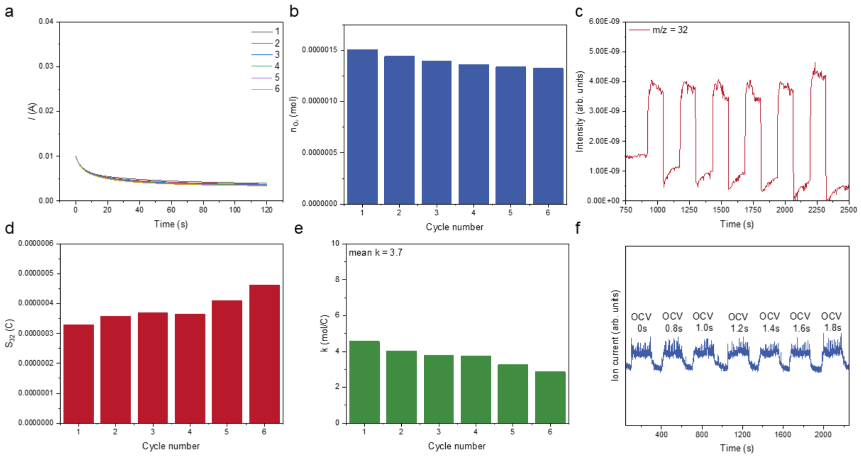

Fig. R2-2. **Calibration of the DEMS response and OCV-time-dependent DEMS measurements.** (a) *I-t* curves recorded at 1.503 V vs. RHE for six repeated calibration cycles. (b) O_2 amount ($n(O_2)$) estimated from the integrated electrochemical charge in each calibration cycle. (c) Simultaneously recorded DEMS signal at m/z=32 during the calibration cycles. (d) Integrated baseline-corrected DEMS m/z=32 signal (S_{32}) for each calibration cycle. (e) Effective DEMS calibration factor k obtained from the corresponding electrochemical and DEMS integrals, with an average value of k=3.7 mol/C (f) DEMS m/z=32 signal recorded under identical pre-charging conditions while varying only the OCV duration (0, 0.8, 1.0, 1.2, 1.4, 1.6, and 1.8 s), used to evaluate the additional O_2 signal develops with increasing OCV time.

Accordingly, the *OOH-related charge used for kinetic analysis is calculated as: $Q(^*OOH) = [(Q_1$-$Q_2)/2$-$Q(^*OO)]$. That is, the calibrated DEMS signal first gives the O_2 amount detected during OCV, this O_2 amount is then converted into the corresponding *OO-evolution-related event contribution, and this term is subtracted from the total apparent OCV charge to obtain the *OOH-related contribution.

To address the comment, we have revised our manuscript as follows:

(line 251, page 8, highlighted in yellow): Moreover, the contribution of the *OO evolution step was determined using *in situ* DEMS (details on the DEMS procedure are provided in the Methods section).

Specifically, the calibrated DEMS signal measured within the OCV window was first converted to the amount of O_2 detected during OCV. This O_2 amount was then converted to the corresponding *OO evolution related charge contribution. As shown in Figure 3c and Supplementary Figure 15, compared with the total charge (Q_1-Q_2)/2 obtained from the OCV–PV measurement, the recorded charge associated with *OO evolution is significantly lower and therefore does not dominate the extracted OCV kinetics, which agrees well with our hypothesis that *O is the most abundant intermediate in the formation of *OOH during the OCV.

(line 461, page 13, highlighted in yellow): **DEMS measurement.** Differential Electrochemical Mass Spectrometry (DEMS) was used to quantify the evolved oxygen molecules within the OCV window at different OCV durations during the OCV-PV measurement. Specifically, after electrochemical activation, the catalyst was held at 1.503 V under steady OER conditions, and the electrochemical charge passed during the steady-state interval was converted to the corresponding O_2 amount using Faraday's law $n(O_2) = Q_{cell}/4F$ (Supplementary Figure 15). The simultaneously recorded, baseline-corrected DEMS m/z = 32 signal was integrated over the same time window to obtain the detector response, from which an effective molar sensitivity was determined as k=3.7 mol/C. This calibration factor was then applied to the OCV-period DEMS signal to estimate the amount of O_2 detected within the OCV window. Within the present mechanistic framework, this O_2 amount was converted to the corresponding *OO evolution related charge contribution according to $Q(^*OO) = F\, n(O_2)$.

3. In figure 3b, how did the author explain the initial discharging current. What does it contribute to? An explanation will be better to visualize the results obtained.

Response: We thank the reviewer for this comment. In Figure 3b, the initial discharging current appears immediately after switching from the charging bias to the cathodic pulse in the PV step. This current corresponds to the designed cathodic readout response of the pre-charged NiOOH surface and reflects the reduction of oxidized Ni species.

To address the comment, we have revised our manuscript as follows: (line 244, page 8, highlighted in yellow): The initial discharging current observed upon switching to the cathodic pulse corresponds to the designed cathodic readout response of the pre-charged NiOOH surface and reflects the reduction of oxidized Ni species.

4. What is the calculated (actual) Faradaic efficiency of the proposed method? A detailing would be beneficial for the readers. Please include it.

Response: We thank the reviewer for this comment. We note that the conventional Faradaic efficiency (FE) definition is not strictly applicable to the OCV–PV method, because the present protocol is designed to quantify the apparent charge contribution associated with *OOH formation, *i.e.*, an intermediate-forming step, rather than the yield of a final stable product under steady-state electrolysis.

To address the reviewer's concern, we instead define an apparent Faradaic efficiency for the OCV-derived *OOH signal as the fraction of the total apparent OCV-induced charge that remains after excluding the *OO-evolution-related contribution determined by calibrated DEMS:

$$FE = Q(^*\mathrm{OOH})/[(Q_1 - Q_2)/2] * 100\%$$

where $Q(^*\mathrm{OOH}) = (Q_1 - Q_2)/2 - Q(^*\mathrm{OO})$, and $Q(^*\mathrm{OO})$ is the *OO evolution related contribution estimated from the calibrated DEMS signal. Based on this analysis, the *OO evolution related contribution is much smaller than the total apparent OCV-induced charge, giving an apparent FE toward *OOH formation of >98% for all OCV durations tested.

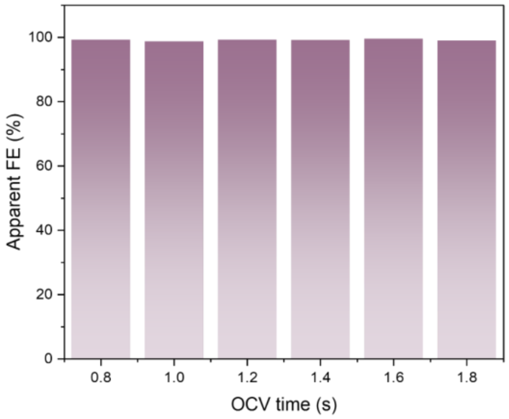

Fig. R2-3. Apparent FE toward *OOH formation at different OCV durations for NiOOH. The charging bias is 1.503 V vs. RHE. (Supplementary Figure 16 in revised manuscript)

We have clarified in the revised manuscript that this quantity should be interpreted as an apparent FE for the OCV-period charge assignment, rather than a conventional product FE in steady-state electrocatalysis.

To address the comment, we have revised our manuscript as follows: (line 258, page 8, highlighted in yellow):

Based on this charge partition, the apparent Faradaic efficiency toward $^*OOH$ formation exceeds 98% across the investigated OCV durations (Supplementary Figure 16), indicating that the OCV-derived charge is predominantly associated with $^*OOH$ formation rather than $^*OO$ evolution.
